# Ethnobotanical uses of plants in Nigeria: an analysis of current research trends and patterns

**DOI:** 10.1186/s13002-025-00788-y

**Published:** 2025-08-21

**Authors:** Basirat O. Rafiu, Abiodun O. Omotayo, Ibraheem O. Lawal, Adeyemi O. Aremu

**Affiliations:** 1 Indigenous Knowledge Systems Centre, Faculty of Natural and Agricultural Sciences, North-West University, Private Bag X2046, Mmabatho, 2790 South Africa; 2 Biomedicinal Research Centre, Forestry Research Institute of Nigeria, PMB 5054, Jericho, 200272 Ibadan Nigeria; 3 Food Security and Safety Niche Area, Faculty of Natural and Agricultural Sciences, North-West University, Private Bag X2046, Mmabatho, 2790 South Africa; 4 Federal College of Forestry, Forestry Research Institute of Nigeria, PMB 5054, Jericho, 200272 Ibadan Nigeria; 5 School of Life Sciences, College of Agriculture, Engineering and Science, University of KwaZulu-Natal, Durban, South Africa

**Keywords:** Agro-ecology, Biodiversity, Conservation, Fabaceae, Healthcare, Indigenous knowledge

## Abstract

**Background:**

The ethnobotanical landscape in Nigeria reflects a complex interplay of biodiversity, cultural traditions, and health practices deeply rooted in indigenous knowledge. However, the fragmented body of knowledge in the existing limited inventory poses a threat to the loss of these plants and the associated indigenous knowledge. This review examined the historical and cultural uses of plants, as well as their long-term utilisation patterns.

**Methods:**

Using a systematic search, the Web of Science, Scopus, and Dimensions were explored to gather ethnobotanical literature on utilisation of plants in Nigeria from 1964 to 2024. In addition, bibliometric tools were applied to establish the research trends and patterns.

**Results:**

From the 79 eligible studies, we collated 963 plants across 144 families (dominated by Fabaceae with 127 plants) utilised in seven categories namely medicinal/healthcare, spiritual, cosmetics, biopesticides, poison, timber, and fuelwood. An estimated 11% (103) of the 963 plants were classified as popular, with mentions ranging from 11 to 42 times, and the three top cited plants were *Carica papaya* (42), *Vernonia amygdalina* (42) and *Mangifera indica* (41). Most of the identified notable plants (e.g., *Azadirachta indica*, *Carica papaya*, *Mangifera indica*, and *Vernonia amygdalina*) had significant geographic and cultural relevance. The spiritual, medicinal, and socio-economic roles of these plants were intricately influenced by ethnic identities, ecological zones, and religious beliefs. Cross-regional and cross-cultural analyses revealed that the Southwest and Northeast regions accounted for the highest (70.98%) and lowest (10.28%) prevalence of plant use in Nigeria. Generally, plant utilisation patterns varied across regions, with certain shared uses identified thereby highlighting inter-ethnic connections, while others revealed localised traditions. We identified 19 plants (e.g., *Azadirachta* indica, *Mangifera indica*, *Vachellia nilotica* and *Ximenia americana*) that were common to the six regions in Nigeria. Bibliometric analysis revealed that the evolution of plant research in Nigeria, from traditional knowledge to more specialised molecular and applied research methodologies. We defined three distinct timelines associated with ethnobotany in Nigeria entailing the initial phase associated with the fundamental period (1964–1989), expansion and growth with shifting focus in research (1990–2009) which was followed by the modernisation and integration (2010–2024).

**Conclusion:**

Ethnobotanical research in Nigeria remains active, reflecting the extensive uses of plants to meet the daily needs of local communities. Nonetheless, challenges persist, including insufficient documentation of indigenous practices, limited collaboration, instances of non-adherence to best practices in ethnobotanical surveys. Addressing these challenges is crucial for the sustainable management of the ethnobotanical heritage in Nigeria.

**Supplementary Information:**

The online version contains supplementary material available at 10.1186/s13002-025-00788-y.

## Introduction

Nigeria boasts a rich pool of plants, each often intertwined with distinct applications, rituals, and cultural beliefs across the diverse ethnic communities [1]. The country is characterised by high biodiversity and cultural diversity [2, 3], with numerous plants supporting the livelihoods of millions [4]. Recognising the value of traditional knowledge associated with these plants [5], and the contributions of local communities is essential for conserving the biodiversity and cultural heritage [6, 7]. Particularly, these plants offer natural-based treatments for diseases such as malaria, diabetes, and cancer. Local communities are often instrumental in safeguarding and sharing this knowledge, facilitating its passage across generations [8]. The understanding of ethnobotanical practices enhances cultural traditions and medicinal customs, exemplified by notable plants such as bitter leaf (*Vernonia amygdalina*) within Igbo culture and kola nut (*Cola acuminata* and* C*. *nitida*) among the Yorubas [9]. For health purpose, plants such as *Morinda lucida, Azadirachta indica,* and *Psidium guajava* are useful for treating malaria, diabetes, and gastrointestinal disorders [10–12]. Furthermore, local communities utilise *Elaeis guineensis*, *Vitellaria paradoxa*, and *Moringa oleifera* to meet diverse health needs [4, 13]. Plants such as *Carica papaya*, *Vernonia amygdalina*, *Ageratum conyzoides*, and *Chromolaena odorata* have demonstrated their importance for the general well-being and survival of the inhabitants in some communities [14–18].

Cultural practices often intersect with traditional knowledge, offering valuable insights into the sustainable use of plant resources [4]. This, in turn, fosters human well-being and environmental sustainability [18]. In Nigeria, plants such as *Guiera senegalensis*, *Parkia biglobosa, Adansonia digitata, Cola nitida, Cola acuminata, Elaeis guineensis,* and *Chrysophyllum albidum,* generally hold significant cultural value [19]. *Guiera senegalensis* plays multiple roles in cultural practices, including burial rituals, and ensuring safe journeys, highlighting the strong connection between plants and cultural traditions [20]. Across communities in Nigeria, *Adansonia digitata* is revered for its sacred nature and is believed to hold spiritual powers [21]. The utilisation of palm fronds from *Elaeis guineensis* holds a crucial place in various religious and cultural ceremonies, including traditional marriage rites and chieftaincy coronations [22].

The profound comprehension of plant ecosystems allows for the formulation of judicious decisions that enhance sustainable resource management and the preservation of biodiversity [23]. Indigenous practices are essential for the protection of plants using traditional knowledge, which in turn supports the conservation of biodiversity and enhances the resilience of cultural communities [24]. It remains pertinent to enhance traditional knowledge by uncovering new uses and benefits of these plants while promoting sustainable resource use by developing eco-friendly cultivation methods [25]. However, challenges including the increasing demand for plants due to their therapeutic properties, rapid population growth in regions where these plants are endemic, and environmental degradation from unsustainable harvesting practices, pose significant threats to their conservation [26–28]. The potential extinction of valuable plants highlights the urgent need for conservation efforts. Thus, the need for research effort and action to implement sustainable harvesting practices, establishing protected areas, and engaging local communities in conservation initiatives to safeguard biodiversity and cultural heritage [29, 30].

This review delves into ethnobotanical activities among the ethnic groups in Nigeria, with a focus on the link between their cultural beliefs and plant uses. Furthermore, using a bibliometric approach, the significance of ethnomedicine in the Nigerian healthcare system and trends in ethnobotanical studies from 1964 to 2024 were applied to identify the knowledge gaps, research themes, and underexplored areas. This study explored the historical and cultural applications of plants in Nigeria, as a means of understanding the current research status and trends from an ethnobotanical perspective. 

## Methods

### Data extraction

The Web of Science, Scopus, and Dimension were selected as the data sources due to their extensive coverage of relevant scientific studies in the field of ethnobotany. Following the Preferred Reporting Items for Systematic Reviews and Meta-Analyses (PRISMA) guidelines [31], a search of these electronic databases was done in June 2024 to retrieve relevant studies on the ethnobotanical uses of plants in Nigeria. Additionally, we assessed theses, dissertations, and books from the Library of the Forestry Research Institute of Nigeria (FRIN). Phrases related to “ethnobotany,” “ethnobotanical studies,” “plant conservation,” “plant species,” and “Nigeria” were used as search terms to find relevant papers in the databases. Furthermore, the bibliographies of the retrieved articles were searched and saved in the EndNote reference manager.

We applied bibliometric analysis which serves as a quantitative examination of research trends, publication patterns, and collaboration networks [32]. This was essential to establish publication trend analysis, co-authorship and institutional networks, keyword analysis, thematic analysis, and word analysis. These method assists in identifying areas where knowledge is insufficient, which may pivotal for funding and policy decisions in the field [32].

### Inclusion and exclusion criteria

The retrieved articles were screened according to the predefined inclusion and exclusion criteria (Table 1). The inclusion criteria consisted of studies conducted in Nigeria from January 1, 1964, to June 30, 2024; scientific articles published in English; and studies addressing ethnobotanical uses, phytochemistry, and the conservation status of plant species in Nigeria, ensuring a comprehensive selection. The exclusion criteria included studies not written in English, review articles, studies conducted outside Nigeria, studies published before 1964, and studies that did not address ethnobotanical uses, phytochemistry, or the conservation status of plant in Nigeria. The search for “ethnobotanical studies in Nigeria” was conducted within the three selected databases to ensure comprehensive coverage and retrieval of relevant literature. We gathered information about ethnobotanical uses, plant identification, preparation methods, and application routes from the eligible studies.

**Table 1:** Criteria applied for the inclusion of articles in this systematic literature review

Search result	Reasons for acceptance	Reasons for rejection
Initial check	Studies published in English	Studies with no scientific names and not written in English
	Articles on ethnobotanical studies and plant utilisation	Articles without full-text: (e.g. conference abstract)
Title with abstract screening	Articles related to ethnobotanical studies focusing on Africa	Articles with irrelevant design
	Studies published in 1964–2024	Extraneous subjects unrelated to the main theme
	Articles addressing plant utilisation in Nigeria with scientific names, and relevant to the aims of the review	Articles that do not meet the inclusion criteria (review articles, Newspapers and books)
Full paper review	Original studies	Articles that lack sufficient detail or necessary data and were written before 1964
	Articles that were unique versions of other articles	
	Studies available on the ISI Web of Knowledge	Duplicate articles or variants of the same study were detected and removed
	Adaptation options under different plant utilisation scenarios	Articles that contravene ethical rules, such as plagiarism or data fabrication

### Data analysis

**Fig. 1:**
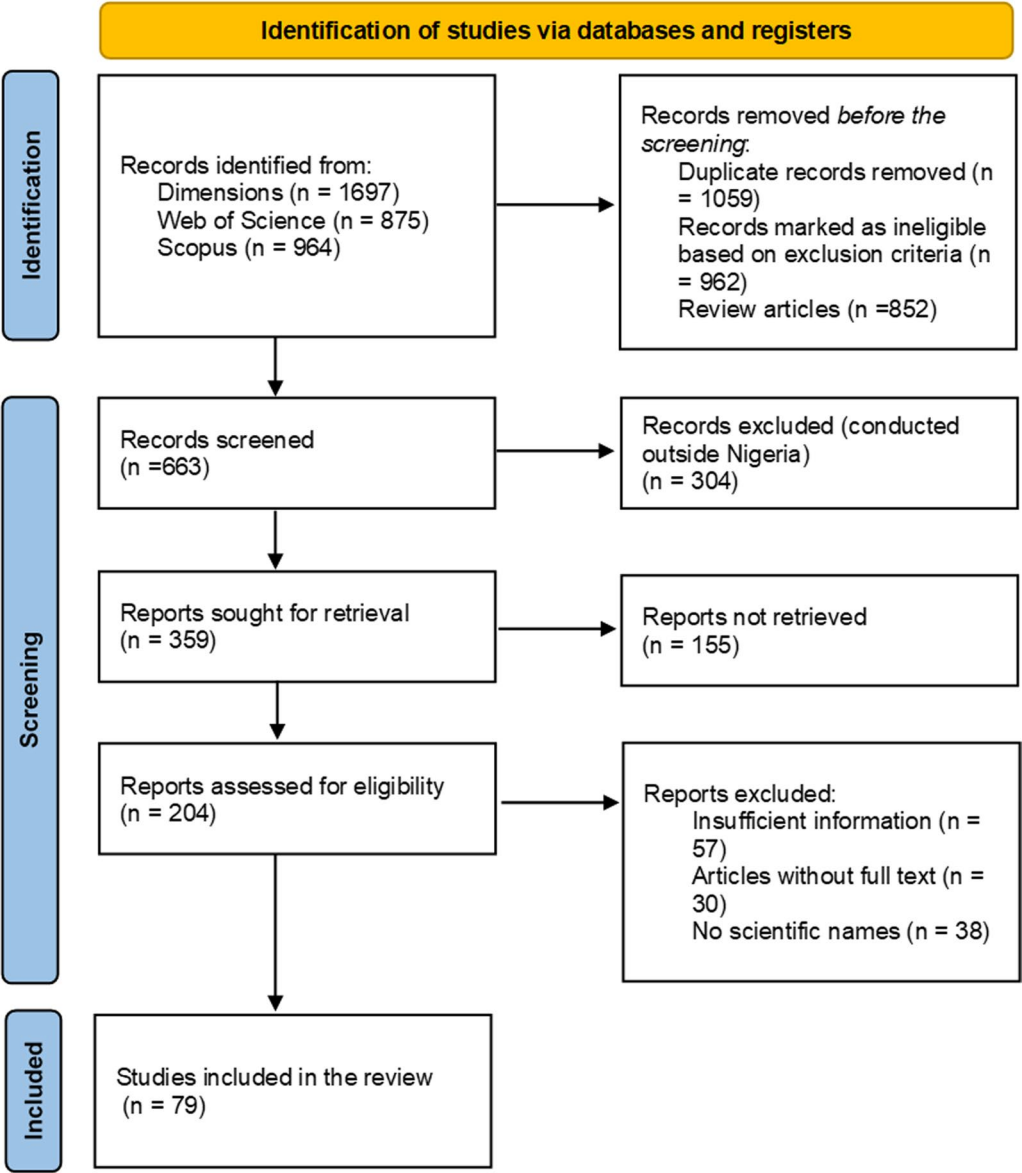
Procedure applied for literature search for the ethnobotanical uses of plants in Nigeria from 1964 to 2024 based on the Preferred Reporting Items for Systematic Reviews and Meta-Analyses (PRISMA) statement

A bibliometrics data analysis spanning from 1st January 1964 to 30th June 2024 was conducted to identify the plant species and their ethnobotanical applications, as well as to establish the trends in research on uses of plant species in Nigeria. The study retrieved 3536 studies from various scientific databases, comprising 1697, 875 and 964 from Dimensions, Web of Science and Scopus, respectively. The titles were examined to eliminate duplicates, and abstracts were systematically evaluated for relevance. Ultimately, 79 articles were eligible with concise focus on ethnobotanical studies (Fig. 1).

The Critical Appraisal Skills Programme tool was used to assess the quality of the included studies. As outlined by Long, French [33], 10 questions were used to evaluate the methodological quality of qualitative studies. After assessing the content in the articles, the questions were answered as Yes, No, or Uncertain (Supplementary material 1).

### Results and discussion

The review outputs from the current endeavour are presented from two core perspectives entailing the systematic (ethnobotany in Nigeria) and bibliometric (patterns and trends in ethnobotanical research in Nigeria) assessments.

### Ethnobotany in Nigeria

Following the systematic search, the current research status on Nigerian ethnobotany was established. This aspect addressed the critical appraisal of the eligible ethnobotanical field surveys and their associated implications.

#### Attributes and quality assessment of the 79 eligible ethnobotanical studies

**Fig. 2:**
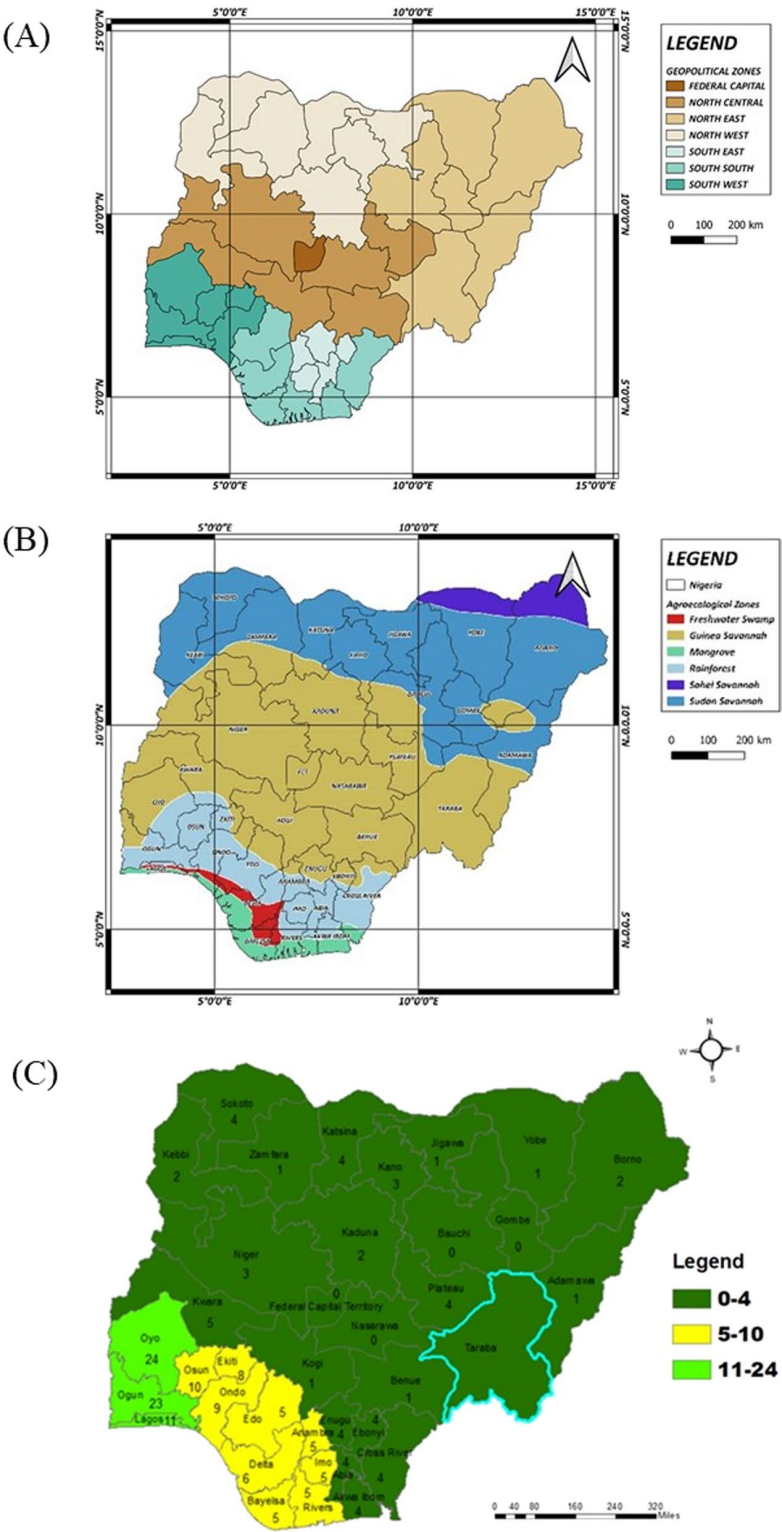
Geography of Nigeria. **A** Geographical delineation of the six regions in Nigeria; **B** Agroecological zones in Nigeria; **C** Distribution of the 79 eligible ethnobotanical studies across the states in Nigeria

A total of 79 eligible literature documenting local knowledge of plants in Nigeria were recorded (Table 2). As depicted in Fig. 2A–C, there has been extensive ethnobotanical research conducted across the six regions in Nigeria, namely North-Central (NC), Northeast (NE), Northwest (NW), Southeast (SE), South-South (SS) and Southwest (SW). The eligible studies entailed invaluable traditional knowledge and practices related to the uses of plants in Nigeria. Each entry stands as a significant contribution to the understanding of conventional plant knowledge in Nigeria, particularly in the realms of healthcare, indigenous and cultural practices. We recorded a wide range of documented plant species, with some studies reporting up to 208 plants across 68 plant families (Table 2), thereby underscoring the remarkable biodiversity in Nigeria.

**Table 2:** Overview of the 79 publications that met the criteria on the use of plants in Nigeria from 1964 to 2024

S/N	Author	Title of the article	Region(s) in Nigeria	Method	Number of plants	Number of plant families	Type of participants	Voucher specimen
1	Abdallah, Mustafa [11]	Ethnobotanical knowledge of the most commonly used plants in the management of gastrointestinal ailments in Yobe State, Nigeria	NE	Semi-structured questionnaires	23	15	Traditional healers	Department of Plant Biology, Herbarium, Bayero University Kano, Nigeria
2	Abo, Fred-Jaiyesimi [39]	Ethnobotanical studies of medicinal plants used in the management of diabetes mellitus in Southwestern Nigeria	SW	Interviews using pilot-tested questionnaires	31	24	Traditional healers	Forest Herbarium Ibadan and Herbarium of Faculty of Pharmacy, Olabisi Onabanjo University, Shagamu
3	Abubakar, Yusuf [35]	Ethnopharmacological survey of medicinal plants used for the management of pediatric ailments in Kano State, Nigeria	NW	Informal interviews	68	36	Traditional medicine practitioners, traditional birth attendants, herb sellers and some health workers	Ethnobotany unit of Bioresources Development Centre Kano, National Biotechnology Development Agency (NABDA), Nigeria
4	Abubakar, Ukwuani-Kwaja [38]	Ethnobotanical study of medicinal plants used for cancer treatment in Kebbi state, North-west Nigeria	NW	Oral interviews using structured questionnaires	48	25	Herbal medicine practitioners	Herbarium of Kebbi State University of Science and Technology, Aliero
5	Abubakar [157]	An inventory of medicinal plants used for treatment of cancer in Kwara and Lagos State, Nigeria	SW, NC	Oral interviews using structured questionnaires	41	28	Herbal medicine practitioner	University of Ilorin Herbarium
6	Abubakar, Kankara [158]	Traditional medicinal plants used for treating emerging and re-emerging viral diseases in northern Nigeria	NW/NC	Oral interviews using structured questionnaires	131	65	Herbal medicine practitioners	Umaru Musa Yaradua University Katsina Herbarium and Kebbi State University of Science and Technology Herbarium, Aliero, Nigeria
7	Adebisi and Bello [42]	An ethnobotanical survey of herbal male contraceptives used in Southwest Nigeria	SW	Direct interviews	7	6	Traditional healers and medicinal plant sellers	Not provided
8	Adedeji, Kayode [40]	An ethnobotanical study of plant species used for medicine by the Eegun	SW	Interviews using semi-structured questionnaires	44	38	Medicinal plant vendors, health, forestry, and community development officers	Herbarium of the Department of Plant Science and Biotechnology, Ekiti State University, Ado-Ekiti
9	Adekunle, Oluwalana [159]	An exploratory survey of forest plants in traditional treatment of guinea worm infections (GWI) (*Dracunculus medinensis* Linn): experiences from Nigeria and Ethiopia	SW	Interviews using pretested and structured open-ended questionnaires	92	68	Elders, herbalists and individuals with Indigenous knowledge	Not provided
10	Afolayan and Sowemimo [14]	Ethnobotanical study of plants used for treating intestinal worms in Ibadan, Nigeria	SW	Interviews using structured questionnaires	45	31	Herb sellers,	Not provided
11	Afolayan, Sulaiman [160]	Ethnobotanical survey of plants used in cancer therapy in Iwo and Ibadan, South-western Nigeria	SW	A structured questionnaire	92	51	Pharmacognosists, herbalists, traditional medical practitioners, and herb vendors	Not provided
12	Ajibesin, Bala [85]	Ethnobotanical survey of Akwa Ibom state of Nigeria	SS	Personal interviews using an ethnobotanical survey list	114	54	Traditional medical practitioners, community elders and patients	Herbarium of the Department of Pharmacognosy and Natural Medicine, Faculty of Pharmacy, University of Uyo
13	Ajibesin, Bala [41]	Ethnomedicinal survey of plants used by the indigenes of Rivers State of Nigeria	SS	Guided field interviews using Semi-structured questionnaires	188	82	Traditional medical practitioners (TMPs) and community elders	Herbarium of the Department of Pharmacognosy and Natural Medicine, Faculty of Pharmacy, University of Uyo, Uyo State Nigeria
14	Ali, Ior [114]	Ethnobotanical survey of plants used as biopesticides by Indigenous people of Plateau State, Nigeria	NC	On-the-spot administration of semi-structured questionnaires	45	30	Experts within different ethnic groups who possess extensive knowledge	Department of Plant Science and Biotechnology Herbarium, University of Jos
15	Ambali, Ajaiyeoba [37]	Ethnobotanical survey of plants used for cancer treatment in Akinyele local government of Ibadan, Nigeria and preliminary cytotoxic activity of selected plants	SW	Focus-group discussion and administration using semi-structured questionnaires	26	18	Traditional medicine practitioners	Forest Herbarium Ibadan
16	Ampitan [34]	Ethnobotanical survey of medicinal plants in Biu local government area of Borno State, Nigeria	NE	Pre-designed structured questionnaires	27	24	Traditional medicine practitioners, and medicinal plant sellers	Not provided
17	Amusa, Jimoh [161]	Ethnobotany and conservation of plant resources of Kainji Lake National Park, Nigeria	NC	Focus group discussions and household surveys using structured and semi-structured questionnaires	37	18	Household head (male or female)	Forest Herbarium Ibadan
18	Ashidi, Houghton [130]	Ethnobotanical survey and cytotoxicity testing of plants of Southwestern Nigeria used to treat cancer, with isolation of cytotoxic constituents from *Cajanus cajan* Millsp. leaves	SW	Interviews using structured questionnaires	45	30	Traditional healers	Forest Herbarium Ibadan
19	Ashidi, Awokoya [131]	Ethnobotanical survey and proposed recipes of potential wound-healing plants in parts of Southwest Nigeria	SW	Interviews using structured questionnaires	71	43	Traditional healer	Forest Herbarium, Ibadan and Elikaf Herbarium, Olabisi Onabanjo University
20	Atawodi, Olowoniyi [162]	Ethnomedical survey of Adavi and Ajaokuta local government areas of Ebiraland, Kogi State, Nigeria	NC	Interview and detailed discussions using structured questionnaires	16	13	Herbalists	Herbarium Section of the Department of Biological Sciences, Ahmadu Bello University, Zaria, Nigeria
21	Aworinde and Erinoso [150]	Ethnobotanical investigation of Indigenous plants used in the management of some infant illnesses in Ibadan, Southwestern Nigeria	SW	Personal interviews using periodic open-ended questionnaire	48	31	Women herb sellers	Forest Herbarium Ibadan
22	Aworinde, Morenikeji [97]	The doctrine of signatures in herbal prescriptions in Ikale and Ilaje communities of Ondo State, Southwestern Nigeria	SW	Personal interviews using structured questionnaires	60	37	Community dwellers	Department of Biological science Herbarium, Olusegun Agagu University of Science and Technology, Ondo-State
23	Ayeni and Kayode [163]	Ethnobotanical survey of plants’ stem barks used in Kaduna State of Nigeria	NW	Group interview	54	28	Officials of the local Governments’ community health units, clerics and teachers	Not provided
24	Borokini and Omotayo [164]	Phytochemical and ethnobotanical study of some selected medicinal plants from Nigeria	SW	Semi-structured interviews and discussions	23	13	Not specified	Herbarium of the Department of Plant Science, University of Ado-Ekiti
25	Dike, Obembe [102]	Ethnobotanical survey for potential antimalarial plants in Southwestern Nigeria	SW	Oral interviews using semi-structured questionnaires	22	18	Indigenous people	Forest Herbarium Ibadan
26	Enebeli-Ekwutoziam, Aruah [143]	Ethnomedicinal survey of plants used in the treatment of skin-related ailments in the Northern Delta State of Nigeria	SS	Personal interviews using semi-structured questionnaires	51	29	Herbalists, herbal drug sellers, and the elderly	Bioresources Development Centre Herbarium (BDU), Ubulu-Uku, Delta State
27	Erinoso and Aworinde [82]	Ethnobotanical survey of some medicinal plants used in traditional health care in Abeokuta areas of Ogun State, Nigeria	SW	Oral interviews using semi-structured questionnaires	58	34	Traditional medical practitioners, herbalists and herb sellers	Forest Herbarium Ibadan
28	Etuk, Ugwah [62]	Ethnobotanical survey and preliminary evaluation of medicinal plants with antidiarrhoea properties in Sokoto state, Nigeria	NW	Oral interviews using structured questionnaires	19	14	Herbalists	Herbarium of the Department of Botany, Usmanu Danfodiyo University, Sokoto
29	Evbuomwan, Adeyemi [101]	Indigenous medicinal plants used in folk medicine for malaria treatment in Kwara State, Nigeria: An ethnobotanical study	NC	oral face-to-face interviews using in-depth, standardised semi-structured questionnaires	62	36	Traditional medicine practitioners	Forest Herbarium Ibadan, National Institute of Pharmaceutical Research and Development (NIPRD), Abuja and University of Benin Herbarium (UBH), Benin City, Nigeria
30	Fred-Jaiyesimi and Ajibesin [165]	Ethnobotanical survey of toxic plants and plant parts in Ogun State, Nigeria	SW	Interviews using semi-structured questionnaires	93	43	Traditional healers, elders, herbalists, and plant experts	Department of Pharmacognosy Herbarium, Faculty of Pharmacy, Olabisi Onabanjo University, Sagamu Campus
31	Fred-Jaiyesimi, Ajibesin [166]	Ethnobotanical studies of folklore phytocosmetics of Southwest Nigeria	SW	Interview using semi-structured questionnaires	80	39	Elders, herb vendors, and other individuals who possess expertise regarding the utilisation of plants as cosmetics	Not provided
32	Gbolade [111]	Inventory of antidiabetic plants in selected districts of Lagos State, Nigeria	SW	Oral interviews using semi-structured questionnaires	49	33	Traditional medical practitioners, herbalists, and herb sellers	Department of Botany, Obafemi Awolowo University Herbarium (IFE) and Forest Herbarium, Ibadan (FHI)
33	Hassan, Edo [90]	An inventory of medicinal plants used as sedative, analgesic and blood tonic in Abeokuta, Ogun State, Nigeria	SW	Oral interviews using structured questionnaires	28	21	The inhabitants of the study location	Not provided
34	Ibrahim, Muazzam [104]	Ethno-medicinal plants and methods used by Gwandara tribe of Sabo Wuse in Niger State, Nigeria, to treat mental illness	NC	Oral interviews using questionnaires	18	12	Traditional healers	Herbarium of National Institute for Pharmaceutical Research and Development, Abuja
35	Idowu, Soniran [167]	Ethnobotanical survey of antimalarial plants used in Ogun State, Southwest Nigeria	SW	Interviews using semi-structured questionnaires	38	24	Farmers, mothers, herb vendors, community leaders, and elders	Not provided
36	Idu, Erhabor [168]	Documentation on Medicinal Plants Sold in Markets in Abeokuta, Nigeria	SW	oral interview using structured questionnaires	60	31	Men, women and young girls	Department of Plant Biology and Biotechnology Herbarium, University of Benin
37	Igoli, Igwue [169]	Traditional medicinal practices among the Igede people of Nigeria	NC	Oral interviews	39	23	The Igede people, particularly the elderly, local healers, and practitioners of herbal medicine	Herbarium of the University of Agriculture, Makurdi
38	Ishola, Oreagba [146]	Ethnopharmacological survey of herbal treatment of malaria in Lagos, Southwest Nigeria	SW	Focus group discussions and interviews using semi-structured questionnaires	41	27	Herb dealers, traditional herbal medicine practitioners, nursing mothers, undergraduate students, and elderly individuals who specialise in herb use	Not provided
39	Iyamah and Idu [59]	Ethnomedicinal survey of plants used in the treatment of malaria in Southern Nigeria	SE, SS, SW	Interviews using semi-structured questionnaires	156	60	Traditional herb sellers and herbal practitioners	Department of Plant Biology and Biotechnology Herbarium, University of Benin, Benin City, Edo State, Nigeria
40	Kadiri, Ojewumi [132]	Indigenous uses and phytochemical contents of plants used in the treatment of menstrual disorders and after-childbirth problems in Abeokuta South local government area of Ogun State, Nigeria	SW	Interview using questionnaires	56	37	Traditional herbal practitioners, herb sellers and Indigenous people	Not provided
41	Kankara, Ibrahim [61]	Ethnobotanical survey of medicinal plants used for traditional maternal healthcare in Katsina State, Nigeria	NW	Interviews using semi-structured questionnaires	111	50	Herbalists, traditional medical practitioners, traditional midwives, housewives, farmers, and others	Umaru Musa Yar’adua University Herbarium, Katsina, Nigeria
42	Kayode [170]	Conservation perception of endangered tree species by rural dwellers of Ekiti State, Nigeria	SW	Individual and group interviews using a semi-structured matrix	48	15	Community dwellers	Department of Plant Science and Forestry Herbarium, University of Ado-Ekiti, Ado-Ekiti, Nigeria
43	Lawal, Olufade [105]	Ethnobotanical survey of plants used for treating cough associated with respiratory conditions in Ede South local government area of Osun State, Nigeria	SW	Semi-structured interview	87	39	Herb-sellers, traditional medical practitioners (herbalists), farmers, and hunters	Forest Herbarium, Ibadan (FHI)
44	Lawal, Rafiu [15]	Ethnobotanical survey of local flora used for medicinal purposes among Indigenous people in five areas in Lagos	SW	Field survey using semi-structured questionnaires	183	61	Herbal vendors, traditionalists, farmers, hunters	Forest Herbarium, Ibadan (FHI)
45	Lor, Otimenyin [171]	Ethnobotanical survey of plants used in the management of mental illnesses in some selected local government areas of Plateau State, Nigeria	NC	Oral interviews using semi-structured questionnaires	42	31	Traditional medicine practitioners, herb sellers and herbalists	Herbarium section of the Federal College of Forestry, Jos, and Department of Biological Sciences Herbarium, Ahmadu Bello University, Zaria (ABU), Nigeria
46	Mahmoud, Labaran [63]	Ethnobotany of medicinal plants with antimalarial potential in Northern Nigeria	NE	Interview using semi-structured questionnaires	21	18	Traditional medical practitioners, birth attendants, herbalists and rural dwellers	Herbarium of the Agricultural Department, School of Science, Federal Polytechnic, Mubi, Adamawa state, Nigeria
47	Malami, Jagaba [172]	Integrating medicinal plants into the traditional medicine system for cancer treatment in Sokoto State, Nigeria	NW	Field survey using modified semi-structured questionnaires	67	31	Herb sellers elders, and herbalists	Herbarium of the Department of Pharmacognosy and Ethnopharmacy, Faculty of Pharmaceutical Sciences, Usmanu Danfodiyo University, Sokoto
48	Mann, Amupitan [173]	An ethnobotanical survey of Indigenous flora for treating tuberculosis and other respiratory diseases in Niger State, Nigeria	NC	Interviews	95	48	Traditional medical practitioners and herbal traders	Department of Biological Sciences, Ahmadu Bello University Zaria, Nigeria and the National Institute for Pharmaceutical Research and Development, Idu-Abuja
49	Ngulde, Sandabe [64]	Ethnobotanical survey of anticancer plants in Askira/Uba local government area of Borno State, Nigeria	NE	Oral interviews using the questionnaire	65	41	Traditional medicine practitioners	Department of Biological Science, University of Maiduguri, Nigeria
50	Nurudeen, Salimon [84]	Ethnopharmacological survey of plants used for the treatment of female sexual dysfunction and infertility in Ilorin, Nigeria	NC	Oral or virtual interviews using semi-structured questionnaires	47	28	Farmers, herb vendors, midwives, herbalists/traditional medicine practitioners, and elderly individuals	University of Ilorin Herbarium, Ilorin, Nigeria
51	Nwosu [174]	Ethnobotanical studies on some pteridophytes of Southern Nigeria	SE	Face-to-face interviews during a field survey	36	22	Herbalists	University of Nigeria, Nsukka, Department of Botany Herbarium, UNN
52	Odebunmi, Adetunji [96]	Ethnobotanical survey of medicinal plants used in the treatment of COVID-19 and related respiratory infections in Ogbomosho South and North local government areas, Oyo State, Nigeria	SW	Interviews using semi-structured questionnaires	26	17	Herb sellers, traditional health practitioners, farmers, and individuals with Indigenous knowledge	Herbarium of the Obafemi Awolowo University, Ile-Ife, Osun State, Nigeria (IFE)
53	Odewo, Ajani [83]	Ethno-botanical survey of indigenous medicinal plants in agroforestry farm of Forest Research Institute of Nigeria, Ibadan, Oyo State, Nigeria	SW	Face-to-face interview	105	49	Agroforestry farmers	Forest Herbarium Ibadan (FHI)
54	Odoh, Uzor [129]	Medicinal plants used by the people of Nsukka local government area, Southeastern Nigeria for the treatment of malaria: An ethnobotanical survey	SE	Oral interviews using semi-structured questionnaires	50	30	Various communities in the local government area	Department of Pharmacognosy Herbarium, University of Nigeria, Nsukka
55	Ofeimun and Temitope [133]	Herbal treatment of benign prostatic hyperplasia: Findings from an ethnobotanical survey of Akinyele local government area, Oyo State Nigeria	SW	Semi-structured questionnaires with open-ended interviews	25	23	Herbal practitioners and herb sellers	Forest Herbarium Ibadan
56	Offiah, Makama [115]	Ethnobotanical survey of medicinal plants used in the treatment of animal diarrhoea in Plateau State, Nigeria	NC	Interviews using a well-structured, open-ended questionnaire with guided dialogue approaches	57	25	Pastoralists and native livestock farmers	Herbarium of the Department of Biological Sciences, Ahmadu Bello University (ABU), Zaria
57	Ogbole and Ajaiyeoba [89]	Traditional management of tuberculosis in Ogun State of Nigeria: The practice and ethnobotanical survey	SW	Oral interviews using semi-structured questionnaires	36	20	Traditional medical practitioners, herb vendors, and herbalists	Forest Herbarium Ibadan
58	Ogunkunle and Ladejobi [175]	Ethnobotanical and phytochemical studies on some species of *Senna* in Nigeria	SW	Face–to–face interviews and exhaustive solvent extraction	5	1	Practitioners of herbal medicine and vendors of herbs	Forest Herbarium Ibadan
59	Ohemu, Okwori [98]	Ethnobotanical survey of medicinal plants used to treat male infertility in Jos North local government area of Plateau State, Nigeria	NC	Face-to-face interviews using semi-structured questionnaires	21	18	Traditional medicines practitioners, herb sellers, and herbalists	Not provided
60	Ojetunde, Tongshuwar [16]	An ethnobotanical survey of plants used in rheumatoid arthritis treatment: A case study of Gusau in Nigeria	NW	One-on-one interviews with an electronic questionnaire	12	10	Male and female residents of Gusau, Zamfara state	Not provided
61	Oladeji and Agbelusi [58]	Capturing Indigenous Knowledge on medicinal plants use: a case study of selected communities in old Oyo National Park, Nigeria	SW	Focus group discussions, interviews, and field observations	78	39	Herbal vendors, practitioners of traditional medicine, and experts in herbal remedies	Not provided
62	Oladunmoye and Kehinde [57]	Ethnobotanical survey of medicinal plants used in treating viral infections among Yoruba tribe of Southwestern Nigeria	SW	Oral discussions using questionnaires	208	54	Herbalists, herb vendors, and villagers	Not provided
63	Olanipekun [17]	Ethnobotanical relevance and conservation of medicinal plants used to treat human diseases in Ifedore, Ondo-State, Nigeria	SW	Semi-structured interviews and field observations	98	47	Community members and elderly people	Herbarium unit of the Department of Plant Science and Biotechnology, Ekiti State University, Ado-Ekiti, Nigeria
64	Olatokunbo, Olanipekun [60]	Ethnobotanical survey and conservation of the Indigenous plants used for traditional orthopaedic care practices in Bayelsa Central Senatorial District, Nigeria	SS	An open-ended, semi-structured questionnaire and direct field observation	39	30	Traditional bone healers and members of the communities who had maintained domicile for less than 15 years and with knowledge of plants used for fracture treatment and bone-related disorders	Department of Plant Science and Biotechnology Herbarium, Bayelsa State University
65	Olorunnisola, Adetutu [176]	Ethnobotanical survey of medicinal plants used in the treatment of malarial in Ogbomoso, Southwest Nigeria	SW	Interviews and general conversations using structured questionnaires	40	32	Herbalist, rural dwellers and the traditional healers	Ladoke Akintola University of Technology Herbarium, Ogbomoso, Oyo State (LHO)
66	Omotayo and Borokini [177]	Comparative phytochemical and ethnomedicinal survey of selected medicinal plants in Nigeria	SW	Semi-structured interviews and discussions	22	15	Not specified	Department of Plant Science Herbarium, University of Ado-Ekiti, Ekiti State (UHAE)
67	Oyeyemi, Akinseye [178]	Ethnobotanical survey of the plants used for the management of malaria in Ondo State, Nigeria	SW	Interviews using standardised semi-structured questionnaires	97	52	Herbal vendors, traditional healers, and other members of the local community	Wesley University of Science and Technology, Ondo, Nigeria
68	Rafiu and Sonibare [179]	Ethnobotanical survey of tree species used for wound healing in Ibadan, Southwest Nigeria	SW	Oral interviews using semi-structured questionnaires	71	30	Herb sellers, traditional medical practitioners, and a few elders in the study location	Not provided
69	Salihu, Olukunle [180]	Ethnomedicinal plant species commonly used to manage arthritis in North-West Nigeria	NW	Interviews using semi-structured questionnaires	30	18	Traditional medicine practitioners	Ethno-botanical survey Herbarium, Nigeria Natural Medicine Development Agency, Lagos State, Nigeria
70	Segun, Ogbole [181]	Medicinal plants used in the management of cancer among the Ijebus of southwestern Nigeria	SW	Interviews using semi-structured questionnaires	90	47	Herbalists, herb sellers and traditional medical practitioners	Department of Pharmacognosy Herbarium, University of Ibadan (DPHUI) and the Forest Herbarium Ibadan (FHI)
71	Shinkafi, Bello [103]	An ethnobotanical survey of antidiabetic plants used by Hausa–Fulani tribes in Sokoto, Northwest Nigeria	NW	Oral interviews using semi-structured questionnaires	54	33	Herbal medicinal practitioners and traditional healers	Usmanu Danfodiyo University Herbarium, Sokoto
72	Soladoye, Sonibare [182]	Indigenous angiosperm biodiversity of Olabisi Onabanjo University permanent site	SW	Site visitation	138	55	Undisturbed and disturbed vegetation of the Olabisi Onabanjo University’s permanent site	Forest Herbarium, Ibadan, University of Ibadan Herbarium and Elikaf Herbarium, Olabisi Onabanjo University
73	Sonibare and Abegunde [183]	Ethnobotanical study of medicinal plants used by the Laniba village people in Southwestern Nigeria	SW	Unstructured interviews	21	15	Imams, herbalists, traditional medicinal practitioners, hunters, and experienced village elders (both male and female)	Forest Herbarium Ibadan
74	Sonibare and Ayoola [184]	Medicinal plants used in the treatment of neurodegenerative disorders in some parts of Southwest Nigeria	SW	Focused group discussion and oral interviews using semi-structured questionnaires	22	19	Traditional medicine practitioners (TMPs), herbalists, herb sellers and the elderly people	Forest Herbarium Ibadan
75	Sonibare and Gbile [185]	Ethnobotanical survey of anti-asthmatic plants in Southwestern Nigeria	SW	Oral interviews	46	13	Herb sellers and traditional medical practitioners	Forest Herbarium Ibadan
76	Sonibare, Moody [186]	Use of medicinal plants for the treatment of measles in Nigeria	SW	Unstructured interviews	23	18	Herbalists, herb vendors, and elderly individuals possessing specialised knowledge of plants used in treating measles in children	Forest Herbarium Ibadan
77	Sonibare, Okorie [187]	Ethno-medicines for mosquito-transmitted diseases from Southwestern Nigeria	SW	Oral interviews using semi-structured questionnaires	37	25	Traditional medical practitioners, herbalists, herb sellers, and elders	Department of Pharmacognosy Herbarium, University of Ibadan (DPHUI)
78	Sulaiman, Arzai [10]	Ethnobotanical survey: A comprehensive review of medicinal plants used to treat gastrointestinal diseases in Kano State, Nigeria	NW	Interviews through a structured questionnaire	33	30	Traditional medical practitioners	Herbarium of Department of plant science, Bayero University, Kano
79	Yaradua and El-Ghani [91]	Ethnobotanical survey of edible plants sold in Katsina metropolis markets	NW	Oral interviews using structured questionnaires	54	33	Food plant vendors and experts in the field who are Indigenous to each site’s region	Not provided

Key: NE–Northeast, NW–Northwest, NC–North Central, SE–Southeast, SS–South-South, SW–Southwest

The 79 eligible ethnobotanical studies included in this review clearly stated their study objectives, had well-defined qualitative findings deemed significant, and collected data relevant to the research topic (Supplementary material 1). Thirty-four studies (43.03%) included ethical considerations in their design, while 70 studies (88.61%) revealed the relationship between the researcher and subjects (participants). Data analysis was rigorous in 48 studies (60.76%), inadequate in 25 studies (31.65%), and inconclusive in six studies (7.60%). Regarding the recruiting strategy, 75 studies (94.93%) applied a suitable approach aligned with their objectives, while four studies (8.33%) had an ambiguous assessment of this issue.

#### Overview of the demographic data from the 79 eligible ethnobotanical studies

The ethnobotanical studies collected demographic information from participants, offering insights into their age, gender, educational background, occupation, and years of experience **(**Table 2**)**. The study revealed that most participants, aged between 41 and 60 years, an indication of the prevalence of older, experienced participants in these communities. Over 85% of traditional healers in Nigeria identified as male, indicating a male-dominated occupation [11, 34]. However, there was a notable presence of women in herb trading [35].

Most participants had either no formal education or only a primary-level education, which underscores the reliance on oral transmission for sharing ethnobotanical knowledge. As a result of this limited formal education, there has been inadequate documentation and a loss indigenous knowledge associated the plants in local communities [36]. The ratio of individuals without formal education compared to those who completed secondary education was 18.7–52.3%, while those with a primary education status were 26% of the participants [34, 35, 37, 38].

The participants consisted mainly of traditional healers (55%), herb vendors (20%), traditional birth attendants (10%), and healthcare professionals (5%) [14, 35]. It is crucial to understand the demographic data collected from these studies to gain further insight into the experiences and perspectives of traditional healers and herb vendors in Nigeria.

#### Assessment of the methodological approaches identified in the 79 eligible ethnobotanical studies

The methods used in the 79 ethnobotanical studies conducted in Nigeria can be categorised into several key components, highlighting a comprehensive approach to the ethnobotanical field surveys. These components include:

*Selection of study areas:* Researchers intentionally selected ethnobotanically diverse regions, focusing on both rural and urban areas (Table 2). Abo, Fred-Jaiyesimi [39] surveyed southwestern states of Nigeria including Ogun, Oyo, Osun, and Lagos. On the other hand, Abubakar, Ukwuani-Kwaja [38] focused on identifying plants used for cancer treatment in the rural areas of Kebbi State, northern Nigeria, Adedeji, Kayode [40] selected the Egun indigenous tribal group in Lagos State with interest on their prominent plants and the preparation techniques. Ajibesin, Bala [41] targeted the indigenous people of River State to record how they use medicinal plants to treat different health conditions.

*Participants sampling:* Various sampling techniques, including random and purposive sampling, were used to gather data from a diverse group of participants (Table 2). Some of these include traditional healers, herbalists, community elders, rural residents, and herb vendors, thereby ensuring a comprehensive understanding of Indigenous practices. For instance, Abdallah, Mustafa [11] surveyed 97 traditional healers in Yobe State to tap into their extensive knowledge. In SW Nigeria, Adebisi and Bello [42] engaged with herbal contraceptive practitioners to explore their specialised expertise.

*Data collection tools:* Different data collection tools were meticulously chosen to ensure the quality and scope of the information gathered from the studies. The qualitative research methods chosen were thoughtfully aligned with the context and aims of the studies. Structured interviews provided a systematic approach to data collection, ensuring consistency and facilitating effective comparison of responses [43]. Semi-structured interviews provided the needed flexibility to delve deeper into the perspectives of the participants while aligning with the predefined themes [44]. Unstructured interviews were utilised to gather authentic narratives and insights that might not have been foreseen before the interviews [45]. Generally, focus groups offered valuable qualitative insights that enriched the quantitative data, leading to an in-depth viewpoints of the participants [43]. Field observations were explored to collect direct information regarding behaviours and practices within the community and offer important contextual insights into the underlying issues being investigated [43]. This multifaceted approach enhanced the validity of the findings and ensured a well-rounded view of the complexities involved in the ethnobotanical research.

*Use of local language*: Ethnobotanical surveys were used to collate local uses of plants, identify practices associated with traditional knowledge, and understand the cultural significance of botanical resources [46]. These surveys provided a valuable platform for engaging with the community members, fostering meaningful dialogue, and enabling them to share their insights and experiences. To promote effective communication and build trust within the community, most participants were interviewed in their native languages [35]. Conducting interviews in the native languages of the participants enabled them to express themselves freely, minimized the chances of misunderstandings, and improved the reliability and accuracy of the data collected. This is consistent with the assertion by Cámara-Leret and Bascompte [47] that knowledge about medicinal plant usage is specific to each language, underscoring the intimate link between language and traditional knowledge. Moreover, applying a variety of data collection tools facilitated a thorough and detailed comprehension of the research topic, thereby capturing the quantitative and qualitative dimensions of the experiences and perspectives of the participants.

*Plant identification:* Given the importance of ensuring the verification of plant identities [48], researchers have applied rigorous protocols for plant identification to ensure the scientific accuracy of their investigations. This often entails the collection of voucher specimens and using herbaria for taxonomic confirmation, and cross-referencing with reliable botanical databases, including the Kew Plant of the World (https://powo.science.kew.org/), for the verification of the scientific names. Based on the eligible study, the Forest Herbarium in Ibadan, Bayero University in Kano, and the University of Lagos Herbarium, were frequently mentioned herbaria for the storage of voucher specimens (Table 2). Voucher specimens play a crucial role in research by providing a permanent record, enabling verification, and ensuring reproducibility, which is essential for biodiversity assessments, accurate species identification, and effective conservation efforts [49]. This practice validates the findings through cross-referencing with physical specimens and enhances the credibility of the conducted research by ensuring transparency and traceability.

#### Approaches for data analysis in the 79 eligible ethnobotanical studies

The method applied in ethnobotanical field surveys integrates both qualitative and quantitative techniques to understand the significance, usage trends, and conservation requirements of various plants. This assists researchers in identifying crucial plants that require conservation efforts, conducting cross-cultural and regional comparisons of plant utilisation, and advocating for the integration of ethnobotanical knowledge into conservation and healthcare systems [50]. One of the common presentation entails descriptive statistics such as frequency distributions, percentages, bar charts, and trend analyses, which assist in organising data and identifying patterns in plant uses [51].

Ethnobotanical indices such as Relative Frequency of Citation (RFC), Use Value (UV), and Informant Consensus Factor (ICF) have been applied to establish the popularity of plants and degree of agreements among the participants [50, 52]. These indices facilitate standardised data collection and enhance the reliability of research findings [52, 53]. However, these indices often oversimplify the cultural and pharmacological significance of plants, leading to potentially misleading conclusions [53].

Thematic analysis, content analysis, case studies, and ethnographic methodologies in qualitative data analysis provide in-depth cultural insights into conventional knowledge systems [54]. Thematic analysis classify data, whereas content analysis investigates oral histories and traditional knowledge [55].

Analysing ethnobotanical data has multiple implications, such as aiding in the identification of crucial plant species for conservation, facilitating comparisons of plant usage among different cultures, and uncovering knowledge gaps in underrepresented regions [53]. It promotes policy recommendations that integrate ethnobotanical knowledge into conservation and healthcare frameworks, protects indigenous knowledge, and bridges traditional and modern medicine for comprehensive healthcare approaches [56].

#### Distribution of studies across different states/regions and insights into ethnobotanical implications

In this review, plants and their ethnobotanical uses were recorded in 32 out of 36 states in Nigeria. Oyo (14.03%), Ogun (13.45%), and Lagos (6.43%) had the highest representation, while Kogi, Yobe, Adamawa, Zamfara, and Benue had the lowest proportion (0.58%) of studies (Fig. 2C). These variations in documentation efforts highlight the diverse levels of research and awareness in ethnobotany across the different states. Conducting further studies in underrepresented states is crucial for establishing valuable insights into the uses and sustainable practices associated with local plants. Expanding the investigation to cover less-represented states such as Kogi, Benue, Jigawa, Zamfara, and Yobe is crucial for expanding our understanding of the floras and their traditional uses in these underrepresented regions. This understanding is crucial for developing conservation policies that consider the unique ethnobotanical perspectives of local communities. Importantly, it is vital to achieve a harmonious balance between conservation initiatives and sustainable practices to protect rich biodiversity in Nigeria for the well-being of the current and future generations.

In SW Nigeria, the ethnobotanical studies have mainly focused on malaria, diabetes, cancer, wound healing, and cosmetic applications. Lawal, Rafiu [15] conducted ethnobotanical surveys in five communities (Alimosho, Badagry, Eti-Osa, Epe, and Ikorodu) in Lagos State, recording 183 plant species from 61 families. Furthermore, Oladunmoye and Kehinde [57] surveyed local communities in Ekiti, Ondo, Osun, and Oyo states and generated 208 plant species used for viral infections. Oladeji and Agbelusi [58] identified 78 plants used by the communities around the old Oyo National Park. Furthermore, existing evidence provide valuable information on how medicinal plants are used in the area, which adds to the overall body of ethnobotanical knowledge in SW Nigeria [14, 39, 42]. Ethnobotanical surveys in SE and SS regions of Nigeria revealed the use of plants for treating malaria, managing fractures, and addressing skin-related ailments. An in-depth study by Iyamah and Idu [59] documented 156 plant species used for malaria treatment in Southern Nigeria, covering SE, SS, and SW regions. This extensive inventory highlights the rich diversity of plants in these areas and their importance in traditional medicine. In Bayelsa Central Senatorial District of Nigeria [60], 39 plants from 30 families were utilised for traditional orthopaedic care.

In NW region, researchers have extensively documented the use of plants for maternal health, arthritis, and paediatric ailments. Kankara, Ibrahim [61] documented 111 plants used for maternal healthcare in Katsina State, highlighting the contributions of traditional midwives and herbalists. Additionally, Abubakar, Yusuf [35] and Etuk, Ugwah [62] conducted studies in Kano and Sokoto states, which revealed that NW region has rich indigenous knowledge on the use of medicinal plants. Research findings from NE region identified plants for managing cancer, gastrointestinal diseases, and malaria. Mahmoud, Labaran [63] recorded 21 plants locally used for managing malaria in Mubi town, Adamawa State. Furthermore, Ngulde, Sandabe [64] conducted a comprehensive study that identified 65 plants recognised for their potential anticancer effects in Askira/Uba local government area of Borno State, thereby highlighting the rich ethnobotanical knowledge prevalent in NE Nigeria.

#### Cross-cultural analysis on the utilisation of botanicals in Nigeria

Nigeria is home to over 371 ethnic groups, broadly classified as majority and minority populations [65]. The Hausa-Fulani, Yoruba, and Igbo collectively constitute 57.8% of the population [66], yet the ethnolinguistic diversity of Nigeria spans six geopolitical zones consisting of NC, NE, NW, SE, SS, and SW (Fig. 2A). These regions are shaped by unique cultural histories, ecological zones, and plant uses [67, 68].

Northwest region is dominated by Hausa-Fulani communities rooted in Islamic traditions and emirate governance structures [69, 70], while NE hosts the Kanuri and other ethnic minorities such as the Tangale and Bura, reflecting a cultural heritage linked to the Kanem-Bornu Empire [70, 71]. North-central zone includes a highly diverse population, such as the Tiv, Nupe, Gwari, and Idoma, known for vibrant dance, music, and festivals [70, 72, 73]. South-South region is associated with the Ijaw, Efik, Urhobo, Itsekiri, and Ibibio, whose traditions feature rich maritime cultures and ceremonial regattas [70, 74]. In SW, Yoruba communities inhabit Lagos, Oyo, Ogun, Osun, Ekiti, and Ondo states, marked by complex political institutions and a strong intellectual and spiritual heritage [70, 73, 75]. They are recognised for their distinctive traditions and significant historical contributions [76]. Southeast is predominantly Igbo, noted for entrepreneurial traditions, decentralised governance, and strong ritual and ceremonial plant use [73]. They are known for their inventive attitude, traditional governance, and substantial commerce activity [66, 76]. These cultural distinctions are often interwoven with regional patterns of plant usage and ecological variation.

**Fig. 3:**
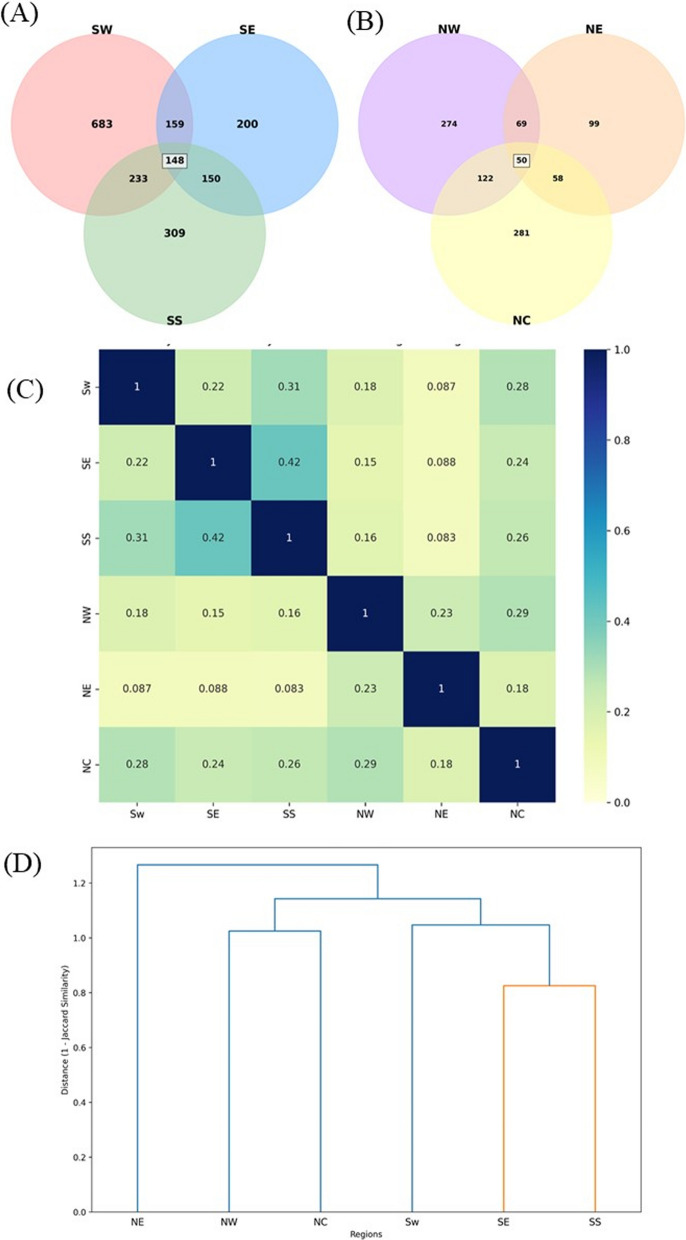
Cross-regional analysis of ethnobotanical uses of plants in Nigeria from 1964 to 2024. **A** Southern region consisting of Southeast (SE), South-South (SS) and Southwest (SW); **B** Northern region consisting of North-Central (NC), Northeast (NE) and Northwest (NW); **C** Jaccard similarity indices on plant utilisation across the six regions in Nigeria; **D** Hierarchical clustering on plant utilisation across the six (6) regions in Nigeria. For detailed list of the plants based on their cross-cultural occurrence, see Supplementary material 2)

Ecologically, Nigeria spans a north–south gradient from humid tropical forests to arid savannahs (Fig. 2B), influencing the floristic composition and cultural knowledge systems. The ethnobotanical inventory analysed across the six regions reveals 963 distinct plants, with the SW region exhibiting the highest diversity (70.98% of plants), followed by SS (32.19%), NC (29.18%), NW (28.45%), SE (20.77%), and NE (10.28%) (Supplementary material 2). The Southern zones (SW, SE, SS) form a coherent forest ethnobotanical bloc (Fig. 3A), characterised by frequent use of taxa, such as *Alstonia boonei*, *Newbouldia laevis*, and *Garcinia kola*. The intersection between SW and SS regions is robust, suggesting a historical interconnectedness and cultural exchange between these areas. In contrast, the northern zones (NE, NW) depend heavily on drought-resistant plants such as *Adansonia digitata* and *Balanites aegyptiaca* [77]. The utilisation of plants within these regions is significantly influenced by the specific cultural beliefs, traditions, and practices of the local communities. The overlap observed between NE and NW regions indicates a strong exchange of ethnobotanical knowledge within the savannah zone, further illustrating the dynamics of knowledge sharing in these environments [78]. North-Central region displays a mixed composition, highlighting its role as an ecological and cultural transition zone.

Each region retains unique plants as highlighted for SW (308 plants, 45.2%), NW (86 plants, 31.5%), SS (64 plants, 20.8%), SE (36 plants, 18.1%), NC (39 plants, 13.9%), and NE (13 plants, 13.3%). The prominence of ferns in SE, notably *Adiantum* spp., suggests a ritual and therapeutic tradition within Igbo culture [79]. Conversely, the unique taxa in NW and NE reflect adaptation to arid ecosystems and transmission of Islamic phytomedical knowledge. The significance of cultural selection processes operating within established ecological boundaries is underscored by such specific regional usage patterns [80].

Despite this diversity, 19 plants (~ 8.3%) were recorded across all six regions, including *Abelmoschus esculentus*, *Carica papaya*, *Azadirachta indica*, and *Vernonia amygdalina* (Supplementary material 2). These form a shared ethnobotanical core, likely sustained by interregional trade, colonial agricultural dissemination, and post-independence integration [18, 79]. The Jaccard similarity heatmap and hierarchical clustering analyses underscore ecological and cultural clustering (Fig. 3C). The SE–SS pairing exhibits the highest similarity (0.42), followed by SW–SS (0.31), reflecting shared rainforest ecology and cultural overlap. NE and NW form a savannah-based cluster with modest internal similarity but minimal overlap with southern zones. The NC demonstrates intermediate similarity with all regions (SW = 0.28; NW = 0.29), confirming its bridging role. The dendrogram clustering reinforces these findings (Fig. 3D), delineating two main ethnobotanical zones: a southern forest belt (SW, SE, SS) and a northern savannah zone (NW, NE), with NC positioned as a transitional cluster.

Ethnobotanical usage of plants is deeply tied to cultural practices and spiritual beliefs [81]. *Newbouldia laevis*, for example, is used for fertility and hypertension in SW [15, 82–84], wound healing in SE [58], and stomach ailments in NC [39, 61, 62]. In SS, it is known for treating infections, joint dislocations, and skin conditions [4, 41, 85]. Across the regions, it also serves symbolic roles in spiritual protection, boundary demarcation, and marriage rituals [21, 86, 87]. Kola nuts (*Cola nitida*, *C. acuminata*) are considered as valuable plant in Nigeria [88]. It is used for digestion, alertness, and social rituals [41, 89–91], central to important ceremonies among the Hausa, Yoruba, and Igbo where it is called ‘*goro*’, ‘*obi abata*/*gbanja*’ and ‘*oji*’, respectively [92]. Economically, they serve as a cash crop and symbol of hospitality, unity, and spiritual connectivity [93, 94]. Other culturally significant plants include:

*Garcinia kola* (bitter kola): is important in the rituals, ceremonies, and spiritual cleansing practices of the Yoruba and Igbo cultures, similar to kola nuts [95]. It is used for respiratory conditions [95, 96], immune support [97], and as an aphrodisiac [98], and it functions as an essential commodity in local and international markets [99].

*Carica papaya* (paw paw)*:* is a vital fruit in many Nigerian households which may be linked to its rich pool of vitamins and antioxidants that boosts immune function [100]. In traditional medicine, its latex is applied for spiritual cleansing rituals and to treat malaria, digestive disorders, and infections [17, 101, 102].

*Combretum altum:* also known as ‘*geza*’ in Hausa and ‘*ogan-ibule*’ in Yoruba. It is used in northern Nigeria to treat diarrhoea and abdominal pain in nursing women [91], diabetes [103], and mental disorders [104]. People in SW use *Combretum altum* to treat rheumatism and cough [82, 105].

*Ocimum gratissimum* and *Vernonia amygdalina:* both plants play significant roles in diets and medicines, indicating a convergence of culinary and medicinal ethnobotany in certain regions [106–108]. *Vernonia amygdalina* holds great cultural importance in SW and SE Nigeria [109] where it is used in purification rituals and traditional ceremonies for healing and cleansing purposes [82, 110]. It is also known for its antidiabetic, antimalarial, and immune-boosting properties [85, 111]. In SE region, the Igbo people use *Ocimum gratissimum* at traditional weddings to bless the couple [112]. The southwestern area recognises *Ocimum gratissimum* for its antidiabetic properties [39], while the northern region uses it to treat gastrointestinal disorders [10].

Although there was instance with similarities in uses of plants among different cultures, there are distinctions in their applications and associated rituals. Particularly, Yoruba and Igbo cultures place emphasis on spiritual elements rather than solely on physical element of medicinal uses. The aspect of spirituality/religious affiliation is important as it influences the uses of plants Among surveyed communities, 65% of participants practising traditional African religions often integrate plants into spiritual rituals. There were also instances where Christian and Islamic practitioners emphasise medicinal uses and religious healing, e.g., Quranic inscriptions with *Newbouldia laevis* in the northern part of Nigeria [113].

#### Inventory of plant species in Nigeria with ethnobotanical uses

The 79 eligible studies generated an inventory of 963 plant species across 144 families utilised in Nigeria for diverse ethnobotanical applications (Appendix 1). From this comprehensive inventory, we extracted the top-cited 103 plants referenced 11 times and above, detailing their associated plant part(s) used, their applications, and preparation methods across multiple states in Nigeria **(**Table 3**)**. The database highlights noteworthy biodiversity and vast indigenous knowledge in Nigeria. This review categorised ethnobotanical applications of plants into seven categories, namely medicinal (for humans, crops and livestock), cosmetics, spiritual, biopesticides, poison, timber, and fuelwood. Based on the eligible studies, the emphasis has been on prevalent diseases such as malaria, cancer, diabetes, gastrointestinal issues, and mental health in ethnobotanical surveys, which is crucial for addressing health concerns affecting a significant portion of the population. Some studies have explored the cultural significance and applications of plants in cosmetics which highlights the intersection of traditional knowledge and modern cosmetic practices. Additionally, the potential of locally sourced plants as biopesticides in agriculture has been explored in Plateau State [114]. Some of the recorded plants were used to manage livestock health [115], an indication of the versatility of locally sourced plants for improving human and agricultural (crop and animal) health. Overall, medicinal applications emerge as the most prominent category due to their widespread utilisation in addressing various health concerns.

**Table 3:** An overview of the ethnobotanical uses of 103 most prominent plant species (cited 11 times or more) in Nigeria from 1964–2024. The scientific names were verified using Plants of the World Online (POWO), the Global Biodiversity Information Facility (GBIF), and World Flora Online (WFO) (accessed 04/12/2024)

Scientific Name (Family)	Nigerian state	*Local name	Ethnobotanical uses/ailments	Plant part	Mode of preparation	Mode of administration	Reference(s) used to generate the number of mentions
*Abrus precatorius* L. (Fabaceae)	Oyo, Lagos, Osun, Ogun, Ondo	Idonzakara (H.), Omisinmisin (Yor.), Anya nnunu (Ig.)	Cough, Expectorant	Leaves	Decoction	Oral	[15, 58, 96, 97, 105, 182]
	Ogun, Lagos, Oyo, Osun, Sokoto		Diabetes	Leaves/Seeds	Decoction/Infusion/Maceration	Oral	[39, 103, 111]
	Ogun		Guinea worm infestation	Seeds	Unspecified	Unspecified	[159]
	Ogun		Tonic	Leaves	Unspecified	Unspecified	[90]
	Kaduna, Kano, Katsina, Jigawa		Arthritis	Root	Decoction	Unspecified	[180]
	Ondo		Malaria	Leaves	Decoction	Oral	[17]
	Delta, Akwa Ibom		Skin diseases	Leaves	Decoction/Pulverisation	Body bath/Topical	[85, 143]
	Ogun		Toxic plant	Seeds	Unspecified	Oral	[165]
	Kogi		Cough, Sore throat, Wounds, Calming the brain	Leaves	Powder/Maceration	Oral/Topical	[162]
	Southern Nigeria states		Malaria	Leaves	Decoction	Unspecified	[59, 167]
	Ogun		Tuberculosis, Jaundice, Yellow fever	Unspecified	Decoction	Oral	[82]
	Osun, Oyo		Cancer	Seeds	Unspecified	Unspecified	[160]
	Oyo		Neurodegenerative disorders	Leaves	Unspecified	Unspecified	[184]
	Ogun, Oyo, Osun		Asthma	Leaves	Infusion	Oral	[185]
	Ogun		Tuberculosis	Seeds	Decoction	Unspecified	[89]
	Oyo		Benign prostatic hyperplasia	Leaves	Maceration	Oral	[133]
*Adansonia digitata* L. (Malvaceae)	Borno	Kukaa (H.), Ose (Yor.), Yiri mango (Ig.)	Asthma, Cough	Leaves	Powder	Oral	[34]
	Kebbi, Ogun		Cancer	Bark/Seeds/Leaves	Decoction	Oral	[38, 181]
	Ogun, Oyo, Osun, Ekiti, Lagos		Body cream	Leaves	Juice extract	Topical	[166]
	Yobe		Pile, Diarrhoea	Stem/Leaves	Powder	Oral	[11]
	Kano		Heat rashes	Stem-bark	Unspecified	Unspecified	[35]
	Niger		Toothache, Cough	Bark	Maceration	Oral/Body bath	[161]
	Oyo, Ogun		Wound	Leaves	Decoction/Powder	Wash/Topical	[131]
	Plateau		Pesticide	Leaves	Unspecified	Smoking	[114]
	Kebbi		Poliomyelitis, Smallpox, Yellow fever, Meningitis, Monkey pox, Hepatitis	Stem-bark	Decoction	Oral	[158]
	Plateau		Animal diarrhoea	Leaves	Unspecified	Unspecified	[115]
	Ekiti		Medicine, Fuelwood	Unspecified	Unspecified	Unspecified	[170]
	Sokoto		Diabetes	Root/Bark	Decoction	Oral	[103]
	Oyo		Weight gain in babies, Caries	Bark	Concoction	Unspecified	[58]
	Ondo		Bladder disease	Fruit	Unspecified	Unspecified	[97]
	Southern Nigeria states		Malaria	Leaves/Stem-bark	Decoction	Oral	[17, 59, 85]
	Ekiti, Ondo, Osun, Oyo		Poliomyelitis	Leaves	Unspecified	Unspecified	[57]
	Katsina		Diarrhoea	Bark	Powder	Oral	[61]
*Adenopus breviflorus* Benth. Syn.* Lagenaria breviflora *(Benth.) Roberty (Cucurbitaceae)	Ogun	Gojin jima/Jiímaà (H.), Tagiri (Yor.), Anyummuo (Ig.)	Toxic plant	Fruit	Unspecified	Oral	[165]
	Ogun, Oyo, Osun, Ekiti, Lagos		Cancer	Unripe fruit/Leaves/Seeds	Poultice/Decoction	Topical/Oral	[130, 181]
	Oyo		Intestinal worm	Fruit	Unspecified	Unspecified	[14]
	Kwara		Monkey pox, Smallpox	Leaves	Decoction	Oral	[158]
	Ogun		Measles	Fruit	Unspecified	Contact	[168]
	Osun		Cough	Unspecified	Unspecified	Unspecified	[105]
	Oyo		Vector-borne diseases	Whole plant	Concoction	Oral	[187]
	Oyo		Benign prostatic hyperplasia	Fruit	Maceration	Oral	[133]
	Oyo		Small pox, Purgative	Fruit	Decoction/Contact	Oral/Positioning	[58]
	Rivers		Convulsion, Laxative	Fruit/Leaves	Infusion/Decoction	Body bath/Oral	[41]
	Ekiti, Ondo, Osun, Oyo		Smallpox, Chickenpox, Measles	Fruit/Root/Bark	Unspecified	Unspecified	[57]
*Aframomum melegueta* K. Schum. (Zingiberaceae)	Oyo, Osun	Chitta (H.), Ataare (Yor.), Ose-oji (Ig.)	Cough, Flu	Seeds/Fruit	Pulverising/Decoction	Oral	[96, 105]
	Kwara, Lagos, Ogun, Oyo, Ekiti		Cancer	Seeds/Fruiting shoot	Decoction/Poultice/Powder/Tincture	Oral/Topical	[37, 130, 157, 181]
	Bayelsa		Fracture	Seeds	Poultice	Topical	[60]
	Ogun, Oyo, Osun, Lagos		Diabetes	Fruit	Tincture	Oral	[39]
	Ogun		Stomachache	Seeds	Decoction	Oral	[132]
	Ogun		Guinea worm infestation	Seeds	Unspecified	Unspecified	[159]
	Ogun, Oyo, Osun, Kwara		Male contraceptive	Seeds	Unspecified	Ring/Incision	[42]
	Ogun		Analgesic	Seeds	Unspecified	Unspecified	[90]
	Ondo		Respiratory tract infection	Seeds	Grinding	Oral/inhalation	[17]
	Lagos		Diabetes	Leaves	Concoction	Oral	[111]
	Delta		Smallpox, chickenpox, measles	Leaves/Stem-bark/Seeds	Decoction/Maceration/Aromatherapy/Mastication	Unspecified	[143]
	Oyo		Ringworm	Underground stem	Powder (mix with black soap)	Body bath	[150]
	Oyo, Ogun		Wound	Seeds	Powder	Topical	[131]
	Kebbi, Kwara		Hepatitis, Monkey pox, COVID-19, Poliomyelitis, Yellow fever	Whole plant/Leaves	Decoction/Maceration	Oral/Topical	[158]
	Southern Nigeria states		Malaria	Stem-bark/Leaves/Seeds	Tincture/Decoction	Oral	[59, 167, 178]
	Ogun		Cancer, Haemorrhoids, Pile, Measles, Chickenpox	Seeds/Leaves	Pulverisation/Decoction	Wash/Oral	[82]
	Ogun		Measles	Leaves	Unspecified	Oral	[168]
	Oyo		Neurodegenerative disorders	Seeds	Unspecified	Unspecified	[184]
	Ogun		Measles	Seeds	Unspecified	Unspecified	[186]
	Oyo		Vector-borne diseases	Fruit/Seeds	Powder	Oral	[187]
	Oyo		Problematic pregnancy	Fruit	Incineration	Oral	[183]
	Ogun, Oyo, Osun		Asthma	Rhizomes	Maceration	Oral	[185]
	Ogun		Tuberculosis	Fruit	Tincture	Unspecified	[89]
	Oyo		Benign prostatic hyperplasia	Fruit	Incineration	Oral	[133]
	Kwara		Female sexual dysfunction, Female Infertility	Leaves	Unspecified	Unspecified	[84]
	Oyo		Diarrhoea, Skin diseases, Malaria, Dietary aid	Leaves	Decoction	Unspecified	[58]
	Ondo		Miscarriage	Fruit	Unspecified	Unspecified	[97]
	Rivers		Cough, Liver problem, Worm infestation	Seeds/Root	Mastication	Oral	[41]
	Niger		Tuberculosis, Respiratory disorders	Fruit	Unspecified	Unspecified	[173]
	Akwa Ibom		Cough, Chest pain	Fruit	Mastication	Oral	[85]
	Ekiti, Ondo, Osun, Oyo		Measles, Chickenpox, Poliomyelitis	Leaves/Whole plant	Unspecified	Unspecified	[57]
	Katsina		Cold	Rhizomes	Decoction	Oral	[61]
*Ageratum conyzoides* L. (Asteraceae)	Lagos, Osun	Ahenhen (H.), Imi-esu (Yor.), Ula ujula (Ig.)	Cough, Hypertension	Unspecified	Unspecified	Unspecified	[15, 105]
	Ogun		Analgesic	Leaves	Unspecified	Unspecified	[90]
	Ondo		Cramps, Wounds, Skin rashes	Leaves	Squeezing	Topical/Oral	[17]
	Lagos		Diabetes	Leaves	Juice	Oral	[111]
	Ogun, Oyo, Lagos, Ekiti		Body cream	Whole plant	Dried powder mixed with oil	Topical	[166]
	Delta		Wounds, Skin diseases	Leaves	Infusion/Juice extraction	Unspecified	[143]
	Ogun		Toxic plant	Leaves	Unspecified	Oral	[165]
	Kogi		High blood pressure, Diabetes	Leaves/Stem	Decoction	Oral	[162]
	Oyo		Intestinal worm	Leaves	Unspecified	Unspecified	[14]
	Oyo, Ogun		Wound	Whole plant	Decoction	Topical	[131]
	Kwara		Hepatitis, Lassa fever, Poliomyelitis	Stem-bark	Powder	Oral	[158]
	Ogun		Skin infection	Unspecified	Infusion	Topical	[82]
	Oyo, Osun, Ogun		Cancer	Leaves/Whole plant	Decoction/Paste	Oral/Rubbing	[37, 160, 181]
	Southern Nigeria states		Malaria	Whole plant/Leaves	Decoction/Juice extraction	Unspecified	[59, 176, 178]
	Oyo		Benign prostatic hyperplasia	Leaves	Decoction	Oral	[133]
	Oyo		Dysentery, Malaria, Stimulant	Fruit/Seed	Decoction	Unspecified	[58]
	Rivers		Skin diseases	Leaves	Poultice/Juice	Topical	[41]
	Ogun		Skin diseases, Infectious diseases, Colic, Wound dressing	Unspecified	Unspecified	Unspecified	[182]
	Ekiti, Ondo, Osun, Oyo		Poliomyelitis, Measles, Yellow fever	Leaves/Whole plant	Unspecified	Unspecified	[57]
	Oyo		Constipation, Ulcer, Malaria, Catarrh	Leaves	Squeezing	Oral	[83]
*Allium ascalonicum* L. (Amaryllidaceae)	Oyo, Osun	Albasa maigo (H.), Alubosa elewe (Yor.), Kaanda (ig.)	Cough, Flu	Bulb/Leaves	Grating/Infusion/Decoction/Juice extraction	Oral	[96, 105]
	Kwara, Lagos, Ogun, Oyo, Ekiti		Cancer	Bulb/Whole plant/Leaves	Concoction/Decoction/Infusion	Oral	[130, 157, 181]
	Ogun		Postpartum haemorrhage	Leaves	Decoction	Oral	[132]
	Ogun, Oyo		Guinea worm infestation, Intestinal worm	Bulb/Leaves	Unspecified	Unspecified	[14, 159]
	Ogun, Oyo, Ekiti, Lagos		Body cream	Leaves/Bulb	Juice extraction	Topical	[166]
	Oyo		Children infections	Leaves	Infusion	Oral	[150]
	Ogun		Stomachic, Antiemetic	Root/Leaves	Unspecified	Oral	[168]
	Oyo		Vector-borne diseases	Leaves	Concoction	Oral	[187]
	Ogun, Oyo, Osun		Asthma	Leaves	Decoction	Oral	[185]
	Oyo		Gonorrhoea, Low sperm count	Whole plant	Concoction	Oral	[183]
	Oyo		Malaria, Cough	Leaves	Decoction	Unspecified	[58]
	Rivers		Eye infection	Leaves	Juice extraction	Eye drops	[41]
	Ekiti, Ondo, Osun, Oyo		Chickenpox	Leaves	Unspecified	Unspecified	[57]
*Allium cepa* L. (Amaryllidaceae)	Oyo	Albasa (H.), Alubosa (Yor.), Yabasi (Ig.)	Cough	Bulb	Pulverising	Oral	[96]
	Sokoto, Ogun		Cancer	Leaves/Bulb	Decoction	Oral	[172, 181]
	Ondo		Respiratory tract infection	Leaves	Cutting	Inhalation	[17]
	Lagos		Diabetes	Leaves	Powder	Oral	[111]
	Oyo, Ogun		Wound	Bulb	Squashing	Mopping	[131]
	Plateau		Pesticide	Bulb	Unspecified	Spraying	[114]
	Kebbi		Poliomyelitis, Meningitis, COVID-19	Bulb	Poultice	Inhalation	[158]
	Southern Nigeria states		Malaria	Leaves/Stem/Stem-bark/Bulb	Decoction/Infusion	Unspecified	[59, 167, 176, 178]
	Oyo		Neurodegenerative disorders	Leaves	Unspecified	Unspecified	[184]
	Oyo		Gonorrhoea, Hypertension	Fruit	Maceration	Oral	[183]
	Ogun		Tuberculosis	Bulb	Decoction	Unspecified	[89]
	Katsina		Hypertension, Measles, Cough	Bulb	Unspecified	Unspecified	[91]
	Rivers		Diabetes, Haemorrhoids, Infertility, Ear infection, Scorpion sting, Hypertension	Bulb	Infusion/Juice extraction/Poultice	Oral/Topical	[41]
	Akwa Ibom		Convulsion, Stomach upset, Rheumatism, Vermifuge	Bulb	Pulverisation	Topical/Oral	[85]
*Allium sativum* L. (Amaryllidaceae)	Oyo	Tafarnuwa/Harshen Nasara (H.), Ayu (Yor.), Ayuu (Ig.)	COVID-19	Bulb	Frying/Mastication	Nasal	[96]
	Oyo, Osun		Cough, Flu	Bulb	Frying	Nasal/Oral	[96, 105]
	Borno		Hypertension, Eye pain	Bulb	Mastication	Oral	[34]
	Borno		Boil	Bulb	Poultice	Topical	[34]
	Ogun		Guinea worm infestation	Leaves/Bulb	Unspecified	Unspecified	[159]
	Zamfara		Rheumatoid arthritis	Bulb	Unspecified	Unspecified	[16]
	Ondo		Respiratory tract infection	Leaves	Pounding	Eating/inhaling	[17]
	Lagos, Sokoto		Diabetes	Bulb/Leaves	Decoction/Raw mastication	Oral	[103, 111]
	Ogun, Oyo, Ekiti, Lagos		Body cream	Bulb	Juice extraction	Topical	[166]
	Kano		Pediatric ailments	Bulb	Unspecified	Unspecified	[35]
	Oyo		Intestinal worm	Bulb	Unspecified	Unspecified	[14]
	Oyo, Ogun		Wound	Bulb	Pulverisation	Topical	[131]
	Plateau		Pesticide	Rhizomes	Unspecified	Spraying	[114]
	Kebbi, Sokoto		Poliomyelitis, COVID-19, Monkey pox, Meningitis, Hepatitis	Whole plant/Bulb	Concoction/Decoction	Oral	[158]
	Ogun		Asthma, Rheumatism, Haemorrhoids, Piles	Bulb	Paste/Decoction	Oral/Topical	[82]
	Osun, Oyo		Cancer	Bulb	Unspecified	Unspecified	[160]
	Southern Nigeria states		Malaria	Bulb/Cloves	Maceration/Decoction/Infusion/Tincture	Oral	[59, 129, 146, 176, 178]
	Ogun		Stroke, Eye pain	Root	Mastication	Oral	[168]
	Ogun, Oyo, Osun		Asthma	Bulb	Maceration	Oral	[185]
	Oyo		Gonorrhoea, Hypertension	Bulb	Infusion	Oral	[183]
	Katsina		Catarrh, Cold, Cough, Antibiotic	Bulb	Unspecified	Unspecified	[91]
	Oyo		Malaria, Cough, Preservative	Bulb	Decoction, Soup ingredient	Unspecified	[58]
	Rivers		Hypertension, diabetes, Asthma, cough, haemorrhoids, Burns, Skin disease	Cloves	Mastication/Maceration	Oral/Topical	[41]
	Akwa Ibom		Diabetes, Hypertension, Stomachache, General debility	Cloves	Mastication/Soup	Oral	[85]
	Ekiti, Ondo, Osun, Oyo		Poliomyelitis	Bulb	Unspecified	Unspecified	[57]
	Katsina		Cold	Bulb	Powder	Oral	[61]
*Aloe vera *(L.) Burm.f. (Asphodelaceae)	Ondo	Alobera (H.), Ahon-erin/Eti-erin (Yor.)	Acne, Burns, Acne, Pimples, Eczema, Scabies	Leaves	Infusion	Dermal	[17]
	Lagos		Diabetes	Leaves	Juice	Oral	[111]
	Ogun, Oyo, Ekiti, Lagos		Body cream	Bulb	Juice extraction	Topical	[166]
	Delta		Wound, Skin infections	Leaves	Decoction	Unspecified	[143]
	Oyo, Ogun		Wound	Leaves/Bulb	Paste	Topical	[131]
	Plateau		Pesticide	Latex	Unspecified	Topical	[114]
	Kebbi, Kwara, Sokoto		Meningitis, Hepatitis, Lassa fever	Whole plant/Gel	Decoction/Powder	Oral/Topical	[158]
	Osun, Oyo		Cancer	Leaves	Juice	Unspecified	[160, 181]
	Southern Nigeria states		Malaria	Leaves/Gel	Maceration/Decoction	Oral	[59, 129, 178]
	Rivers		Skin disease, Burns, Haemorrhoids, Diabetes, Menstrual disorder	Leaves	Gel extraction	Topical/Oral	[41]
*Alstonia boonei* De Wild. (Apocynaceae)	Oyo	Ahun/Dokita igbo (Yor.), Eghu (Ig.)	COVID-19	Leaves	Decoction	Oral	[96]
	Oyo		Wound	Stem-bark	Concoction	Oral/Wash	[179]
	Ogun		Breast pain	Stem-bark	Decoction	Oral	[132]
	Ogun, Lagos, Oyo, Osun		Diabetes	Stem-bark/Leaves/Bark	Tincture/Decoction/Powder	Oral	[39, 111]
	Osun		Cough	Unspecified	Unspecified	Unspecified	[105]
	Ogun		Guinea worm infestation	Bark	Unspecified	Unspecified	[159]
	Kwara, Southern Nigeria states		Malaria	Stem-bark/Root/LeavesTwig	Decoction	Oral	[59, 85, 101, 129, 146, 167, 176, 182]
	Ondo		Malaria, Respiratory tract infection; Dysentery	Leaves/bark	Decoction	Oral	[17]
	Kwara		Lassa fever, Yellow fever, Monkey pox, Smallpox	Stem-bark	Concoction	Oral	[158]
	Ogun		Tuberculosis	Unspecified	Decoction	Oral	[82, 168]
	Ogun		Malaria, Body pain	Bark	Unspecified	Oral	[168]
	Oyo		Vector-borne diseases	Bark	Concoction	Oral	[187]
	Ogun		Tuberculosis	Bark	Maceration	Unspecified	[89]
	Ekiti		Medicine, Timber, Fuelwood	Unspecified	Unspecified	Unspecified	[170]
	Ondo		Galactogogue	Stem, latex	Unspecified	Unspecified	[97]
	Rivers		Malaria, Asthma, Cough, Rheumatism	Bark/Leaves	Decoction/Poultice	Oral/Topical	[41]
	Ekiti, Ondo, Osun, Oyo		Jaundice	Leaves	Unspecified	Unspecified	[57]
*Alstonia congensis* Engl. (Apocynaceae)		Gwarndardaji (H.), Awogbo ahun (Yor.), Egbu-ora (Ig.)	Worm infestation, Malaria, Toothache, Bronchitis, Rheumatism, Fever, Diabetes, Genital infections	Unspecified	Unspecified	Unspecified	[163]
	Kwara, Lagos		Cancer (Prostate, breast)	Leaves	Concoction	Oral	[157]
	Ogun		Guinea worm infestation	Bark	Unspecified	Unspecified	[159]
	Ogun		Analgesic	Stem bark	Unspecified	Unspecified	[90]
	Lagos		Diabetes	Root	Decoction	Oral	[111]
	Oyo, Ogun		Wound	Stem-bark	Concoction	Topical	[131]
	Southern Nigeria states		Malaria	Leaves/Bark/Stem-bark	Decoction	Unspecified	[59, 102, 176, 178]
	Ogun		Fevers, Typhoid, Pain	Unspecified	Unspecified	Unspecified	[182]
	Ekiti, Ondo, Osun, Oyo		Chickenpox	Stem-bark	Unspecified	Unspecified	[57]
*Anacardium occidentale* L. (Anacardiaceae)	Kwara, Southern Nigeria states	Fisa/Kashu (H.), Kaju (Yor.), Okpokpo (Ig.)	Malaria	Stem-bark/Leaves/Root/Bark	Decoction/Concoction	Oral	[15, 17, 58, 59, 85, 101, 102, 129, 146, 176, 178]
	Ondo, Osun		Cough, Respiratory tract infection	Bark	Decoction	Oral	[17, 105]
	Sokoto, Kebbi		Cancer	Bark/Root/Leaves	Maceration/Decoction/Powder	Oral/Topical	[38, 172, 181]
	Kaduna, Kano, Katsina, Jigawa		Arthritis	Stem-bark	Decoction	Unspecified	[180]
	Sokoto		Diarrhoea	Leaves	Unspecified	Unspecified	[62]
	Ogun		Burns, Death	Seeds	Unspecified	Oral	[165]
	Kano		Malaria, Heat rashes, Fever	Leaves/Stem-bark	Unspecified	Unspecified	[35]
	Plateau		Aggression/Insomnia	Leaves/Stem-bark	Infusion	Oral	[171]
	Oyo, Ogun		Wound	Seed	Decoction	Wash	[131]
	Kwara, Sokoto		Smallpox, Lassa fever, Yellow fever	Root/Leaf	Decoction	Oral	[158]
	Ekiti, Ondo, Osun, Oyo		Jaundice	Leaves/Stem-bark	Unspecified	Unspecified	[57]
	Ogun		Oral infection	Bark	Unspecified	Oral	[168]
	Ogun, Oyo, Osun		Asthma	Bark	Decoction	Oral	[185]
	Katsina		Chronic cough	Bark/Fruit	Unspecified	Unspecified	[91]
	Rivers		Malaria, Toothache, Ringworm	Bark/Twig/Leaves	Decoction/Mastication/Poultice	Oral/Topical	[41]
	Niger		Tuberculosis, Respiratory disorders	Stem-bark/Leaves	Unspecified	Unspecified	[173]
	Ogun		Malaria, Typhoid, Cough	Unspecified	Unspecified	Unspecified	[182]
	Katsina		Stomachache	Bark	Maceration	Oral	[61]
*Ananas comosus* (L.) Merr. (Bromeliaceae)	Kwara, Lagos, Ogun	Abarba (H.), Ope-oyinbo (Yor.), Akwu-olu (Ig.)	Cancer	Unripe fruit/Leaves	Maceration/Decoction/Juice extraction	Oral	[157, 181]
	Osun		Cough	Unspecified	Unspecified	Unspecified	[105]
	Kwara, Southern Nigeria states		Malaria	Peel/Fruit/Unripe fruit	Decoction/Maceration	Oral	[59, 101, 129, 146, 176, 178]
	Ondo		Respiratory tract infection	Fruit	Concoction/Crushing	Oral	[17]
	Lagos		Diabetes	Fruit	Decoction	Oral	[111]
	Ogun		Toxic plant	Peel	Unspecified	Oral	[165]
	Kebbi, Kwara		Meningitis, Lassa fever, Yellow fever, COVID-19	Bark peel	Concoction/Decoction	Oral	[158]
	Ogun		Asthma	Unspecified	Decoction	Oral	[82]
	Oyo		Benign prostatic hyperplasia	Fruit	Decoction	Oral	[133]
	Katsina		Hypertension, Constipation	Unripe fruit	Unspecified	Unspecified	[91]
*Annickia chlorantha* (Oliv.) Setten & Maas syn. *Enantia chlorantha* Oliv. (Annonaceae)	Kwara, Lagos	Kakerim/Likitana (H.), Awopa (Yor.), Erumeru (Ig.)	Cancer (Breast)	Root	Concoction	Oral	[157]
	Kaduna		Aches, Malaria, Typhoid fever, Hepatitis, Jaundice, Fever	Unspecified	Unspecified	Unspecified	[163]
	Oyo		Wound, Skin disorders	Leaves/Bark/Stem-bark	Powder/Concoction	Topical/Oral/Body bath	[179]
	Ogun		Guinea worm infestation	Bark	Unspecified	Unspecified	[159]
	Kwara, Southern Nigeria states		Malaria	Stem-bark/Bark/Leaves/Root	Decoction/Infusion/Powder	Oral	[58, 59, 101, 129, 146, 176, 178]
	Kwara		Poliomyelitis, Meningitis, Lassa fever, Yellow fever	Stem-bark	Decoction	Oral	[158]
	Osun		Cough	Unspecified	Unspecified	Unspecified	[105]
	Ekiti, Ondo, Osun, Oyo		Jaundice, Poliomyelitis, Measles	Stem-bark/Root	Unspecified	Unspecified	[57]
*Annona senegalensis* Pers. (Annonaceae)	Lagos	Gwandar daji (H.), Abo ibobo (Yor.), Uburu-ọcha (Ig.)	Potency	Unspecified	Unspecified	Oral	[15]
	Kebbi, Borno, Ogun, Oyo, Lagos, Ekiti		Cancer	Leaves/Root	Infusion/Decoction	Oral	[38, 64, 130]
	Kano		Diarrhoea	Leaves	Unspecified	Unspecified	[10]
	Oyo		Flu	Leaves	Decoction	Oral	[96]
	Ogun		Guinea worm infestation	Fruit	Unspecified	Unspecified	[159]
	Kaduna, Kano, Katsina, Jigawa		Arthritis	Root/Leaves	Decoction	Unspecified	[180]
	Niger		Mental illness	Root	Decoction	Bathing	[104]
	Kano		Pediatric ailments	Leaves/Stem-bark/Root	Unspecified	Unspecified	[35]
	Niger		Wounds	Young shoot/Leaves	Juice extraction	Topical	[161]
	Plateau		Psychosis/Depression	Leaves/Stem-bark	Decoction	Oral	[171]
	Lagos		Convulsion, Epilepsy	Bark	Decoction	Oral	[40]
	Kebbi, Sokoto		Poliomyelitis, Hepatitis, Yellow fever	Leaves/Stem/Seeds	Concoction	Oral	[158]
	Osun, Oyo		Cancer	Bark/Root	Unspecified	Unspecified	[160]
	Plateau		Low libido	Leaves	Maceration	Oral	[98]
	Katsina		Snakebite, Yellow fever, Hernia	Stem	Unspecified	Unspecified	[91]
	Sokoto		Diabetes	Leaves/Seed	Maceration	Oral	[103]
	Niger		Female sexual dysfunction, Female Infertility	Leaves	Unspecified	Unspecified	[84]
	Oyo		Diabetes, Venereal disease, Gonorrhoea, Watery sperm	Bark/Leaves	Concoction	Unspecified	[58]
	Niger		Tuberculosis, Respiratory disorders	Root	Unspecified	Unspecified	[173]
*Anogeissus leiocarpa* (DC.) Guill. & Perr. (Annonaceae)	Kano	Marke (H.), Ayin (Yor.), Atara (Ig.)	Stomachache, Cough,	Leaves	Unspecified	Unspecified	[10]
	Kaduna		Yellow fever, Jaundice, Hepatitis, Common cold, Headache,	Unspecified	Unspecified	Unspecified	[163]
	Oyo		Wound	Bark	Decoction	Bathing	[179]
	Ogun		Breast infection	Stem-bark	Decoction	Bathing	[132]
	Kwara, Southern Nigeria states		Malaria	Stem-bark/Leaves/Root	Decoction	Unspecified	[59, 101]
	Sokoto, Borno		Cancer	Leaves/Bark/Root/Stem-bark	Powder/Maceration	Oral/Topical	[64, 172]
	Sokoto, Yobe, Plateau		Diarrhoea	Bark/Stem/Leaves/Stem-bark	Powder	Oral	[11, 62, 115]
	Kano		Pediatric ailments	Leaves/Stem-bark	Unspecified	Unspecified	[35]
	Niger		Stomachache, Worms	Bark	Decoction	Oral	[161]
	Kebbi, Kwara, Sokoto		Monkey pox, Poliomyelitis, Meningitis, Yellow fever, COVID-19	Stem-bark/Leaves/Root	Concoction/Decoction	Oral	[158]
	Oyo		Vector-borne diseases	Bark	Concoction	Oral	[187]
	Ogun, Oyo, Osun		Asthma	Stem-bark	Decoction	Oral	[185]
	Ekiti		Medicine, Fuelwood	Unspecified	Unspecified	Unspecified	[170]
	Katsina		Ulcer, Pile	Bark	Unspecified	Unspecified	[91]
	Sokoto		Diabetes	Leaves/Bark	Maceration/Decoction	Oral	[103]
	Kwara		Female sexual dysfunction, Female Infertility	Bark/Root	Unspecified	Unspecified	[84]
	Oyo		Mouth disease, Dysentery	Root/Bark	Mastication/Concoction	Oral	[58]
	Niger		Tuberculosis, Respiratory disorders	Stem-bark	Unspecified	Unspecified	[173]
	Oyo		High blood pressure	Leaves	Decoction	Oral	[83]
	Katsina		Fever	Bark	Powder	Oral	[61]
*Anthocleista djalonensis* A.Chev. (Gentianaceae)	Lagos	Putaa (H.), Sapo (Yor.), Akpakoro (Ig.)	Hypertension	Unspecified	Unspecified	Unspecified	[15]
	Oyo, Ogun		Wound	Leaves/Root/Stem-bark	Concoction	Oral/Body bath/Topical	[131, 179]
	Lagos		Diabetes	Root/Leaves	Decoction	Oral	[111]
	Lagos		Intestinal problems, Malaria, Jaundice, Skin infections, Hernia	All parts	Decoction	Oral	[40]
	Oyo		Intestinal worm	Root	Unspecified	Unspecified	[14]
	Ogun, Oyo, Osun		Asthma	Bark	Decoction	Oral	[185]
	Oyo		Skin disease, Malaria	Bark	Concoction	Unspecified	[58]
	Rivers		Skin disease, Wound, Stomach ulcer, Stomachache, Asthma, Diabetes, Gonorrhoea, Infertility, Menstrual disorder	Bark/Whole plant	Decoction	Topical/Body bath/Oral	[41]
	Ogun		Wound, Diabetes, Malaria, Dysentery	Unspecified	Unspecified	Unspecified	[182]
	Akwa ibom		Malaria, Gonorrhoea	Root	Decoction/Tincture	Oral	[85]
	Southern Nigeria states		Malaria	Stem-bark	Decoction	Unspecified	[59]
*Argemone mexicana* L. (Papaveraceae)	Ogun	Ƙùùrár fataakee (H.), Egun arugbo/Ekanna ekun/Mafowo-kan-omo-mi (Yor.)	Guinea worm infestation	Leaves/Bark	Unspecified	Unspecified	[159]
	Ogun		Toxic plant	Fruit/Leaves	Unspecified	Oral/Contact	[165]
	Kogi		Anti-ageing, General body vitality	Leaves	Decoction	Oral/Body bath	[162]
	Lagos		Skin infections, Female infertility, Leprosy	Bark	Decoction	Oral	[40]
	Oyo, Ogun		Wound	Leaves	Decoction/Pounding	Wash	[131]
	Southern Nigeria states		Malaria	Leaves	Decoction	Unspecified	[59, 167]
	Ogun		Measles	Leaves/Whole plant	Unspecified	Unspecified	[168, 186]
	Ekiti, Ondo, Osun, Oyo		Hepatitis, Jaundice	Leaves/Whole plant	Unspecified	Unspecified	[57]
	Katsina		Cold	Leaves	Decoction	Steam bath	[61]
*Aspilia africana* (Pers.) C.D. Adams (Asteraceae)	Lagos	Tozalin (H.), Yunyun (Yor.), Orangila (Ig.)	Fibroid, Cough, Purgative	Unspecified	Unspecified	Unspecified	[15]
	Ondo		Wounds, Itching, Rheumatic pains	Leaves	Pulverisation	Topical	[17]
	Delta		Wound dotting	Leaves	Juice extract	Unspecified	[143]
	Kwara		Female sexual dysfunction, Female Infertility	Leaves	Unspecified	Unspecified	[84]
	Rivers		Wound, Bleeding	Leaves	Juice extraction	Topical	[41]
	Southern Nigeria states		Malaria	Leaves	Decoction/Juice extraction	Unspecified	[178]. [59]
	Ogun		Wound, Sores,	Unspecified	Unspecified	Unspecified	[182]
	Ogun		Cancer	Whole plant	Decoction	Oral	[181]
	Akwa Ibom		Bleeding	Leaves	Pulverisation	Topical	[85]
	Oyo		Dysentery, Ulcer	Leaves	Pulverisation	Oral	[83]
*Azadirachta indica* A. Juss. (Meliaceae)	Oyo, Osun, Katsina, Kebbi, Kwara	Darbejiya/Maina (H.), Dongoyaro (Yor.), Atu yabasi (Ig.)	COVID-19, Flu, Cough	Leaves/Bark	Decoction/Infusion/Concoction	Oral/Steam-bath	[96, 105, 158]
	Lagos		Anti-snake bite	Unspecified	Unspecified	Unspecified	[15]
	Ogun, Lagos, Oyo, Osun, Sokoto		Diabetes	Leaves/Fruit//Root	Decoction/Infusion	Oral	[39, 103]
	Borno		Headache, Fever	Leaves/Bark	Concoction	Oral	[34]
	Kebbi		Cancer	Bark	Unspecified	Unspecified	[38]
	Kwara, Southern Nigeria states, Adamawa		Malaria	Leaves/Bark/Seeds/Root/Stem-bark/Flower	Decoction/Infusion/Concoction	Oral	[58, 59, 63, 82, 97, 101, 102, 129, 146, 167, 176, 178]
	Ondo		Malaria, Respiratory tract infection; Skin itching, Eczema	Leaves/Seeds	Decoction/Infusion	Topical/Oral	[17]
	Ogun, Oyo, Lagos, Ekiti		Body cream	Leaves/Bark/Seeds	Maceration	Topical	[166]
	Kano		Pediatric ailments	Leaves	Unspecified	Unspecified	[35]
	Oyo		Intestinal worm	Leaves	Unspecified	Unspecified	[14]
	Oyo, Ogun		Wound	Leaves	Decoction	Oral/Cleansing	[131]
	Plateau		Pesticide	Bark/Leaves/Seeds/Stem-bark	Unspecified	Spreading/Spraying/Topical	[114]
	Kebbi, Kwara, Sokoto		Smallpox, Monkey pox, COVID-19, Poliomyelitis, Yellow fever, Meningitis, Lassa fever	Leaves/Bark	Decoction/Concoction	Oral	[158]
	Benue, Katsina		Fevers	Leaves	Decoction/Maceration	Oral/Body bath	[61, 169]
	Oyo		Vector-borne diseases	Leaves	Smoking	Positioning	[187]
	Plateau		Animal diarrhoea	Leaves	Unspecified	Unspecified	[115]
	Katsina		Yellow fever	Leaves	Unspecified	Unspecified	[91]
	Rivers		Skin disease, Boil, Malaria, Cough	Leaves/Bark	Infusion/Decoction	Topical/Oral	[41]
	Ogun		Malaria, Fevers	Unspecified	Unspecified	Unspecified	[182]
	Ekiti, Ondo, Osun, Oyo		Jaundice	Stem-bark	Unspecified	Unspecified	[57]
	Oyo		Malaria, Measles, Typhoid	Leaves	Decoction	Oral	[83]
*Balanites aegyptiaca* (L.) Delile (Balanitaceae)	Kano, Katsina	Aduwa (H.), Teji (Yor.), Utazi (Ig.)	Gastrointestinal ailments, Stomachache	Leaves	Maceration	Oral	[10, 61]
	Borno		Snakebite	Root	Maceration	Topical	[34]
	Sokoto, Kebbi		Cancer	Bark/Leaves	Decoction	Oral	[38, 172]
	Borno		Yellow fever	Bark	Concoction	Oral	[34]
	Kaduna		Cough, Fever, Parasitic skin diseases, Schistosomiasis, Strong antidote to arrow poison	Unspecified	Unspecified	Unspecified	[163]
	Ogun		Guinea worm infestation	Root/Fruit/Bark	Unspecified	Unspecified	[159]
	Yobe		Pile	Stem	Pulverisation	Oral	[11],
	Kano		Pediatric ailments	Stem-bark/Seeds	Unspecified	Unspecified	[35]
	Kebbi		Hepatitis, Monkey pox, Meningitis, Smallpox, Poliomyelitis	Stem-bark	Decoction	Oral	[158]
	Katsina		Bladder stone	Fruit	Unspecified	Unspecified	[91]
	Sokoto		Diabetes	Leaves/Root/Bark	Maceration/Decoction	Oral	[103]
	Southern Nigeria states		Malaria	Root	Decoction	Unspecified	[59]
*Bambusa vulgaris* Schrad. (Poaceae)	Lagos	Góoràa (H.), Oparun (Yor.)	Cough	Unspecified	Unspecified	Unspecified	[105]
	Ondo		Diabetes, Rashes	Leaves	Decoction/Pounding	Oral	[17]
	Ogun		Toxic plant	Leaves	Unspecified	Oral	[165]
	Southern Nigeria states		Malaria	Root/Leaves	Decoction	Oral/Bathing	[59, 59, 167]
	Ogun, Ondo		Measles, Chickenpox, Gonorrhoea, Syphilis	Leaves	Decoction/Infusion	Oral	[57, 82, 168, 186]
	Kwara		Female sexual dysfunction, Female Infertility	Leaves	Unspecified	Unspecified	[84]
	Ogun		Weight loss, Fevers	Unspecified	Unspecified	Unspecified	[182]
	Ogun		Cancer	Leaves	Decoction	Oral/Bathing	[181]
*Baphia nitida* G.Lodd. (Fabaceae)	Lagos	Majigi (H.), Irosun (Yor.), Aboshi (Ig.)	Divinity	Unspecified	Unspecified	Unspecified	[15]
	Ogun		Guinea worm infestation	Leaves	Unspecified	Unspecified	[159]
	Ogun, Oyo, Lagos, Ekiti		Body cream	Bark/Root	Juice	Dermal	[166]
	Delta, Ogun		Boil	Leaves/Twig/Stem-bark/Roots	Mastication/Poultice	Unspecified	[143, 182]
	Southern Nigeria states		Malaria	Leaves/Stem-bark	Decoction	Unspecified	[59, 167]
	Oyo		Neurodegenerative disorders	Leaves	Unspecified	Unspecified	[184]
	Ogun		Tuberculosis	Bark	Infusion	Unspecified	[89, 182]
	Ondo		Tooth decay	Leaves	Unspecified	Unspecified	[97]
	Akwa Ibom		Gonorrhoea, haemorrhoid	Root-bark	Decoction/Tincture	Oral	[85]
*Boswellia dalzielii* Hutch. (Burseraceae)	Kano	Hano/Harrab (H.),	Stomachache	Leaves	Unspecified	Unspecified	[10]
	Kaduna, Kano, Katsina, Jigawa		Arthritis	Stem-bark	Decoction	Unspecified	[180]
	Sokoto, Niger, Katsina		Diarrhoea, Gastrointestinal disorders, Worms, Pile	Leaves	Maceration/Concoction/Powder	Oral	[61, 62, 161]
	Niger		Mental illness	Stem-bark	Powder/Infusion	Oral	[104]
	Kano		Pediatric ailments	Stem-bark	Unspecified	Unspecified	[35]
	Plateau		Hallucinations/Aggression	Leaves/Bark/Root	Infusion/Incense	Oral/inhalation	[171]
	Kebbi		Poliomyelitis, Smallpox	Stem-bark	Decoction	Oral	[158]
	Plateau		Animal diarrhoea	Unspecified	Unspecified	Unspecified	[115]
	Katsina		Yellow fever	Bark/Leaves	Unspecified	Unspecified	[91]
	Sokoto		Diabetes	Root	Decoction	Oral	[103]
	Borno		Cancer	Stem-bark	Unspecified	Unspecified	[64]
*Bridelia ferruginea* Benth. (Phyllanthaceae)	Kano, Ogun, Sokoto	Kirni/Kizni (H.), Ira/Iralodan (Yor.), Oha/Ede/Ola (Ig.)	Stomachache, Stomach pain, Diarrhoea	Bark/Stem-bark	Unspecified	Unspecified	[10, 62, 132]
	Lagos, Osun, Kaduna		Cough, Birth control	Unspecified	Unspecified	Unspecified	[15, 105, 163]
	Kaduna		Fever, Pain, Dysentery, Laxatives,	Unspecified	Unspecified	Unspecified	[163]
	Oyo		Wound	Bark	Unspecified	Unspecified	[179]
	Ogun, Lagos, Oyo, Osun, Sokoto		Diabetes	Leaves/Bark	Decoction	Oral	[39, 103]
	Ondo		Respiratory tract infection, Malaria	Leaves/Bark	Decoction	Oral	[17]
	Ogun, Oyo, Lagos, Ekiti		Body wash	Leaves	Powder mixed with black soap	Bathing	[166]
	Niger		Dysentery, Whooping cough	Root/Stem	Decoction/Mastication	Oral	[161]
	Ogun, Borno		Cancer	Stem-bark/Bark/Leaves	Decoction	Oral/Bathing	[64, 181]
	Kebbi		Poliomyelitis	Leaves	Decoction	Oral	[158]
	Benue		Fish poison, Arrow poison	Bark	Maceration/Paste	Pouring/Rubbing	[169]
	Ogun, Oyo, Osun		Asthma	Stem-bark	Decoction	Oral	[185]
	Ekiti		Fuelwood	Unspecified	Unspecified	Unspecified	[170]
	Oyo		Dysentery, Teeth disease, Antibacterial, Malaria	Leaves/Stem	Decoction/Mastication	Unspecified	[58]
	Ondo		Mouth wash	Stem	Unspecified	Unspecified	[97]
	Niger		Tuberculosis, Respiratory disorders	Stem-bark/Root	Unspecified	Unspecified	[173]
	Ondo		Malaria	Stem-bark	Decoction	Unspecified	[178]
	Ekiti, Ondo, Osun, Oyo		Hepatitis	Stem-bark	Unspecified	Unspecified	[57]
	Katsina		General well-being	Bark	Decoction	Oral	[61]
*Bryophyllum pinnatum* (Lam.) Oken (Crassulaceae)	Kwara, Lagos, Osun, Oyo, Ogun	Odundun/Abamoda (Yor.), Oda opuye/Okpokoroko (Ig.)	Cancer	Leaves/Root	Concoction	Oral	[37, 157, 160, 181]
	Lagos		Sight, Pimples	Unspecified	Unspecified	Unspecified	[15]
	Osun, Ogun		Cough	Leaves	Incineration	Oral	[82, 105]
	Ogun		Guinea worm infestation	Leaves	Unspecified	Unspecified	[159]
	Ogun		Sedative	Leaves	Unspecified	Unspecified	[90]
	Plateau		Anxiety	Leaves	Decoction	Oral	[171]
	Oyo, Ogun		Wound	Seed	Concoction	Oral	[131]
	Ogun		Tuberculosis	Leaves	Infusion	Unspecified	[89]
	Ondo		Fever, Earache	Leaves	Unspecified	Unspecified	[97]
	Rivers		Ear infection, Cough, Boil, Convulsion	Leaves	Juice extraction	Eye drops/Oral	[41]
	Ondo		Malaria	Leaves	Decoction	Unspecified	[178]
	Ekiti, Ondo, Osun, Oyo		Measles, Hepatitis, Poliomyelitis	Leaves	Unspecified	Unspecified	[57]
*Cajanus cajan* (L.) Millsp. (Fabaceae)	Osun	Aduwa (H.), Ewa otili (Yor.), Fio fio, Hiohio (Ig.)	Cough	Leaves	Concoction	Oral	[105]
	Sokoto		Diarrhoea	Bark	Unspecified	Unspecified	[62]
	Ogun, Oyo, Lagos, Ekiti		Cancer	Leaves/Seed	Poultice/Decoction/Concoction	Topical/Oral	[37, 130, 160]
	Southern Nigeria states		Malaria	Leaves/Stem	Decoction	Unspecified	[59, 146, 167, 176, 178]
	Ogun, Akwa Ibom		Measles, Chickenpox, Smallpox	Leaves/Seed	Decoction/Pulverisation	Oral/Topical	[82, 85, 168]
	Oyo		Malaria, Protein constituent	Leaves/Seed	Concoction/Decoction	Unspecified	[58]
	Ekiti, Ondo, Osun, Oyo		Measles, Chickenpox	Leaves	Unspecified	Unspecified	[57]
*Calotropis procera* (Aiton) Dryand (Asclepiadaceae)	Lagos	Tumifafiya (H.), Bomu-bomu (Yor.), Otosi (Ig.)	Conjunctivitis, Cirrhosis	Leaves	Unspecified	Unspecified	[15]
	Kaduna		Rheumatism, Respiratory diseases	Unspecified	Unspecified	Unspecified	[163]
	Ogun		Lactation problem	Leaves	Decoction	Oral/Washing	[132]
	Osun		Cough	Unspecified	Unspecified	Unspecified	[105]
	Kebbi, Ogun, Osun, Oyo		Cancer	Leaves/Root/Aerial part	Decoction	Oral	[37, 38, 82, 160]
	Ogun		Guinea worm infestation	Leaves	Unspecified	Unspecified	[159]
	Ondo		Skin infection, Inflammation	Leaves	Squeezing/Decoction	Oral/Dermal	[17]
	Ogun, Oyo, Ekiti, Lagos		Poison, Death	Leaves	Unspecified	Oral	[165]
	Kano		Pediatric ailments	Latex/Root	Unspecified	Unspecified	[35]
	Lagos		Malaria, Skin diseases	Leaves/Stem	Decoction	Oral/Topical	[40]
	Plateau		Pesticide	Latex	Unspecified	Topical	[114]
	Kebbi, Kwara		Smallpox, COVID-19, Monkey pox, Poliomyelitis	Leaves	Concoction/Decoction	Oral/Body bath	[158]
	Oyo		Benign prostatic hyperplasia	Leaves	Powder	Oral	[133]
	Katsina		Scorpion sting, Whitlow	Leaves	Unspecified	Unspecified	[91]
	Niger		Tuberculosis, Respiratory disorders	Root/Fruit	Unspecified	Unspecified	[173]
	Ondo		Malaria	Stem-bark/Leaves	Decoction	Unspecified	[178]
	Ogun		Jaundice, Skin diseases, Scorpion sting, Paralysis	Unspecified	Unspecified	Unspecified	[182]
	Ekiti, Ondo, Osun, Oyo		Measles	Leaves	Unspecified	Unspecified	[57]
*Capsicum frutescens* L. (Solanaceae)	Oyo	Taashi (H.), Ata wewe/Ata eiye/Ata ijosi (yor.), Ose mkpe (Ig.)	Cough, COVID-19	Seeds/Fruit	Pulverisation/Infusion/Decoction	Oral	[96]
	Ogun, Lagos, Oyo, Osun		Diabetes	Fruit	Decoction	Oral	[39]
	Ogun		Stomach pain	Seeds	Decoction	Oral	[132]
	Oyo, Ogun		Wound	Leaves	Decoction/Powder	Mopping/Topical	[131]
	Plateau		Pesticide	Pod/Whole plant	Unspecified	Dressing/Positioning	[114]
	Southern Nigeria states		Malaria	Fruit	Decoction	Unspecified	[59, 176]
	Ogun		Measles	Seeds	Unspecified	Unspecified	[186]
	Oyo		Stimulant, Laryngitis	Seeds/Fruit	Soup condiment/Concoction	Oral	[58]
	Niger		Tuberculosis, Respiratory disorders	Fruit	Unspecified	Unspecified	[173]
	Ogun		Wound, Stimulant, Pain, Diabetes	Unspecified	Unspecified	Unspecified	[182]
*Carica papaya* L. (Caricaeae)	Kano	Gwanda (H.), Ibepe (Yor.), Okwuru-ezi/Okwuru-bekee/Mgbimgbi (Ig.)	Dysentery, Stomachache	Leaves, Seeds	Unspecified	Unspecified	[10]
	Oyo, Osun		Flu, Cough	Seeds	Decoction	Oral	[96, 105]
	Ogun, Lagos, Oyo, Osun		Diabetes	Fruit/Root/Seeds	Infusion/Decoction/Powder	Oral	[39, 111]
	Bayelsa		Luxation	Leaves	Poultice	Topical	[60]
	Ogun		Lactation problem	Root	Decoction	Oral/Washing	[132]
	Borno		Stomach pain, Asthma	Leaves	Squeezing	Oral	[34]
	Ogun, Oyo		Guinea worm infestation, Intestinal worm	Leaves/Seeds	Unspecified	Unspecified	[14, 159]
	Kwara, Adamawa, Southern Nigeria states		Malaria	Leaves/Unripe fruit/Seed/Fruit	Infusion/Decoction	Oral	[59, 63, 101, 102, 129, 146, 167, 176, 178]
	Ogun, Oyo, Osun, Kwara		Male contraceptive	Bark	Unspecified	Ring/Decoction	[42]
	Ondo		Respiratory tract infection, Constipation, Pain	Leaves/Seed	Pounding/Squeezing	Oral	[17]
	Ogun, Oyo, Lagos, Ekiti		Body cream	Leaves/Fruit	Juice	Dermal	[166]
	Delta		Eczema, After-shave bumps	Leaves/Fruit (Ripe and Unripe)/Seeds	Unspecified	Unspecified	[143]
	Kano		Pediatric ailments	Leaves	Unspecified	Unspecified	[35]
	Plateau		Psychosis	Leaves	Infusion	Oral	[171]
	Oyo, Ogun		Wound	Fruit/Leaves	Decoction	Topical	[131]
	Katsina, Kebbi, Kwara, Sokoto		Hepatitis, Meningitis, COVID-19, Poliomyelitis, Smallpox, Yellow fever, Monkey pox	Leaves/Seeds	Concoction/Decoction	Oral	[158]
	Ogun		Asthma, Malaria, Hypertension, Typhoid	Leaves	Decoction/Infusion	Oral	[82]
	Oyo		Benign prostatic hyperplasia	Fruit (Ripe)	Decoction	Oral	[133]
	Plateau		Animal diarrhoea	Stem-bark/Seeds	Unspecified	Unspecified	[115]
	Katsina		Boil, Purgative	Unripe fruit	Unspecified	Unspecified	[91]
	Sokoto		Diabetes	Leaves/Seeds/Fruit	Maceration	Oral	[103]
	Oyo		Malaria. Jaundice	Leaves/Fruit	Decoction/Concoction	Oral	[58]
	Ondo		Lactation	Leaves/Latex	Unspecified	Unspecified	[97]
	Rivers		Malaria, Typhoid, Diabetes, Waist pain, Syphilis	Leaves/Fruit/Root	Decoction	Oral	[41]
	Niger		Tuberculosis, Respiratory disorders	Leaves	Unspecified	Unspecified	[173]
	Ogun		Cancer	Fruit	Decoction	Oral	[181]
	Rivers		Diabetes	Leaves/Seeds	Pulverisation	Oral	[85]
	Ekiti, Ondo, Osun, Oyo		Jaundice, Poliomyelitis, Measles	Leaves	Unspecified	Unspecified	[57]
	Katsina		Fever	Leaves	Decoction	Oral	[61]
*Ceiba pentandra *(L.) Gaertn. (Malvaceae)	Ogun	Rimi (H.), Araba (Yor.), Asisaye (Ij.), Akpu-ogwu (Ig.)	Menorrhagia	Bark, Root	Exudate	Oral	[132]
	Bayelsa, Oyo		Luxation, Wound	Bark/Stem-bark	Pulverisation/Concoction	Topical/Oral/Washing	[60, 179]
	Kaduna		Asthma, Colic pain, Conception	Unspecified	Unspecified	Unspecified	[163]
	Ogun		Guinea worm infestation	Bark/Leaves	Unspecified	Unspecified	[159]
	Ogun		Toxic plant	Stem	Unspecified	Oral	[165]
	Plateau		Pesticide	Seeds	Unspecified	Spreading	[114]
	Osun, Oyo		Cancer	Seeds	Unspecified	Unspecified	[160]
	Southern Nigeria states		Malaria	Leaves	Decoction	Unspecified	[59, 146]
	Ekiti		Medicine, Fuelwood	Unspecified	Unspecified	Unspecified	[170]
	Katsina		Diarrhoea	Leaves	Powder	Oral	[61]
*Chromolaena odorata* (L.) R.M.King & H.Rob. syn. *Eupatorium odoratum* L. (Asteraceae)	Lagos	Ewe Akintola (Yor.), Queen Elizabeth/Ekamanturu (Ig.)	Malaria, Potency	Unspecified	Unspecified	Unspecified	[15]
	Oyo, Osun		Flu, Cough	Leaves	Pulverisation/Infusion	Oral	[96, 105]
	Ogun		Sedative	Leaves	Unspecified	Unspecified	[90]
	Ondo		Respiratory tract infection, Wounds, Rashes	Leaves	Decoction	Oral/Topical	[17]
	Oyo, Ogun, Ekiti, Lagos		Body cream	Leaves/Seed	Dried powder mixed with oil	Topical	[166]
	Ogun		Toxic plant	Leaves	Unspecified	Oral	[165]
	Oyo, Ogun		Wound	Leaves	Maceration/Powder	Cleansing/Body bath	[131]
	Southern Nigeria states		Malaria	Aerial part/Root/Leaves/Branch/Flower	Maceration/Decoction/Infusion	Oral	[59, 85, 129, 146, 167, 176, 178]
	Ogun		Tuberculosis, Diarrhoea	Unspecified	Decoction/Infusion	Oral	[82]
	Osun, Oyo		Cancer	Root	Unspecified	Unspecified	[160]
	Oyo		Vector-borne diseases	Whole plant	Cultivation	Dusting	[187]
	Rivers, Oyo		Malaria, Typhoid	Leaves	Decoction	Oral	[41, 83]
	Ogun, Oyo		Burns, Wound, Sores, Skin infections	Leaves	Decoction	Unspecified	[83, 182]
*Citrus limon* (L.) Osbeck (Rutaceae)	Oyo, Akwa Ibom	Lemun tsami (H.), Oronbo/Osan ijaganyin (Yor.), Oroma na gbakasi onu (Ig.)	COVID-19, Cough, Flu	Fruit	Juice extraction	Oral/Topical	[85, 96]
	Ogun		Bacterial vaginosis	Fruit	Squeezing	Oral	[132]
	Ogun, Katsina		Guinea worm infestation, Dewormer	Leaves/Fruit	Unspecified	Unspecified	[91, 159]
	Kwara, Southern Nigeria states		Malaria	Fruit/Leaves	Infusion/Decoction	Unspecified	[59, 101, 167, 176, 178]
	Ogun, Oyo, Osun, Kwara		Male contraceptive	Juice	Decoction	Oral	[42]
	Delta		Spots, Scrabs, Wounds, Scars, Insect bites	Fruit Juice	Decoction/Tincture/Infusion	Unspecified	[143]
	Kano		Fever, Jaundice	Leaves	Unspecified	Unspecified	[35]
	Lagos		Diabetes, Scurvy, Kidney stone disease	Fruit	Infusion	Oral	[40]
	Oyo, Ogun		Wound	Fruit	Juice extraction	Topical	[131]
	Sokoto		Meningitis, COVID-19	Fruit/Leaves	Infusion	Oral	[158]
	Ogun		Tuberculosis	Fruit	Infusion	Unspecified	[89]
	Rivers		Rheumatism, Laxative, Kidney disease, Malaria, Cough, Waist pain	Fruit	Juice extraction/Decoction	Oral	[41]
*Citrus sinensis* (L.) Osbeck (Rutaceae)	Oyo, Borno	Leemu/Lemun zaki (H.), Osan (Yor.), Oroma (Ig.)	Cough	Fruit/Seeds	Lemonade	Oral	[34, 96]
	Kebbi		Cancer	Bark/Fruit	Decoction/Juice extraction	Oral/Topical	[38, 181]
	Ogun, Lagos, Oyo, Osun		Diabetes	Fruit/Seeds	Lemonade/Infusion	Oral	[39]
	Kwara, Adamawa, Southern Nigeria states		Malaria	Stem-bark/Leaves/Fruit	Raw/Infusion/Decoction/Maceration	Oral	[59, 63, 101, 129, 178]
	Ondo		Respiratory tract infection, Ringworm	Fruit	Decoction/Squeezing	Oral	[17]
	Lagos		Fever, Catarrh, Asthma, High blood pressure, Liver ailment	Fruit/All parts	Decoction	Oral	[40]
	Plateau		Pesticide	Fruit/Leaves/Fruit peel	Unspecified	Positioning/Smoking	[114]
	Kebbi		Hepatitis, COVID-19, Yellow fever, Poliomyelitis	Leaves	Decoction	Oral	[158]
	Ogun		Tuberculosis	Unspecified	Juice	Oral	[82]
	Katsina		Scurvy	Fruit	Unspecified	Unspecified	[91]
	Rivers		Laxative, Waist pain, Malaria	Fruit	Juice extraction/Decoction	Oral	[41]
	Ekiti, Ondo, Osun, Oyo		Jaundice	Fruit	Unspecified	Unspecified	[57]
*Citrus* × *aurantiifolia* (Christm.) Swingle (Rutaceae)	Lagos	Babban leemu/Waawan kurmi (H.), Osan wewe (Yor.), Ugiri/Oromankirisi (Ig.)	Cough, Malaria, Constipation	Leaves	Maceration	Oral	[15]
	Ogun, Lagos, Oyo, Osun		Diabetes	Fruit	Lemonade	Oral	[39, 111]
	Oyo, Kaduna, Osun, Ondo		Cough, Expectorant, Respiratory tract infection	Fruit/Leaves	Juice extraction/Decoction/Squeezing	Oral	[17, 96, 105, 163]
	Bayelsa		Infection	Leaves	Poultice	Topical	[60]
	Kwara, Southern Nigeria states		Malaria	Stem-bark/Leaves/Fruit/Twig/Root	Decoction	Oral	[59, 101, 129, 146, 176, 178]
	Ogun, Oyo, Lagos, Ekiti		Body soap	Fruit	Juice mixed with black soap	Body bath	[166]
	Delta		Ringworm, Eczema	Fruit Juice	Aromatherapy/Bath/Decoction/Infusion	Unspecified	[143]
	Yobe		Diarrhoea, Dysentery	Leaves	Powder/Decoction	Oral	[11]
	Lagos, Ogun		Cough, Cold, Fever, Tuberculosis	Fruit	Juice extraction/Infusion	Oral	[40, 89]
	Oyo		Intestinal worm	Fruit	Unspecified	Unspecified	[14]
	Kebbi, Kwara		Yellow fever, Poliomyelitis	Leaves	Decoction	Oral	[158]
	Benue		Typhoid fever	Fruit	Unspecified	Unspecified	[169]
	Ogun		Boil, Cancer	Fruit/Root	Juice/Decoction	Wash/Oral	[82]
	Oyo		Cholera, Gonorrhoea	Fruit juice	Maceration solvent	Oral	[183]
	Oyo		Benign prostatic hyperplasia	Fruit Juice	Maceration solvent	Oral	[133]
	Kwara		Female sexual dysfunction, Female Infertility	Root	Unspecified	Unspecified	[84]
	Oyo		Typhoid, Malaria, Dysentery	Fruit/Leaves/Bark	Decoction/Concoction	Oral	[58]
	Niger		Tuberculosis, Respiratory disorders	Fruit	Unspecified	Unspecified	[173]
	Ogun		Cancer	Bark/Fruit	Decoction/Juice extraction	Oral	[181]
	Akwa ibom		Stomachache	Fruit	Juice extraction	Oral	[85]
	Ekiti, Ondo, Osun, Oyo		Measles, Jaundice, Chickenpox	Leaves/Fruit	Unspecified	Unspecified	[57]
	Katsina		Fever, Nausea	Leaves	Decoction	Steam bath	[61]
*Cocos nucifera* L. (Arecaceae)	Oyo, Osun, Ogun	Kuwapa (H.), Agbon (Yor.), Akubeeke (Ig.)	Cough, Asthma	Pod/Fruit/Husk	Decoction	Oral	[96, 105, 185]
	Oyo		Wound	Root	Unspecified	Unspecified	[179]
	Ogun		Stomachache	Fruit	Unspecified	Unspecified	[132]
	Kwara, Southern Nigeria states		Malaria	Husk/Edible part/Water/Stem-bark/Fruit	Infusion/Decoction	Oral	[59, 101, 178]
	Ondo		Rheumatism, Eczema	Bark	Concoction	Oral	[17]
	Ogun, Oyo, Lagos, Ekiti		Body cream	Nut	Juice extracted as oil	Dermal	[166]
	Delta		Skin diseases	Stem-bark/Root/Fruit	Decoction	Unspecified	[143]
	Oyo		Intestinal worm	Juice	Unspecified	Unspecified	[14]
	Osun. Oyo		Cancer	Juice	Unspecified	Unspecified	[160]
	Ogun		Tuberculosis	Husk	Decoction	Unspecified	[82, 89]
	Oyo		Benign prostatic hyperplasia	Fruit Juice	Maceration solvent	Oral	[133]
	Katsina		Anti-poison, Measles	Nut	Unspecified	Unspecified	[91]
	Oyo		Skin disease, Uterine disease	Nut/Bark	Decoction/Mastication	Oral	[58]
	Akwa Ibom		Oral rehydration therapy	Liquid endosperm	Cracking	Oral	[85]
	Ekiti, Ondo, Osun, Oyo		Poliomyelitis, Measles	Leaves	Unspecified	Unspecified	[57]
*Cola acuminata* (P.Beauv.) Schott & Endl. (Malvaceae)	Ogun	Goro (H.), Obi abata (Yor.), Oji (Ig.)	Diabetes	Leaves	Decoction	Oral	[39]
	Osun		Cough	Unspecified	Unspecified	Unspecified	[105]
	Ogun		Tonic	Stem-bark	Unspecified	Unspecified	[90]
	Lagos		Stomach ulcer, Piles, Male infertility, Dysentery, Diarrhoea	All parts/Nut	Infusion/Mastication	Oral	[40]
	Oyo, Ogun		Wound	Bark/Leaves	Pounding	Oral	[131]
	Osun, Oyo		Cancer	Fruit	Unspecified	Unspecified	[160]
	Ogun		Tuberculosis	Fruit	Infusion	Unspecified	[89]
	Southern Nigeria states		Malaria	Seeds/Stem-bark	Decoction	Unspecified	[59, 178]
	Ogun		Stimulant, Fatigue, Depression, Arthritis, Rheumatism, Diarrhoea, Dysentery	Unspecified	Unspecified	Unspecified	[182]
	Akwa Ibom		Putrid sore	Cotyledons	Powder	Topical	[85]
*Corchorus olitorius* L. (Malvaceae)	Akwa Ibom	Ayoyo/Lalo (H.), Ewedu (Yor.), Ariraa/Ulogburu (Ig.)	Cancer	Root	Infusion	Oral	[85]
	Ogun		Diabetes	Leaves	Decoction	Oral	[39]
	Osun		Cough	Unspecified	Unspecified	Unspecified	[105]
	Ogun		Guinea worm infestation	Leaves	Unspecified	Unspecified	[159]
	Ogun		Toxic plant	Root	Unspecified	Oral	[165]
	Lagos		Diabetes, Aches, Pains, Dysentery, Fever, Piles, Gonorrhoea	Leaves	Concoction/Cold infusion	Oral	[40]
	Southern Nigeria states		Anaemia, Folic acid deficiency, Blood purifiers, Purgative, Diuretics, Febrifuge, Constipation	Leaves/Seed/Root	Decoction/Infusion/Scrapping	Oral	[164]
	Ogun		Asthma	Unspecified	Unspecified	Oral	[82]
	Ogun		Measles	Whole plant	Unspecified	Unspecified	[186]
	Katsina		Blood purifier	Leaves	Unspecified	Unspecified	[91]
	Rivers		Stomachache, Worm infestation, Diarrhoea	Leaves	Infusion	Oral	[41]
	Southern Nigeria states		Malaria	Stem/Leaves	Decoction	Unspecified	[59, 178]
	Ekiti, Ondo, Osun, Oyo		Measles	Whole plant	Unspecified	Unspecified	[57]
	Oyo		Obesity, Difficult delivery, Constipation	Leaves	Juice extraction	Oral	[83]
*Crinum jagus* (J.Thomps.) Dandy (Amaryllidaceae)	Oyo, Lagos, Osun	Albasar kwadi (H.), Ogede-odo (Yor.), Edè-chúkwú (Ig.)	Cough, Flu	Bulb	Pulverisation/Grating/Infusion/Pounding	Oral	[15, 96, 105]
	Ondo		Respiratory tract infection, Inflammation	Leaves	Infusion	Oral	[17]
	Lagos		Diabetes	Bulb	Maceration	Oral	[111]
	Oyo		Abscess	Leaves	Maceration	Oral	[150]
	Plateau		Pesticide	Bulb/Leaves/Whole plant	Unspecified	Oral/Positioning/Spraying	[114]
	Ogun, Osun, Oyo		Cancer	Tuber/Root	Decoction	Oral	[160, 181]
	Ogun, Osun, Oyo		Asthma, Tuberculosis	Root/Leaves	Maceration/Concoction	Oral	[168, 185]
	Oyo		Convulsion, Cough	Root	Concoction	Oral	[58]
*Croton gratissimus var. gratissimus* syn. *Croton zambesicus* Müll.Arg. (Euphorbiaceae)	Lagos	Koriba (H.), Ajẹ́ kòfòlé (Yor.)	Hypertension, Malaria	Unspecified	Unspecified	Unspecified	[15]
	Ogun		Guinea worm infestation	Leaves	Unspecified	Unspecified	[159]
	Ogun		Toxic plant	Leaves/Seeds	Unspecified	Oral	[165]
	Ogun, Oyo, Lagos, Ekiti		Cancer	Stem-bark/Root	Decoction/Concoction	Oral	[130, 181]
	Plateau		Aggression/Mania	Leaves	Infusion	Oral	[171]
	Oyo, Ogun		Wound	Leaves	Squashing	Wash/Body bath	[131]
	Oyo		Vector-borne diseases	Leaves	Decoction	Unspecified	[187]
	Plateau		Animal diarrhoea	Unspecified	Unspecified	Unspecified	[115]
	Rivers, Akwa Ibom		Diarrhoea, Dysentery, Malaria	Leaves	Decoction	Oral	[41, 85]
*Cryptolepis nigrescens* (Wennberg) L.Joubert & Bruyns syn. *Parquetina nigrescens* (Wennberg) Bullock syn. (Apocynaceae)	Lagos, Ogun	Kwankwani (H.), Ewe-ogbo/Ewidun (Yor.), Mgbidingbe (Ig.)	Cancer	Leaves/Root	Concoction/Decoction	Oral	[157, 181]
	Ogun		Guinea worm infestation	Leaves	Unspecified	Unspecified	[159]
	Oyo		Wound	Root	Concoction	Oral/Body bath	[179]
	Kwara, Southern Nigeria states		Malaria	Leaves/Whole plant	Decoction/Infusion	Oral	[59, 82, 101, 146, 178]
	Ondo		Pile, Skin lesions, Respiratory tract infection	Leaves	Infusion	Topical/Oral	[17]
	Southern Nigeria states		Anti-sickling agent, Gastroenteritis, Gonorrhoea, Jaundice, Rickets, Asthma	Leaves/Whole plant	Unspecified	Unspecified	[177]
*Curcuma longa* L. (Zingiberaceae)	Ogun	Gangamau (H.), Ata ile pupa (Yor.), Boboch (Ig.)	Diabetes	Rhizomes	Decoction	Oral	[39]
	Oyo, Ondo		COVID-19, Respiratory tract infection, Inflammation	Rhizomes/Leaves/Root	Decoction/Grinding	Oral	[17, 96]
	Kwara, Southern Nigeria states		Malaria	Rhizome/Leaves	Decoction/Infusion	Unspecified	[59, 101, 146]
	Sokoto		Cancer	Unspecified	Unspecified	Unspecified	[172]
	Zamfara		Rheumatoid arthritis	Root	Unspecified	Unspecified	[16]
	Oyo, Ogun, Ekiti, Lagos		Body cream	Rhizomes	Dried powder mixed with oil	Topical	[166]
	Kwara		Female sexual dysfunction, Female Infertility	Root	Unspecified	Unspecified	[84]
	Rivers, Oyo		Intestinal worm, Worm infestation, Jaundice, Skin disease, Eye infection	Rhizomes	Decoction	Oral/Topical	[14, 41]
*Cymbopogon citratus* (DC.) Stapf (Poaceae)	Oyo, Osun	Chiawa sami (H.), Ewe tii/Koriko oba (Yor.), Ahihia lemon (Ig.)	Cough, Flu, COVID-19	Leaves	Decoction/Infusion	Oral	[96, 105]
	Lagos		Longevity	Unspecified	Unspecified	Unspecified	[15]
	Bayelsa		Fracture, Wound, Irritation	Leaves	Poultice	Topical	[60]
	Kwara, Adamawa, Southern Nigeria states		Malaria	Leaves/Whole plant	Infusion/Decoction/Concoction	Oral	[58, 59, 63, 85, 97, 101, 102, 129, 146, 167, 168, 176, 178]
	Ogun		Analgesic	Leaves	Unspecified	Unspecified	[90]
	Kaduna, Kano, Katsina, Nige		Arthritis	Leaves	Infusion	Unspecified	[180]
	Ondo		Respiratory tract infection, Inflammation	Leaves	Infusion	Inhalation/Oral	[17]
	Ogun, Oyo, Ekiti, Lagos		Cancer	Leaves/Rhizome	Concoction/Powder mixed with black soap	Oral/Body bath	[130, 181]
	Lagos		Malaria, High blood pressure, Diarrhoea, Fever	Leaves	Decoction	Oral/Bathing	[40]
	Oyo		Children scalp infections	Leaves	Decoction	Oral/Body bath	[150]
	Plateau		Mosquito repellant	Leaves	Smoking	Positioning	[114]
	Kwara, Sokoto, Ogun		Hepatitis, Jaundice, Yellow fever	Leaves	Decoction/Infusion	Oral	[82, 158]
	Oyo		Vector-borne diseases	Leaves	Decoction	Unspecified	[187]
	Ogun		Tuberculosis	Leaves	Infusion	Unspecified	[89]
	Ogun		Hypotension, Pain, Emetic, Convulsion	Unspecified	Unspecified	Unspecified	[182]
	Akwa Ibom		Malaria	Leaves	Decoction	Oral	[85]
	Ekiti, Ondo, Osun, Oyo		Jaundice	Leaves	Unspecified	Unspecified	[57]
*Daniellia oliveri* (Rolfe) Hutch. & Dalziel (Fabaceae)	Kaduna	Maje (H.), Iyaa (Yor.), Ozabwa/Agba (Ig.)	Dysentery, Diarrhoea, Toothache, Urinary infection, Stomachache, Wound	Unspecified	Unspecified	Unspecified	[163]
	Oyo		Wound	Stem-bark	Decoction	Bathing	[179]
	Ogun		Stomach pain	Stem-bark	Decoction	Oral	[132]
	Kwara		Malaria	Stem-bark	Unspecified	Unspecified	[101]
	Niger, Plateau		Dysentery, Animal diarrhoea	Bark/Leaves	Maceration	Oral	[115, 161]
	Plateau		Hallucination	Stem-bark	Infusion	Oral/Bathing	[171]
	Benue		Gastroenteritis, Kwashiorkor	Leaves	Fermentation	Oral	[169]
	Ogun		Rashes	Bark	Unspecified	Oral	[168]
	Sokoto		Diabetes	Bark	Decoction	Oral	[103]
	Niger		Tuberculosis, Respiratory disorders	Mistletoes	Unspecified	Unspecified	[173]
*Detarium microcarpum* Guill. & Perr. (Fabaceae)	Kano	Taura (H.), Ogbogbo/Arira (Yor.), Ofor (Ig.)	Stomachache	Leaves/Bark	Unspecified	Unspecified	[10]
	Kaduna		Tuberculosis, Leprosy, Intestinal worm, Malaria, Rheumatism, Stomachache, Pile	Unspecified	Unspecified	Unspecified	[163]
	Oyo		Wound, Skin disorders	Stem-bark	Concoction	Bathing	[179]
	Niger, Yobe, Ogun		Dysentery, Joint pain	Bark/Leaves/Root	Concoction/Powder	Bathing/Oral	[11, 161, 168]
	Borno		Cancer	Root/Stem-bark	Unspecified	Unspecified	[64]
	Niger		Tuberculosis, Respiratory disorders	Stem-bark	Unspecified	Unspecified	[173]
	Ondo		Malaria	Stem-bark	Decoction	Unspecified	[178]
	Ekiti, Ondo, Osun, Oyo		Hepatitis	Stem-bark	Unspecified	Unspecified	[57]
	Katsina		Pile	Bark	Maceration	Oral	[61]
*Elaeis guineensis* Jacq. (Arecaceae)	Oyo, Osun	Kwakwa/Banje/Farakaye (H.), Eyin/Ope (Yor.), Mkpuruaku (Ig.)	Cough, Respiratory tract infection	Seeds/Bark/Fruit	Infusion/Decoction	Oral	[17, 96, 105]
	Lagos		Pile, Blood tonic	Unspecified	Unspecified	Unspecified	[15]
	Kwara, Lagos, Osun, Oyo, Ogun		Cancer	Bark/Kernel/Fruit/Leaves/Seeds	Concoction/Juice/Soaking	Oral	[157, 160, 181]
	Kaduna		Bronchitis, Asthma, Gonorrhoea, Menorrhagia, Wound, Abdominal pain, Skin infection	Unspecified	Unspecified	Unspecified	[163]
	Bayelsa		Luxation, Fracture	Leaves/Frond ribs	Pulverisation/Cut into pieces	Topical/Splint	[60]
	Ogun, Oyo		Guinea worm infestation, Intestinal worm	Bark/Seeds/Fruit/Kernel	Unspecified	Unspecified	[14, 159]
	Oyo, Ogun, Ekiti, Lagos, Delta, Ondo		Body cream, Skin infection, Skin troubles	Nut/Oil/Unripe kernel/Fruit/Leaves	Oil extraction/Pulverisation/Decoction/Mastication/Syrup	Dermal/Aromatherapy	[17, 143, 166]
	Ogun		Noxious plant	Leaves	Unspecified	Oral	[165]
	Oyo, Ogun		Wound	Seeds	Concoction	Mopping	[131]
	Plateau		Pesticide	Fruit/Kernel	Unspecified	Positioning/Topical	[114]
	Ekiti, Ondo, Osun, Oyo		Chickenpox, Measles	Root	Unspecified	Unspecified	[57]
	Kwara		Hepatitis, Meningitis, Lassa fever, Yellow fever, Poliomyelitis	Root	Decoction	Oral	[158]
	Southern Nigeria states		Malaria	Leaves/Fruit/Root	Decoction	Unspecified	[59, 176, 178]
	Oyo		Neurodegenerative disorders	Leaves	Unspecified	Unspecified	[184]
	Ogun		Measles	Leaves	Unspecified	Unspecified	[186]
	Oyo		Antimicrobial agent, Coolant	Seeds	Unspecified	Unspecified	[183]
	Ogun		Tuberculosis	Oil	Soup	Unspecified	[89]
	Oyo		Benign prostatic hyperplasia	Stem-bark	Powder	Oral	[133]
	Katsina		Easy flow menses	Nut	Unspecified	Unspecified	[91]
	Rivers, Akwa ibom		Boil	Fruit pericarp	Peeling	Topical	[41, 85]
	Ogun		Wound, Gonorrhoea, Menorrhagia, Bronchitis, Skin diseases	Unspecified	Unspecified	Unspecified	[182]
*Erythrophleum suaveolens* (Guill. & Perr.) Brenan (Fabaceae)	Oyo, Ogun	Baska (H.), Erun-obo (Yor.), Inyi (Ig.)	Wound	Stem-bark	Concoction/Incense	Bathing/Smoking	[131, 179]
	Osun		Cough	Unspecified	Unspecified	Unspecified	[105]
	Ogun		Guinea worm infestation	Bark/Leaves	Unspecified	Unspecified	[159]
	Ogun, Oyo, Lagos, Ekiti		Body cream	Leaves	Dried powder mixed with oil	Dermal	[166]
	Ogun		Toxic plant	Leaves/Root/Stem/Bark/Seeds	Unspecified	Oral	[165]
	Oyo		Abscess	Bark	Powder	Bathing	[150]
	Ogun		Skin irritation	Bark	Concoction	Bathing	[168]
	Plateau		Animal diarrhoea	Unspecified	Unspecified	Unspecified	[115]
	Ogun		Medicine, Fuelwood	Unspecified	Unspecified	Unspecified	[170]
	Oyo		Evil spirit repellant	Bark	Decoction/Incense	Oral/Smoking	[58]
	Ogun		Cancer	Bark/Seeds	Decoction/Paste	Oral/Wash	[181]
	Ekiti, Ondo, Osun, Oyo		Chickenpox	Stem-bark	Unspecified	Unspecified	[57]
*Euphorbia hirta* L. (Euphorbiaceae)	Kwara, Lagos, Oyo, Ogun	Nóónòn kúrcíyáá (H.), Emile (Yor.), Oba ala (Ig.)	Cancer	Stem/Shoot/Whole plant/Leaves	Decoction/Infusion/Concoction	Oral/Topical	[37, 130, 157, 181]
	Ogun, Delta		Lactation problem	Leaves	Grinding/Decoction/Infusion/Aromatherapy	Washing/Oral/Topical	[132, 143]
	Ondo		Eczema, Wounds	Leaves	Infusion	Dermal	[17]
	Sokoto, Plateau		Diarrhoea, Animal diarrhoea	Aerial part	Unspecified	Unspecified	[62, 115]
	Ogun		Toxic plant	Sap	Unspecified	Oral	[165]
	Plateau		Insomnia/Depression	Leaves	Infusion	Inhalation/Steambath	[171]
	Kwara		Hepatitis, Meningitis, Lassa fever, Yellow fever, Poliomyelitis, Monkey pox, Smallpox, COVID-19	Stem-bark	Decoction	Oral	[158]
	Ogun, Oyo, Osun, Rivers, Akwa ibom		Asthma	Whole plant	Decoction/Maceration	Oral	[41, 82, 85, 185]
	Ondo		Poison antidote	Whole plant	Unspecified	Unspecified	[97]
	Niger		Tuberculosis, Respiratory disorders	Whole plant	Unspecified	Unspecified	[173]
	Ondo		Malaria	Leaves	Decoction	Unspecified	[178]
	Ogun		Respiratory diseases, Skin conditions, Digestive problems	Unspecified	Unspecified	Unspecified	[182]
*Euphorbia lateriflora* Schumach. (Euphorbiaceae)	Ogun, Osun, Oyo	Fidda sartse (H.), Enu opiri (Yor.)	Guinea worm infestation	Leaves/Whole plant/Root	Unspecified	Unspecified	[14, 37, 159, 181]
	Ogun		Toxic plant	Root/Sap	Unspecified	Oral	[165]
	Oyo, Ogun, Lagos, Ekiti, Borno		Cancer	Root/Leaves/Whole-stem/Aerial part/Whole plant	Infusion/Powder	Topical/Oral	[64, 130]
	Oyo		Abscess	Leaves	Decoction	Oral/Bathing	[150]
	Oyo, Ogun		Wound	Leaves	Maceration/Grounding	Cleansing/Bathing	[131]
	Kwara		Smallpox	Stem-bark	Decoction	Oral/Body bath	[158]
	Ogun		Typhoid	Root	Unspecified	Oral	[168]
	Ogun, Osun, Oyo		Asthma	Stem	Maceration	Oral	[185]
	Oyo		Skin diseases, Hives	Stem/Leaves	Decoction/Powder	Oral/Topical	[58]
	Ondo		Ear infections	Leaves/Root	Unspecified	Unspecified	[97]
	Ekiti, Ondo, Osun, Oyo		Chickenpox	Leaves	Unspecified	Unspecified	[57]
*Ficus exasperata* Vahl (Moraceae)	Oyo	Gamji (H.), Epin/Ewe-ipin (Yor.), Awinrinwa/Asesa (Ig.)	Wound	Stem	Exudate	Topical	[179]
	Lagos, Osun		Cough, Hypertension	Unspecified	Unspecified	Unspecified	[15, 105]
	Ogun		Diabetes	Root	Decoction	Oral	[39]
	Kebbi, Osun, Oyo		Cancer	Bark/Root	Unspecified	Unspecified	[38, 160]
	Kwara, Southern Nigeria states		Malaria	Leaves/Stem-bark	Decoction	Unspecified	[59, 101, 102, 178]
	Ogun		Analgesic	Leaves	Unspecified	Unspecified	[90]
	Ondo		Respiratory tract infection, Boils, Ringworm	Leaves	Pounding/Decoction	Topical	[17]
	Ogun		Toxic plant	Leaves/Root/Sap	Unspecified	Contact/Oral	[165]
	Ogun		Boil, Ringworm, Skin infection	Leaf	Pulverisation/Scratching	Topical	[82]
	Sokoto		Diabetes	Leaves/Root/Bark	Maceration/Decoction	Oral	[103]
	Rivers		Bleeding, Wound, Stomachache, Eye infection, Cough, Fibroid, Urinary tract infection, Skin disease, Boil	Leaves/Bark/Root	Juice extraction/Decoction	Topical/Oral/Eye drops	[41]
	Southern Nigeria states		Sexually transmitted diseases, Gastroenteritis, Microbial infections, Hypertension, Sores	Leaves/Sap	Unspecified	Oral/Topical	[177]
	Ogun		Hypertension, Wound, Arthritis	Unspecified	Unspecified	Unspecified	[182]
*Gambeya albida* (G.Don) Aubrév. & Pellegr. syn. *Chrysophyllum albidum *G.Don (Sapotaceae)	Ogun	Agwaliba (H.), Agbalumo (Yor.), Udala (Ig.)	Guinea worm infestation	Leaves/Bark	Concoction	Oral	[159]
	Osun		Cough	Unspecified	Unspecified	Unspecified	[105]
	Ogun		Toxic plant	Unripe fruit	Unspecified	Oral	[165]
	Lagos		Yellow fever, Malaria, Skin infection, Gonorrhoea, Urinary tract infections	Root/Stem	Decoction	Oral	[40]
	Southern Nigeria states		Malaria	Stem-bark/Leaves	Decoction	Unspecified	[59, 146]
	Ogun, Oyo, Osun		Asthma	Stem-bark	Maceration	Oral	[185]
	Oyo		Cholera	Seeds	Maceration	Oral	[183]
	Oyo		Benign prostatic hyperplasia	Leaves	Powder	Oral	[133]
	Ekiti		Medicine, Fuelwood, Fruit	Unspecified	Unspecified	Unspecified	[170]
	Katsina		Anti-nausea	Fruit	Unspecified	Unspecified	[91]
	Rivers		Malaria, Urinary tract infection, Impotence	Stem/Seeds/Whole plant	Decoction/Powder	Oral	[41]
	Ogun		Cancer	Bark/Seeds	Decoction/Powder	Oral	[181]
	Ogun		Guinea worm infestation	Leaves/Bark	Concoction	Oral	[159]
*Garcinia kola* Heckel (Clusiaceae)	Oyo, Osun, Katsina	Namijin goro (H.), Orogbo (Yor.), Akuilu (Ig.)	Cough, Flu	Seeds/Fruit	Infusion/Pulverisation	Oral	[91, 96, 105]
	Ogun		Breast pain	Stem-bark	Decoction	Oral	[132]
	Bayelsa		Fracture	Leaves	Poultice	Topical	[60]
	Ogun		Diabetes	Fruit/Root	Maceration	Oral	[39]
	Ogun		Guinea worm infestation	Root/Bark	Unspecified	Unspecified	[159]
	Kwara, Southern Nigeria states		Malaria	Stem-bark/Leaves/Fruit/Seeds	Maceration/Infusion/Decoction	Oral	[59, 101, 102, 129, 146, 167, 176, 178]
	Ondo		Respiratory tract infection	Seeds	Pounding/Decoction	Oral	[17]
	Lagos		Diabetes	Root	Decoction	Oral	[111]
	Plateau		Pesticide	Fruit	Unspecified	Positioning	[114]
	Kwara		Hepatitis, Meningitis, Lassa fever, Yellow fever, Poliomyelitis	Root	Decoction	Oral	[158]
	Ogun		Asthma, Cough, Cancer, Rheumatism, Tuberculosis	Seeds	Pulverisation/Mastication/Decoction	Oral	[82]
	Osun, Oyo, Ogun		Cancer	Root/Seeds/Stem-bark	Tincture/Decoction	Oral	[37, 160, 181]
	Ogun		Body pain	Bark	Unspecified	Oral	[168]
	Oyo, Osun, Ogun		Asthma	Seeds	Maceration	Oral	[185]
	Ogun		Tuberculosis	Leaves	Decoction	Unspecified	[89]
	Oyo		Benign prostatic hyperplasia	Seeds	Pulverisation	Oral	[133]
	Plateau		Erectile dysfunction	Nut	Unspecified	Oral	[98]
	Kwara		Female sexual dysfunction, Female Infertility	Seeds	Unspecified	Unspecified	[84]
	Oyo		Cough, Bronchitis, Throat, Respiratory disorders	Seeds/Root/Bark	Mastication/Decoction	Oral	[58]
	Ondo		Hepatoprotective	Seeds	Unspecified	Unspecified	[97]
	Rivers, Akwa ibom		Cough, Chest pain, Liver problem	Seeds	Mastication	Oral	[41, 85]
	Ekiti, Ondo, Osun, Oyo		Smallpox	Root	Unspecified	Unspecified	[57]
*Gossypium barbadense* L. (Malvaceae)	Oyo, Ogun	Gwandi (H.), Owu akese (Yor.), Oluluogho/Owula (Ig.)	Wound	Seeds/Leaves	Decoction/Incineration	Oral/Washing/Topical	[131, 179]
	Osun		Cough	Unspecified	Unspecified	Unspecified	[105]
	Kwara, Adamawa, Southern Nigeria states		Malaria	Leaves/Bark/Root/Seeds	Decoction	Oral	[59, 63, 101, 146, 176, 178]
	Lagos		Female infertility, Typhoid fever, High blood pressure	All parts	Decoction	Oral	[40]
	Ogun, Oyo, Osun		Asthma	Seeds	Maceration	Oral	[185]
	Ogun		Hypertension, Urinary-tract infections, Skin diseases	Unspecified	Unspecified	Unspecified	[182]
	Ekiti, Ondo, Osun, Oyo		Hepatitis	Leaves	Unspecified	Unspecified	[57]
	Katsina		Diarrhoea	Leaves	Powder	Oral	[61]
*Gossypium hirsutum* L. (Malvaceae)							
	Ondo	Auduga (H.), Owu/ela-owu (Yor.)	Respiratory tract infection, Skin rash	Leaves/Seed	Pounding/Decoction	Oral/dermal	[17]
	Oyo		Children scalp infections	Seed	Decoction	Oral	[150]
	Ogun, Oyo		Wound	Leaves	Incineration	Topical	[131]
	Southern Nigeria states		Malaria	Leaves	Decoction	Unspecified	[59, 146]
	Rivers		Gonorrhoea, Haemorrhoid	Leaves	Infusion	Oral	[41]
	Akwa ibom		Gonorrhoea, Sore throat	Leaves/Root	Decoction/Infusion	Oral	[85]
*Guiera senegalensis* J.F.Gmel. (Combretaceae)	Kano	Sabara (H), Olofun (Yor)	Dysentery, Diarrhoea, Pile	Leaves	Unspecified	Unspecified	[10]
	Borno		Diarrhoea	Leaves	Pounding	Oral	[34]
	Kaduna		Arthritis, Rheumatism, Diarrhoea, Dysentery, Venereal diseases, Hypertension, Diabetes, Malaria, Fever, Stomach complains	Unspecified	Unspecified	Unspecified	[163]
	Kebbi, Sokoto		Cancer	Leaves/Bark/Seeds/Root	Powder/Decoction	Oral/Topical	[38, 172]
	Yobe		Dysentery, Diarrhoea, Pile, Ulcer	Leaves/Root	Powder	Oral	[11]
	Plateau		Psychosis	Leaves	Maceration	Oral	[171]
	Katsina, Kebbi, Sokoto		Poliomyelitis, Yellow fever, Smallpox, COVID-19, Meningitis, Hepatitis, Monkey pox	Leaves/Bark/Root	Decoction	Oral	[158]
	Adamawa		Malaria	Bark/Root	Direct	Unspecified	[63]
	Katsina		Ulcer, Pile	Leaves	Unspecified	Unspecified	[91]
	Sokoto		Diabetes	Leaves/Root	Maceration/Decoction	Oral	[103]
	Katsina		Nausea, Vomiting, Diarrhoea, General welbeing	Leaves	Powder	Oral	[61]
*Harungana madagascariensis *Lam. ex Poir. (Hypericaceae)	Oyo	Alíllìbár rààfíí (H.), Asunje (Yor.), Nyemu nyemu (Ig.)	Wound	Bark	Decoction	Oral/Topical	[179]
	Lagos		Anti-snake bite	Unspecified	Unspecified	Unspecified	[15]
	Bayelsa		Fracture, Itching	Leaves	Poultice/Sap	Topical	[60]
	Southern Nigeria states		Malaria	Stem-bark/Leaves/Yellow leaf	Decoction	Unspecified	[59, 101, 178]
	Ogun		Tonic	Stem-bark	Unspecified	Unspecified	[90]
	Delta		Skin diseases—Itches/Leprous spots	Plant sap/Leaves/Stem-bark	Decoction/Topical	Unspecified	[143]
	Kwara		Hepatitis, Meningitis, Poliomyelitis	Leaves	Decoction	Oral	[158]
	Ogun, Osun, Oyo		Asthma	Bark	Maceration	Oral	[185]
	Ekiti		Medicine, Fuelwood	Unspecified	Unspecified	Unspecified	[170]
	Oyo		Anaemia	Bark/Leaves	Concoction	Oral	[58]
	Southern Nigeria states		Microbial skin infections, Worm infestation, Gastroenteritis, Anemia, Malaria, Fever	Leaves/Stem-bark	Unspecified	Unspecified	[177]
	Ogun		Malaria, Pain, Inflammation	Unspecified	Unspecified	Unspecified	[182]
	Ekiti, Ondo, Osun, Oyo		Jaundice	Stem-bark	Unspecified	Unspecified	[57]
*Heliotropium indicum* L. (Boraginaceae)	Kwara, Lagos, Ogun	Kalkashin korama (H.), Ogbe-akuko/Agogo igun (Yor.),	Cancer	Whole plant/Leaves/Root	Concoction/Decoction	Oral	[157, 181]
	Lagos		Erection, Pile	Unspecified	Unspecified	Unspecified	[15]
	Ogun		Guinea worm infestation	Leaves	Unspecified	Unspecified	[159]
	Ondo		Respiratory tract infection, Pains, Small pox, Wounds	Leaves	Concoction/Infusion	Oral/Topical	[17]
	Delta		Sting, Insect bites, Boils	Leaves/Whole plant	Decoction/Infusion	Unspecified	[143]
	Kogi		Gastrointestinal disorders, Worms, Convulsion	Leaves/Stem/Flower	Infusion	Oral	[162]
	Ogun		Body pain	Root	Unspecified	Oral	[168]
	Southern Nigeria states		Malaria	Leaves	Decoction	Unspecified	[59, 176]
	Ondo		Mouth wash	Fruit/Leaves	Unspecified	Unspecified	[97]
	Ekiti, Ondo, Osun, Oyo		Measles	Leaves	Unspecified	Unspecified	[57]
	Akwa ibom		Sore throat, Boil	Leaves	Decoction	Oral	[85]
*Jatropha curcas* L. (Euphorbiaceae)	Oyo, Osun	Bini da Zugu (H.), Botuje/lapalapa funfun (Yor.), odo- ala(Ig.)	Cough	Fruit	Decoction	Oral	[96, 105]
	Oyo, Ogun		Wound	Root, Stem-bark,	Exudate	Topical	[131, 179]
	Lagos		Pile, Sight	Unspecified	Unspecified	Unspecified	[15]
	Ogun		Menorrhagia	Leaves	Decoction	Body bath	[132]
	Borno		Wound, Ringworm	Leaves	Latex	Topical	[34]
	Ogun		Guinea worm infestation	Root/Stem/Leaf/Seeds	Unspecified	Unspecified	[159]
	Kwara, Southern Nigeria states		Malaria	Leaves/Stem-bark (Dried)	Decoction	Unspecified	[59, 101, 176, 178]
	Ondo		Skin spots, Diarrhoea	Leaves/Latex	Squeezing latex	Topical/Oral	[17]
	Lagos		Diabetes	Leaves	Decoction	Oral	[111]
	Ogun		Toxic plant	Leaves	Unspecified	Oral	[165]
	Ogun, Oyo, Lagos, Ekiti, Osun		Cancer	Root/Stem-bark/Leaves	Decoction	Topical/Oral	[130, 160] [181]
	Plateau		Psychosis/Aggression	Leaves	Infusion	Oral	[171]
	Lagos		Asthma, Malaria fever, Eczema	Root/Leaves	Decoction/Infusion	Oral	[40]
	Plateau		Pesticide	Leaves/Stem/Seeds/Stem-bark	Unspecified	Topical	[114]
	Kwara		Meningitis, Lassa fever	Leaves	Decoction	Oral	[158]
	Benue		Earache	Leaves/Bark	Warming/Squeezing	Ear drops	[169]
	Ogun		Gonorrhoea, Syphilis	Seeds	Incineration	Oral	[82]
	Oyo		Neurodegenerative disorders	Fruit	Unspecified	Unspecified	[184]
	Ogun		Stomach pain	Leaves	Unspecified	Unspecified	[91]
	Plateau		Erectile dysfunction	Root	Decoction	Oral	[98]
	Plateau		Animal diarrhoea	Leaves/Stem-bark/Whole plant	Unspecified	Unspecified	[115]
	Sokoto		Diabetes	Leaves/Root/Bark	Maceration/Decoction	Oral	[103]
	Kwara		Female sexual dysfunction, Female Infertility	Leaves	Unspecified	Unspecified	[84]
	Rivers		Skin disease, Toothache, Syphilis	Leaves/Twig/Root	Juice extraction/Poultice/Mastication/Decoction	Topical/Oral	[41]
	Niger		Tuberculosis, Respiratory disorders	Root/Leaves	Unspecified	Unspecified	[173]
	Ogun		Stomachache, Dysentery, Pile, Ringworm	Unspecified	Unspecified	Unspecified	[182]
	Akwa Ibom		Skin disease, Laxative, Convulsion	Seed/Leaves	Oil extraction/Pulverisation	Oral	[85]
	Ekiti, Ondo, Osun, Oyo		Poliomyelitis	Leaves	Unspecified	Unspecified	[57]
	Katsina		General well-being	Leaves	Maceration	Oral	[61]
*Jatropha gossypifolia* L. (Euphorbiaceae)	Lagos, Osun	Bini da Zugu (H.), Lapalapa/Botuje pupa (Yor.), Ake mgogho (Ig.)	Cough, Pile	Unspecified	Unspecified	Unspecified	[15, 105]
	Ogun		Oligomenorrhea	Leaves	Squeezing	Oral	[132]
	Ondo		Pile, Respiratory tract infection	Leaves/Latex	Concoction	Topical/Oral	[17]
	Ogun, Oyo, Lagos, Ekiti		Cancer	Root/Stem-bark/Leaves	Decoction	Topical/Oral	[130, 160]
	Oyo		Vector-borne diseases	Seeds	Powder	Topical	[187]
	Southern Nigeria states		Malaria	Leaves	Decoction/Juice extraction	Unspecified	[59, 178]
	Ogun		Wound, fevers, Carbuncles, Eczema, Itches, Sores, Swollen breasts, Stomachache	Unspecified	Unspecified	Unspecified	[182]
*Khaya grandifoliola* C.DC. (Meliaceae)	Oyo	Madachi (H.), Oganwo (Yor.), Odala (Ig.)	Wound	Stem-bark	Concoction	Oral/Bathing	[179]
	Osun		Cough	Unspecified	Unspecified	Unspecified	[105]
	Ogun		Analgesic	Stem bark	Unspecified	Unspecified	[90]
	Southern Nigeria states		Malaria	Stem-bark	Decoction/Infusion	Unspecified	[59, 146, 167]
	Ekiti		Timber, Fuelwood	Unspecified	Unspecified	Unspecified	[170]
*Khaya ivorensis* A.Chev. (Meliaceae)	Kwara, Southern Nigeria states	Oganwo (Yor.), Onu (Ig.)	Malaria	Stem-bark	Decoction	Unspecified	[59, 101]
	Lagos		Diabetes	Bark	Soaking	Oral	[111]
	Oyo, Ogun, Ekiti, Lagos		Body cream	Bark	Dried powder mixed with oil	Dermal	[166]
	Osun, Oyo		Intestinal worm	Bark	Unspecified	Unspecified	[14]
	Oyo		Children scalp infections	Bark	Decoction	Oral/Bathing	[150]
	Osun, Oyo, Ogun		Cancer	Bark/Stem-bark	Decoction	Oral/Bathing	[37, 160, 181]
	Ogun		Anemia	Bark	Unspecified	Oral	[168]
	Ogun, Osun, Oyo		Asthma	Bark	Maceration	Oral	[185]
	Plateau		Erectile dysfunction	Bark	Infusion	Oral	[98]
	Ekiti		Timber, Fuelwood	Unspecified	Unspecified	Unspecified	[170]
	Ekiti, Ondo, Osun, Oyo		Chickenpox, Jaundice	Stem-bark	Unspecified	Unspecified	[57]
*Khaya senegalensis* (Desr.) A.Juss.) (Meliaceae)	Borno, Niger	Madaci (H.), Ògbògbò (Yor.), Onu (Ig.)	Pile, Stomachache, Anaemia	Bark	Maceration	Oral	[34, 161]
	Kebbi, Sokoto, Borno		Cancer	Root/Bark/Stem-bark	Powder/Decoction	Oral/Topical	[38, 64, 172]
	Yobe, Kano		Pile, Dysentery, Diarrhoea	Stem/Bark	Powder	Oral	[10, 11]
	Plateau		Psychosis	Leaves/Stem-bark	Infusion	Oral	[171]
	Plateau		Pesticide	Stem-bark/Leaves/Seeds	Unspecified	Topical	[114]
	Benue		Fevers, Typhoid	Bark	Maceration	Oral	[169]
	Oyo		Malaria	Leaves/Stem	Unspecified	Unspecified	[176]
	Plateau		Erectile dysfunction	Bark	Infusion	Oral	[98]
	Plateau		Animal diarrhoea	Stem-bark	Unspecified	Unspecified	[115]
	Sokoto		Diabetes	Bark	Decoction	Oral	[103]
	Oyo		Skin disease, Malaria	Bark	Decoction/Concoction	Oral	[58]
*Kigelia africana* (Lam.) Benth. (Bignoniaceae)	Oyo	Rawuya (H.), Pandoro (Yor.), Amo-ibi/Uturubien (Ig.)	Flu	Bark	Pulverisation/Infusion	Oral/Topical	[96]
	Kwara, Lagos, Ogun, Oyo, Ekiti, Osun		Cancer	Bark/Fruit/Leaves/Root	Decoction	Oral/Topical	[37, 82, 97, 130, 157, 160]
	Oyo		Wound	Fruit/Bark	Concoction	Oral/Washing	[179]
	Lagos, Osun		Cough, Pile	Unspecified	Unspecified	Unspecified	[15, 105]
	Kwara, Southern Nigeria states		Malaria	Stem-bark/Fruit/Root	Infusion/Decoction	Unspecified	[59, 101]
	Ogun		Toxic plant	Stem	Unspecified	Contact with fluid	[165]
	Niger		High blood pressure, Stomachache, Yellow fever	Root/Bark/Fruit	Concoction	Oral	[161]
	Lagos		Obesity, Digestive disorder, Constipation, Venereal diseases, Rheumatism	Unspecified	Decoction	Oral/Topical	[40]
	Plateau		Pesticide	Fruit	Unspecified	Positioning	[114]
	Kwara		Hepatitis, Yellow fever, Poliomyelitis	Leaves	Decoction	Oral	[158]
	Benue		Pelvic inflammatory disease	Leaves	Decoction	Oral	[169]
	Oyo		Vector-borne diseases	Bark	Powder	Oral	[187]
	Ogun, Oyo, Osun		Asthma	Stem-bark	Maceration	Oral	[185]
	Plateau		Animal diarrhoea	Stem-bark	Unspecified	Unspecified	[115]
	Oyo		Malaria, Dysentery, Gonorrhoea, Rheumatism	Bark/Stem/Root/Fruit	Concoction	Oral	[58]
	Ekiti, Ondo, Osun, Oyo		Poliomyelitis, Chickenpox, Measles	Leaves	Unspecified	Unspecified	[57]
*Lawsonia inermis* L. (Lythraceae)	Kaduna	Marandaa/Lalle (H.), Laali (Yor.)	Gonorrhoea, Fever, Dysentery, Leprosy, Skin diseases, Diarrhoea	Unspecified	Unspecified	Unspecified	[163]
	Lagos		Malaria, Gonorrhoea	Unspecified	Unspecified	Unspecified	[15]
	Ogun		Dysmenorrhea	Leaves/Flower	Paste	Oral	[132]
	Kebbi, Borno		Cancer	Root/Leaves	Unspecified	Unspecified	[38, 64]
	Borno		Typhoid	Leaves	Pounding	Oral	[34]
	Osun		Cough	Unspecified	Unspecified	Unspecified	[105]
	Borno		Whitlow	Leaves	Pounding	Topical	[34]
	Ogun		Guinea worm infestation	Leaves	Unspecified	Unspecified	[159]
	Kwara, Southern Nigeria states		Malaria	Leaves/Fruit/Twig	Decoction	Unspecified	[59, 101, 167, 176, 178]
	Lagos		Diabetes	Leaves	Decoction	Oral	[111]
	Ogun, Oyo, Lagos, Ekiti		Body cream	Leaves/Bark/Seeds	Maceration	Topical	[166]
	Lagos		Malaria, Fever, Dysentery, Diarrhoea, Sore throat, Liver problems, Toothache	Bark/Leaves	Decoction/Infusion	Oral	[40]
	Oyo, Ogun		Wound	Leaves	Decoction	Oral/Cleansing	[131]
	Plateau		Pesticide	Flower	Unspecified	Topical	[114]
	Kebbi, Kwara		Monkey pox, Meningitis, COVID-19, Yellow fever, Lassa fever	Leaves	Decoction	Oral	[158]
	Ogun		Typhoid	Leaves	Unspecified	Oral	[168]
	Oyo		Vector-borne diseases	Leaves	Squeezing	Oral/Bathing	[187]
	Ekiti, Ondo, Osun, Oyo		Poliomyelitis, Measles	Leaves	Unspecified	Unspecified	[57]
*Lophira alata* Banks ex C.F.Gaertn. (Ochnaceae)	Kaduna	Kujeme (H.), Ekki (Yor.), Akufo/Okopia (Ig.)	Kidney problem, Menstrual problems, Convulsion, Epilepsy, Eye problem, Yaws, Cough, Fever, Jaundice	Unspecified	Unspecified	Unspecified	[163],
	Oyo		Wound	Stem-bark	Concoction	Oral/Bathing	[179]
	Kwara, Southern Nigeria states		Malaria	Stem-bark	Infusion	Unspecified	[59, 101, 167]
	Oyo, Ogun, Ekiti, Lagos		Skin infection	Bark	Mixed with black soap	Bathing	[166]
	Niger		Mental illness	Stem-bark	Unspecified	Unspecified	[104]
	Ogun		Typhoid	Bark	Unspecified	Oral	[168]
	Ondo		Respect/Favour	Leaf	Unspecified	Unspecified	[97]
	Ogun		Cancer	Bark	Decoction	Oral	[181]
	Ekiti, Ondo, Osun, Oyo		Jaundice	Stem-bark	Unspecified	Unspecified	[57]
*Mangifera indica* L. (Anacardiaceae)	Oyo, Osun	Mangwaro (H.), Mongoro (Yor.), Mangoro (Ig.)	Cough	Bark	Decoction	Oral	[96, 105]
	Lagos		Malaria, Fever	Unspecified	Unspecified	Unspecified	[15]
	Oyo		Wound, Skin disorders	Bark	Concoction	Body bath	[179]
	Kaduna		Malaria, Dysentery, Rheumatism, Malaria, Diarrhoea, Active laxative	Unspecified	Unspecified	Unspecified	[163]
	Kwara, Lagos, Sokoto, Kebbi, Osun, Oyo		Cancer	Leaves/Bark	Maceration/Decoction	Oral	[38, 157, 160, 172]
	Ogun, Kano, Katsina		Stomach pain, Diarrhoea, Dysentery	Fruit/Leaves/Stem/Root/Bark	Infusion	Unspecified	[10, 91, 132]
	Borno		Haemorrhoids	Bark	Infusion	Oral	[34]
	Bayelsa, Kaduna, Kano, Katsina, Jigawa		Fracture, Arthritis	Leaves/Stem-bark	Poultice/Decoction	Topical	[60, 180]
	Kwara, Adamawa, Southern Nigeria states		Malaria	Stem-bark/Leaves/Bark/Fruit/Immature stem	Decoction	Oral	[59, 63, 101, 102, 146, 167]
	Ogun		Sedatives	Stem-bark	Unspecified	Unspecified	[90]
	Ondo		Malaria, Respiratory tract infection; Bleeding, Piles	Fruit/Leaves	Decoction	Oral	[17]
	Lagos, Sokoto		Diabetes	Leaves/Bark	Decoction/Maceration	Oral	[103, 111]
	Oyo, Ogun, Ekiti, Lagos		Face cleanser	Fruit	Juice extraction	Topical	[166]
	Delta		Skin disease	Leaves/Stem-bark/Fruit	Decoction/Maceration	Body bath	[143]
	Yobe		Ulcer	Leaves/Stem	Powder/Decoction	Oral	[11]
	Katsina, Kebbi, Kwara, Sokoto		Yellow fever, Hepatitis, Meningitis, Lassa fever, Monkey pox, Small pox, COVID-19	Leaves/Stem-bark	Decoction/Concoction	Oral	[158]
	Benue		Dysentery	Leaves/Bark	Unspecified	Unspecified	[169]
	Ogun		Jaundice, Yellow fever	Unspecified	Decoction	Oral	[82]
	Oyo, Anambra, Ondo		Malaria	Leaves/Bark//Stem-bark/Root-bark	Decoction	Unspecified	[129, 176, 178]
	Rivers		Malaria, Headache, Jaundice, Skin diseases	Leaves/Bark	Infusion/Juice	Oral/Topical	[41]
	Oyo		Malaria, Juice production	Leaves/Bark/Fruit	Concoction	Oral	[58]
	Ogun		Fever, Skin disorders, Diabetes, Malaria	Unspecified	Unspecified	Unspecified	[182]
	Akwa ibom		Hypertension	Leaves	Decoction	Oral	[85]
	Ekiti, Ondo, Osun, Oyo		Jaundice, Yellow fever	Stem-bark/Leaves	Unspecified	Unspecified	[57]
	Katsina		General well-being	Bark	Maceration	Oral	[61]
*Milicia excelsa* (Welw.) C.C.Berg (Moraceae)	Oyo, Ogun	Loko (H.), Iroko (Yor.), Oji (Ig.)	Wound	Stem-bark	Concoction	Oral	[131, 179]
	Ogun		Guinea worm infestation	Leaves/Bark	Unspecified	Unspecified	[159]
	Niger		Mental illness	Stem-bark	Powder/Infusion	Oral	[104]
	Osun, Oyo		Cancer	Bark	Unspecified	Unspecified	[160]
	Southern Nigeria states		Malaria	Stem-bark/Root	Decoction	Unspecified	[59, 146]
	Ekiti		Timber, Fuelwood	Unspecified	Unspecified	Unspecified	[170]
	Oyo		Neutralizer, Spiritual deliverance from oppression	Root/Fruit/Bark/Seeds	Concoction	Unspecified	[58]
	Rivers		Haemorrhoid, Wound, Rheumatism	Stem/Bark	Decoction/Poultice/Infusion	Oral/Topical	[41]
	Ogun		Coughs, Heart problems, Sedatives, Painful disorders, Wound, Tumours	Unspecified	Unspecified	Unspecified	[182]
*Momordica charantia* L. (Cucurbitaceae)	Oyo, Osun	Garafuni (H.), Ejinrin (Yor.), Akban ndene (Ig.)	Respiratory disorders, Cough	Leaves	Decoction	Oral	[96, 105]
	Ogun, Lagos		Diabetes	Root/Leaves	Decoction	Oral	[39, 111]
	Ogun		Dysmenorrhea	Leaves	Decoction	Oral	[132]
	Kwara, Lagos, Osun, Oyo, Borno		Cancer	Leaves/Aerial part/Whole plant	Concoction/Decoction	Oral	[64, 157, 160, 181]
	Ogun		Guinea worm infestation	Leaves/Whole plant	Unspecified	Unspecified	[159]
	Zamfara		Rheumatoid arthritis	Leaves	Unspecified	Unspecified	[16]
	Ondo		Pile, Indigestion, Respiratory tract infection, Rashes, Sores	Leaves	Concoction	Topical/Oral	[17]
	Oyo, Ogun, Ekiti, Lagos		Body cream	Leaves	Powder	Topical	[166]
	Ogun		Toxic plant	Seeds/Root	Unspecified	Oral	[165]
	Yobe		Ulcer	Leaves	Powder/Decoction	Oral	[11]
	Plateau		Psychosis	Fruit	Decoction	Oral	[171]
	Oyo		Abscess	Leaves	Decoction	Oral/Body bath	[150]
	Kebbi, Kwara		Meningitis, Lassa fever, Yellow fever, Poliomyelitis, Monkey pox, Small pox, COVID-19	Leaves	Decoction/Concoction	Oral	[158]
	Ogun		Malaria, haemorrhoid, Pile, Diabetes, Ringworm	Leaves	Infusion/Squeezing/Decoction	Oral/Topical	[82]
	Southern Nigeria states		Malaria	Whole plant/Vine/Leaves/Fresh fruit	Infusion/Decoction/Juice extraction	Unspecified	[59, 102, 176, 178]
	Ogun		Measles	Whole plant	Unspecified	Unspecified	[186]
	Oyo		Vector-borne diseases	Leaves	Decoction	Oral	[187]
	Rivers		Diabetes, Worm, Infertility	Leaves/Fruit	Decoction	Oral	[41]
	Ekiti, Ondo, Osun, Oyo		Jaundice, Yellow fever	Whole plant	Unspecified	Unspecified	[57]
	Oyo		Pile, Kidney stone, Low immunity, Haemorrhoid	Leaves	Squeezing	Oral	[83]
*Morinda lucida* Benth. (Rubiaceae)	Oyo, Lagos	Oruwo (Yor.), Eze-ogu/Ogere (Ig.)	Flu, COVID-19, Cough	Leaves/Bark	Pulverisation/Infusion/Decoction	Oral	[96, 105]
	Lagos		Jaundice, Fever	Unspecified	Unspecified	Unspecified	[15]
	Oyo		Wound	Bark	Decoction	Oral	[179]
	Ogun, Lagos		Diabetes	Root/Leaves	Decoction/Squeezing	Oral	[39, 111]
	Kwara, Southern Nigeria states		Malaria	Stem-bark/Leaves/Root/Bark/Twig	Decoction/Concoction/Juice extraction	Oral	[59, 101, 102, 146, 167, 176, 183]
	Ondo		Fever, Respiratory tract infection, Skin infection	Leaves	Decoction	Oral/Topical	[17]
	Ogun, Oyo, Lagos, Ekiti		Cancer	Leaves/Stem-bark/Bark/Root	Infusion/Decoction/Paste	Oral/Topical	[82, 130, 181] [37, 82]
	Kwara		Hepatitis, Lassa fever, Yellow fever	Root	Concoction	Oral	[158]
	Benue		Fevers	Leaves	Maceration	Oral	[169]
	Oyo		Vector-borne diseases	Root	Powder	Oral	[187]
	Oyo		Benign prostatic hyperplasia	Leaves	Maceration	Oral	[133]
	Ondo, Oyo, Anambra		Malaria	Leaves	Decoction	Oral	[58, 83, 97, 129, 178]
	Ogun		Tumour, Fevers	Unspecified	Unspecified	Unspecified	[182]
	Ekiti, Ondo, Osun, Oyo		Jaundice, Yellow fever	Leaves/Root	Unspecified	Unspecified	[57]
*Moringa oleifera* Lam. (Moringaceae)	Kano	Zogale (H.), Ewe igbale (Yor)	Gastrointestinal disorders, Ulcers, Cough, Neck pain	Leaves/Flower	Decoction	Oral	[10]
	Lagos		Cough	Unspecified	Unspecified	Unspecified	[15]
	Borno		Ulcer	Leaves/Flower	Squeezing	Oral	[34]
	Kebbi, Sokoto, Borno, Oyo		Cancer	Leaves/Root	Decoction	Oral/Inhalation	[38, 64, 160, 172]
	Ogun		Sedatives, Analgesic	Leaves	Unspecified	Unspecified	[90]
	Zamfara		Rheumatoid arthritis	Leaves/Fruit	Unspecified	Unspecified	[16]
	Ondo		Respiratory tract infection; Skin infection, Pain, Insomnia	Leaves	Concoction	Oral/Dermal	[17]
	Yobe, Katsina, Plateau		Pile, Diarrhoea, Dysentery, Abdominal disorders, Animal diarrhoea	Leaves/Stem-bark/Seeds	Powder/Decoction	Oral	[11, 91, 115]
	Kano		Pediatric ailments	Root	Unspecified	Unspecified	[35]
	Katsina, Kebbi, Kwara, Sokoto		Yellow fever, Hepatitis, Meningitis, Smallpox, Monkey pox, COVID-19, Poliomyelitis	Leaves/Root	Decoction/Mastication/Concoction	Oral	[158]
	Benue		Eyes infection	Leaves	Squeezing	Eye drops	[169]
	Southern Nigeria states		Malaria	Leaves/Seeds/Stem-bark	Maceration/Decoction	Oral	[129, 146],, [59, 178]
	Sokoto		Diabetes	Leaves/Bark/Root	Infusion/Decoction	Oral	[103]
	Katsina		Anemia	Leaves	Direct	Oral	[61]
*Musa* × *sapientum* L. (Musaceae)	Kwara, Lagos	Ayaba (H.), Ogede wewe (Yor.), Unere (Ig.)	Cancer (Breast, Prostate)	Tuber	Concoction	Oral	[157]
	Kano		Pediatric ailments	Leaves	Unspecified	Unspecified	[35]
	Katsina, Kebbi, Kwara		Hepatitis, Hepatitis, Meningitis, Lassa fever, Poliomyelitis	Leaves/Root	Decoction	Oral	[158]
	Ogun		Eczema	Leaves	Ashing	Topical	[82]
	Southern Nigeria states		Malaria	Fruit/Leaves/Fruit peel	Decoction	Oral	[129, 146],, [59, 178]
	Oyo		Neurodegenerative disorders	Fruit	Unspecified	Unspecified	[184]
	Ogun, Oyo, Osun		Asthma	Fruit	Mastication	Oral	[185]
	Oyo		Problematic delivery	Inflorescence	Concoction	Oral	[183]
	Katsina		High blood pressure	Fruit	Unspecified	Unspecified	[91]
	Rivers		Ulcer	Leaves	Decoction	Oral	[85]
*Musa x paradisiaca* L. (Musaceae)	Oyo, Osun	Agade/Ayaba (H.), Ogede-agbagba (Yor.), Jioko/Oji-Oko (Ig.)	Flu, Cough	Leaves	Extracted juice	Oral	[96, 105]
	Ogun		Diabetes	Fruit	Peeling	Eaten raw	[39]
	Ogun		Guinea worm infestation	Leaves	Unspecified	Unspecified	[159]
	Kwara, Southern Nigeria states		Malaria	Leaves	Decoction	Oral/Body bath	[59, 101, 176, 178]
	Oyo, Ogun		Wound	Stem-bark	Incineration	Topical	[131]
	Ogun		haemorrhoids, Pile	Unspecified	Unspecified	Topical	[82]
	Oyo		Neurodegenerative disorders	Leaves	Unspecified	Unspecified	[184]
	Katsina		High iron, Potent astringent	Leaves	Unspecified	Unspecified	[91]
	Kwara		Female sexual dysfunction, Female Infertility	Fruit	Unspecified	Unspecified	[84]
	Rivers		Impotence, Aphrodisiac, Menstrual disorder, Urinary tract infection, Hypertension, Snake bite, Measles	Root/Stem/Leaves	Infusion/Extracted juice	Oral/Topical	[41]
	Ogun		Cancer	Leaves	Unspecified	Unspecified	[181]
	Ogun		Measles	Stem (decayed)	Pulverisation	Topical	[85]
	Oyo		Ulcer, Convulsion, Epilepsy	Peels	Powder	Oral	[83]
*Nauclea latifolia* Sm. syn. *Sarcocephalus latifolius* (Sm.) E.A.Bruce. (Rubiaceae)	Kwara, Lagos, Ogun, Oyo, Ekiti	Tiwon biri (H.), Egbesi (Yor.), Odo-uburu (Ig.)	Cancer	Seeds/Root/Leaves	Decoction	Oral/Body bath	[130, 157, 181]
	Lagos		Malaria, Pimples, Diabetes	Unspecified	Unspecified	Unspecified	[15]
	Osun, Ogun, Oyo, Niger		Cough, Asthma, Tuberculosis, Respiratory disorders	Root	Maceration	Oral	[105, 173, 185]
	Oyo		Wound	Leaves/Root/Stem-bark	Concoction	Body bath	[179]
	Kwara, Southern Nigeria states		Malaria	Stem-bark/Leaves/Root/Stem	Concoction/Decoction/Maceration	Oral	[101, 129, 178],, [58, 59, 85, 102, 146, 167]
	Ogun, Niger		Analgesic, Waist and Back pain	Root	Decoction	Body bath/Oral	[90, 161]
	Ogun		Toxic plant	Leaves	Unspecified	Contact	[165]
	Plateau		Psychosis/Aggression	Leaves/Bark/Root	Decoction	Oral/Inhalation	[171]
	Oyo		Children scalp infections	Root	Decoction	Oral/Body bath	[150]
	Kwara		Hepatitis, Lassa fever, Poliomyelitis	Root	Maceration	Oral	[158]
	Benue		Guinea worm	Leaves/Root	Decoction	Oral	[169]
	Ogun, Ekiti, Ondo, Osun, Oyo		Measles, Chickenpox, Jaundice	Root/Stem-bark	Decoction	Oral	[57, 82]
	Ogun		Jaundice, Yellow fever	Root	Unspecified	Oral	[168]
	Plateau		Animal diarrhoea	Leaves/Stem-bark/Root	Unspecified	Unspecified	[115]
	Sokoto		Diabetes	Root	Decoction	Oral	[103]
	Kwara		Female sexual dysfunction, Female Infertility	Root	Unspecified	Unspecified	[84]
	Rivers		Skin disease, Gonorrhoea	Root	Decoction	Oral	[41]
	Ogun		Fevers, Headaches, Migraines, Microbial infections, Inflammation	Unspecified	Unspecified	Unspecified	[182]
*Newbouldia laevis* (Beauverd) Seem. (Bignoniaceae)	Lagos	Aduruku (H.), Akoko (Yor.), Ogirisi (Ig.)	Diabetes	Unspecified	Unspecified	Unspecified	[15]
	Bayelsa		Luxation, Damaged connective tissues,	Leaves	Poultice	Topical	[60]
	Ogun		Guinea worm infestation	Leaves	Unspecified	Unspecified	[159]
	Ondo, Niger		Tuberculosis, Respiratory disorders	Leaves/Stem-bark	Boiling	Oral	[17, 173]
	Delta		Septic wound, Bleeding, Skin disease	Leaves/Stem-bark/Root	Decoction/Infusion/Topical/Leaf juice/Poultice	Unspecified	[143]
	Ogun		Toxic plant	Leaves/Root	Unspecified	Oral	[165]
	Kogi		Stomach upset, Indigestion, Flatulence	Stem-bark	Decoction	Oral	[162]
	Plateau		Psychosis/Insomnia	Leaves	Infusion	Oral/Steam bath	[171]
	Ogun, Oyo		Wound	Leaves	Steaming	Topical	[131]
	Benue		Constipation, Fevers	Bark	Unspecified	Unspecified	[169]
	Ogun		Cough, Jaundice, Yellow fever, Hypertension	Leaves	Infusion/Decoction	Oral	[82]
	Southern Nigeria states		Malaria	Leaves	Decoction	Unspecified	[59, 176]
	Ogun		Measles	Leaves	Unspecified	Unspecified	[186]
	Oyo		Vector-borne diseases	Leaves	Decoction	Oral	[187]
	Ekiti		Medicine, Traditional rights	Unspecified	Unspecified	Unspecified	[170]
	Kwara		Female sexual dysfunction, Female Infertility	Leaves	Unspecified	Unspecified	[84]
	Ogun		Gastrointestinal disorders, Rheumatic swelling, Syphilis	Unspecified	Unspecified	Unspecified	[182]
	Akwa ibom		Boil/Dysmenorrhoea	Stem-bark/Leaves	Decoction	Oral	[85]
	Oyo		Infertility	Leaves/Stem-bark	Infusion	Oral	[83]
*Nicotiana tabacum* L. (Solanaceae)	Oyo, Osun, Ogun	Taba (H.), Ewe-taba (Yor.), Anwere (Ig.)	Flu, Cough, Asthma, Tuberculosis, Respiratory disorders	Leaves	Infusion/Maceration	Oral	[96, 105, 173, 185]
	Kwara, Lagos, Ogun, Oyo		Cancer, Benign prostatic hyperplasia	Leaves	Powder/Decoction	Topical/Oral	[133, 157, 181]
	Ondo		Rheumatic swelling, Piles, Heart diseases, Depression	Leaves	Infusion	Oral	[17]
	Lagos		Diabetes	Leaves	Concoction	Oral	[111]
	Ogun, Oyo, Ekiti, Lagos		Body cream, Hair growth	Leaves	Dried powder mixed with oil	Topical	[166]
	Ogun		Toxic plant	Leaves	Unspecified	Oral/Contact	[165]
	Plateau		Mania/Depression	Leaves/Root	Infusion/Incense	Oral/Inhalation	[171]
	Ogun, Oyo		Wound	Leaves	Decoction	Oral/Topical	[131]
	Plateau		Pesticide	Leaves	Unspecified	Positioning/Spraying/Spreading/Smoking	[114]
	Oyo		Vector-borne diseases	Whole plant/Root	Pulverisation	Body bath	[187]
	Kebbi, Kwara		Hepatitis, Meningitis, Poliomyelitis	Leaves	Decoction	Oral	[57, 158]
	Kwara		Female sexual dysfunction, Female Infertility	Leaves	Unspecified	Unspecified	[84]
*Ocimum basilicum* L. (Lamiaceae)	Kwara, Lagos, Sokoto, Ogun	Dai dooyaata gidaa (H.), Efinrin ata/Efinrin (Yor.), Nchuanwu/alolo. Ahigbe (Ig.)	Cancer	Leaf/Root	Decoction/Smoking	Oral/Dermal absorption	[157, 172, 181]
	Ogun		Breast infection	Leaf	Decoction	Bathing	[132]
	Plateau		Aggression/Insomnia	Leaf/Stem-bark	Decoction/infusion	Oral	[171]
	Oyo, Ogun		Wound	Leaf	Maceration	Topical	[131]
	Kwara		Poliomyelitis, Meningitis, Hepatitis, Poliomyelitis	Stem-bark	Decoction	Oral	[158]
	Oyo		Dysentery, Loss of appetite, Sleeplessness	Leaf	Decoction	Unspecified	[58]
	Rivers		Skin disease, Stomachache, Dysentery, Laxative, Haemorrhoid	Leaf	Juice extraction/Infusion	Topical/Oral	[41]
	Ondo		Malaria	Leaf	Unspecified	Unspecified	[178]
	Katsina		Ease Labour	Whole plant	Maceration	Oral	[61]
*Ocimum gratissimum* L. (Lamiaceae)	Bayelsa	Daidoya/Daddoya ta gida (H.), Efinrin (Yor.), Nchuawun (Ig.)	Fracture	Leaves	Concoction	Topical	[60]
	Lagos, Osun		Cough	Unspecified	Unspecified	Unspecified	[15, 105]
	Ogun		Diabetes	Leaves	Maceration	Oral	[39]
	Kano, Lagos		Vomiting, Diarrhoea, Stomachache, Purgative,	Leaves	Unspecified	Unspecified	[10, 15]
	Ogun		Guinea worm infestation	Leaves	Unspecified	Unspecified	[159]
	Kwara, Southern Nigeria states		Malaria	Leaves	Maceration/Decoction/Juice extraction	Oral	[101, 129, 146, 167, 176],, [59, 178]
	Sokoto, Osun, Oyo, Ogun		Cancer	Whole plant/Leaves	Decoction	Oral	[160, 172, 181]
	Oyo		Vector-borne diseases	Leaves	Concoction	Oral	[187]
	Ondo		Pile, Diarrhoea, Dysentery, Respiratory tract infection	Leaves	Concoction	Oral	[17]
	Lagos		Diabetes	Leaves	Squeezing	Oral	[111]
	Lagos		Fever, Diarrhoea, Colds, Impotence, Dysentery, Rheumatism	Leaves	Infusion/Squeezing	Oral	[40]
	Oyo		Intestinal worm	Leaves	Unspecified	Unspecified	[14]
	Benue, Ogun		Cough, Tuberculosis	Leaves	Squeezing	Oral	[89, 169]
	Ogun		haemorrhoid, Pile, Typhoid, Diarrhoea	Leaves	Decoction/Infusion	Oral	[82]
	Ogun		Measles	Leaves	Unspecified	Unspecified	[186]
	Plateau		Animal diarrhoea	Leaves	Unspecified	Unspecified	[115]
	Rivers, Akwa ibom		Haemorrhoid, Typhoid, Stomachache, Cough	Leaves	Infusion/Soup	Oral/Enema	[41, 85]
*Olax subscorpioidea* Oliv. (Olacaceae)	Ogun, Osun, Oyo	Gwaanon kurmii (H.), Ifon (Yor.), Osaja/Igbulu (Ig.)	Cough, Asthma	Bark/Root	Pulverisation/Maceration	Oral	[96, 185]
	Oyo		Wound, Skin disorders	Root	Concoction	Body bath	[179]
	Kaduna		Convulsion in children, polio, jaundice, hepatitis, rheumatism, asthma, fever, cold	Unspecified	Unspecified	Unspecified	[163]
	Ogun		Breast infection	Root	Decoction	Body bath	[132]
	Niger		Mental illness	Whole plant	Unspecified	Unspecified	[104]
	Ogun, Oyo, Lagos, Ekiti		Cancer	Root/Bark	Decoction	Oral	[130, 181]
	Oyo		Intestinal worm	Root	Unspecified	Unspecified	[14]
	Oyo		Children scalp infections	Fruit	Powder	Body bath	[150]
	Oyo		Neurodegenerative disorders	Stem-bark	Unspecified	Unspecified	[184]
	Kwara		Female sexual dysfunction, Female Infertility	Root	Unspecified	Unspecified	[84]
	Oyo, Rivers		Jaundice, Yellow fever, Convulsion, Toothache	Leaves/Stem/Root	Decoction/Infusion/Mastication	Oral	[41, 58]
	Ondo, Oyo		Malaria	Leaves/Root	Decoction/Concoction	Oral	[58, 178]
	Ogun		Inflammation, Depression, Infectious diseases	Unspecified	Unspecified	Unspecified	[182]
	Ekiti, Ondo, Osun, Oyo		Poliomyelitis	Root	Unspecified	Unspecified	[57]
*Parkia biglobosa* (Jacq.) R.Br. ex G.Don syn. *Parkia clappertoniana* Keay (Fabaceae)	Ogun	Dorawa (H.), Iru/igba/Lasangba (Yor.), Ogiri (Ig.)	Diabetes	Leaves	Infusion	Oral	[39]
	Oyo		Wound	Bark	Decoction	Body bath	[179]
	Osun		Cough	Unspecified	Unspecified	Unspecified	[105]
	Borno		Yellow fever, Constipation	Fruit, Pulp	Maceration	Oral	[34]
	Borno		High blood pressure	Bark	Soaking	Oral	[34]
	Kebbi, Sokoto, Ogun		Cancer	Bark/Root	Decoction	Oral	[38, 172, 181]
	Ogun, Oyo, Osun, Kwara		Male contraceptive	Seeds	Unspecified	Ring	[42]
	Kaduna, Kano, Katsina, Jigawa		Arthritis	Stem-bark	Decoction	Unspecified	[180]
	Ondo		Fever, Respiratory tract infection	Seeds	Decoction	Oral	[17]
	Oyo		Abscess	Fruit	Decoction	Oral/Bathing	[150]
	Plateau		Pesticide	Fruit/Leaves/Seeds	Unspecified	Dressing/Positioning/Spraying/Smoking	[114]
	Katsina, Kebbi, Niger		Yellow fever, Poliomyelitis, Smallpox, Meningitis	Leaves/Stem-bark/Root	Decoction/Powder/Concoction	Oral	[158, 161]
	Benue		Constipation, Fevers	Bark	Concoction/Decoction	Oral	[169]
	Plateau		Animal diarrhoea	Stem-bark	Unspecified	Unspecified	[115]
	Katsina		Snake venom	Seeds	Unspecified	Unspecified	[91]
	Oyo		Tonic, Malaria, Intestinal disorders, Mental disorder, Condiment	Bark/Leaves/Seed/Fruit pulp	Concoction/Soup	Oral	[58]
	Ondo		Visual modality	Seeds	Unspecified	Unspecified	[97]
	Ondo		Malaria	Seeds/Stem-bark	Infusion	Unspecified	[178]
	Katsina		Stomachache	Bark	Powder	Oral	[61]
*Persea americana* Mill. (Lauraceae)	Osun	Fiya (H.), Pia (Yor.), Ubebekee/Ube Oyibo (Ig.)	Cough	Unspecified	Unspecified	Unspecified	[105]
	Lagos		Longevity	Unspecified	Unspecified	Unspecified	[15]
	Southern Nigeria states		Malaria	Stem-bark/Leaves	Decoction/Maceration/Juice extraction	Oral	[101, 102, 129, 167, 176],, [59, 178]
	Zamfara		Rheumatoid arthritis	Leaves	Unspecified	Unspecified	[16]
	Plateau		Pesticide	Seeds	Unspecified	Bait	[114]
	Osun, Oyo		Cancer	Fruit	Unspecified	Unspecified	[160]
	Kwara		Female sexual dysfunction, Female Infertility	Seeds	Unspecified	Unspecified	[84]
	Rivers, Akwa Ibom		Hypertension, Menstrual disorder	Fruit/Leaves/Seeds	Decoction/Infusion/Powder	Oral	[41, 85]
	Ekiti, Ondo, Osun, Oyo		Poliomyelitis, Chickenpox, Measles	Fruit	Unspecified	Unspecified	[57]
*Phyllanthus amarus* Schumach. & Thonn. (Phyllanthaceae)	Ogun	Geron tsuntsaye (H.), Eyin olobe (Yor.), Ngwu/Ite kwonwa-nazu (Ig.)	Diabetes	Whole plant	Decoction	Oral	[39]
	Bayelsa		Fracture	Whole plant	Poultice	Topical	[60]
	Lagos, Osun		Cough, Immune booster	Unspecified	Unspecified	Unspecified	[15, 105]
	Ondo		Skin spot, Dysentery, Wound	Leaves	Squeezing	Topical	[17]
	Ogun		Toxic plant	Leaves/Stem	Unspecified	Contact	[165]
	Kogi		Anti-poison, Abdominal pain	Leaves/Stem	Infusion/Powder	Oral	[162]
	Ogun, Oyo, Lagos, Ekiti		Cancer, Benign prostatic hyperplasia	Leaves	Decoction/Squeezing	Topical/Oral	[130, 133]
	Katsina, Kwara		Hepatitis, COVID-19	Leaves	Decoction	Oral	[158]
	Southern Nigeria states		Malaria	Leaves	Decoction/Infusion	Oral	[59, 85, 167, 178]
	Kwara		Female sexual dysfunction, Female Infertility	Leaves	Unspecified	Unspecified	[84]
	Oyo		Hives, Smallpox	Leaves	Powder	Cream	[58]
	Ondo		Kidney stones	Leaves	Unspecified	Unspecified	[97]
	Ogun		Liver problems, Diabetes, Fever	Unspecified	Unspecified	Unspecified	[182]
*Piliostigma thonningii* (Schumach.) Milne-Redh. (Fabaceae)	Kano, Plateau	Kalgo (H.), Abafe (Yor.), Okpoatu (Ig.)	Diarrhoea, Dysentery, Animal diarrhoea	Leaves	Unspecified	Unspecified	[10, 115]
	Lagos		Potency	Unspecified	Unspecified	Unspecified	[15]
	Ogun		Guinea worm infestation	Leaves/Root	Unspecified	Unspecified	[159]
	Oyo		Wound	Bark	Concoction	Body bath	[179]
	Ogun		Stomach pain	Stem-bark	Decoction	Oral	[132]
	Kebbi, Sokoto		Cancer	Bark/Root	Unspecified	Unspecified	[38, 172]
	Kwara, Oyo		Malaria, Diabetes	Stem-bark/Root/Bark/Leaves	Concoction	Unspecified	[58, 101]
	Niger		Backache, Dysentery, Cough, Piles	Leaves/Root	Concoction	Body bath/Oral/Steambath	[161]
	Katsina, Kebbi		Hepatitis, Poliomyelitis, Smallpox, Meningitis	Leaves/Stem	Decoction	Oral	[158]
	Benue, Niger		Cough, Tuberculosis, Respiratory disorders, Skin disease	Leaves/Bark/Twig	Decoction	Oral/Steam bath	[169, 173]
	Ogun		Jaundice, Yellow fever, Diabetes	Leaves/Seed	Decoction	Oral	[82]
*Piper guineense* Schumach. & Thonn. (Piperaceae)	Kwara, Lagos	Masoro (H.), Iyere (Yor.), Uziza (Ig.)	Breast cancer	Seeds	Powder	Topical	[157]
	Bayelsa		Inflammation	Fruit/Leaves	Poultice	Topical	[60]
	Ogun, Oyo		Guinea worm infestation	Seeds/Root	Unspecified	Unspecified	[14, 159]
	Kwara, Southern Nigeria states		Malaria	Fruit/Seeds	Infusion/Decoction	Oral	[59, 101, 178]
	Ondo, Osun		Respiratory tract infection, Inflammation, Cough	Fruit/Seeds	Concoction	Oral	[17, 105]
	Ogun		Toxic plant	Root	Unspecified	Oral	[165]
	Oyo, Ogun		Wound	Leaves	Decoction	Topical	[131]
	Katsina, Kebbi, Kwara		COVID-19, Poliomyelitis, Yellow fever, Monkeypox, Hepatitis, Lassa fever, Meningitis	Seeds/Stem-bark	Concoction/Decoction/Powder	Oral	[158]
	Oyo		Neurodegenerative disorders	Leaves/Fruit	Unspecified	Unspecified	[184]
	Oyo		Memory enhancer, Problematic delivery, Low sperm count, Weak erection	Seeds	Concoction	Oral	[183]
	Katsina		Womb cleanser	Seed/Leaf	Unspecified	Unspecified	[91]
	Ondo		Hypertension	Fruit	Unspecified	Unspecified	[97]
	Ekiti, Ondo, Osun, Oyo, Ogun		Chickenpox, Measles	Leaves/Seed	Unspecified	Unspecified	[57, 186]
*Prosopis africana* (Guill. & Perr.) Taub. (Fabaceae)	Kaduna	Kiryia (H.), Àyàn (Yor.), Okpei (Ig.)	Skin diseases, Caries, Fever, Eye wash, Gonorrhoea, Toothache, Astrigent	Unspecified	Decoction/Mastication	Oral/Wash	[163]
	Kebbi		Cancer	Bark/Root	Powder	Oral/Topical	[38]
	Kaduna, Kano, Katsina, Jigawa		Arthritis	Root	Decoction	Unspecified	[180]
	Ogun		Toxic plant	Seeds	Unspecified	Oral	[165]
	Yobe, Ogun, Plateau		Diarrhoea, Animal diarrhoea	Stem/Stem-bark	Powder/Decoction	Oral	[11, 82, 115]
	Niger		Toothache	Leaves/Stem	Decoction/Mastication	Oral	[161]
	Katsina, Kebbi		Yellow fever	Leaves	Decoction	Oral	[158]
	Benue		Stimulant	Bark/Root	Maceration	Oral	[169]
	Anambra		Malaria	Leaves/Stem-bark	Decoction	Oral	[129],
	Katsina		Fever	Bark	Maceration	Oral	[61]
*Pseudocedrela kotschyi* Harms (Meliaceae)	Oyo	Emigbegiri (Yor.)	COVID-19	Leaves	Decoction	Oral	[96]
	Oyo		Wound, Skin disorders	Stem-bark	Decoction	Oral	[179]
	Kwara		Malaria	Stem-bark	Unspecified	Unspecified	[101]
	Oyo, Ogun, Lagos, Ekiti		Body cream	Bark	Dried powder mixed with oil	Dermal	[166]
	Ogun		Children scalp infections	Bark	Decoction	Oral/Body bath	[150]
	Ogun, Osun, Oyo, Borno		Cancer	Bark/Stem-bark	Decoction	Oral	[64, 160, 181]
	Oyo		Vector-borne diseases	Bark	Concoction	Oral	[187]
	Sokoto		Diabetes	Root	Decoction	Oral	[103]
	Oyo		Skin diseases, Dysentery	Bark/Root	Mastication/Concoction	Unspecified	[58]
	Ekiti, Ondo, Osun, Oyo		Hepatitis	Stem-bark	Unspecified	Unspecified	[57]
*Psidium guajava* L. (Myrtaceae)	Oyo, Osun	Gwaiba (H.), Girofa (Yor.)	Cough	Leaves/Bark	Decoction	Oral	[96, 105]
	Katsina		Dysentery	Leaves	Unspecified	Unspecified	[10]
	Kaduna		Malaria, Enteric bacteria, Antimicrobial, Antioxidant	Unspecified	Unspecified	Unspecified	[163]
	Lagos		Stomachache, Malaria, Insanity	Unspecified	Unspecified	Unspecified	[15]
	Lagos, Kwara, Kebbi, Sokoto		Cancer	Leaves/Bark/Root	Powder/Maceration/Decoction	Topical/Oral	[38, 157, 172]
	Bayeisa		Wound, Bone hardening	Leaves	Concoction	Oral/Body bath	[60]
	Borno, Kwara, Adamawa, Southern Nigeria states		Malaria	Leaves/Bark/Stem-bark/Immature stem/Fruit	Decoction/Direct	Steam-bath/Oral	[34, 63, 101, 102, 129, 146, 167, 178],, [59, 176]
	Ogun		Sedative	Leaves	Unspecified	Unspecified	[90]
	Ondo, Ogun		Respiratory tract infection, Tuberculosis	Fruit/Leaves/Bark	Cooking/Squeezing/Tincture	Oral	[17, 89]
	Yobe, Sokoto, Benue, Plateau		Diarrhoea, Dysentery, Pile, Animal diarrhoea	Leaves	Powder	Oral	[11, 62, 115, 169]
	Lagos		Obesity	Unspecified	Decoction	Oral	[40]
	Kebbi, Kwara, Sokoto		Meningitis, COVID-19, Lassa fever	Leaves/Stem-bark	Decoction	Oral	[158]
	Katsina		Reduce frigidity, Allergy	Fruit	Unspecified	Unspecified	[91]
	Rivers		Malaria, Typhoid, Stomachache, Laxative, Infertility, Convulsion, Cough, Diarrhoea	Leaves	Decoction/Infusion	Oral	[41]
	Ogun		Diarrhoea, Dysentery, Gastroenteritis, Fever, Cough, Pain	Unspecified	Unspecified	Unspecified	[182]
	Ekiti, Ondo, Osun, Oyo		Jaundice	Stem-bark	Unspecified	Unspecified	[57]
	Oyo		Low sperm count, Malaria	Leaves	Squeezing	Oral	[83]
	Katsina		Fever	Leaves	Decoction	Oral	[61]
*Rauvolfia vomitoria* Wennberg syn*. Rauvolfia vomitoria* Afzel (Apocynaceae)	Ogun	Kukumaka (H.), Asofeyeje (Yor.), Akata (Ig.)	Antiperiodic problem, Premenstrual dysphoric disorder	Root	Grinding	Oral	[132]
	Lagos		Hypertension, Muscle relaxant, Cough	Unspecified	Unspecified	Unspecified	[15]
	Oyo		Wound	Root	Decoction	Oral/Body bath	[179]
	Ogun, Oyo		Guinea worm infestation, Intestinal worm	Leaves	Unspecified	Unspecified	[14, 159]
	Lagos		Diabetes	Leaves/Root	Concoction/Squeezing	Oral	[111]
	Delta		Skin disease, Smallpox	Leaves/Root	Decoction/Infusion	Unspecified	[143]
	Ogun		Stomach upset, Vomiting	Root/Seeds	Unspecified	Oral	[165]
	Lagos		Asthma, Diarrhoea, Rheumatism, Jaundice	Bark	Infusion	Oral	[40]
	Ogun, Lagos, Anambra, Southern Nigeria states		Malaria	Leaves/Root/Stem-bark/Sap	Decoction	Oral	[129, 146, 167],, [59]
	Ogun		Haemorrhoids, Pile	Unspecified	Decoction	Oral	[82]
	Osun, Oyo, Ogun		Cancer	Root/Leaves	Concoction/Powder	Oral	[37, 160, 181]
	Oyo		Neurodegenerative disorders	Root	Unspecified	Unspecified	[184]
	Kwara		Female sexual dysfunction, Female Infertility	Root	Unspecified	Unspecified	[84]
	Rivers		Mental illness, Aphrodisiac, Skin disease	Root/Leaves	Infusion/Juice	Oral/Bathing	[41]
	Southern Nigeria states		Yellow fever, Internal pain, Gastroenteritis, Constipation, Mental disorder, Hypertension, Malaria, Abortifacient, Purgative	Leaves/Bark/Root	Unspecified	Unspecified	[177]
	Akwa Ibom, Ogun		Mental illness/disorders, Insomnia	Root	Decoction	Oral	[85, 168, 182]
*Saccharum officinarum* L. (Poaceae)	Kwara, Lagos, Ogun	Rakke (H.), Ireke (Yor.), Okpete (Ig.)	Cancer (Prostate/Cervical)	Stem-juice	Decoction	Oral	[82, 157]
	Oyo, Osun		Cough	Stem	Pounding/Infusion	Oral	[96, 105]
	Ogun		Tonic	Stem	Unspecified	Unspecified	[90]
	Ondo, Akwa ibom		Malaria	Leaves/Fruit/Stem	Squeezing/Decoction	Oral	[17, 85, 178]
	Lagos		Diabetes	Root/Leaves	Squeezing	Oral	[111]
	Lagos		Urinary tract infection	Stem	Infusion/Mastication	Oral	[40]
	Kwara		Yellow fever, Poliomyelitis	Whole plant	Raw	Oral	[158]
	Ogun, Oyo, Osun		Asthma	Stem	Mastication/Juice extraction	Oral	[185]
*Schwenckia americana* L. (Solanaceae)	Sokoto	Dandana (H.), Igbale odan/Ewe-dandan (Yor.)	Cancer	Whole plant	Maceration	Oral	[172]
	Ogun		Toxic plant	Leaves	Unspecified	Oral	[165]
	Sokoto		Diabetes	Leaves/Root/Bark	Maceration/Decoction	Oral	[103]
	Niger		Tuberculosis, Respiratory disorders	Whole plant	Unspecified	Unspecified	[173]
	Katsina, Plateau		Diarrhoea, Animal diarrhoea	Whole plant	Decoction	Oral	[61, 115]
*Securidaca longipedunculata *Fresen. (Polygalaceae)	Oyo, Ogun, Osun	Uwar magunguna/Sanya (H.), Ipeta (Yor.), Ezeogwu (Ig.)	Cough, Asthma	Bark/Root	Pulverisation/Maceration	Oral	[96, 185]
	Ogun, Oyo		Guinea worm infestation, Intestinal worm	Leaves/Root	Unspecified	Unspecified	[14, 159]
	Oyo, Ogun		Wound	Bark/Root/Stem-bark	Decoction/Powder	Body bath/Oral	[131, 179]
	Kebbi, Ogun, Oyo, Lagos, Ekiti, Borno		Cancer	Bark/Leaves/Root/Stem-bark	Decoction/Concoction	Oral/Topical/Body bath	[37, 38, 64, 130, 181]
	Lagos, Sokoto		Diabetes	Root/Leaves	Pulverisation/Maceration	Oral	[103, 111]
	Sokoto		Diarrhoea	Bark	Unspecified	Unspecified	[62]
	Niger, Plateau		Mental illness, Psychosis	Root/Leaves	Decoction/Infusion/Incense	Oral/Body bath/Inhalation	[104, 171]
	Oyo		Abscess	Root	Decoction	Oral/Body bath	[150]
	Ekiti, Ondo, Osun, Oyo		Jaundice	Stem-bark	Unspecified	Unspecified	[57]
	Kebbi, Kwara, Sokoto		Hepatitis, Meningitis, Poliomyelitis	Leaves/Stem	Decoction	Oral/Topical	[158]
	Plateau		Erectile dysfunction	Root	Decoction	Oral	[98]
	Anambra, Southern Nigeria states		Malaria	Stem-bark/Root	Decoction/Powder	Oral	[129],, [59]
	Oyo		Venereal diseases, Children’s skin diseases	Root	Decoction	Unspecified	[58]
	Katsina		General well-being	Bark	Decoction	Oral	[61]
*Senna alata* (L.) Roxb. Syn. *Cassia alata* L. (Fabaceae)	Ogun	Rai dore (H.), Asunwon oyinbo (Yor.), Ogala/Omirima (Ig.)	Diabetes	Leaves	Maceration	Oral	[39]
	Ogun		Guinea worm infestation	Leaves/Root	Unspecified	Unspecified	[159]
	Ogun		Blackish period blood	Flower	Pulverisation	Oral	[132]
	Oyo		COVID-19	Leaves	Decoction	Oral	[96]
	Ondo		Cramp, Ringworm, Scabies, Eczema, Craw-craw	Leaves	Boiling	Topical	[17]
	Oyo		Purgative, Pile, Fever, Gonorrhoea	Leaves/Flower bud	Powder/Concoction	Oral	[175]
	Ogun, Oyo, Lagos, Ekiti		Body cream	Leaves/Seed	Dried powder mixed with oil	Topical	[166]
	Delta		Eczema, Ringworm, Abscess, Skin disease, Bleeding	Leaves/Root	Infusion/Juice extraction/Poultice	Unspecified	[143]
	Kogi		General body vitality, Stomach upset, Pile	Leaves/Flower	Powder/Decoction	Oral	[162]
	Lagos		Fever, Stomach ulcer, Stomach pain	Leaves	Decoction	Oral	[40]
	Ogun		Skin infections	Unspecified	Infusion	Topical	[82]
	Borno, Osun, Oyo, Ogun		Cancer	Flower/Leaves/Whole plant	Powder	Oral	[64, 160, 181]
	Oyo		Benign prostatic hyperplasia	Leaves	Tincture	Oral	[133]
	Kwara		Female sexual dysfunction, Female Infertility	Leaves/Flower	Unspecified	Unspecified	[84]
	Ondo		Malaria	Leaves	Decoction	Unspecified	[178]
*Senna occidentalis* (L.) Link syn. *Cassia occidentalis* L. (Fabaceae)	Kaduna	Sanga-Sanga/Tafasar masar (H.), Abo-rere (Yor.), Sigbunmuo/Akidi-agbara (Ig.)	Constipation, Respiratory tract disorder, Wound, Improving digestion	Unspecified	Unspecified	Unspecified	[163]
	Kwara, Katsina		Malaria, Fever	Leaves	Decoction	Steam bath	[61, 101]
	Sokoto		Cancer	Leaves	Decoction	Oral	[172]
	Kaduna, Kano, Katsina, Jigawa		Arthritis	Leaves	Infusion	Unspecified	[180]
	Oyo		Constipation, Eczema, Skin infections	Root/Leaves	Concoction/Squeezing	Oral/Topical	[175]
	Delta		Abscess, Skin diseases, Inflammation, Bleeding	Leaves/Root	Infusion/Poultice	Unspecified	[143]
	Kebbi		Hepatitis, Meningitis, COVID-19, Yellow fever, Poliomyelitis	Whole plant	Decoction	Oral	[158]
	Benue		Stomachache	Leaves/Bark	Maceration	Oral	[169]
	Ogun, Ekiti, Ondo, Osun, Oyo		Measles	Leaves	Unspecified	Unspecified	[57, 186]
	Sokoto		Diabetes	Leaves/Root	Infusion/Decoction	Oral	[103]
*Sida acuta *Burm.f. (Malvaceae)	Ogun	Kalkashin kwado (H.), Isekotu/Osepotu (Yor.), Udo (Ig.)	Guinea worm infestation	Leaves	Unspecified	Unspecified	[159]
	Kwara, Ondo, Southern Nigeria states, Oyo		Malaria	Leaves/Whole plant/Aerial part	Decoction	Oral/Body bath	[59, 101, 176, 178]
	Ondo, Akwa Ibom		Whitlow, Liver, Dysentery, Cholera	Leaves/Root	Squeezing/Decoction/Paste	Topical/Oral	[17, 85]
	Ogun		Toxic plant	Leaves	Unspecified	Oral	[165]
	Ogun		Gonorrhoea, Syphilis	Leaves	Infusion	Oral	[82]
	Oyo		Benign prostatic hyperplasia	Leaves	Decoction	Oral	[133]
	Ondo		Ulcer	Whole plant	Unspecified	Unspecified	[97]
	Rivers		Stomachache, Dysentery, Convulsion	Leaves/Root	Decoction/Infusion	Oral	[41]
	Ogun		Fever, Pain, Microbial infections	Unspecified	Unspecified	Unspecified	[182]
	Lagos		Fibroids	Unspecified	Unspecified	Unspecified	[15]
*Sorghum bicolor* (L.) Moench (Poaceae)	Kwara, Ondo, Lagos, Southern Nigeria states	Dawa/Jero (H.), Oka baba (Yor.), Soro (Ig.)	Malaria	Leaves/Root/Grain	Decoction	Unspecified	[15, 17, 59, 101, 178]
	Ogun, Lagos, Ondo		Stomach pain, Deworming, Diarrhoea	Grains/Leaves	Decoction/Concoction	Oral	[15, 17, 132]
	Osun		Cough	Unspecified	Unspecified	Unspecified	[105]
	Ogun, Oyo, Ondo		Tonic, Anemia, Blood tonic	Leaves/Stem	Concoction	Unspecified	[58, 90, 97]
	Ekiti, Ondo, Osun, Oyo		Chickenpox	Leaves	Unspecified	Unspecified	[57]
*Sphenocentrum jollyanum* Pierre (Menispermaceae)	Kwara, Lagos, Oyo, Ogun, Southern Nigeria states	Akerejupon/Ajo (Yor.), Ezeogwu (Ig.)	Malaria	Root/Leaves/Fruit	Decoction	Oral	[58, 59, 101, 146, 168, 176, 178]
	Oyo, Ekiti, Ondo, Osun, Ogun		Yellow fever, Jaundice, Typhoid	Root/Fruit	Concoction	Oral	[57, 58, 168]
	Ondo		Pile	Root	Unspecified	Unspecified	[97]
	Ogun		Wounds, Fever, Coughs, High blood pressure, Breast tumour, Constipation	Unspecified	Unspecified	Unspecified	[182]
	Lagos		Dewormer	Unspecified	Unspecified	Unspecified	[15]
*Spondias mombin* L. (Anacardiaceae)	Oyo, Osun	Tsardar masar/Isada (H.), Iyeye (Yor.), Ijikara/Ngulungwu (Ig.)	Cough	Bark	Decoction	Oral	[96, 105]
	Ogun		Diabetes	Leaves	Decoction	Oral	[39]
	Bayelsa		Pain relief, Antiseptic	Bark/Root	Decoction	Washing	[60]
	Lagos		Insomnia, Diabetes, Anti-snakebite	Unspecified	Unspecified	Unspecified	[15]
	Ogun		Postpartum haemorrhoid, Placenta evacuation	Stem-bark/Leaves	Decoction	Oral	[97, 132]
	Ogun, Oyo		Guinea worm infestation, Intestinal worm	Bark/Leaves	Unspecified	Unspecified	[14, 159]
	Kaduna		Malignant tumour, Gonorrhoea, Conjunctivitis, Sour throat, Bronchitis	Unspecified	Unspecified	Unspecified	[163]
	Ondo, Rivers, Akwa ibom		Dysentery, haemorrhoid, Pile	Leaves/Fruit/Root-bark	Cooking/Decoction/Squeezing/Powder	Oral	[17, 41, 83, 85]
	Ogun, Oyo		Wound	Leaves	Decoction	Oral/Cleansing	[131]
	Southern Nigeria states		Eye problem, Fever, Yaw, Diuretic, Gastroenteritis, Dysentery, Diarrhoea	Leaves/Flower/Gum/Fruit	Decoction	Oral	[177]
	Kwara, Ondo, Ogun, Southern Nigeria states		Yellow fever, Malaria	Stem-bark/Leaves	Decoction	Oral	[17, 59, 158, 167]
	Benue		Hypertension	Leaves/Bark	Fermentation	Oral	[169]
	Oyo, Osun, Ogun		Cancer, Prostate enlargement	Leaves/Bark	Decoction	Oral	[83, 160, 181]
	Oyo		Neurodegenerative disorders	Leaves	Unspecified	Unspecified	[184]
	Ogun, Rivers, Niger		Tuberculosis, Cough, Respiratory disorders	Leaves/Stem-bark	Maceration/Decoction	Oral	[41, 89, 173]
	Rivers		Gonorrhoea, Toothache, Aphrodisiac, Fibroid	Leaves/Bark	Decoction/Juice	Oral	[41]
	Ekiti, Ondo, Osun, Oyo		Chickenpox, Jaundice	Stem-bark	Unspecified	Unspecified	[57]
	Oyo, Kaduna		Fibroid	Fruit/Leaves	Decoction	Oral	[83, 163]
*Syzygium aromaticum* (L.) Merr. & L.M.Perry syn. *Eugenia aromatica* (L.) Baill. (Myrtaceae)	Oyo	Kanumfarii (H.), Kanafuru (Yor.), Kloovu/Kanafure (Ig.)	Cough, Flu	Seeds	Infusion/Pulverisation	Oral	[96]
	Ondo		Respiratory tract infection, Inflammation	Cloves	Concoction	Oral	[17]
	Oyo		Intestinal worm	Flower (dried)	Unspecified	Unspecified	[14]
	Oyo		Children infections	Fruits	Infusion	Oral	[150]
	Oyo, Ogun		Wound	Flower	Concoction	Oral/Topical	[131]
	Katsina, Kebbi, Kwara, Sokoto		Hepatitis, COVID-19, Yellow fever	Seeds/Fruit	Decoction	Oral	[158]
	Ogun, Oyo, Osun		Asthma	Flower	Maceration	Oral	[185]
	Kwara		Female sexual dysfunction, Female Infertility	Seeds	Unspecified	Unspecified	[84]
	Borno		Cancer	Root	Unspecified	Unspecified	[64]
	Oyo		Dysentery, Diarrhoea, Condiment, Preservatives	Seeds	Decoction/Soup	Oral	[58]
*Talinum fruticosum* (L.) Juss. syn. *Talinum triangulare* (Jacq.) Willd. Syn. (Talinaceae)	Lagos	Alenyruwai (H.), Gbure (Yor.), Mgbolodi (Ig.)	Blood tonic, Malaria	Unspecified	Unspecified	Unspecified	[15]
	Osun		Cough	Unspecified	Unspecified	Unspecified	[105]
	Delta, Ogun		Boil	Leaves	Juice extract/Paste	Topical	[82, 143]
	Ogun		Toxic plant	Root	Unspecified	Oral	[165]
	Oyo		Neurodegenerative disorders	Leaves	Unspecified	Unspecified	[184]
	Oyo		Microbial infection	Leaves	Unspecified	Unspecified	[183]
	Sokoto		Diabetes	Root/Bark	Decoction	Oral	[103]
	Rivers		Inflammation, Laxative	Whole plant	Poultice/Decoction	Topical/Oral	[41]
	Ondo, Rivers		Malaria	Aerial part	Decoction	Oral	[41, 178]
	Ogun		Diabetes, Cancer, Stroke, Obesity, Measles, Anemia, Ulcers, High blood pressure	Unspecified	Unspecified	Unspecified	[182]
	Delta, Southern Nigeria states		Measles	Leaves	Decoction	Oral	[57, 143]
*Terminalia avicennioides* Guill. & Perr. (Combretaceae)	Ogun	Baushe (H.), Idi (Yor.), Ebo (Ig.)	Breast infection	Stem-bark	Decoction	Bathing	[132]
	Kano		Diarrhoea	Leaf/Bark	Unspecified	Unspecified	[10]
	Kaduna		Wounds, Skin infections, Leprosy, Impetigo, Athletic foot, Burns, Bruises, Toothache	Unspecified	Unspecified	Unspecified	[163]
	Kaduna, Kano, Katsina, Jigawa		Arthritis	Stem-bark/Leaf	Decoction	Unspecified	[180]
	Katsina, Kebbi, Sokoto		Poliomyelitis, Meningitis, Monkey pox, Yellow fever	Stem-bark/Bark	Decoction	Oral	[158]
	Ogun		Skin irritation	Bark	Unspecified	Oral	[168]
	Plateau		Animal diarrhoea	Unspecified	Unspecified	Unspecified	[115]
	Niger		Tuberculosis, Respiratory disorders	Mistletoes/Root-bark/Fruit	Unspecified	Unspecified	[173]
	Ogun		Cancer	Bark	Decoction	Oral	[181]
	Southern Nigeria states		Malaria	Leaf/Stem-bark	Decoction	Unspecified	[59]
*Tetrapleura tetraptera* (Schum. and Thonn.) Taub. (Fabaceae)	Oyo	Dawo (H.), Aridan (Yor.), Oshosho (Ig.)	COVID-19	Fruit	Decoction	Oral	[96]
	Ogun		Guinea worm infestation	Seed/Bark/Root/Leaves	Unspecified	Unspecified	[159]
	Oyo, Osun		Cough	Fruit	Pulverisation/Grating/Infusion	Oral	[96, 105]
	Oyo		Wound	Bark	Decoction	Oral/Topical	[179]
	Oyo		Flu	Fruit	Decoction/Infusion/Pulverisation	Oral	[96]
	Ogun		Breast infection	Seeds	Decoction	Bathing	[132]
	Kwara, Oyo		Malaria	Fruit	Powder	Oral	[58, 101]
	Ogun, Oyo, Osun, Kwara		Male contraceptive	Fruit	Decoction	Oral	[42]
	Lagos		Diabetes	Fruit	Decoction	Oral	[111]
	Oyo		Vector-borne diseases	Leaves	Concoction	Oral	[187]
	Oyo, Ogun, Ekiti, Lagos		Body cream	Fruit	Dried powder mixed with oil	Dermal	[166]
	Oyo		Children scalp infections	Fruit	Decoction/Powder	Oral/Bathing	[150]
	Osun, Oyo, Ogun		Cancer	Fruit/Bark	Powder/Decoction	Body wash/Oral	[160, 181]
	Ogun		Convulsion	Fruit	Unspecified	Oral	[168]
	Ogun, Oyo, Osun		Asthma	Fruit	Maceration/Concoction	Oral	[185]
	Kwara		Female sexual dysfunction, Female Infertility	Fruit/Leaves	Unspecified	Unspecified	[84]
	Oyo, Ekiti, Ondo, Osun		Skin disease	Bark/Fruit	Powder/Decoction	Body bath	[57, 58]
	Akwa Ibom		Conjunctivitis	Fruit	Oil extraction	Topical	[85]
	Kwara, Ekiti, Ondo, Osun, Oyo		Hepatitis, Poliomyelitis, Smallpox	Fruit/Seeds	Concoction	Oral	[57, 158]
*Uvaria chamae* P. Beauv. (Annonaceae)	Oyo	Kaskaifi (H.), Eruju/Akisan (Yor.), Afuru-agu/Mmimi ohia (Ig.)	COVID-19	Bark	Decoction	Oral	[96]
	Ogun, Oyo		Guinea worm infestation, Intestinal worm	Leaves/Root	Unspecified	Unspecified	[14, 159]
	Ogun, Oyo, Delta		Wound, Boil, Sore, Bleeding	Root/Stem-bark/Leaves	Decoction/Powder/Juice extraction	Oral/Body bath/Topical	[41, 85, 131, 143, 164, 179]
	Lagos		Diabetes	Stem	Decoction	Oral	[111]
	Plateau		Aggression/Psychosis	Leaves/Root	Infusion	Oral/Steam bath	[171]
	Plateau		Pesticide	Seeds	Unspecified	Topical	[114]
	Southern Nigeria states		Diarrhoea, Catarrh, Menorrhagia, Epistaxis, Hematuria, Haemolysis, Piles, Gastroenteritis	Leaves/Stem/Root-bark/Sap	Decoction/Infusion	Oral/Topical	[85, 164]
	Oyo, Osun, Ogun		Cancer, Benign prostatic hyperplasia	Root/Stem	Decoction	Oral	[133, 160, 181]
	Southern Nigeria states		Malaria	Leaves/Stem-bark/Seeds/Root	Decoction	Oral	[129],, [41, 59, 168]
	Ogun, Oyo, Osun		Asthma	Root	Maceration	Oral	[185]
	Rivers, Akwa Ibom		Haemorrhoid, Jaundice	Root	Decoction/Maceration	Oral	[41, 85]
*Vachellia nilotica *(L.) P.J.H.Hurter & Mabb syn. *Acacia nilotica* (L.) Willd. ex Delile (Fabaceae)	Kano, Sokoto, Yobe	Gabaruwa (H.), Igi Kasia/Booni (Yor.)	Constipation, Diarrhoea, Dysentery, Stomachache	Leaves/Root/Bark/Stem	Powder	Oral	[10, 11, 62]
	Borno		Rheumatism	Leaves	Squeezing	Topical	[34]
	Kebbi, Sokoto, Borno, Ogun		Cancer	Leaves/Bark/Root/Seeds/Stem	Powder/Maceration/Decoction	Oral/Topical	[38, 64, 172, 181]
	Kwara, Southern Nigeria states		Malaria	Stem-bark/Leaves/Seeds	Decoction	Unspecified	[59, 101]
	Ogun, Oyo, Lagos, Ekiti		Body cream	Flower/Bark	Dried powder mixed with oil	Topical	[166]
	Kano		Pediatric malaria	Leaves/Seeds	Unspecified	Unspecified	[35]
	Oyo		Intestinal worm	Seeds	Unspecified	Unspecified	[14]
	Oyo		Children scalp infections	Fruit	Decoction	Oral/bathing	[150]
	Kebbi, Sokoto		Hepatitis, Monkey pox, Meningitis, Smallpox, Poliomyelitis, COVID-19, Yellow fever	Leaves/Stem-bark	Decoction	Oral	[158]
	Ogun, Oyo, Osun		Asthma	Fruit	Maceration	Oral	[185]
	Sokoto		Diabetes	Root	Decoction	Oral	[103]
	Kwara		Female sexual dysfunction, Female Infertility	Seed	Unspecified	Unspecified	[84]
	Oyo		Fungal fumigant	Bark	Fumigation	Unspecified	[58]
	Katsina		Postpartum, Wound healing	Pod	Decoction	Topical	[61]
*Vernonia amygdalina* Delile (Asteraceae)	Kano, Borno	Shuwaka (H), Ewuro (Yor.), Onugbu (Ig.)	Typhoid, Stomachache, Diarrhoea, Stomach pain	Stem/Leaves	Pounding	Oral	[10, 34]
	Ogun, Lagos, Rivers		Diabetes	Leaves	Decoction/Infusion/Squeezing	Oral	[39–41, 82, 111]
	Kwara, Lagos, Borno, Ogun, Oyo, Ekiti		Cancer	Leaves/Root	Decoction	Oral	[64, 130, 157]
	Bayelsa, Zamfara, Katsina		Fracture, Rheumatoid arthritis	Leaves	Poultice	Topical	[16, 60, 91]
	Lagos		Asthma, Diarrhoea, Oral hygiene	Unspecified	Unspecified	Unspecified	[15]
	Osun		Cough	Unspecified	Unspecified	Unspecified	[105]
	Oyo		Vector-borne diseases	Leaves	Powder	Oral	[187]
	Ogun, Oyo		Guinea worm infestation, Intestinal worm	Leaves	Unspecified	Unspecified	[14, 159]
	Kwara, Southern Nigeria states		Malaria	Leaves/Root	Infusion/Maceration/Decoction	Oral	[40, 97, 101, 129, 178],, [41, 59, 82, 146, 167, 176]
	Ondo		Respiratory tract infection, Pains, Ringworm, Rashes, Eczema, Smallpox, Measles	Leaves	Concoction	Oral/Topical	[17]
	Oyo, Ogun, Ekiti, Lagos		Body cream	Leaves	Dried powder mixed with oil	Topical	[166]
	Delta, Ekiti, Ondo, Ogun, Osun, Oyo		Measles, Smallpox, Chickenpox, Jaundice	Leaves/Leaf sap/Root epidermis	Decoction/Tincture/Maceration/Poultice	Unspecified	[57, 143, 186]
	Ogun		Toxic plant	Root	Unspecified	Oral	[165]
	Plateau		Hallucinations	Leaves	Decoction	Oral	[171]
	Ogun, Oyo		Wound	Leaves	Decoction	Topical	[131]
	Kebbi, Kwara, Sokoto		Yellow fever, Smallpox, Lassa fever, COVID-19, Meningitis, Monkey pox	Leaves	Decoction	Oral	[158]
	Ogun		Hypertension	Leaves	Infusion/Decoction	Oral	[82]
	Oyo		Benign prostatic hyperplasia	Leaves	Squeezing	Oral	[133]
	Kwara		Female sexual dysfunction, Female Infertility	Leaves	Unspecified	Unspecified	[84]
	Rivers		Typhoid, Laxative, Rheumatism	Leaves/Bark	Decoction	Oral	[41]
	Niger		Tuberculosis, Respiratory disorders	Leaves	Unspecified	Unspecified	[173]
	Ogun		Fever, Inflammation, Infectious diseases	Unspecified	Unspecified	Unspecified	[182]
	Akwa ibom		Diabetes, Itching conditions	Leaves	Decoction/Soup/Pulverisation	Oral/Topical	[85]
	Katsina		Breast milk enhancement	Leaves	Powder	Oral	[61]
*Vitellaria paradoxa* C.F Gaertn (Sapotaceae)	Oyo	Dan ka’raye/Kade (H.), Emiyemi/Ori (Yor.), Okwuma (Ig.)	COVID-19	Seeds	Heating	Nasal	[96]
	Oyo, Osun		Cough, Flu	Seeds	Frying	Nasal/Topical	[96, 105]
	Oyo		Wound	Bark	Powder	Topical	[179]
	Kebbi, Sokoto, Ogun, Borno		Cancer	Bark/Oil/Leaves/Stem-bark/Butter	Powder/Paste	Oral/Topical	[38, 64, 172, 181]
	Borno		Swollen pain, Toothache	Fruit/Bark	Heating/Pounding	Topical	[34]
	Kwara, Ondo		Malaria	Root/Leaves/Stem-bark	Decoction	Unspecified	[101, 178]
	Zamfara, Niger		Rheumatoid arthritis, Luxation, Body pain	Fruit/Seeds	Cream	Topical	[16, 161]
	Ondo, Ogun, Niger		Respiratory tract infection/disorders, Tuberculosis, Nasal congestion	Leaves/Bark/Oil/Seeds	Decoction	Oral	[17, 89, 97, 173]
	Oyo, Ogun, Ekiti, Lagos		Moisturizer	Fat	Mixed with honey	Dermal	[166]
	Kano, Ogun		Chicken pox, Measles	Stem-bark/Fruit	Unspecified	Unspecified	[35, 186]
	Oyo		Children scalp infections	Bark/Fruit	Decoction/Powder	Oral/Bathing	[150]
	Plateau		Pesticide	Seeds	Unspecified	Smoking	[114]
	Kwara, Kebbi		Poliomyelitis, Yellow fever, COVID-19, Meningitis, Small pox, Monkey pox, Hepatitis	Stem-bark/Oil	Decoction/Ointment	Oral/Topical	[158]
	Oyo		Vector-borne diseases	Leaves	Cream	Topical	[187]
	Oyo		Microbial infection, Coolant	Leaves	Unspecified	Unspecified	[183]
	Ondo		Medicine, Fuelwood	Unspecified	Unspecified	Unspecified	[170]
	Kwara		Female sexual dysfunction, Female Infertility	Shell/Nut	Unspecified	Unspecified	[84]
	Oyo		Vitamin, Ointment, Malaria, Hypertension	Seeds	Oil extraction	Unspecified	[58]
	Katsina, Plateau, Niger		Stomachache, Dysentery, Animal diarrhoea	Bark/Stem-bark	Maceration/Concoction	Oral	[61, 115, 161]
*Vitex doniana* sweet (Lamiaceae)	Kebbi	Dunya (H.), Oori-nla (Yor.), Uchakoro/Uchakiri (Ig.)	Cancer	Bark/Leaves	Unspecified	Unspecified	[38]
	Lagos		Hypertension, Indigestion, Wound	Unspecified	Unspecified	Unspecified	[15]
	Ogun		Guinea worm infestation	Leaves	Unspecified	Unspecified	[159]
	Borno		Body weakness	Leaves/Bark	Maceration	Oral	[34]
	Kano		Ringworm	Leaves	Unspecified	Unspecified	[35]
	Niger		Stomachache	Leaves/Bark	Decoction	Oral	[161]
	Plateau		Psychosis/Anxiety	Leaves	Decoction	Oral	[171]
	Kebbi		Monkey pox, COVID-19, Smallpox, Poliomyelitis	Leaves	Decoction	Oral	[158]
	Ogun, Osun, Oyo, Niger		Asthma, Tuberculosis, Respiratory disorders	Bark/Stem-bark	Decoction	Oral	[173, 185]
	Plateau, Rivers		Animal diarrhoea, Diarrhoea, Dysentery	Leaves/Root/Seed/Fruit	Maceration/Mastication	Oral	[115] , [41]
	Anambra		Malaria	Leaves	Decoction	Oral	[129],
	Sokoto		Diabetes	Bark	Decoction	Oral	[103]
*Xylopia aethiopica *(Dunal) A.Rich. (Annonaceae)	Oyo	À góógè/Chimba (H.), Eeru alamo (Yor.), Uda (Ig.)	Stomachache, Dysentery, Wound, Bronchitis, Ulcers, Fever, Debility, Rheumatism	Seeds	Pulverisation/Infusion	Oral	[96]
	Ogun		Breast infection	Stem-bark	Decoction	Body bath	[132]
	Ogun		Vector-borne diseases	Fruit/Seeds	Powder	Oral	[187]
	Kwara, Lagos,Osun, Oyo, Ogun, Ekiti		Cancer (Prostate, Breast, Cervical)	Seeds/Dried fruit/Leaves/Root	Concoction/Decoction/Poultice	Oral/Topical	[82, 130, 157, 160, 181]
	Oyo, Ondo		Flu, Respiratory tract infection	Seeds/Leaves	Pulverisation/Infusion/Decoction	Oral	[17, 96]
	Bayelsa, Zamfara		Fracture, Luxation, Rheumatoid arthritis	Fruit/Stem	Paste	Topical	[16, 60]
	Oyo		Wound	Bark/Fruit	Concoction	Oral/Body bath	[179]
	Kwara, Southern Nigeria states		Malaria	Stem-bark/Seeds/Fruit/Leaves	Decoction	Unspecified	[59, 101, 146, 167, 178]
	Ogun, Oyo, Osun, Kwara		Male contraceptive	Fruit	Decoction	Oral	[42]
	Oyo, Ogun, Ekiti, Lagos		Hair growth	Leaves	Powder/Oil	Topical	[166]
	Oyo		Children scalp infections	Fruit	Decoction	Oral/Body bath	[150]
	Kwara		Meningitis, Lassa fever, Poliomyelitis	Seeds	Decoction	Oral	[158]
	Ogun		Boil, Skin irritation	Seeds	Paste	Topical/Oral	[82, 168]
	Ogun, Osun, Oyo		Asthma	Fruit	Maceration	Oral	[185]
	Kwara		Female sexual dysfunction, Female Infertility	Seeds	Unspecified	Unspecified	[84]
	Oyo		Skin diseases, Purgative, Diabetes, Dysentery	Bark/Seeds/Pod (without seed)	Decoction/Concoction/Powder	Unspecified	[58]
	Rivers		Cough, Rheumatism, Diarrhoea, Dysentery	Seeds/Fruit	Mastication/Poultice/Decoction	Oral/Topical	[41]
	Niger		Tuberculosis, Respiratory disorders	Fruit	Unspecified	Unspecified	[173]
	Ekiti, Ondo, Osun, Oyo		Measles, Chickenpox, Jaundice	Leaves/Fruit	Unspecified	Unspecified	[57]
*Zingiber officinale* Roscoe (Zingiberaceae	Oyo, Lagos	Chita (H.), Ata ile (Yor.), Jinja (Ig.)	COVID-19	Rhizomes	Decoction	Oral	[96]
	Ogun		Diabetes	Rhizomes	Maceration	Oral	[39]
	Oyo, Lagos		Cough, Indigestion, Flu	Rhizomes/Root	Decoction/Pulverisation/Infusion	Oral	[15, 96]
	Bayelsa, Zamfara		Pain relief, Fracture, Rheumatoid arthritis	Rhizomes/Leaf/Root/Bulb	Poultice	Topical	[16, 60]
	Kwara, Lagos, Sokoto, Ogun, Oyo, Ekiti		Cancer	Rhizomes	Concoction/Decoction/Powder mixed with black soap	Oral/Wash	[130, 157, 172, 181]
	Kwara, Southern Nigeria states		Malaria	Rhizome/Underground stem/Leaves	Maceration/Decoction	Oral	[101, 178] [59, 129, 146, 167, 176, 178]
	Ondo		Respiratory tract infection, Inflammation	Rhizomes	Pounding/Powdering	Oral	[17]
	Lagos		Migraine, Gastrointestinal tract disease, High blood pressure, Weight loss	Root/Rhizomes	Infusion	Oral	[40]
	Oyo		Intestinal worm	Rhizomes	Unspecified	Unspecified	[14]
	Ogun, Oyo		Wound	Rhizomes	Powder	Topical	[131]
	Sokoto		Yellow fever, COVID-19	Stem/Bark	Decoction	Oral	[158]
	Ogun, Osun, Oyo		Asthma	Rhizomes/Root	Pulverisation/Maceration/Decoction	Oral	[82, 185]
	Ogun		Typhoid	Root/Rhizomes	Mastication	Oral	[82, 168]
	Katsina		Liver detoxification, Bronchitis, Cough	Corm	Unspecified	Unspecified	[91]
	Rivers		Hypertension, Laxative, Cough, Catarrh, Stomachache	Rhizomes	Mastication/Juice extraction	Oral	[41]
	Niger		Tuberculosis, Respiratory disorders	Rhizomes	Unspecified	Unspecified	[173]
	Katsina, Akwa Ibom		Cough, Catarrh, Cold, Stomachache	Rhizomes	Mastication/Decoction	Oral	[61, 85]
	Ekiti, Ondo, Osun, Oyo		Poliomyelitis, Measles, Jaundice, Yellow fever	Rhizomes/Root	Unspecified	Unspecified	[57]

^*^Local name, H–Hausa, Yor–Yoruba, Ig–Igbo, Ef–Efik, Ur–Urhobo

Based on the generated plant inventory, some of the notable ones were *Abrus precatorius* L., *Vachellia nilotica* (L.) P.J.H. Hurter & Mabb. Syn., *Acacia nilotica* (L.) Willd. ex Delile, *Adansonia digitata* L., and *Aframomum melegueta* K. Schum. *Balanites aegyptiaca* (L.) Delile*, Carica papaya* L*., Vernonia amygdalina* Delile*, Boswellia dalzielii* Hutch*.*, and *Bambusa vulgaris* Schrad*.*, offering unique contributions to traditional medicine in Nigeria. These noteworthy plants address a range of conditions, including respiratory issues (cough, asthma), metabolic disorders (diabetes), skin ailments, inflammatory conditions (arthritis, rheumatism), infectious diseases (malaria), and cardiovascular concerns (hypertension).

The medicinal applications underscore the critical role of traditional knowledge in addressing health challenges in communities and its capacity to inform innovative treatment strategies. The 20 most utilised plants in Nigeria, identified by their high frequency of use (25 or more mentions) comprised of *Carica papaya* L., *Vernonia amygdalina* Delile, *Mangifera indica* L., and *Aframomum melegueta* K. Schum. *Azadirachta indica* A. Juss., *Jatropha curcas* L., and *Garcinia kola* Heckel, *Psidium guajava* L., *Allium sativum* L., *Cymbopogon citratus* (DC.) Stapf, *Citrus* × *aurantiifolia* (Christm.) Swingle, *Elaeis guineensis* Jacq., *Nauclea latifolia* Sm., *Zingiber officinale* Roscoe, *Vitellaria paradoxa* C.F Gaertn, *Morinda lucida* Benth., *Xylopia aethiopica* (Dunal) A.Rich., *Momordica charantia* L., *Ocimum gratissimum* L., and *Spondias mombin* L. are commonly utilised in traditional medicine for the treatment of gastrointestinal diseases, respiratory infections, malaria, and a range of other health issues. These plants play vital roles in African traditional medicine [116–118]. They address gastrointestinal diseases, respiratory tract infections, malaria, venereal diseases, cardiovascular diseases, wounds, and viral diseases.

In terms of the families for the 963 collated plant species, Fabaceae was the most dominant comprising 127 plants, followed by Malvaceae (52 plants) and Asteraceae (43 plants). Euphorbiaceae had 38 plants, while Apocynaceae and Rubiaceae comprised 36 plants each. About 33% (47 families) of 144 recorded families were represented by 1 plant each (Appendix 1). Fabaceae had twice the number of plant species compared to the Malvaceae, which was the next dominant family with a notable plant count. Generally, plants belonging to the Fabaceae are esteemed for their versatile applications in medicine, nutrition, and livestock health care, underscoring their importance from an ethnobotanical perspective [119].

**Fig. 4:**
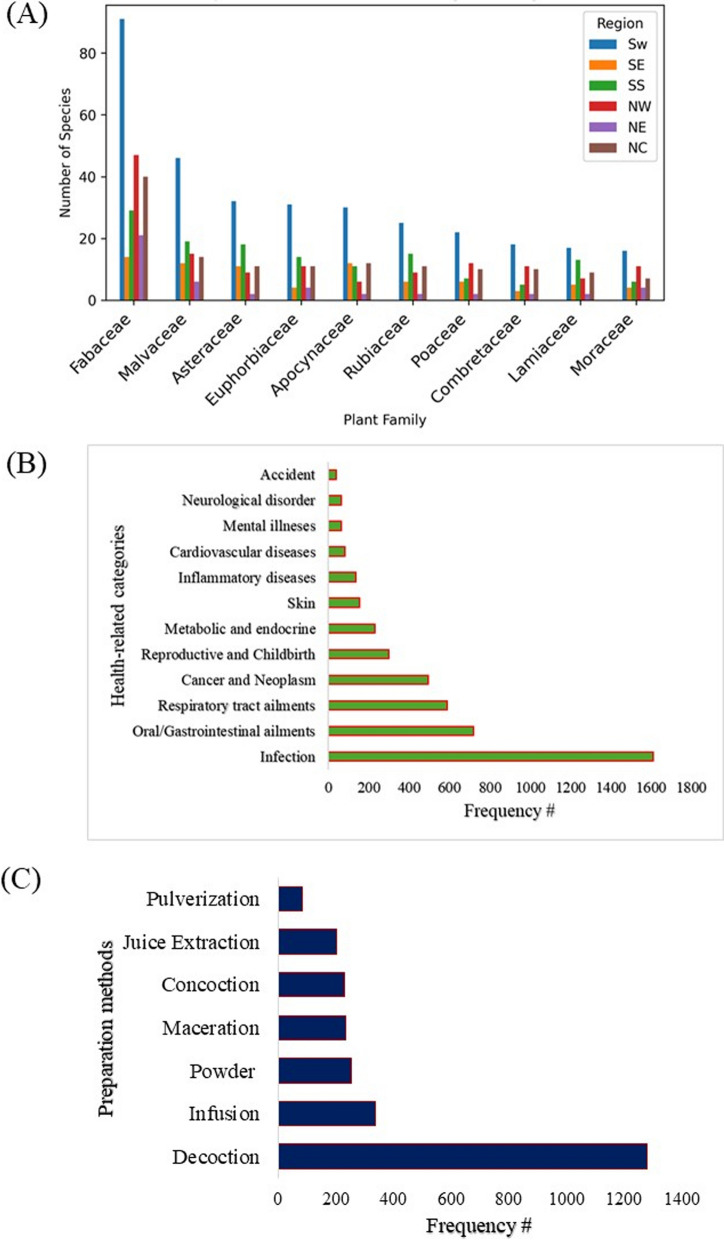
Frequency for the generated inventory of 963 plants with ethnobotanical uses in Nigeria from 1964 to 2024. **A** Distribution of the top 10 plant families across the six regions in Nigeria; **B** Top twelve (12) categories of ailments managed with plants in Nigeria; **C** preparation methods. Note that 144 plant families were recorded, and the number of mentions for the remaining 134 plant families (not shown here) ranged from 1 to 20 (Appendix 1)

From a cross regionally analysis, Fabaceae was the most represented family across all the six regions, with SW reporting the highest plant count (Fig. 4A). The ecological adaptability of Fabaceae and ethnobotanical versatility, including its use in traditional medicine, reaffirm its central role in African ethnobotany [119–121]. Recent hypotheses by Mongalo and Raletsena [121] offer evidence supporting the prominence of Fabaceae as a plant family recognised for its medicinal properties. African traditional medicine values plants in the Fabaceae due to their accessibility, prevalence, and remarkable adaptability to diverse environments [120]. Southwest region exhibited a preeminent presence of plants across numerous families, particularly within Malvaceae, Asteraceae, Euphorbiaceae, Apocynaceae, and Lamiaceae. This prevalence may be attributed to several factors, including the diverse rainforest ecology of the region, which fosters a richer assemblage of plant species and inevitably usages among local communities [122]. The observed trend highlighted the ecological richness characteristic of forested zones and signifies heightened ethnobotanical engagement and activity in these areas [123].

Northwest and NE regions, which are characterised as savanna zones, exhibited a moderate representation of Fabaceae and Combretaceae (Fig. 4A). Both families are well-adapted to arid environments, which accounts for their prominent presence in these areas. Fabaceae is known for its nitrogen-fixing capabilities, enhancing soil fertility and providing crucial resources for various herbivores. In contrast, Combretaceae is typically associated with woody vegetation that can withstand dry conditions. The savanna regions show a comparatively low species richness in families (e.g., Annonaceae and Moraceae) related to rainforest ecosystems. The scarcity of these families highlights the stark differences in biodiversity and ecological dynamics between the savanna and rainforest habitats.

In NC which can be regarded as a transitional profile emerges, there were a mix of plant species that straddles the characteristics of arid savannas and rainforest environments [124]. This region contributes intermediate values across most families, indicating a gradual shift in vegetation and potentially offering a unique blend of plants from both ecosystems. The transitional nature of NC enriches its biodiversity and serves as an important ecological corridor, facilitating plant species movement and genetic exchange between the contrasting zones of NW and NE.

Malvaceae is renowned for its diverse array of mucilaginous and medicinal plants, including genera such as *Abelmoschus* and *Adansonia*. This family exhibits a particularly prominent distribution in the SW and NW regions, where it has been integral to local ethnobotanical practices and traditional medicine. The mucilage properties of these plants are often harnessed for their therapeutic benefits, which underscores their significance in both cultural and contemporary pharmacology [79, 125]. In contrast, Asteraceae holds considerable global importance due to the presence of various species recognised for their medicinal properties [126]. Key genera such as *Vernonia* and *Aspilia* are particularly noteworthy, displaying a strong presence in SW and SS regions (Fig. 4A). This distribution aligns with areas historically associated with rich traditions of herbal medicine, where these plants are utilised extensively in local healthcare practices [126]. The prominence of these families in specific geographic regions revealed the intricate relationships between biodiversity, cultural practices, and the utilisation of natural resources for medicinal purposes.

Northeastern regions exhibit the lowest plant counts across the various botanical families (Fig. 4A). This observation may indicate several factors, including a climate characterised by aridity that may restrict overall biodiversity or possibly reflect a lack of comprehensive documentation pertaining to ethnobotanical knowledge within this area [78]. It is crucial to consider that the underrepresentation of plants could also stem from socio-cultural dynamics that influence the recording of traditional ecological knowledge.

Nonetheless, the observed utilisation of drought-resistant plants, particularly those belonging to the Combretaceae and Fabaceae families is noteworthy. This practice aligns with the ecological constraints imposed by the climatic conditions of the region and highlights an adaptive strategy among local populations [127]. The selective use of these resilient species underscores a deeper understanding of sustainable practices in the face of environmental challenges, illustrating the relationship between ecological adaptation and traditional knowledge systems. Further research is warranted to investigate the extent of ethnobotanical diversity and its implications for conservation strategies in these drier climates.

#### Assessment of health conditions managed with the medicinal plants and method of preparation

This review identified 78 distinct health conditions, categorised into several groups: infectious diseases (35.93%), oral/gastrointestinal ailments (16.03%), respiratory tract issues (13.15%), cancers and neoplasms (11.08%), reproductive and childbirth-related conditions (6.7%) (Fig. 4B). These classifications are based on the Health Research Classification System (HRCS). Environmental and socio-economic factors, such as the tropical climate, inadequate healthcare infrastructure, and industrial activities, significantly contribute to the high prevalence of these ailments in various tropical regions [37, 128, 129]. The utilisation of plants such as *Abrus precatorius* L., *Ageratum conyzoides* L., *Aloe vera* (L) Burm.f., *Anacardium occidentale* L., and *Annona muricata* L. in managing various ailments, including infectious diseases such as tuberculosis and hepatitis, and chronic conditions including diabetes and hypertension, highlight their therapeutic potential. Plants such as *Alstonia boonei* De Wild., *Azadirachta indica* A. Juss., *Carica papaya* L., *Citrus* × *aurantium* L., *Mangifera indica* L. are used to treat various ailments including malaria, tuberculosis, viral infections, diabetes, hypertension, cancer, burns, wounds, eczema, female infertility, postpartum issues, wound healing, benign prostatic hyperplasia, and depression have all documented [111, 130–133].

The most common preparation method for medicinal plant applications was decoction (Fig. 4C). This method effectively extracts beneficial properties from resilient plant materials, including roots and bark [134]. Following decoction, infusion, powdering, and maceration were also popular methods. The widespread use of these methods aims to maximise the extraction of bioactive compounds specific to various plant parts and their applications [134, 135]. The straightforward nature of decoction and infusion methods likely account for their prevalent applications in local communities. Decoction involves the heating or boiling of plant material in water, while infusion consists of immersing the plant material in cold or pre-warmed water [136]. Highlighting the importance of oral administration, it signifies a crucial role in addressing internal health conditions through herbal remedies. Topical applications play a crucial role in treating a range of external conditions such as wounds, skin diseases, and burns, showcasing the diverse applications of herbal therapies. This highlights the adaptability of herbal therapies, showing their effectiveness in treating both internal and external health concerns.

Furthermore, the preparation methods for the plants varied across regions. Decoction was most common in SW and SS for internal ailments, including malaria and diabetes, while poultices and infusions were prevalent in northern Nigeria and SE for skin and respiratory ailments, respectively. For instance, in SW Nigeria, *Corchorus olitorius* (Ewedu) is used for diabetes in a decoction form [46] while in SS, the root is infused for cancer treatment [103]. Similarly, people in the SS region of Nigeria use *Laportea aestuans* as a paste for skin diseases [48], while it is used to prevent bedwetting in the SW [115]. Some plants serve different functions depending on the region. In SW, people use *Aloe vera* as a topical infusion to treat acne and burns [19], and they also consume it as a juice to treat diabetes [134].

#### Contribution of the 79 eligible ethnobotanical studies to Nigeria primary healthcare system

Traditional medicine serves as the primary healthcare for many communities in Nigeria [137]. Documentation of information helps to protect cultural heritage and helps to make healthcare more accessible to people in rural and underserved areas [138]. In Nigeria, ethnobotanical surveys have systematically documented the use of medicinal plants in addressing primary healthcare needs [139]. These ethnobotanical surveys remain a pivotal mechanism for preserving traditional knowledge, which is indispensable for the sustainable use of natural resources in contemporary medicinal practices [46].

These findings from the 79 eligible studies underscored the vital role that Indigenous knowledge plays in informing and enhancing healthcare strategies and thereby facilitating a more integrative approach to healthcare that recognises the value of both traditional remedies and modern medical interventions [140]. The integration of ethnobotanical knowledge into mainstream healthcare could potentially lead to holistic treatment strategies that are tailored to the specific needs and contexts of diverse communities across Nigeria [141].

A study conducted in Lagos State recorded 183 plants used to manage various health conditions [15], highlighting the significant dependence on traditional medicine in the selected areas. These studies serve as essential repositories of cultural heritage, documenting and safeguarding the knowledge of local healers and practitioners amid globalisation and modernisation. Ethnobotanical research has led to the identification of numerous plants with therapeutic potential. A study conducted in Ilorin (NC Nigeria) revealed 47 plants from 28 families used to treat female sexual dysfunction and infertility [84]. The study highlighted a vast biodiversity of the region and how local communities use it to meet their healthcare needs. These plants included *Vitellaria paradoxa*, *Anogeissus leiocarpa*, and *Peperomia pellucida*, which were used to treat female sexual dysfunction and infertility [84].

The medicinal plants recorded from the 79 eligible studies are essential for primary healthcare, offering accessible and cost-effective treatment options for local communities. In Lagos State, researchers created a comprehensive checklist of medicinal plants used in primary healthcare, drawing on ethnobotanical surveys conducted in herbal markets [142]. This detailed catalogue illustrated the dependence of the communities on these natural resources for self-medication and treatment, especially in areas where formal healthcare services might be scarce [139]. In Delta State, the incorporation of valuable medicinal plants into local healthcare practices demonstrates how traditional medicine enhances the existing Western health systems [143].

The insights obtained from ethnobotanical studies provide an essential basis for pharmacological research and exploring the development of new drugs. A notable example is *Vernonia amygdalina*, commonly known as a bitter leaf in Nigerian traditional medicine and recognised for its diverse medicinal properties, including antimalarial and antidiabetic effects [101, 111]. This plant has attracted the attention of researchers who are investigating its active compounds for possible therapeutic uses [144]. Furthermore, well-known Nigerian pharmacologists have researched traditional medical practices and how they might be used in modern medicine [145]. This work paves the way for the development of new drugs that are driven by traditional knowledge. Ethnobotanical studies are essential to establish the value of local floras and generally promote the importance of biodiversity conservation. Studies in locations such as the Old Oyo National Park have documented a range of medicinal plants [58] endemic to these ecosystems, highlighting their importance for health and maintaining ecological equilibrium. These studies highlight the importance of conservation, motivating local communities and policymakers to focus on protecting their plants.

#### Indigenous knowledge-driven diagnosis and treatment

The findings from the 79 eligible studies underscored a range of conventional diagnostic methods used by traditional healers and herbalists (Tables 2 and 3), which reflect a unique interplay between cultural practices and healthcare.

*Diagnosis based on observational signs*: traditional healers apply a symptomatology approach that highlights sharp observational skills to recognise illnesses such as diabetes and malaria. For example, diabetes is frequently identified by noticeable symptoms including polyuria (frequent urination) and unexplained weight loss, which are acknowledged markers within the community [39, 111]. Practitioners and patients recognise a range of symptoms, including fever, chills, and fatigue, as indicators of malaria [59, 146].

*Integration of spiritual and herbal elements*: The combination of spiritual and herbal elements in healing practices embodies a comprehensive approach that recognises the interrelationship between physical, emotional, and spiritual well-being [147]. Traditional healers hold the view that health problems stem from physical factors, spiritual influences, cultural beliefs, and individual experiences [148]. Numerous practitioners use divination methods to reveal the root causes of health issues [147]. The combination of these insights with herbal treatments grounded in traditional knowledge effectively tackles both the symptoms and underlying causes of ailments, which are frequently associated with emotional or spiritual distress [149]. This synthesis embodies a comprehensive perspective on health that goes beyond the common Western medicine, advocating for a worldview that appreciates spirituality and the natural world [148]. Integrative practices can improve well-being and empower individuals by promoting balance and harmony.

*Symptom-focused treatment approaches*: Community often adopts a model of healthcare that is predominantly symptom-focused, addressing various ailments, ranging from diarrhoeal diseases to dermatological infections, through a reliance on observable symptoms coupled with collective folklore and understanding of these health conditions [10, 150]. This practice underscores the experiential knowledge of the community, which is often rooted in shared cultural narratives.

Take together, it is imperative to advocate for the integration of traditional diagnostic methodologies into contemporary community health programs. Such integration acknowledges the value of indigenous knowledge systems and fosters a more inclusive approach to health care. Furthermore, rigorous research is essential to substantiate the effectiveness and reliability of these conventional diagnostic techniques, thereby enhancing their credibility and acceptance within the broader healthcare system.

### Trends and patterns in ethnobotanical research in Nigeria

**Fig. 5:**
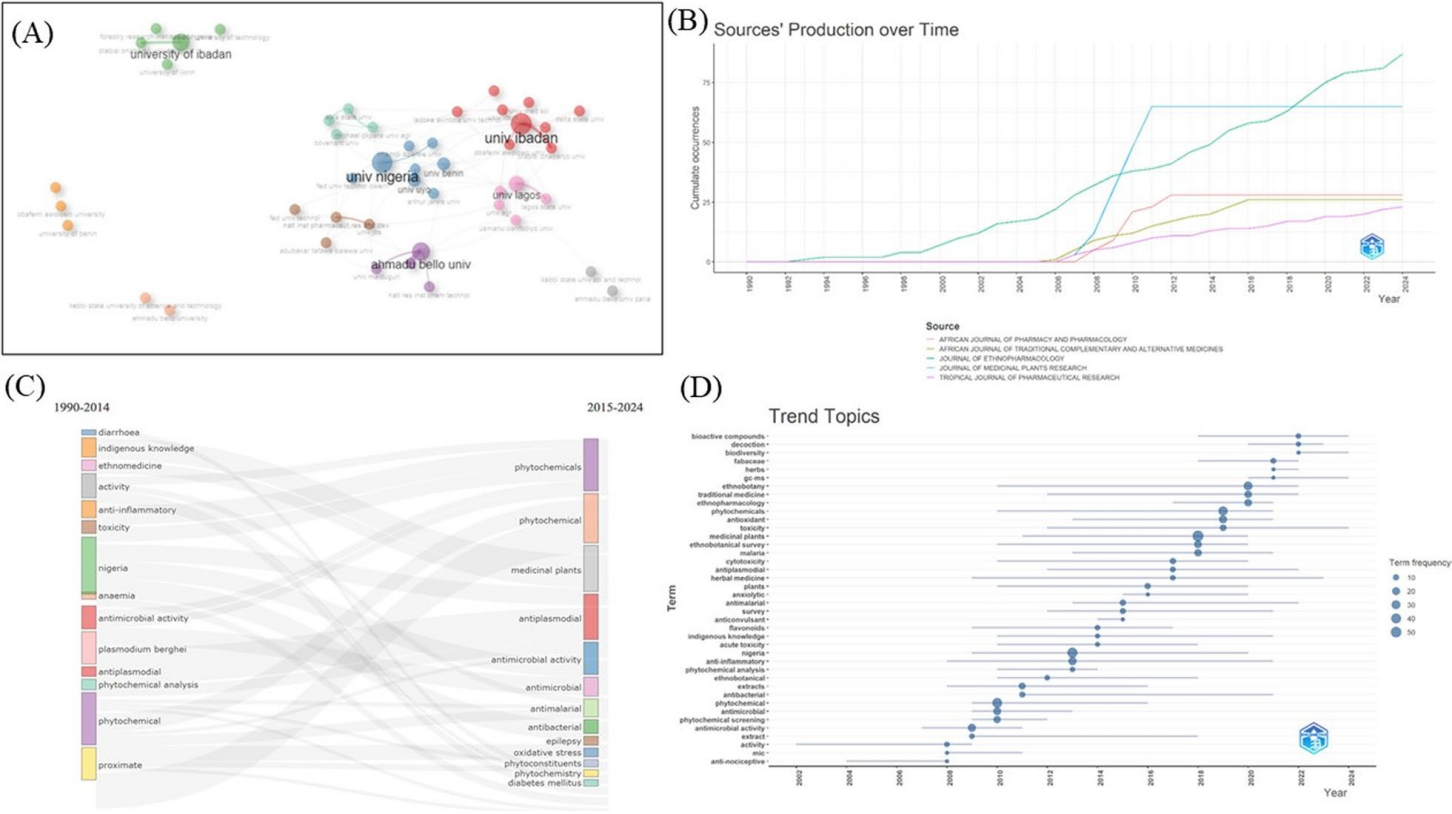
Research patterns and trends on ethnobotanical uses of plants in Nigeria from 1964 to 2024. **A** Visualisation of collaborative networks among universities in Nigeria; **B** Annual source production of scientific outputs in dominant journals over the past six decades; **C** Thematic evolution of ethnobotanical research and **D** Trend of topics associated with ethnobotanical research in Nigeria

Ethnobotanical research in Nigeria has experienced notable transformations in the last 60 years (1964–2024), influenced by an increasing acknowledgement of the importance of indigenous knowledge, medicinal plants, and biodiversity conservation. This analysis examined the key trends, thematic focuses, collaborative dynamics, and research outputs during this period (Fig. 5). The bibliometric analysis from 1 st January 1964 to 30th June 2024 revealed the patterns and expansion of ethnobotanical research in Nigeria.

#### Initial phase (1964–1989): Fundamental period

From 1964 to 1989, ethnobotanical research in Nigeria was predominantly investigative, concentrating on the documentation of indigenous knowledge regarding medicinal plants [142]. Researchers initiated the aggregation of data concerning medicinal flora, underscoring the significance of indigenous wisdom in healthcare [151]. In the late 1980s and early 1990s, ethnobotany experienced a surge in scholarly engagement, with an increasing number of researchers, including botanists and anthropologists, collaborating to document plant species, their applications, and cultural significance [152, 153]. This period witnessed the establishment of Botanical gardens and research institutions in Nigeria, serving as central hubs for ethnobotanical research [154]. Some challenges impede the growth of ethnobotanical research during this era, including insufficient funding, restricted pharmacological research, and surveys highlighting local flora and their roles in traditional healthcare.

#### Expansion and growth (1990–2009): Shifting focus in research

The 1990s experienced a notable rise in ethnobotanical studies, driven by thematic expansion, advancements in phytochemistry, investigations into antimicrobial activity, and the exploration of plant-based treatments for diseases, such as malaria and diabetes. In this phase, researchers were primarily concentrating on the preservation of ethnobotanical knowledge through documentation, collaborating with environmental organisations, and using technological tools to investigate plant genetics and biodiversity.

#### Modernisation and integration (2010–2024)

The past decade witnessed the most dynamic changes in Nigerian ethnobotanical research, characterised by interdisciplinary approaches and advanced methodologies. The research ranges from molecular studies that use genomics and proteomics to identify plant metabolites, antioxidant research that concentrates on oxidative stress-related diseases, and toxicology studies that examine the safety profiles of ethnobotanical formulations. The key themes were the focus on preserving indigenous medicine, addressing climate change, and rigorously testing ethnomedicinal claims to ensure their preservation and validity.

#### Institutional impact and growth

Institutions such as the University of Ibadan, Ahmadu Bello University, and others in Nigeria have emerged as main research centres (Fig. 5A), establishing facilities focused on biodiversity and pharmacognosy. The chart depict how the Nigerian institutions involved in ethnobotanical research collaborate. The nodes show the amount of research being done, and the edges show the connections between the institutions. The closer the institutions are, the stronger the connections. The University of Ibadan holds the largest and most central node in Nigeria’s network, signifying its leading role in ethnobotanical research in Nigeria. It collaborates extensively with other institutions, demonstrating its influence in the field. Ahmadu Bello University and the University of Lagos also occupy central positions, making major contributions to ethnobotany and related disciplines.

The chart identifies distinct groups of institutions. For example, the University of Ibadan works with the Forestry Research Institute of Nigeria [155], and the University of Ilorin focuses on studying biodiversity, traditional medicine, and phytochemicals. Within the University of Nigeria cluster, institutions such as the Michael Okpara University of Agriculture and the Federal University of Technology (Owerri) collaborate specifically on agricultural ethnobotany. The Ahmadu Bello University Cluster includes institutions such as the National Research Institute for Chemical Technology, indicating a focus on applied sciences and ethnopharmacology. Prominent institutions, such as the University of Ibadan and the University of Nigeria, which are characterised by large nodes and numerous connections, play a pivotal role in spearheading multi-institutional projects and fostering knowledge exchange. Conversely, smaller institutions, with fewer connections, specialise in niche ethnobotany. Strong partnerships between well-known institutions show strong support from funding bodies or national research priorities, while weaker partnerships between institutions in the periphery show fewer connections.

#### Analysis of collaboration and publication trends on ethnobotanical research in Nigeria

The University of Ibadan and Ahmadu Bello University dominate publications, fostering networks with other universities. Figure 5B depicts the publication patterns and trends of ethnobotanical journals over the past six decades. The graph illustrates the frequency of ethnobotanical research publications in five prominent journals from 1990 to 2024. These are the African Journal of Pharmacy and Pharmacology, the African Journal of Traditional, Complementary, and Alternative Medicines, the Journal of Ethnopharmacology, the Journal of Medicinal Plant Research, and the Tropical Journal of Pharmaceutical Research. The Journal of Medicinal Plants Research has undergone considerable expansion from 2007 to 2015. The Journal of Ethnopharmacology has consistently grown, but after 2010, the Journal of Medicinal Plants Research overtook it. The Tropical Journal of Pharmaceutical Research and the African Journal of Pharmacy and Pharmacology exhibit moderate growth. Before 2000, ethnobotanical research in Nigeria was scanty in the journals. Between 2000 and 2010, there was a significant rise in publication frequency, driven by global interest in traditional medicine, biodiversity, and the quest for bioactive chemicals [156].

From 2010 to 2024, pharmacology and medicinal plants made a lot of progress. This shows that more and more researchers are interested in ethnobotany and how it affects pharmacology. The popularity of Journal of Medicinal Plants Research shows how much it values plant-based research, while the consistent contributions of the Journal of Ethnopharmacology signify its dedication to the pharmacological substantiation of traditional wisdom. The slow growth of other journals likely suggests the compartmentalisation of research themes. The growing importance of African-based journals in studying biodiversity in Nigeria underscores the importance of local initiatives in documenting and analysing the ecosystems of the country. International journals such as the Journal of Ethnopharmacology underscore the global significance of ethnobotanical research. Future initiatives include expanding journals focused on African biodiversity and conservation, integrating ethnobotany with biotechnology, genetics, and pharmacology, and influencing policy formulations for the conservation and sustainable utilisation of plant resources.

#### Thematic evolution of ethnobotanical research in Nigeria

The thematic evolution chart shows the progression of research themes for Nigerian plants from 1990–2014 to 2015–2024 **(**Fig. 5C). The chart illustrates the relationship between older and newer research words. This Sankey-style visualisation elucidates the evolution of research objectives and the emergence of themes over time. The persistent emphasis on"medicinal plants"and"phytochemicals"indicates their importance in the study of Nigerian plants. These themes connect the earlier (1990–2014) and later (2015–2024) periods, demonstrating their fundamental importance in ethnobotany and phytochemistry research. Interest in"antimicrobial activity"has persisted for decades, showing a continued emphasis on developing plant-based remedies to prevent microbial illnesses.

Between 1990 and 2014, research focused on indigenous knowledge, ethnomedicine, and toxicity, specifically addressing health issues, such as diarrhoea and anaemia. Research on malaria and antiplasmodial activity was prominent, highlighting the continued importance of traditional remedies in addressing public health challenges. Between 2015 and 2024, there was a huge increase in research on oxidative stress, epilepsy, phytoconstituents, and diabetes mellitus. Phytochemistry and phytochemical analysis study the molecular and chemical makeup of plants. The ongoing investigation into antibacterial and antimalarial activities suggests a promising avenue for targeted therapeutic applications.

Similarly, the evolution of plant research in Nigeria, from traditional knowledge to more specialised molecular and applied research methodologies (Fig. 5C). This development underscores the implementation of contemporary scientific approaches and the variety of themes involved. Nonetheless, the lack of conservation-related terminology highlights a deficiency in tackling the sustainability of plant resources. The investigation underscores the significance of research on Nigerian flora in tackling health challenges that are pertinent both locally and globally. The thematic evolution chart underscores the necessity to broaden investigations into conservation themes, enhance partnerships between local communities and scholars, and prioritise emerging health issues, including chronic diseases, all while continuing to address infectious diseases.

Evidence from this review illustrated a rising interest in ethnobotany and medicinal plants in Nigeria, especially after 2010 (Fig. 5D). The subjects of “ethnopharmacology” and “indigenous knowledge” correspond with the synthesis of traditional applications and scientific inquiry. Terms such as “phytochemical screening” and “flavonoids,” which refer to research into bioactive compounds, show that phytochemical studies have gained popularity. Health applications encompass antimicrobial, anti-inflammatory, and antimalarial properties for therapeutic purposes. The growing interest in anxiolytic and anticonvulsant activity indicates potential neurological benefits. Conservation and biodiversity are under examination, with the term “biodiversity” reaching a plateau, indicating a stable level of species diversity that may have implications for conservation efforts. Advancing conservation in conjunction with therapeutic discoveries is essential for the sustainable preservation and use of plant resources in Nigeria. For example, the conservation of specific medicinal plant species alongside the development of effective therapies can ensure long-term availability and benefit for future generations.

#### Overview on the word cloud related to ethnobotanical research in Nigeria

**Fig. 6:**
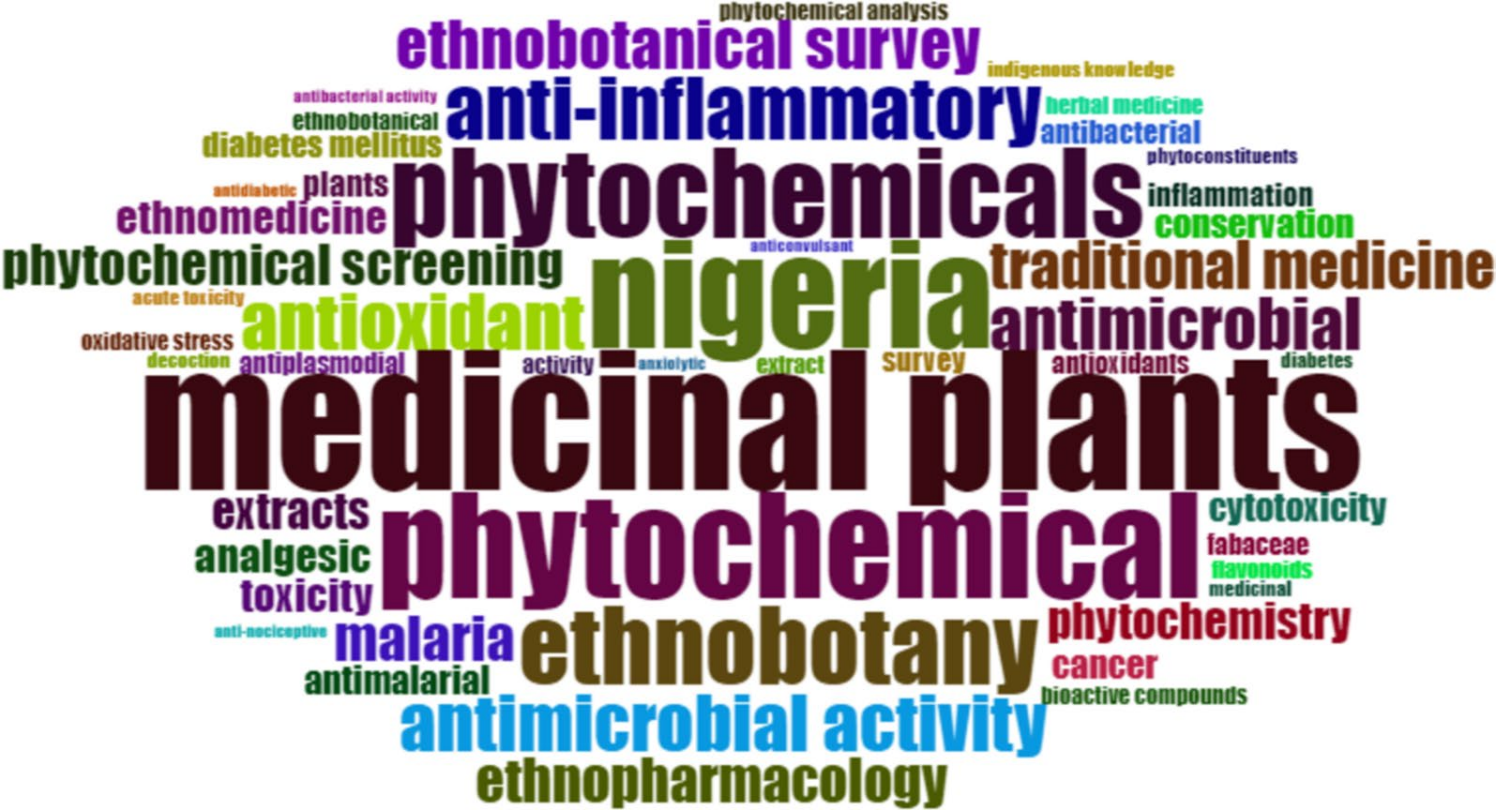
Overview of the recurrent terminology relevant to ethnobotanical study in Nigeria

The word cloud visualisation presents a graphical depiction of keywords and themes associated with Nigerian plant research (Fig. 6). The size of each term correlates with its frequency or prominence in the studied literature or data. The predominant keyword in the word cloud signifies a substantial emphasis on medicinal plants within Nigerian studies. This underscores their cultural, traditional, and commercial significance as a source of natural cures and pharmaceutical precursors. Terminology such as “phytochemicals,” “phytochemistry,” and “phytochemical screening” underscores the examination of chemical substances in flora. According to this, the study of bioactive chemicals that have medical effects includes flavonoids, which are antioxidants, alkaloids, which relieve pain, and terpenoids, which reduce inflammation. The terms “toxicity” and “cytotoxicity” underscore the emphasis on evaluating safety profiles and potential therapeutic dosages for plant extracts.

The significance of “ethnopharmacology” illustrates the incorporation of traditional remedies within the realm of pharmacological inquiry. The use of words such as “antimicrobial,” “antioxidant,” “anti-inflammatory,” “antimalarial,” and “cytotoxicity” shows that people are trying to find treatments for infections, cancer, oxidative stress, malaria, and inflammation that come from plants. The word cloud illustrates the extensive range of applications for Nigerian flora, encompassing the treatment of infectious diseases (e.g., “antimicrobial activity”) as well as the management of chronic conditions, such as diabetes and cancer.

## Limitations of the review

The overall value of this review with respective to its impact on Nigerian ethnobotany cannot be overemphasised but some limitations were unavoidable. These constraints may have affected the comprehensiveness, applicability, and generalisability of the findings to certain extend. We highlight the limitations below.

- The review covered the period 1964 to 2024, and the restricted time frame means the exclusion of literature outside this period. The use of plants was long existing prior to 1964 and the implications of this time limit was not addressed in this review.
- We focused on articles published in English and excluded materials in Nigerian local languages of multi-lingual nature of the country. Particularly, it could have been practically impossible and financially strenuous to hire several translators required to ensure the reliability of such information. This implies that existing data written in local languages were omitted in our generated inventory of plants, thereby affecting the completeness of Nigerian ethnobotany as recorded in this review.
- The number and choice of the databases used for this systematic review may have conferred some restriction on the total number of eligible articles that were generated. Searching databases can be laborious and time-consuming, which influence the restricted number of selected databases for sourcing the eligible articles.
- This review was primarily confined to an ethnobotanical perspective, with minimal integration of insights from related disciplines, such as ethnopharmacology, phytochemistry, and conservation biology. This lack of interdisciplinary engagement may affect the overall elucidation on the implications of Nigerian ethnobotany.

While we acknowledge these limitations, the findings from this review remain a vital reference materials for Nigerian ethnobotany as the integrity, reliability and validity of data reported cannot be disputed.

## Conclusion

This review meticulously examined the ethnobotanical applications of various plant species in Nigeria, emphasising the intricate connections between Indigenous communities and their ecosystems. We revealed the dynamic evolution of ethnobotanical research and Indigenous knowledge related to plant use in Nigeria, which has been driven by rich biodiversity. Application of systematic approach yielded 79 eligible studies spanning six decades, which generated 963 plant species across 144 families with diverse ethnobotanical applications. Medicinal applications emerged as the predominant category, highlighting the continued relevance of plant-based remedies in addressing public health challenges in Nigeria. The findings illustrated the extensive connections between traditional knowledge, ecological diversity, and healthcare practices across the six geopolitical regions in Nigeria. Notable plants such as *Carica papaya*, *Vernonia amygdalina*, *Cola acuminata*, and *Garcinia kola* exemplified the cultural, medicinal, and economic significance of botanicals among the various ethnic groups. Fabaceae was the most represented and distributed plant family across the six regions in Nigeria. Decoction, infusion, and maceration were the most common preparation methods, emphasising the practicality and accessibility of plants for traditional healing practices.

Despite the increasing body of ethnobotanical research, significant challenges persist, including inadequate documentation of indigenous knowledge, limited interdisciplinary collaboration, and the underrepresentation of specific geographic regions. Additionally, variations in data collection methodologies remain a major hindrance to the standardisation and generalisation of the existing findings. Moving forward, bridging the gap between traditional and modern medicine through policy integration, collaborative research, and conservation initiatives is crucial. Particularly, strengthening interdisciplinary partnerships among ethnobotanists, medical practitioners, and policymakers will enhance the generation of scientific evidence on the efficacy and sustainable utilisation of medicinal plants. Furthermore, expanding research coverage to underrepresented regions will ensure a more comprehensive understanding of the ethnobotanical landscape in Nigeria. By addressing these research gaps, the potential for integrating traditional medicine into the primary healthcare system in Nigeria can be maximised, preserving invaluable Indigenous knowledge for future generations while promoting public healthcare and biodiversity conservation.

## Supplementary Information

Additional file 1.

Additional file 2.

Additional file 3.

## Data Availability

Data on ethnobotanical use of botanical resources in Nigeria entails dataset that we have collated and deposited, the dataset is publicly available on Figshare: 10.6084/m9.figshare.28376042.

## Abbreviations

FRIN: Forestry Research Institute of Nigeria
ICF: Informant consensus factor
NC: North-central
NE: Northeast
NW: Northwest
PRISMA: Preferred reporting items for systematic reviews and meta-analyses
RFC: Relative frequency of citation
SE: Southeast
SS: South-south
SW: Southwest
UN SDGs: United Nations sustainable development goals
UV: Use value
