# Supplementary material for: Ethnobotanical uses of plants in Nigeria: an analysis of current research trends and patterns
Source: J Ethnobiol Ethnomed. 2025 Aug 21;21:57. doi: 10.1186/s13002-025-00788-y (PMC12369106; doi:10.1186/s13002-025-00788-y)
Supplement: Supplementary file 1 — Additional file 1. [file 13002_2025_788_MOESM1_ESM.docx]

**Supplementary Table S1:** Assessment of the quality of the included ethnobotanical studies using the Critical Appraisal Skills Programme Tool.

| Authors | Was there a clear statement of the research aims? | Is the qualitative methodology suitable? | Was the research design suitable for achieving the research objectives? | Was the recruitment strategy aligned with the research objectives? | Did the data collection effectively address the research issue? | Has the relationship between the researcher and participants been sufficiently addressed? | Have ethical considerations been addressed? | Was the data analysis adequately rigorous? | Does the document provide a definitive statement of findings? | To what extent is the research valuable? |
| --- | --- | --- | --- | --- | --- | --- | --- | --- | --- | --- |
| Abdallah *et al.* (2020) | Yes | Yes | Yes | Yes | Yes | Yes | No | Yes | Yes | Yes |
| Abo *et al.* (2008) | Yes | Yes | Yes | Yes | Yes | Yes | No | No | Yes | Yes |
| Abubakar *et al.* (2017) | Yes | Yes | Yes | Yes | Yes | Yes | No | No | Yes | Yes |
| Abubakar *et al*. (2020) | Yes | Yes | Yes | Yes | Yes | Yes | Yes | Unable to determine | Yes | Yes |
| Abubakar *et al*. (2020) | Yes | Yes | Yes | Yes | Yes | Yes | Yes | Yes | Yes | Yes |
| Abubakar *et al*. (2022) | Yes | Yes | Yes | Yes | Yes | Yes | Yes | Yes | Yes | Yes |
| Adebisi and Bello (2011) | Yes | Yes | Yes | Yes | Yes | Yes | No | No | Yes | Yes |
| Adedeji *et al.* (2018) | Yes | Yes | Yes | Yes | Yes | Yes | No | No | Yes | Yes |
| Adekunle *et al*. (2007) | Yes | Yes | Yes | Yes | Yes | Unable to determine | No | No | Yes | Yes |
| Afolayan and Sowemimo (2023) | Yes | Yes | Yes | Yes | Yes | Yes | No | Yes | Yes | Yes |
| Afolayan *et al*. (2020) | Yes | Yes | Yes | Yes | Yes | Yes | Yes | Yes | Yes | Yes |
| Ajibesin *et al*. (2008) | Yes | Yes | Yes | Yes | Yes | Yes | No | Yes | Yes | Yes |
| Ajibesin *et al*. (2012) | Yes | Yes | Yes | Yes | Yes | Yes | No | Yes | Yes | Yes |
| Ali *et al*. (2022) | Yes | Yes | Yes | Yes | Yes | Yes | No | Yes | Yes | Yes |
| Ambali *et al*. (2021) | Yes | Yes | Yes | Yes | Yes | Yes | Yes | Yes | Yes | Yes |
| Ampitan (2013) | Yes | Yes | Unable to determine | Yes | Yes | Yes | No | No | Yes | Yes |
| Amusa *et al*. (2010) | Yes | Yes | Yes | Yes | Yes | Yes | No | Yes | Yes | Yes |
| Ashidi *et al*. (2010) | Yes | Yes | Yes | Unable to determine | Yes | Yes | No | Unable to determine | Yes | Yes |
| Ashidi *et al*. (2021) | Yes | Yes | Yes | Yes | Yes | Yes | No | No | Yes | Yes |
| Atawodi *et al*. (2014) | Yes | Yes | Unable to determine | Yes | Yes | Yes | No | No | Yes | Yes |
| Aworinde and Erinoso (2015) | Yes | Yes | Yes | Unable to determine | Yes | Unable to determine | No | No | Yes | Yes |
| Aworinde *et al*. (2018) | Yes | Yes | Yes | Yes | Yes | Yes | No | No | Yes | Yes |
| Ayeni and Kayode (2019) | Yes | Yes | Yes | Yes | Yes | Yes | No | No | Yes | Yes |
| Borokini and Omotayo (2012) | Yes | Yes | Yes | Unable to determine | Yes | No | No | Unable to determine | Yes | Yes |
| Dike *et al*. (2012) | Yes | Yes | Yes | Yes | Yes | Yes | No | Unable to determine | Yes | Yes |
| Enebeli-Ekwutoziam *et al*. (2021) | Yes | Yes | Yes | Yes | Yes | Yes | Yes | Yes | Yes | Yes |
| Erinoso and Aworinde (2012) | Yes | Yes | Yes | Unable to determine | Yes | Yes | No | Yes | Yes | Yes |
| Etuk *et al*. (2009) | Yes | Yes | Yes | Yes | Yes | Yes | Yes | Yes | Yes | Yes |
| Evbuomwan *et al*. (2023) | Yes | Yes | Yes | Yes | Yes | Yes | No | Yes | Yes | Yes |
| Fred-Jaiyesimi and Ajibesin (2012) | Yes | Yes | Yes | Yes | Yes | Yes | Yes | Yes | Yes | Yes |
| Fred-Jaiyesimi *et al*. (2015) | Yes | Yes | Yes | Yes | Yes | Yes | No | Yes | Yes | Yes |
| Gbolade (2009) | Yes | Yes | Yes | Yes | Yes | Yes | Yes | No | Yes | Yes |
| Hassan *et al.* (2023) | Yes | Yes | Yes | Yes | Yes | Unable to determine | Yes | Yes | Yes | Yes |
| Ibrahim *et al*. (2007) | Yes | Yes | Yes | Yes | Yes | Yes | Yes | Yes | Yes | Yes |
| Idowu *et al*. (2010) | Yes | Yes | Yes | Yes | Yes | Yes | Yes | Yes | Yes | Yes |
| Idu *et al.* (2010) | Yes | Yes | Yes | Yes | Yes | Yes | Yes | No | Yes | Yes |
| Igoli *et al.* (2004) | Yes | Yes | Yes | Yes | Yes | Yes | No | Yes | Yes | Yes |
| Ishola *et al*. (2014) | Yes | Yes | Yes | Yes | Yes | Yes | Yes | Yes | Yes | Yes |
| Iyamah and Idu (2015) | Yes | Yes | Yes | Yes | Yes | Yes | Yes | Yes | Yes | Yes |
| Kadiri *et al*. (2015) | Yes | Yes | Yes | Yes | Yes | Unable to determine | No | Yes | Yes | Yes |
| Kankara *et al*. (2015) | Yes | Yes | Yes | Yes | Yes | Yes | No | Yes | Yes | Yes |
| Kayode (2005) | Yes | Yes | Yes | Yes | Yes | Yes | No | Yes | Yes | Yes |
| Lawal *et al*. (2020) | Yes | Yes | Yes | Yes | Yes | Yes | Yes | Yes | Yes | Yes |
| Lawal *et al.* (2022) | Yes | Yes | Yes | Yes | Yes | Yes | Yes | Yes | Yes | Yes |
| Lor *et al.* (2017) | Yes | Yes | Yes | Yes | Yes | Yes | Yes | Yes | Yes | Yes |
| Mahmoud *et al*. (2020) | Yes | Yes | Yes | Yes | Yes | Yes | No | Yes | Yes | Yes |
| Malami *et al.* (2020) | Yes | Yes | Yes | Yes | Yes | Yes | Yes | Yes | Yes | Yes |
| Mann *et al*. (2007) | Yes | Yes | Yes | Yes | Yes | Yes | No | Yes | Yes | Yes |
| Ngulde *et al.* (2014) | Yes | Yes | Yes | Yes | Yes | Yes | No | No | Yes | Yes |
| Nurudeen *et al*. (2022) | Yes | Yes | Yes | Yes | Yes | Yes | No | Yes | Yes | Yes |
| Nwosu (2002) | Yes | Yes | Yes | Yes | Yes | Yes | No | No | Yes | Yes |
| Odebunmi *et al*. (2022) | Yes | Yes | Yes | Yes | Yes | Yes | No | Yes | Yes | Yes |
| Odewo *et al*. (2022) | Yes | Yes | Yes | Yes | Yes | Yes | No | No | Yes | Yes |
| Odoh *et al.* (2018) | Yes | Yes | Yes | Yes | Yes | Yes | Yes | Yes | Yes | Yes |
| Ofeimun and Temitope (2019) | Yes | Yes | Yes | Yes | Yes | Yes | Yes | Yes | Yes | Yes |
| Offiah *et al*. (2011) | Yes | Yes | Yes | Yes | Yes | Yes | Yes | No | Yes | Yes |
| Ogbole and Ajaiyeoba (2010) | Yes | Yes | Yes | Yes | Yes | Yes | Yes | No | Yes | Yes |
| Ogunkunle and Ladejobi (2006) | Yes | Yes | Yes | Yes | Yes | Yes | No | No | Yes | Yes |
| Ohemu *et al.* (2024) | Yes | Yes | Yes | Yes | Yes | Yes | Yes | Yes | Yes | Yes |
| Ojetunde *et al.* (2021) | Yes | Yes | Yes | Yes | Yes | Yes | Yes | Yes | Yes | Yes |
| Oladeji and Agbelusi (2018) | Yes | Yes | Yes | Yes | Yes | Yes | No | Yes | Yes | Yes |
| Oladunmoye and Kehinde (2011) | Yes | Yes | Yes | Yes | Yes | Unable to determine | Yes | Unable to determine | Yes | Yes |
| Olanipekun (2023) | Yes | Yes | Yes | Yes | Yes | Yes | Yes | Yes | Yes | Yes |
| Olatokunbo *et al*. (2022) | Yes | Yes | Yes | Yes | Yes | Unable to determine | No | No | Yes | Yes |
| Olorunnisola *et al*. (2013) | Yes | Yes | Yes | Yes | Yes | Yes | Yes | No | Yes | Yes |
| Omotayo and Borokini (2012) | Yes | Yes | Yes | Yes | Yes | No | No | Unable to determine | Yes | Yes |
| Oyeyemi *et al*. (2019) | Yes | Yes | Yes | Yes | Yes | Yes | Yes | No | Yes | Yes |
| Rafiu and Sonibare (2017) | Yes | Yes | Yes | Yes | Yes | Unable to determine | Yes | Yes | Yes | Yes |
| Salihu *et al.* (2018) | Yes | Yes | Yes | Yes | Yes | Yes | Yes | Yes | Yes | Yes |
| Segun *et al.* (2018) | Yes | Yes | Yes | Yes | Yes | Yes | No | Yes | Yes | Yes |
| Shinkafi *et al*. (2015) | Yes | Yes | Yes | Yes | Yes | Yes | No | Yes | Yes | Yes |
| Soladoye *et al.* (2005) | Yes | Yes | Yes | Yes | Yes | Unable to determine | No | No | Yes | Yes |
| Sonibare and Abegunde (2012) | Yes | Yes | Yes | Yes | Yes | Yes | Yes | Yes | Yes | Yes |
| Sonibare and Ayoola (2015) | Yes | Yes | Yes | Yes | Yes | Yes | Yes | Yes | Yes | Yes |
| Sonibare and Gbile (2008) | Yes | Yes | Yes | Yes | Yes | Unable to determine | No | No | Yes | Yes |
| Sonibare *et al*. (2009) | Yes | Yes | Yes | Yes | Yes | Yes | No | No | Yes | Yes |
| Sonibare *et al*. (2015) | Yes | Yes | Yes | Yes | Yes | Yes | Yes | Yes | Yes | Yes |
| Sulaiman *et al*. (2022a) | Yes | Yes | Yes | Yes | Yes | Yes | No | Yes | Yes | Yes |
| Yaradua and El-Ghani (2014) | Yes | Yes | Yes | Yes | Yes | Yes | No | Yes | Yes | Yes |
