# Supplementary material for: Ethnobotanical uses of plants in Nigeria: an analysis of current research trends and patterns
Source: J Ethnobiol Ethnomed. 2025 Aug 21;21:57. doi: 10.1186/s13002-025-00788-y (PMC12369106; doi:10.1186/s13002-025-00788-y)
Supplement: Supplementary file 2 — Additional file 2. [file 13002_2025_788_MOESM2_ESM.docx]

**Supplementary material 2:** Cross-cultural analysis of plant utilisation across the six (6) regions in Nigeria

| S/N | Southwest (SW) | Southeast (SE) | South-South (SS) | Northwest (NW) | Northeast (NE) | North Central (NC) |
| --- | --- | --- | --- | --- | --- | --- |
| 1 | *Abelmoschus esculentus (L.) Moench (Malvaceae)* | *Abrus precatorius L. (Fabaceae)* | *Abelmoschus esculentus* (L.) Moench (Malvaceae) | *Abelmoschus esculentus* (L.) Moench (Malvaceae) | *Abelmoschus esculentus* (L.) Moench (Malvaceae) | *Abelmoschus esculentus* (L.) Moench (Malvaceae) |
| 2 | *Abrus precatorius L. (Fabaceae)* | *Acanthospermum hispidum DC. (Asteraceae)* | *Abrus precatorius* L. (Fabaceae) | *Abrus precatorius* L. (Fabaceae) | *Acanthospermum hispidum* DC. (Asteraceae) | *Abrus precatorius* L. (Fabaceae) |
| 3 | *Acalypha fimbriata Schumach. & Thonn. (Euphorbiaceae)* | *Achyranthes aspera L. (Amaranthaceae)* | *Acalypha fimbriata* Schumach. & Thonn. (Euphorbiaceae) | *Acanthospermum hispidum* DC. (Asteraceae) | *Achyranthes aspera* L. (Amaranthaceae) | *Acanthospermum hispidum* DC. (Asteraceae) |
| 4 | *Acalypha wilkesiana Müll.Arg. (Euphorbiaceae)* | *Adansonia digitata L. (Malvaceae)* | *Acanthospermum hispidum* DC. (Asteraceae) | *Adansonia digitata* L. (Malvaceae) | *Adansonia digitata* L. (Malvaceae) | *Acanthus montanus* (Nees) T.Anderson (Acanthaceae) |
| 5 | *Acanthospermum hispidum DC. (Asteraceae)* | *Adiantum aethiopicum L. (Pteridaceae)* | *Acanthus montanus* (Nees) T.Anderson (Acanthaceae) | *Aframomum melegueta* K. Schum. (Zingiberaceae) | *Afzelia africana* Sm. ex Pers. (Fabaceae) | Acmella caulirhiza Delile (Asteraceae) |
| 6 | *Acanthus montanus (Nees) T.Anderson (Acanthaceae)* | *Adiantum capillus-veneris L. (Pteridaceae)* | *Achyranthes aspera* L. (Amaranthaceae) | *Albizia chevalieri* Harms (Fabaceae) | *Aloe buettneri* A.Berger (Asphodelaceae) | *Adansonia digitata* L. (Malvaceae) |
| 7 | *Achillea millefolium L.(Asteraceae)* | *Adiantum caudatum L. (Pteridaceae)* | *Adansonia digitata* L. (Malvaceae) | *Albizia lebbeck* (L.) Benth. (Fabaceae) | *Andira inermis* (W.Wright) DC. (Fabaceae) | *Adenopus breviflorus* Benth. (Cucurbitaceae) |
| 8 | *Achyranthes aspera L. (Amaranthaceae)* | *Adiantum incisum Forssk. (Pteridaceae)* | *Adenostemma mauritianum* DC. (Asteraceae) | *Allium cepa* L. (Amaryllidaceae) | *Annona senegalensis* Pers. (Annonaceae) | *Aframomum melegueta* K. Schum. (Zingiberaceae) |
| 9 | *Acmella caulirhiza Delile (Asteraceae)* | *Adiantum venustum D.Don (Pteridaceae)* | *Aframomum melegueta* K. Schum. (Zingiberaceae) | *Allium sativum* L. (Amaryllidaceae) | *Anogeissus leiocarpa* (DC.) Guill. & Perr. (Annonaceae) | *Afzelia africana* Sm. ex Pers. (Fabaceae) |
| 10 | *Adansonia digitata L. (Malvaceae)* | *Aframomum melegueta K. Schum. (Zingiberaceae)* | *Afrofittonia silvestris* Lindau (Acanthaceae) | *Alstonia congensis* Engl. (Apocynaceae) | *Ansellia africana* Lindl. (Orchidaceae) | *Agelanthus dodoneifolius* (DC.) Polhill & Wiens (Loranthaceae) |
| 11 | *Adenia cissampeloides (Planch. ex Hook.) Harms (Passifloraceae)* | *Ageratum conyzoides L. (Asteraceae)* | *Ageratum conyzoides* L. (Asteraceae) | *Amaranthus caudatus* L. (Amaranthaceae) | *Aristolochia albida* Duch. (Aristolochiaceae) | *Ageratum conyzoides* L. (Asteraceae) |
| 12 | *Adenia lobata (Jacq.) Engl. (Passifloraceae)* | *Allamanda cathartica L. (Apocynaceae)* | *Albizia zygia* (DC.) J.F.Macbr. (Fabaceae) | *Ampelocissus africana var. africana* (Lour.) Merr. (Vitaceae) | *Aristolochia indica* L. (Aristolochiaceae) | *Alafia barteri* Oliv. (Apocynaceae) |
| 13 | *Aframomum daniellii (Hook.f.) K.Schum. (Zingiberaceae)* | *Allanblackia floribunda Oliv. (Clusiaceae)* | *Alcea rosea* L. (Malvaceae) | *Anacardium occidentale* L. (Anacardiaceae) | *Asparagus africanus* Lam. (Asparagaceae) | *Alchornea cordifolia* (Schumach. & Thonn.) Müll.Arg. (Euphorbiaceae) |
| 14 | *Aframomum melegueta K. Schum. (Zingiberaceae)* | *Allium cepa L. (Amaryllidaceae)* | *Alchornea cordifolia* (Schumach. & Thonn.) Müll.Arg. (Euphorbiaceae) | *Ananas comosus* (L.) Merr. (Bromeliaceae) | *Azadirachta indica* A. Juss. (Meliaceae) | *Allium ascalonicum* L. (Amaryllidaceae) |
| 15 | *Afromorus mesozygia (Stapf) E.M.Gardner (Moraceae)* | *Allium sativum L. (Amaryllidaceae)* | *Alchornea laxiflora* (Benth.) Pax & K.Hoffm. (Euphorbiaceae) | *Anchomanes difformis* (Blume) Engl. (Araceae) | *Balanites aegyptiaca* (L.) Delile (Zygophyllaceae) | *Allium cepa* L. (Amaryllidaceae) |
| 16 | *Afzelia africana Sm. ex Pers. (Fabaceae)* | *Aloe vera (L.) Burm.f. (Asphodelaceae)* | *Allamanda cathartica* L. (Apocynaceae) | *Andira inermis* (W.Wright) DC. (Fabaceae) | *Bambusa arundinacea* (Retz.) Willd. (Poaceae) | *Allium sativum* L. (Amaryllidaceae) |
| 17 | *Agelaea pentagyna (Lam.) Baill. (Connaraceae)* | *Alstonia boonei De Wild. (Apocynaceae)* | *Allanblackia floribunda* Oliv. (Clusiaceae) | *Andropogon gayanus* Kunth (Poaceae) | *Barleria prionitis* L. (Acanthaceae) | *Aloe buettneri* A.Berger (Asphodelaceae) |
| 18 | *Agelanthus brunneus (Engl.) Tiegh. (Loranthaceae)* | *Alstonia congensis Engl. (Apocynaceae)* | *Allium ascalonicum* L. (Amaryllidaceae) | *Anisopus mannii*N.E.Br. (Apocynaceae) | *Boswellia dalzielii* Hutch. (Burseraceae) | *Aloe schweinfurthii*Baker (Asphodelaceae) |
| 19 | *Ageratum conyzoides L. (Asteraceae)* | *Anacardium occidentale L. (Anacardiaceae)* | *Allium cepa* L. (Amaryllidaceae) | *Annickia chlorantha* (Oliv.) Setten & Maas (Annonaceae) | *Bridelia ferruginea* Benth. (Phyllanthaceae) | *Aloe vera* (L.) Burm.f. (Asphodelaceae) |
| 20 | *Alafia barteri Oliv. (Apocynaceae)* | *Ananas comosus (L.) Merr. (Bromeliaceae)* | *Allium sativum* L. (Amaryllidaceae) | *Annona senegalensis* Pers. (Annonaceae) | *Bridelia scleroneura* Müll.Arg. (Phyllanthaceae) | *Alstonia boonei* De Wild. (Apocynaceae) |
| 21 | *Albizia adianthifolia (Schumach.) W.Wight (Fabaceae)* | Annickia chlorantha (Oliv.) Setten & Maas (Annonaceae) | *Allium schoenoprasum* L. (Amaryllidaceae) | *Anogeissus leiocarpa* (DC.) Guill. & Perr. (Annonaceae) | *Calamus guruba* Buch.-Ham. ex Mart. (Arecaceae) | *Alstonia congensis* Engl. (Apocynaceae) |
| 22 | *Albizia ferruginea (Guill. & Perr.) Benth. (Fabaceae)* | *Annona muricata L. (Annonaceae)* | *Aloe vera* (L.) Burm.f. (Asphodelaceae) | *Argemone mexicana* L. (Papaveraceae) | *Carica papaya* L. (Caricaceae) | *Anacardium occidentale* L. (Anacardiaceae) |
| 23 | *Albizia glaberrima (Schumach. & Thonn.) Benth. (Fabaceae)* | *Anogeissus leiocarpa (DC.) Guill. & Perr. (Annonaceae)* | *Alstonia boonei* De Wild. (Apocynaceae) | *Aristolochia albida* Duch. (Aristolochiaceae) | *Carissa spinarum* L. (Apocynaceae) | *Ananas comosus* (L.) Merr. (Bromeliaceae) |
| 24 | *Albizia lebbeck (L.) Benth. (Fabaceae)* | *Anthocleista djalonensis A.Chev. (Gentianaceae)* | *Alternanthera sessilis* (L.) R.Br. ex DC. (Amaranthaceae) | *Aristolochia ringens* Vahl (Aristolochiaceae) | *Cassia arereh* Delile (Fabaceae) | *Ananas comosus* (L.) Merr. (Bromeliaceae) |
| 25 | *Albizia zygia (DC.) J.F.Macbr. (Fabaceae)* | *Anthocleista vogelii Planch. (Gentianaceae)* | *Amaranthus viridis* L. (Amaranthaceae) | *Artemisia absinthium* L. (Asteraceae) | *Cassia sieberiana* DC. (Fabaceae) | *Anchomanes difformis* (Blume) Engl. (Araceae) |
| 26 | *Alchornea cordifolia (Schumach. & Thonn.) Müll.Arg. (Euphorbiaceae)* | *Argemone mexicana L. (Papaveraceae)* | *Anacardium occidentale* L. (Anacardiaceae) | *Artemisia annua*L. (Asteraceae) | *Cissampelos mucronata* A.Rich. (Menispermaceae) | *Anisopus mannii*N.E.Br. (Apocynaceae) |
| 27 | *Alchornea laxiflora (Benth.) Pax & K.Hoffm. (Euphorbiaceae)* | *Aspilia africana (Pers.) C.D.Adams (Asteraceae)* | *Ananas comosus* (L.) Merr. (Bromeliaceae) | *Asclepias tuberosa* L. (Apocynaceae) | *Cissus quadrangularis* L. (Vitaceae) | *Annickia chlorantha* (Oliv.) Setten & Maas (Annonaceae) |
| 28 | *Alectra sessiliflora (Vahl) Kuntze (Orobanchaceae)* | *Asplenium adiantum-nigrum L. (Aspleniaceae)* | *Anchomanes difformis* (Blume) Engl. (Araceae) | *Asparagus africanus* Lam. (Asparagaceae) | *Citrus sinensis* (L.) Osbeck (Rutaceae) | *Annona muricata* L. (Annonaceae) |
| 29 | *Allamanda cathartica L. (Apocynaceae)* | *Asplenium bulbiferum G.Forst. (Aspleniaceae)* | *Anethum graveolens* L. (Apiaceae) | *Azadirachta indica* A. Juss. (Meliaceae) | *Citrus × aurantiifolia (Christm.) Swingle (Rutaceae)* | *Annona senegalensis* Pers. (Annonaceae) |
| 30 | *Allanblackia floribunda Oliv. (Clusiaceae)* | *Axonopus compressus (Sw.) P.Beauv. (Poaceae)* | *Annickia chlorantha* (Oliv.) Setten & Maas (Annonaceae) | *Baccharoides adoensis var. kotschyana* (Sch.Bip. ex Walp.) Isawumi, El-Ghazaly & B.Nord. (Asteraceae) | *Combretum molle* R.Br. ex G.Don (Combretaceae) | *Annona squamosa* L. (Annonaceae) |
| 31 | *Allium ascalonicum L. (Amaryllidaceae)* | *Azadirachta indica A. Juss. (Meliaceae)* | *Annona muricata* L. (Annonaceae) | *Balanites aegyptiaca* (L.) Delile (Zygophyllaceae) | *Commiphora africana var africana* (A. Rich.) Engl. (Burseraceae) | *Anogeissus leiocarpa* (DC.) Guill. & Perr. (Annonaceae) |
| 32 | *Allium cepa L. (Amaryllidaceae)* | *Balanites aegyptiaca (L.) Delile (Zygophyllaceae)* | *Anogeissus leiocarpa* (DC.) Guill. & Perr. (Annonaceae) | *Bauhinia rufescens* Lam. (Fabaceae) | *Commiphora kerstingii* Engl. (Burseraceae) | *Antidesma venosum*E.Mey. ex Tul. (Phyllanthaceae) |
| 33 | *Allium fistulosum L. (Amaryllidaceae)* | *Bambusa vulgaris Schrad. (Poaceae)* | *Anthocleista djalonensis* A.Chev. (Gentianaceae) | *Bobgunnia madagascariensis* (Desv.) J.H.Kirkbr. & Wiersema (Fabaceae) | *Curculigo pilosa*(Schumach. & Thonn.) Engl. (Hypoxidaceae) | *Arachis hypogaea* L. (Fabaceae) |
| 34 | *Allium sativum L. (Amaryllidaceae)* | *Baphia nitida G.Lodd. (Fabaceae)* | *Anthonotha macrophylla* P.Beauv. (Fabaceae) | *Boerhavia diffusa* L. (Nyctaginaceae) | *Cymbopogon citratus* (DC.) Stapf (Poaceae) | *Argemone mexicana* L. (Papaveraceae) |
| 35 | *Allophylus africanus P.Beauv. (Sapindaceae)* | *Berlinia grandiflora (Vahl) Hutch. & Dalziel (Fabaceae)* | *Argemone mexicana* L. (Papaveraceae) | *Bombax costatum* Pellegr. & Vuillet (Malvaceae) | *Dalbergia melanoxylon* Guill. & Perr. (Fabaceae) | *Aristolochia ringens* Vahl (Aristolochiaceae) |
| 36 | *Aloe arborescens Mill. (Asphodelaceae)* | *Blighia sapida K.D.Koenig (Sapindaceae)* | *Artemisia dracunculus* L. (Asteraceae) | *Borassus flabellifer* L. (Arecaceae) | *Daucus carota*L. (Apiaceae) | *Asclepias syriaca* L. (Apocynaceae) |
| 37 | *Aloe schweinfurthii Baker (Asphodelaceae)* | *Blumea perrottetiana DC. (Asteraceae)* | *Artocarpus altilis* (Parkinson) Fosberg (Moraceae) | *Boscia salicifolia* Oliv. (Capparaceae) | *Detarium microcarpum* Guill. & Perr. (Fabaceae) | *Asparagus africanus* Lam. (Asparagaceae) |
| 38 | *Aloe vera (L.) Burm.f. (Asphodelaceae)* | *Botrychium lanuginosum Wall. ex Hook. & Grev. (Ophioglossaceae)* | *Aspilia africana* (Pers.) C.D.Adams (Asteraceae) | *Boscia senegalensis* Lam. (Capparaceae) | *Dichrostachys cinerea* (L.) Wight & Arn. (Fabaceae) | *Aspilia africana* (Pers.) C.D.Adams (Asteraceae) |
| 39 | *Alstonia boonei De Wild. (Apocynaceae)* | *Buchholzia coriacea Engl. (Capparaceae)* | *Astraea lobata* (L.) Klotzsch (Euphorbiaceae) | *Boswellia dalzielii* Hutch. (Burseraceae) | *Eucalyptus camaldulensis* Dehn. (Myrtaceae) | *Azadirachta indica* A. Juss. (Meliaceae) |
| 40 | *Alstonia congensis Engl. (Apocynaceae)* | *Burkea africana Hook. (Fabaceae)* | *Axonopus compressus* (Sw.) P.Beauv. (Poaceae) | *Boswellia odorata* Hutch. (Burseraceae) | *Euphorbia lateriflora* Schumach. (Euphorbiaceae) | *Baccharoides guineensis*(Benth.) H.Rob. (Asteraceae) |
| 41 | *Alternanthera brasiliana (L.) Kuntze (Amaranthaceae)* | *Cactus opuntia var. inermis DC. (Cactaceae)* | *Azadirachta indica* A. Juss. (Meliaceae) | *Breonadia salicina* (Vahl) Hepper & J.R.I.Wood (Rubiaceae) | *Euphorbia poissonii* Pax (Euphorbiaceae) | *Bambusa vulgaris* Schrad. (Poaceae) |
| 42 | *Alternanthera pungens Kunth (Amaranthaceae)* | *Caesalpinia pulcherrima (L.) Sw. (Fabaceae)* | *Balanites aegyptiaca* (L.) Delile (Zygophyllaceae) | *Bridelia ferruginea* Benth. (Phyllanthaceae) | *Euphorbia unispina* N.E.Br. (Euphorbiaceae) | *Bauhinia rufescens* Lam. (Fabaceae) |
| 43 | *Alternanthera sessilis (L.) R.Br. ex DC. (Amaranthaceae)* | *Cajanus cajan (L.) Millsp. (Fabaceae)* | *Bambusa vulgaris* Schrad. (Poaceae) | *Cadaba farinosa* Forssk. (Capparaceae) | *Faidherbia albida* (Delile) A.Chev. (Fabaceae) | *Bidens pilosa* L. (Asteraceae) |
| 44 | *Amaranthus cruentus L. (Amaranthaceae)* | *Canna indica L. (Cannaceae)* | *Baphia nitida* G.Lodd. (Fabaceae) | *Cajanus cajan* (L.) Millsp. (Fabaceae) | *Ficus platyphylla* Delile (Moraceae) | *Bombax ceiba* L. (Malvaceae) |
| 45 | *Amaranthus spinosus L. (Amaranthaceae)* | *Capsicum frutescens L. (Solanaceae)* | *Basella alba* L. (Basellaceae) | *Calotropis procera* (Aiton) Dryand (Asclepiadaceae) | *Ficus sur* Forssk. (Moraceae) | *Boswellia dalzielii* Hutch. (Burseraceae) |
| 46 | *Amaranthus viridis L. (Amaranthaceae)* | *Carica papaya L. (Caricaceae)* | *Berlinia grandiflora* (Vahl) Hutch. & Dalziel (Fabaceae) | *Caralluma dalzielii* N.E.Br. (Apocynaceae) | *Ficus sycomorus*  L. (Moraceae) | *Breonadia salicina* (Vahl) Hepper & J.R.I.Wood (Rubiaceae) |
| 47 | *Amblygonocarpus andongensis (Welw. ex Oliv.) Exell & Torre (Fabaceae)* | *Cassia fistula L. (Fabaceae)* | *Bixa orellana* L. (Bixaceae) | *Carica papaya* L. (Caricaceae) | *Ficus thonningii* Blume (Moraceae) | *Bridelia exaltata* F.Muell. (Phyllanthaceae) |
| 48 | *Ananas comosus (L.) Merr. (Bromeliaceae)* | *Ceiba pentandra (L.) Gaertn. (Malvaceae)* | *Blighia sapida* K.D.Koenig (Sapindaceae) | *Cassia arereh* Delile (Fabaceae) | *Gardenia aqualla* Stapf & Hutch. (Rubiaceae) | *Bridelia ferruginea* Benth. (Phyllanthaceae) |
| 49 | *Anchomanes difformis (Blume) Engl. (Araceae)* | *Chasmanthera dependens Hochst. (Menispermaceae)* | *Blumea perrottetiana* DC. (Asteraceae) | *Cassia sieberiana* DC. (Fabaceae) | *Gossypium barbadense* L. (Malvaceae) | *Bryophyllum pinnatum* (Lam.) Oken (Crassulaceae) |
| 50 | *Andira inermis (W.Wright) DC. (Fabaceae)* | *Chromolaena odorata (L.) R.M.King & H.Rob. (Asteraceae)* | *Boerhavia diffusa* L. (Nyctaginaceae) | *Ceiba pentandra* (L.) Gaertn. (Malvaceae) | *Guiera senegalensis* J.F.Gmel. (Combretaceae) | *Burkea africana* Hook. (Fabaceae) |
| 51 | *Andrographis paniculata (Burm.f.) Wall. ex Nees (Acanthaceae)* | *Cinchona pubescens Vahl (Rubiaceae)* | *Bryophyllum pinnatum* (Lam.) Oken (Crassulaceae) | *Celosia trigyna* L. (Amaranthaceae) | *Gymnosporia senegalensis* (Lam.) Loes. (Celastraceae) | *Calliandra haematocephala* Hassk. (Fabaceae) |
| 52 | *Aneilema umbrosum (Vahl) Kunth (Commelinaceae)* | *Citrullus lanatus (Thunb.) Matsum. & Nakai (Cucurbitaceae)* | *Burkea africana* Hook. (Fabaceae) | *Cenchrus americanus* (L.) Morrone (Poaceae) | *Habenaria zambesina* Rchb.f. (Orchidaceae) | *Calotropis procera* (Aiton) Dryand (Asclepiadaceae) |
| 53 | *Angraecum eichlerianum Kraenzl. (Orchidaceae)* | *Citrus ×aurantium L. (Rutaceae)* | *Cactus opuntia var. inermis*DC*.* (Cactaceae) | *Cenchrus pedicellatus* (Trin.) Morrone (Poaceae) | *Hibiscus sabdariffa* L. (Malvaceae) | *Canarium schweinfurthii*Engl. (Burseraceae) |
| 54 | *Angylocalyx oligophyllus (Baker) Baker f. (Fabaceae)* | *Citrus medica var. limon L. (Rutaceae)* | *Caesalpinia pulcherrima* (L.) Sw. (Fabaceae) | *Centaurea alba subsp. subciliaris* (Boiss. & Heldr.) Dostál (Asteraceae) | *Hygrophila auriculata* (Schumach.) Heine (Acanthaceae) | *Capparis brassii* DC. (Capparaceae) |
| 56 | *Annickia chlorantha (Oliv.) Setten & Maas (Annonaceae)* | *Citrus sinensis (L.) Osbeck (Rutaceae)* | *Cajanus cajan* (L.) Millsp. (Fabaceae) | *Centaurea perrottettii* DC. (Asteraceae) | *Ipomoea mauritiana* Jacq. (Convolvulaceae) | *Capsicum frutescens* L. (Solanaceae) |
| 57 | *Annona muricata L. (Annonaceae)* | *Citrus × aurantiifolia (Christm.) Swingle (Rutaceae)* | *Caladium bicolor* (Aiton) Vent. (Araceae) | *Centaurea praecox* Oliv. & Hiern (Asteraceae) | *Jatropha curcas* L. (Euphorbiaceae) | *Carica papaya* L.(Caricaceae) |
| 58 | *Annona senegalensis Pers. (Annonaceae)* | *Citrus × paradisi Macfad. (Rutaceae)* | *Calendula officinalis*L. (Asteraceae) | *Chrozophora senegalensis* (Lam.) Spreng. (Euphorbiaceae) | *Khaya senegalensis* (Desr.) A.Juss.) (Meliaceae) | *Carissa spinarum* L. (Apocynaceae) |
| 59 | *Annona squamosa L. (Annonaceae)* | *Clausena anisata (Willd.) Hook.f. ex Benth. (Rutaceae)* | *Caloncoba echinata* (Oliv.) Gilg (Achariaceae) | *Cinnamomum verum* J.Presl (Lauraceae) | *Lawsonia inermis* L. (Lythraceae) | *Cassia fistula* L. (Fabaceae) |
| 60 | *Anogeissus leiocarpa (DC.) Guill. & Perr. (Annonaceae)* | *Cleistopholis patens (Benth.) Engl. & Diels (Annonaceae)* | *Canna indica* L. (Cannaceae) | *Cissus populnea* Guill. & Perr. (Vitaceae) | *Leptadenia lanceolata* (Poir.) Goyder (Apocynaceae) | *Cassytha filiformis* L. (Lauraceae) |
| 61 | *Anthocleista djalonensis A.Chev. (Gentianaceae)* | *Clerodendrum paniculatum L. (Lamiaceae)* | *Capsicum frutescens* L. (Solanaceae) | *Cissus populnea* Guill. & Perr. (Vitaceae) | *Lophira lanceolata* Tiegh. ex Keay (Ochnaceae) | *Ceiba pentandra* (L.) Gaertn. (Malvaceae) |
| 62 | *Anthocleista liebrechtsiana De Wild. & T.Durand (Gentianaceae)* | *Cochlospermum tinctorium Perrier ex A.Rich. (Bixaceae)* | *Carapa procera*DC. (Meliaceae) | *Citrullus lanatus* (Thunb.) Matsum. & Nakai (Cucurbitaceae) | *Maerua angolensis*DC. (Capparaceae) | *Celastrus paniculatus* Willd. (Celastraceae) |
| 63 | *Anthocleista nobilis G.Don (Gentianaceae)* | *Cocos nucifera L. (Arecaceae)* | *Carica papaya* L. (Caricaceae) | *Citrus ×aurantium* L. (Rutaceae) | *Mangifera indica* L. ( Anacardiaceae) | *Cenchrus americanus* (L.) Morrone (Poaceae) |
| 64 | *Anthocleista vogelii Planch. (Gentianaceae)* | *Cola acuminata (P.Beauv.) Schott & Endl. (Malvaceae)* | *Carpolobia lutea* G.Don (Polygalaceae) | *Citrus medica* var. limon L. (Rutaceae) | *Maranthes polyandra* (Benth.) Prance (Chrysobalanaceae) | *Chasmanthera dependens* Hochst. (Menispermaceae) |
| 65 | *Antiaris toxicaria Lesch. (Moraceae)* | *Coniogramme serrulata (Blume) Fée (Pteridaceae)* | *Cassia fistula L.* (Fabaceae) | *Citrus sinensis* (L.) Osbeck (Rutaceae) | *Mitracarpus hirtus*(L.) DC. (Rubiaceae) | *Chloris pilosa* Schumach. (Poaceae) |
| 66 | *Apodostigma pallens (Planch. ex Oliv.) R.Wilczek (Celastraceae)* | *Corchorus olitorius L. (Malvaceae)* | *Cassytha filiformis* L. (Lauraceae) | *Citrus × aurantiifolia*(Christm.) Swingle (Rutaceae) | *Moringa oleifera* Lam. (Moringaceae) | *Cissus populnea* Guill. & Perr. (Vitaceae) |
| 67 | *Arachis hypogaea L. (Fabaceae)* | *Cryptolepis nigrescens (Wennberg) L.Joubert & Bruyns (Apocynaceae)* | *Ceiba pentandra* (L.) Gaertn. (Malvaceae) | *Citrus × paradisi*Macfad. (Rutaceae) | *Nymphaea lotus*L. (Nymphaeaceae) | *Citrullus colocynthis* (L.) Schrad. (Cucurbitaceae) |
| 68 | *Argemone mexicana L. (Papaveraceae)* | *Cryptolepis sanguinolenta (Lindl.) Schltr. (Apocynaceae)* | *Chamaecrista mimosoides*(L.) Greene (Fabaceae) | *Cleome gynandra* L. (Cleomaceae) | *Parkia biglobosa* (Jacq.) R.Br. ex G.Don (Fabaceae) | *Citrullus lanatus* (Thunb.) Matsum. & Nakai (Cucurbitaceae) |
| 69 | *Argyreia nervosa (Burm.f.) Bojer (Convolvulaceae)* | *Cymbopogon citratus (DC.) Stapf (Poaceae)* | *Chasmanthera dependens* Hochst. (Menispermaceae) | *Clerodendrum capitatum*(Willd.) Schumach. (Lamiaceae) | *Pericopsis laxiflora* (Benth. ex Baker) Meeuwen (Fabaceae) | *Citrus medica* var. limon L. (Rutaceae) |
| 70 | *Aristolochia albida Duch. (Aristolochiaceae)* | *Cynodon dactylon (L.) Pers. (Poaceae)* | *Chromolaena odorata* (L.) R.M.King & H.Rob. (Asteraceae) | *Cochlospermum planchonii* Hook.fil. ex Planch. (Cochlospermaceae) | *Piliostigma reticulatum* (DC.) Hochst. (Fabaceae) | *Citrus sinensis* (L.) Osbeck (Rutaceae) |
| 71 | *Aristolochia erecta L. (Aristolochiaceae)* | *Dicranopteris linearis (Burm.f.) Underw. (Gleicheniaceae)* | *Cinchona pubescens*Vahl (Rubiaceae) | *Cochlospermum tinctorium* Perrier ex A.Rich. (Bixaceae) | *Prosopis africana* (Guill. & Perr.) Taub. (Fabaceae) | *Citrus × aurantiifolia*(Christm.) Swingle |
| 72 | *Aristolochia indica L. (Aristolochiaceae)* | *Dioscorea dumetorum (Kunth) Pax (Dioscoreaceae)* | *Cissus quadrangularis* L. (Vitaceae) | *Cocos nucifera* L. (Arecaceae) | *Pseudocedrela kotschyi* Harms (Meliaceae) | *Citrus × aurantiifolia*(Christm.) Swingle (Rutaceae) |
| 73 | *Aristolochia ringens Vahl (Aristolochiaceae)* | *Diplazium esculentum (Retz.) Sw. (Aspleniaceae)* | *Citrullus colocynthis* (L.) Schrad. (Cucurbitaceae) | Cola nitida (Vent.) Schott & Endl. (Malvaceae) | *Psidium guajava* L. (Myrtaceae) | *Clausena anisat*a (Willd.) Hook.f. ex Benth. (Rutaceae) |
| 74 | *Artocarpus altilis (Parkinson) Fosberg (Moraceae)* | *Dryopteris filix-mas (L.) Schott (Polypodiaceae)* | *Citrullus lanatus* (Thunb.) Matsum. & Nakai (Cucurbitaceae) | *Combretum camporum*Engl. (Combretaceae) | *Pterocarpus erinaceus* Poir. (Fabaceae) | *Cleome viscosa* L. (Cleomaceae) |
| 75 | *Asclepias curassavica L. (Apocynaceae)* | *Dryopteris wallichiana (Spreng.) Hyl. (Polypodiaceae)* | *Citrus ×aurantium* L. (Rutaceae) | *Combretum glutinosum* Perr. ex DC. (Combretaceae) | *Sclerocarya birrea* (A.Rich.) Hochst. (Anacardiaceae) | *Clerodendrum capitatum*(Willd.) Schumach. (Lamiaceae) |
| 76 | *Asparagus africanus Lam. (Asparagaceae)* | *Dysphania ambrosioides (L.) Mosyakin & Clemants (Amaranthaceae)* | *Citrus medica* var. limon L. (Rutaceae) | *Combretum micranthum* G. Don (Combretaceae) | *Securidaca longipedunculata* Fresen. (Polygalaceae) | *Cnestis ferruginea* Vahl ex DC. (Connaraceae) |
| 77 | *Aspilia africana (Pers.) C.D.Adams (Asteraceae)* | *Emilia sonchifolia (L.) DC. (Asteraceae)* | *Citrus sinensis* (L.) Osbeck (Rutaceae) | *Combretum molle* R.Br. ex G.Don (Combretaceae) | *Senegalia polyacantha* (Willd.) Seigler & Ebinger (Fabaceae) | *Cocos nucifera* L. (Arecaceae) |
| 78 | *Astraea lobata (L.) Klotzsch (Euphorbiaceae)* | *Equisetum diffusum D.Don (Equisetaceae)* | *Citrus × aurantiifolia*(Christm.) Swingle (Rutaceae) | *Combretum nigricans* Lepr. ex Guill. & Perr. (Combretaceae) | *Senna alata* (L.) Roxb. (Fabaceae) | *Combretum bracteatum* (M.A.Lawson) Engl. & Diels. |
| 79 | *Asystasia gangetica (L.) T.Anderson (Acanthaceae)* | *Erythrina senegalensis DC. (Fabaceae)* | *Citrus × paradisi*Macfad. (Rutaceae) | *Combretum nioroense* Aubrév. ex Keay (Combretaceae) | *Senna italica* Mill. (Fabaceae) | *Combretum collinum subsp. geitonophyllum* (Diels) Okafa (Combretaceae) |
| 80 | *Avicennia germinans (L.) L. (Acanthaceae)* | *Eucalyptus camaldulensis Dehn. (Myrtaceae)* | *Clausena anisat*a (Willd.) Hook.f. ex Benth. (Rutaceae) | *Combretum sericeum* G.Don (Combretaceae) | *Senna siamea* (Lam.) H.S.Irwin & Barneby (Fabaceae) | *Combretum glutinosum* Perr. ex DC. (Combretaceae) |
| 81 | *Axonopus compressus (Sw.) P.Beauv. (Poaceae)* | *Ficus capensis Thunb. (Moraceae)* | *Cleistopholis patens* (Benth.) Engl. & Diels (Annonaceae) | *Combretum tomentosum* G.Don (Combretaceae) | *Senna singueana (Delile) Lock (Fabaceae)* | *Combretum micranthum* G. Don (Combretaceae) |
| 82 | *Azadirachta indica A. Juss. (Meliaceae)* | *Ficus exasperata Vahl (Moraceae)* | *Cleome viscosa* L. (Cleomaceae) | *Commiphora africana var africana* (A. Rich.) Engl. (Burseraceae) | *Senna tora (L.) Roxb. (Fabaceae)* | *Combretum molle* R.Br. ex G.Don (Combretaceae) |
| 83 | *Azolla filiculoides Lam. (Salviniaceae)* | *Funtumia africana (Benth.) Stapf (Apocynaceae)* | *Clerodendrum paniculatum* L. (Lamiaceae) | *Commiphora hildebrandtii*Engl. (Burseraceae) | *Spinacia oleracea* L. (Amaranthaceae) | *Combretum nigricans* Lepr. ex Guill. & Perr. (Combretaceae) |
| 84 | *Bacopa floribunda (R.Br.) Wettst. (Plantaginaceae)* | *Gambeya albida (G.Don) Aubrév. & Pellegr. (Sapotaceae)* | *Cnestis ferruginea* Vahl ex DC. (Connaraceae) | *Commiphora kerstingii* Engl. (Burseraceae) | *Steganotaenia araliacea* Hochst. (Apiaceae) | *Commiphora africana var africana* (A. Rich.) Engl. (Burseraceae) |
| 85 | *Bakerella poissonii (Lecomte) Balle (Loranthaceae)* | *Garcinia kola Heckel (Clusiaceae)* | *Cochlospermum tinctorium* Perrier ex A.Rich. (Bixaceae) | *Corchorus olitorius* L. (Malvaceae) | *Sterculia setigera*Delile (Malvaceae) | *Crateva adansonii* DC. (Capparaceae) |
| 86 | *Balanites aegyptiaca (L.) Delile (Zygophyllaceae)* | *Glyphaea brevis (Biehler) Monach. (Malvaceae)* | *Cocos nucifera* L. (Arecaceae) | *Cordia africana* Lam. (Boraginaceae) | *Strychnos innocua* Delile (Loganiaceae) | *Crinum jagus* (J.Thomps.) Dandy (Amaryllidaceae |
| 87 | *Bambusa vulgaris Schrad. (Poaceae)* | *Gmelina arborea Roxb. ex Sm. (Lamiaceae)* | *Cola acuminata* (P.Beauv.) Schott & Endl. (Malvaceae) | *Crescentia cujete* L. (Bignoniaceae) | *Strychnos spinosa* Lam. (Loganiaceae) | *Crossopteryx febrifuga* (Afzel. ex G.Don) Benth. (Rubiaceae) |
| 88 | *Baphia nitida G.Lodd. (Fabaceae)* | *Goniophlebium fieldingianum (Kunze ex Mett.) T.Moore (Polypodiaceae)* | *Cola millenii*K.Schum. (Malvaceae) | *Cucumis pustulatus* Naudin ex Hook.f. (Cucurbitaceae) | *Syzygium aromaticum* (L.) Merr. & L.M.Perry (Myrtaceae) | *Croton zambesicus* Müll.Arg. (Euphorbiaceae) |
| 89 | *Barleria buxifolia L. (Acanthaceae)* | *Gossypium barbadense L. (Malvaceae)* | *Cola nitida* (Vent.) Schott & Endl. (Malvaceae) | *Cucurbita maxima* Duchesne (Cucurbitaceae) | *Tamarindus indica* L. (Fabaceae) | *Cryptolepis nigrescens* (Wennberg) L.Joubert & Bruyns (Apocynaceae) |
| 90 | *Barleria opaca (Vahl) Nees (Acanthaceae)* | *Gossypium hirsutum L. (Malvaceae)* | *Colocasia esculenta* (L.) Schott (Araceae) | *Cucurbita pepo* L. (Cucurbitaceae) | *Trianthema portulacastrum* L. (Aizoaceae) | *Cucumis metuliferus* Jacques (Cucurbitaceae) |
| 91 | *Basella alba L. (Basellaceae)* | *Harungana madagascariensis Lam. ex Poir. (Hypericaceae)* | *Combretum racemosum* P.Beauv. (Combretaceae) | *Curcuma longa* L. (Zingiberaceae) | *Vachellia nilotica (L.) P.J.H.Hurter & Mabb.* (Fabaceae) | *Curcuma longa* L. (Zingiberaceae) |
| 92 | *Berlinia grandiflora (Vahl) Hutch. & Dalziel (Fabaceae)* | *Heliotropium indicum L. (Boraginaceae)* | *Corchorus olitorius* L. (Malvaceae) | *Cymbopogon citratus* (DC.) Stapf (Poaceae) | *Vernonia amygdalina* Delile (Asteraceae) | *Cussonia arborea* Hochst. ex A.Rich. (Araliaceae) |
| 93 | *Bidens bipinnata L. (Asteraceae)* | *Hemionitis albomarginata (C.B.Clarke) Christenh. (Pteridaceae)* | *Corylus avellana* L. (Betulaceae) | *Cynodon dactylon* (L.) Pers. (Poaceae) | *Vitellaria paradoxa* C.F Gaertn (Sapotaceae) | *Cymbopogon citratus* (DC.) Stapf (Poaceae) |
| 94 | *Bidens pilosa L. (Asteraceae)* | *Hemionitis feei (T.Moore) Christenh. (Pteridaceae)* | *Corynanthe johimbe* K.Schum. (Rubiaceae) | *Cyperus articulatus* L. (Cyperaceae) | *Vitex doniana* sweet (Lamiaceae) | *Daniellia oliveri* (Rolfe) Hutch. & Dalziel (Fabaceae) |
| 95 | *Bixa orellana L. (Bixaceae)* | *Hibiscus × rosa-sinensis L. (Malvaceae)* | *Costus afer* Ker Gawl. (Costaceae) | *Cyperus difformis* L. (Cyperaceae) | *Vitex madiensis subsp. Madiensis (Lamiaceae)* | *Datura stramonium* L. (Solanaceae) |
| 96 | *Blighia sapida K.D.Koenig (Sapindaceae)* | *Huperzia selago (L.) Bernh. ex Schrank & Mart. (Lycopodiaceae)* | *Costus lucanusianus* J.Braun & K.Schum. (Costaceae) | *Daniellia oliveri* (Rolfe) Hutch. & Dalziel (Fabaceae) | *Waltheria indica* L. (Malvaceae) | *Desmodium scorpiurus* (Sw.) Poir. (Fabaceae) |
| 97 | *Blumea perrottetiana DC. (Asteraceae)* | *Icacina trichantha Oliv. (Icacinaceae)* | *Crassocephalum crepidioides* (Benth.) S.Moore (Asteraceae) | *Daucus carota*L. (Apiaceae) | *Ximenia americana* L. (Olacaceae) | *Detarium microcarpum* Guill. & Perr. (Fabaceae) |
| 98 | *Boerhavia diffusa L. (Nyctaginaceae)* | *Irvingia gabonensis (Aubry-Lecomte ex O'Rorke) Baill. (Irvingiaceae)* | *Crateva adansonii* DC. (Capparaceae) | *Detarium microcarpum* Guill. & Perr. (Fabaceae) | *Ziziphus mauritiana* Lam. (Rhamnaceae) | *Detarium senegalense* J.F.Gmel. (Fabaceae) |
| 99 | *Bombax buonopozense Beauverd (Malvaceae)* | *Jatropha curcas L. (Euphorbiaceae)* | *Croton zambesicus*Müll.Arg. (Euphorbiaceae) | *Detarium senegalense* J.F.Gmel. (Fabaceae) | *Ziziphus spina-christi* (L.) Desf. (Rhamnaceae) | *Dichrostachys cinerea* (L.) Wight & Arn. (Fabaceae) |
| 100 | *Brachystegia nigerica Hoyle & A.P.D.Jones (Fabaceae)* | *Jatropha gossypifolia L. (Euphorbiaceae)* | *Cryptolepis nigrescens* (Wennberg) L.Joubert & Bruyns (Apocynaceae) | *Dialium guineense* Willd. (Fabaceae) |  | *Dioscorea abyssinica* Hochst. ex Kunth (Dioscoreaceae) |
| 101 | *Bridelia atroviridis Müll.Arg. (Phyllanthaceae)* | *Khaya grandifoliola C.DC. (Meliaceae)* | *Cryptolepis sanguinolenta* (Lindl.) Schltr. (Apocynaceae) | *Dichrostachys cinerea* (L.) Wight & Arn. (Fabaceae) |  | *Distimake dissectus* (Jacq.) A.R.Simões & Staples (Convolvulaceae) |
| 102 | *Bridelia ferruginea Benth. (Phyllanthaceae)* | *Khaya ivorensis A.Chev. (Meliaceae)* | *Cucurbita maxima* Duchesne (Cucurbitaceae) | *Dicliptera paniculata* (Forssk.) I.Darbysh. (Acanthaceae) | | *Dracaena perrottetii* Baker (Asparagaceae) |
| 103 | *Bridelia micrantha (Hochst.) Baill. (Hochst.) (Phyllanthaceae)* | *Kigelia africana (Lam.) Benth. (Bignoniaceae)* | *Curcuma longa* L. (Zingiberaceae) | *Dioscorea alata*L. (Dioscoreaceae) |  | *Dysphania ambrosioides (L.) Mosyakin & Clemants (Amaranthaceae)* |
| 104 | *Bryophyllum pinnatum (Lam.) Oken (Crassulaceae)* | *Lantana camara L. (Verbenaceae)* | *Cyathula prostrata* (L.) Blume (Amaranthaceae) | *Dioscorea bulbifera* L. (Dioscoreaceae) |  | *Echeveria agavoides* Lem. (Crassulaceae) |
| 105 | *Burkea africana Hook. (Fabaceae)* | *Laportea aestuans (L.) Chew (Urticaceae)* | *Cydonia oblonga* Mill. (Rosaceae) | *Diospyros macrocarpa* Hiern (Ebenaceae) |  | *Eclipta alba* (L.) Hassk. (Asteraceae) |
| 106 | *Cactus opuntia var. inermis DC. (Cactaceae)* | *Lawsonia inermis L. (Lythraceae)* | *Cylicodiscus gabunensis* Harms (Fabaceae) | *Diospyros mespiliformis* Hochst. ex A.DC. (Ebenaceae) | | *Elaeis guineensis* Jacq. (Arecaceae) |
| 107 | *Cadaba fruticosa (L.) Druce (Capparaceae)* | *Lippia multiflora Moldenke (Verbenaceae)* | *Cymbopogon citratus* (DC.) Stapf (Poaceae) | *Diospyros mespiliformis* Hochst. ex A.DC. (Ebenaceae) | | *Eleusine indica* (L.) Gaertn. (Poaceae) |
| 108 | *Caesalpinia pulcherrima (L.) Sw. (Fabaceae)* | *Lophira alata Banks ex C.F.Gaertn. (Ochnaceae)* | *Daniellia ogea*(Harms) Rolfe ex Holland (Fabaceae) | *Distemonanthus benthamianus* Baill. (Fabaceae) |  | *Entandrophragma utile* (Dawe & Sprague) Sprague (Meliaceae) |
| 109 | *Cajanus cajan (L.) Millsp. (Fabaceae)* | *Ludwigia hyssopifolia (G.Don) Exell (Onagraceae)* | *Daucus carota*L. (Apiaceae) | *Dyschoriste nagchana*(Nees) Bennet (Acanthaceae) |  | *Eragrostis cilianensis* (All.) Vignolo ex Janch. (Poaceae) |
| 110 | *Caladium bicolor (Aiton) Vent. (Araceae)* | *Lycopodium clavatum L. (Lycopodiaceae)* | *Dioscorea cayenensis subsp. rotundata* (Poir.) J.Miège (Dioscoreaceae) | *Echinochloa pyramidalis* (Lam.) Hitchc. & Chase (Poaceae) | | *Eremophila oldfieldii* F.Muell. (Scrophulariaceae) |
| 111 | *Calendula officinalis L. (Asteraceae)* | *Lygodium flexuosum (L.) Sw. (Schizaeaceae)* | *Dioscorea dumetorum* (Kunth) Pax (Dioscoreaceae) | *Echinochloa stagnina* (Retz.) P.Beauv. (Poaceae) |  | *Erythrina senegalensis* DC. (Fabaceae) |
| 112 | *Calliandra haematocephala Hassk. (Fabaceae)* | *Lygodium palmatum (Bernh.) Sw. (Schizaeaceae)* | *Dioscorea villosa*L. (Dioscoreaceae) | *Elaeis guineensis* Jacq. (Arecaceae) |  | *Erythrophleum africanum* (Benth.) Harms (Fabaceae) |
| 113 | *Callichilia barteri (Hook.f.) Stapf (Apocynaceae)* | *Mallotus oppositifolius (Geiseler) Müll.Arg. (Euphorbiaceae)* | *Distemonanthus benthamianus* Baill. (Fabaceae) | *Eleusine coracana* (L.) Gaertn. (Poaceae) |  | *Eucalyptus camaldulensis* Dehn. (Myrtaceae) |
| 114 | *Caloncoba glauca (P.Beauv.) Gilg (Achariaceae)* | *Mangifera indica L. ( Anacardiaceae)* | *Dracaena arborea* (Willd.) Link (Asparagaceae) | *Eleusine indica* (L.) Gaertn. (Poaceae) |  | *Eucalyptus tereticornis* Sm. (Myrtaceae) |
| 115 | *Calophyllum inophyllum L. (Calophyllaceae)* | *Manihot esculenta Crantz (Euphorbiaceae)* | *Dracocephalum officinale*(L.) Y.P.Chen & B.T.Drew (Lamiaceae) | *Eragrostis atrovirens* (Desf.) Trin. ex Steud. (Poaceae) | | *Euphorbia convolvuloides* Hochst. ex Benth. (Euphorbiaceae) |
| 116 | *Calopogonium mucunoides Desv. (Fabaceae)* | *Marsdenia latifolia (Benth.) K.Schum. (Apocynaceae)* | *Elaeis guineensis* Jacq. (Arecaceae) | *Erythrina senegalensis* DC. (Fabaceae) |  | *Euphorbia deightonii*Croizat (Euphorbiaceae) |
| 117 | *Calotropis procera (Aiton) Dryand (Asclepiadaceae)* | *Marsilea quadrifolia L. (Marsileaceae)* | *Eleusine indica* (L.) Gaertn. (Poaceae) | *Erythrina sigmoidea*Hua (Fabaceae) |  | *Euphorbia hirta* L. (Euphorbiaceae) |
| 118 | *Canarium schweinfurthii Engl. (Burseraceae)* | *Mesosphaerum suaveolens (L.) Kuntze (Lamiaceae)* | *Emilia coccinea* (Sims) G.Don (Asteraceae) | *Eucalyptus camaldulensis* Dehn. (Myrtaceae) |  | *Euphorbia lateriflora* Schumach. (Euphorbiaceae) |
| 119 | *Canavalia ensiformis (L.) DC. (Fabaceae)* | *Microdesmis puberula Hook.f. ex Planch. (Pandaceae)* | *Emilia sonchifolia*(L.) DC. (Asteraceae) | *Eucalyptus globulus* Labill. (Myrtaceae) |  | *Euphorbia tirucalli* L. (Euphorbiaceae) |
| 120 | *Canna indica L. (Cannaceae)* | *Milicia excelsa (Welw.) C.C.Berg (Moraceae)* | *Equisetum arvense* L. (Equisetaceae) | *Euphorbia balsamifera* Aiton (Euphorbiaceae) |  | *Euphorbia unispina* N.E.Br. (Euphorbiaceae) |
| 121 | *Cannabis sativa L. (Cannabaceae)* | *Momordica charantia L. (Cucurbitaceae)* | *Eremomastax speciosa* (Hochst.) Cufod. (Acanthaceae) | *Euphorbia convolvuloides* Hochst. ex Benth. (Euphorbiaceae) | | *Faidherbia albida* (Delile) A.Chev. (Fabaceae) |
| 122 | *Capparis brassii DC. (Capparaceae)* | *Mondia whitei (Hook.f.) Skeels (Apocynaceae)* | *Erythrina senegalensis* DC. (Fabaceae) | *Euphorbia heterophylla* L. (Euphorbiaceae) |  | *Ficus exasperata* Vahl (Moraceae) |
| 123 | *Capsicum annuum L. (Solanaceae)* | *Monosis conferta (Benth.) C.Jeffrey (Asteraceae)* | *Eucalyptus camaldulensis* Dehn. (Myrtaceae) | *Euphorbia hirta* L. (Euphorbiaceae) |  | *Ficus ingens* (Miq.) Miq. (Moraceae) |
| 124 | *Capsicum frutescens L. (Solanaceae)* | *Morinda lucida Benth. (Rubiaceae)* | *Eugenia uniflora*L. (Myrtaceae) | *Euphorbia hyssopifolia* L. (Euphorbiaceae) |  | *Ficus macropodocarpa* H.Lév. & Vaniot (Moraceae) |
| 125 | *Carapa procera DC. (Meliaceae)* | *Morinda morindoides (Baker) Milne-Redh. (Rubiaceae)* | *Euphorbia heterophylla* L. (Euphorbiaceae) | *Euphorbia prostrata* Aiton (Euphorbiaceae) |  | *Ficus platyphylla* Delile (Moraceae) |
| 126 | *Carica papaya L. (Caricaceae)* | *Moringa oleifera Lam. (Moringaceae)* | *Euphorbia hirta* L. (Euphorbiaceae) | *Evolvulus alsinoides*(L.) L. (Convolvulaceae) |  | *Ficus sycomorus* L. (Moraceae) |
| 127 | *Carpolobia lutea G.Don (Polygalaceae)* | *Musa ×sapientum L. (Musaceae)* | *Ficus carica*L. (Moraceae) | *Excoecaria grahamii* Stapf (Euphorbiaceae) |  | *Ficus umbellata* Vahl (Moraceae) |
| 128 | *Cascabela thevetia (L.) Lippold (Apocynaceae)* | *Musa x paradisiaca L. (Musaceae)* | *Ficus exasperata* Vahl (Moraceae) | *Faidherbia albida* (Delile) A.Chev. (Fabaceae) |  | *Flueggea virosa* (Roxb. ex Willd.) Royle (Phyllanthaceae) |
| 129 | *Cassia fistula L. (Fabaceae)* | *Musanga cecropioides R.Br. ex Tedlie (Urticaceae)* | *Ficus lutea* Vahl (Moraceae) | *Ficus abutilifolia* (Miq.) Miq. (Moraceae) |  | *Fuirena umbellata* Rottb. (Cyperaceae) |
| 130 | *Cassia sieberiana DC. (Fabaceae)* | *Nauclea diderrichii (De Wild.) Merr. (Rubiaceae)* | *Foeniculum vulgare* Mill. (Apiaceae) | *Ficus capensis* Thunb. (Moraceae) |  | *Garcinia kola* Heckel (Clusiaceae) |
| 131 | *Cassytha filiformis L. (Lauraceae)* | *Nauclea latifolia Sm. (Rubiaceae)* | *Funtumia africana* (Benth.) Stapf (Apocynaceae) | *Ficus exasperata* Vahl (Moraceae) |  | *Gardenia aqualla* Stapf & Hutch. (Rubiaceae) |
| 132 | *Ceiba pentandra (L.) Gaertn. (Malvaceae)* | *Neocarya macrophylla (Sabine) Prance (Chrysobalanaceae)* | *Funtumia elastica*(Preuss) Stapf (Apocynaceae) | *Ficus glumosa* Delile (Moraceae) |  | *Gladiolus ferrugineus* Goldblatt & J.C.Manning (Iridaceae) |
| 133 | *Celosia argentea L. (Amaranthaceae)* | *Nephrolepis cordifolia (L.) C.Presl (Polypodiaceae)* | *Galium aparine* L. (Rubiaceae) | *Ficus platyphylla* Delile (Moraceae) |  | *Gloriosa superba* L. (Colchicaceae) |
| 134 | *Celosia trigyna L. (Amaranthaceae)* | *Newbouldia laevis (Beauverd) Seem. (Bignoniaceae)* | *Gambeya africana* (A.DC.) Pierre (Sapotaceae) | *Ficus polita* Vahl (Moraceae) |  | *Glycine max* (L.) Merr. (Fabaceae) |
| 135 | *Celtis mildbraedii Engl. (Cannabaceae)* | *Ocimum gratissimum L. (Lamiaceae)* | *Garcinia kola* Heckel (Clusiaceae) | *Ficus sur* Forssk. (Moraceae) |  | *Glyphaea brevis*(Biehler) Monach. (Malvaceae) |
| 136 | *Celtis zenkeri Engl. (Cannabaceae)* | *Ophioglossum vulgatum L. (Ophioglossaceae)* | *Gloriosa superba* L. (Colchicaceae) | *Ficus sycomorus* L. (Moraceae) |  | *Gnetum africanum* Welw. (Gnetaceae) |
| 137 | *Centaurea perrottettii DC. (Asteraceae)* | *Osmunda regalis L. (Osmundaceae)* | *Glycine max* (L.) Merr. (Fabaceae) | *Ficus thonningii* Blume (Moraceae) |  | *Gossypium barbadense* L. (Malvaceae) |
| 138 | *Centrosema plumieri (Turpin ex Pers.) Benth. (Fabaceae)* | *Pachylobus edulis G.Don (Burseraceae)* | *Glyphaea brevis*(Biehler) Monach. (Malvaceae) | *Ficus trichopoda*Baker (Moraceae) |  | *Grewia mollis* Juss. (Malvaceae) |
| 139 | *Centrosema pubescens Benth. (Fabaceae)* | *Palisota hirsuta (Thunb.) K.Schum. (Commelinaceae)* | *Gnetum africanum* Welw. (Gnetaceae) | *Ficus vallis-choudae* Delile (Moraceae) |  | *Guiera senegalensis* J.F.Gmel. (Combretaceae) |
| 140 | *Ceratonia siliqua L. (Fabaceae)* | *Pennisetum purpureum Schumach. (Poaceae)* | *Gossypium barbadense* L. (Malvaceae) | *Gambeya albida* (G.Don) Aubrév. & Pellegr. (Sapotaceae) | | *Guilandina bonduc* L. (Fabaceae) |
| 141 | *Cerbera odollam Gaertn. (Apocynaceae)* | *Peperomia pellucida (L.) Kunth (Piperaceae)* | *Gossypium hirsutum* L. (Malvaceae) | *Garcinia kola* Heckel (Clusiaceae) |  | *Gymnanthemum amygdalinum* (Delile) Sch.Bip. ex Walp. (Asteraceae) |
| 142 | *Chamaecrista mimosoides (L.) Greene (Fabaceae)* | *Pergularia daemia (Forssk.) Chiov. (Apocynaceae)* | *Hamamelis virginiana* L. (Hamamelidaceae) | *Gardenia aqualla* Stapf & Hutch. (Rubiaceae) |  | *Gymnema sylvestre* (Retz.) R.Br. ex Sm. (Apocynaceae) |
| 143 | *Chasmanthera dependens Hochst. (Menispermaceae)* | *Persea americana Mill. (Lauraceae)* | *Harungana madagascariensis* Lam. ex Poir. (Hypericaceae) | *Gardenia erubescens* Stapf & Hutch. (Rubiaceae) |  | *Gymnosporia senegalensis* (Lam.) Loes. (Celastraceae) |
| 144 | *Chassalia kolly (Schumach.) Hepper (Rubiaceae)* | *Petersianthus macrocarpus (P.Beauv.) Liben (Lecythidaceae)* | *Heinsia crinita* (Wennberg) G.Taylor (Rubiaceae) | *Gossypium barbadense* L. (Malvaceae) |  | *Harungana madagascariensis* Lam. ex Poir. (Hypericaceae) |
| 145 | *Chloris pilosa Schumach. (Poaceae)* | *Petiveria alliacea L. (Petiveriaceae)* | *Heliotropium indicum* L. (Boraginaceae) | *Grewia mollis* Juss. (Malvaceae) |  | *Heliotropium indicum* L. (Boraginaceae) |
| 146 | *Chromolaena odorata (L.) R.M.King & H.Rob. (Asteraceae)* | *Phyllanthus amarus Schumach. & Thonn. (Phyllanthaceae)* | *Hexasepalum sarmentosum* (Sw.) Delprete & J.H.Kirkbr. (Rubiaceae) | *Grewia villosa* Willd. (Malvaceae) |  | *Hibiscus cannabinus* L. (Malvaceae) |
| 147 | *Chrysopogon nigritanus (Benth.) Veldkamp (Poaceae)* | *Phyllanthus muellerianus (Kuntze) Exell (Phyllanthaceae)* | *Hibiscus surattensis*L. (Malvaceae) | *Guiera senegalensis* J.F.Gmel. (Combretaceae) |  | *Hibiscus sabdariffa* L. (Malvaceae) |
| 148 | *Cinchona pubescens Vahl (Rubiaceae)* | *Physalis angulata L. (Solanaceae)* | *Hibiscus × rosa-sinensis* L. (Malvaceae) | *Gymnosporia senegalensis* (Lam.) Loes. (Celastraceae) | | *Holarrhena floribunda* (G.Don) T.Durand & Schinz (Apocynaceae) |
| 149 | *Cinnamomum camphora (L.) J. Presl (Lauraceae)* | Picralima nitida (Stapf) T.Durand & H.Durand (Apocynaceae) | *Homalium letestui*Pellegr*. (Salicaceae)* | *Haematostaphis barteri* Hook.fil. (Anacardiaceae) |  | *Hoslundia opposita* Vahl (Lamiaceae) |
| 150 | *Cissampelos mucronata A.Rich. (Menispermaceae)* | *Piper guineense Schumach. & Thonn. (Piperaceae)* | *Hybanthus enneaspermus* (L.) F.Muell. (Violaceae) | *Hallea stipulosa* (DC.) J.-F.Leroy (Rubiaceae) |  | *Hybanthus enneaspermus* (L.) F.Muell. (Violaceae) |
| 151 | *Cissampelos owariensis P.Beauv. ex DC. (Menispermaceae)* | *Prosopis africana (Guill. & Perr.) Taub. (Fabaceae)* | *Icacina trichantha* Oliv. (Icacinaceae) | *Hibiscus cannabinus* L. (Malvaceae) |  | *Hymenocardia acida* Tul. (Phyllanthaceae) |
| 152 | *Cissus aralioides (Welw. ex Baker) Planch. (Vitaceae)* | *Psidium guajava L. (Myrtaceae)* | *Ipomoea batatas* (L.) Lam. (Convolvulaceae) | *Hibiscus sabdariffa* L. (Malvaceae) |  | *Imperata cylindrica* (L.) Raeusch. (Poaceae) |
| 153 | *Cissus arguta Hook.f. (Vitaceae)* | *Pteridium aquilinum (L.) Kuhn (Dennstaedtiaceae)* | *Ipomoea involucrata* P.Beauv. (Convolvulaceae) | *Holarrhena floribunda* (G.Don) T.Durand & Schinz (Apocynaceae) | | *Indigofera erecta*Thunb. (Fabaceae) |
| 154 | *Cissus populnea Guill. & Perr. (Vitaceae)* | *Pteris quadriaurita Retz. (Pteridaceae)* | *Ipomoea mauritiana* Jacq. (Convolvulaceae) | *Hygrophila auriculata* (Schumach.) Heine (Acanthaceae) | | *Ipomoea aquatica* Forssk. (Convolvulaceae) |
| 155 | *Cissus quadrangularis L. (Vitaceae)* | *Pteris vittata L. (Pteridaceae)* | Ipomoea quamoclit L. (Convolvulaceae) | *Hymenocardia acida* Tul. (Phyllanthaceae) |  | *Jatropha curcas* L. (Euphorbiaceae) |
| 156 | *Citrullus colocynthis (L.) Schrad. (Cucurbitaceae)* | *Pterocarpus santalinoides L'Hér. ex DC. (Fabaceae)* | *Irvingia gabonensis* (Aubry-Lecomte ex O'Rorke) Baill. (Irvingiaceae) | *Hyphaene thebaica* (L.) Mart. (Arecaceae) |  | *Khaya ivorensis* A.Chev. (Meliaceae) |
| 157 | *Citrullus colocynthis (L.) Schrad. (Cucurbitaceae)* | *Pycnanthus angolensis (Welw.) Warb. (Myristicaceae)* | *Ixora coccinea* L. (Rubiaceae) | *Indigofera astragalina* DC. (Fabaceae) |  | *Khaya senegalensis* (Desr.) A.Juss.) (Meliaceae) |
| 158 | *Citrullus lanatus (Thunb.) Matsum. & Nakai (Cucurbitaceae)* | *Pyrenacantha staudtii (Engl.) Engl. (Icacinaceae)* | *Jatropha curcas* L. (Euphorbiaceae) | *Indigofera hirsuta* L. (Fabaceae) |  | *Kigelia africana* (Lam.) Benth. (Bignoniaceae) |
| 159 | *Citrus ×aurantium L. (Rutaceae)* | *Rauvolfia vomitoria Wennberg (Apocynaceae)* | *Jatropha gossypifolia* L. (Euphorbiaceae) | *Indigofera tinctoria* L. (Fabaceae0 |  | *Laccosperma secundiflorum* (P.Beauv.) Kuntze (Arecaceae) |
| 160 | *Citrus medica var. limon L. (Rutaceae)* | *Rytigynia nigerica (S.Moore) Robyns (Rubiaceae)* | *Jatropha tanjorensis* J.L.Ellis & Saroja (Euphorbiaceae) | *Ipomoea asarifolia* (Desr.) Roem. & Schult. (Convolvulaceae) | | *Lagenaria siceraria* (Molina) Standl. (Cucurbitaceae) |
| 161 | *Citrus sinensis (L.) Osbeck (Rutaceae)* | *Securidaca longipedunculata Fresen. (Polygalaceae)* | *Justicia insularis* T.Anderson (Acanthaceae) | *Ipomoea batatas* (L.) Lam. (Convolvulaceae) |  | *Lannea acida* A.Rich. (Anacardiaceae) |
| 162 | *Citrus × aurantiifolia (Christm.) Swingle (Rutaceae)* | *Selaginella cinerascens A.A.Eaton (Selaginellaceae)* | *Kalanchoe pinnata* (Lam.) Pers. (Crassulaceae) | *Isoberlinia doka* Craib & Stapf (Fabaceae) |  | *Lannea kerstingii*Engl. & K.Krause (Anacardiaceae) |
| 163 | *Citrus × paradisi Macfad. (Rutaceae)* | *Selaginella pallescens (C.Presl) Spring (Selaginellaceae)* | *Khaya grandifoliola* C.DC. (Meliaceae) | *Jatropha curcas* L. (Euphorbiaceae) |  | *Lannea microcarpa* Engl. & K.Krause (Anacardiaceae) |
| 164 | *Clausena anisata (Willd.) Hook.f. ex Benth. (Rutaceae)* | *Selaginella serpens (Desv.) Spring (Selaginellaceae)* | *Khaya ivorensis* A.Chev. (Meliaceae) | *Khaya senegalensis* (Desr.) A.Juss.) (Meliaceae) |  | *Lantana camara* L. (Verbenaceae) |
| 165 | *Cleistopholis patens (Benth.) Engl. & Diels (Annonaceae)* | *Selliguea capitellata (Mett.) X.C.Zhang & L.J.He (Polypodiaceae)* | *Kigelia africana* (Lam.) Benth. (Bignoniaceae) | *Laccosperma secundiflorum* (P.Beauv.) Kuntze (Arecaceae) | | *Lasiosiphon kraussianus* (Meisn.) Meisn. (Thymelaeaceae) |
| 166 | *Cleome ciliata Schumach. & Thonn. (Cleomaceae)* | *Senegalia senegal (L.) Britton (Fabaceae)* | *Lagenaria breviflora* (Benth.) Roberty (Cucurbitaceae) | *Lannea acida* A.Rich. (Anacardiaceae) |  | *Lawsonia inermis* L. (Lythraceae) |
| 167 | *Cleome gynandra L. (Cleomaceae)* | *Senna podocarpa (Guill. & Perr.) Lock (Fabaceae)* | *Lantana camara* L. (Verbenaceae) | *Lannea barteri* (Oliv.) Engl. (Anacardiaceae) |  | *Lonchocarpus cyanescens* (Schumach. & Thonn.) Benth. (Fabaceae) |
| 168 | *Clerodendrum capitatum (Willd.) Schumach. (Lamiaceae)* | *Senna siamea (Lam.) H.S.Irwin & Barneby (Fabaceae)* | *Laportea aestuans* (L.) Chew (Urticaceae) | *Lannea microcarpa* Engl. & K.Krause (Anacardiaceae) |  | *Lophira alata* Banks ex C.F.Gaertn. (Ochnaceae) |
| 169 | *Clerodendrum paniculatum L. (Lamiaceae)* | *Sida acuta Burm.f. (Malvaceae)* | *Lasianthera africana* P.Beauv. (Stemonuraceae) | *Lawsonia inermis* L. (Lythraceae) |  | *Lophira lanceolata* Tiegh. ex Keay (Ochnaceae) |
| 170 | *Clerodendrum umbellatum Poir. (Lamiaceae)* | *Solanum americanum Mill. (Solanaceae)* | *Lawsonia inermis* L. (Lythraceae) | *Leptadenia lanceolata* (Poir.) Goyder (Apocynaceae) |  | *Luffa cylindrica* (L.) M.Roem. (Cucurbitaceae) |
| 171 | *Clerodendrum volubile P.Beauv. (Lamiaceae)* | *Sorghum bicolor (L.) Moench (Poaceae)* | *Leplaea thompsonii*(Sprague & Hutch.) E.J.M.Koenen & J.J.de Wilde (Meliaceae) | *Leucas martinicensis*(Jacq.) R.Br. (Lamiaceae) |  | *Mangifera indica* L. ( Anacardiaceae) |
| 172 | *Cnestis corniculata Lam. (Connaraceae)* | *Spathodea campanulata P.Beauv. (Bignoniaceae)* | *Lippia multiflora* Moldenke (Verbenaceae) | *Lophira alata* Banks ex C.F.Gaertn. (Ochnaceae) |  | *Manihot esculenta* Crantz (Euphorbiaceae) |
| 173 | *Cnestis ferruginea Vahl ex DC. (Connaraceae)* | *Sphenocentrum jollyanum Pierre (Menispermaceae)* | *Loeseneriella africana* (Willd.) R.Wilczek (Celastraceae) | *Lophira lanceolata* Tiegh. ex Keay (Ochnaceae) |  | *Maranthes polyandra* (Benth.) Prance (Chrysobalanaceae) |
| 174 | *Cnidoscolus aconitifolius (Mill.) I.M.Johnst. (Euphorbiaceae)* | *Spondias mombin L. (Anacardiaceae)* | *Lonchocarpus cyanescens* (Schumach. & Thonn.) Benth. (Fabaceae) | *Ludwigia octovalvis* (Jacq.) P.H.Raven (Onagraceae) |  | *Margaritaria discoidea* (Baill.) G.L.Webster (Phyllanthaceae) |
| 175 | *Coccinia barteri (Hook.f.) Keay (Cucurbitaceae)* | *Stachytarpheta cayennensis (Rich.) Vahl (Verbenaceae)* | *Lophira alata* Banks ex C.F.Gaertn. (Ochnaceae) | *Maerua oblongifolia* (Forssk.) A.Rich. (Capparaceae) |  | *Marsdenia latifolia*(Benth.) K.Schum. (Apocynaceae) |
| 176 | *Coccinia grandis (L.) Voigt (Cucurbitaceae)* | *Strophanthus hispidus DC. (Apocynaceae)* | *Ludwigia hyssopifolia* (G.Don) Exell (Onagraceae) | *Mangifera indica* L. ( Anacardiaceae) |  | *Massularia acuminata* (G.Don) Bullock ex Hoyle (Rubiaceae) |
| 177 | *Cochlospermum tinctorium Perrier ex A.Rich. (Bixaceae)* | *Synedrella nodiflora (L.) Gaertn. (Asteraceae)* | *Ludwigia octovalvis* (Jacq.) P.H.Raven (Onagraceae) | *Manihot esculenta* Crantz (Euphorbiaceae) |  | *Melissa officinalis* L. (Lamiaceae) |
| 178 | *Cocos nucifera L. (Arecaceae)* | *Tectaria coadunata (J.Sm.) C.Chr. (Polypodiaceae)* | *Maesobotrya barteri*(Baill.) Hutch. (Phyllanthaceae) | *Mentha × piperita*L. (Lamiaceae) |  | *Mesosphaerum suaveolens* (L.) Kuntze (Lamiaceae) |
| 179 | *Coix lacryma-jobi L. (Poaceae)* | Terminalia avicennioides Guill. & Perr. (Combretaceae) | *Maesobotrya klaineana* (Pierre) J.Léonard (Phyllanthaceae) | *Mimosa pigra* L. (Fabaceae) |  | *Milicia excelsa* (Welw.) C.C.Berg (Moraceae) |
| 180 | *Cola acuminata (P.Beauv.) Schott & Endl. (Malvaceae)* | *Terminalia catappa L. (Combretaceae)* | *Mallotus oppositifolius* (Geiseler) Müll.Arg. (Euphorbiaceae) | *Mitracarpus hirtus*(L.) DC. (Rubiaceae) |  | *Millettia thonningii*(Schumach. & Thonn.) Baker (Fabaceae) |
| 181 | *Cola gigantea A.Chev. (Malvaceae)* | *Terminalia ivorensis A.Chev. (Combretaceae)* | *Malvastrum coromandelianum* (L.) Garcke (Malvaceae) | *Mitragyna inermis* (Willd.) Kuntze (Rubiaceae) |  | *Mitracarpus hirtus*(L.) DC. (Rubiaceae) |
| 182 | *Cola millenii K.Schum. (Malvaceae)* | *Thelypteris erubescens (Wall. ex Hook.) Ching (Aspleniaceae)* | *Mangifera indica* L. ( Anacardiaceae) | *Momordica balsamina* L. (Rubiaceae) |  | *Mitragyna inermis* (Willd.) Kuntze (Rubiaceae) |
| 183 | *Cola nitida (Vent.) Schott & Endl. (Malvaceae)* | *Theobroma cacao L. (Malvaceae)* | *Manihot esculenta* Crantz (Euphorbiaceae) | *Monotes kerstingii*Gilg (Dipterocarpaceae) |  | *Momordica cabrae* (Cogn.) C.Jeffrey (Cucurbitaceae) |
| 184 | *Colocasia esculenta (L.) Schott (Araceae)* | *Tithonia diversifolia (Hemsl.) A.Gray (Asteraceae)* | *Marsdenia latifolia*(Benth.) K.Schum. (Apocynaceae) | *Moringa oleifera* Lam. (Moringaceae) |  | *Momordica charantia* L. (Cucurbitaceae) |
| 185 | *Combretum bracteatum (M.A.Lawson) Engl. & Diels. (Combretaceae)* | *Treculia africana Decne. ex Trécul (Moraceae)* | *Massularia acuminata* (G.Don) Bullock ex Hoyle (Rubiaceae) | *Moringa oleifera Lam. (Moringaceae)* |  | *Morinda lucida* Benth. (Rubiaceae) |
| 186 | *Combretum indicum (L.) DeFilipps (Combretaceae)* | *Trema orientale (L.) Blume (Cannabaceae)* | *Melanthera scandens* (Schumach. & Thonn.) Roberty (Asteraceae) | *Musa ×sapientum* L. (Musaceae) |  | *Moringa oleifera* Lam. (Moringaceae) |
| 187 | *Combretum micranthum G. Don (Combretaceae)* | *Trichilia monadelpha (Thonn.) J.J.de Wilde (Meliaceae)* | *Mesosphaerum pectinatum* (L.) Kuntze (Lamiaceae) | *Musa acuminata* Colla (Musaceae) |  | *Musa ×sapientum* L. (Musaceae) |
| 188 | *Combretum molle R.Br. ex G.Don (Combretaceae)* | *Tridax procumbens L. (Asteraceae)* | *Mesosphaerum suaveolens* (L.) Kuntze (Lamiaceae) | *Musa x paradisiaca* L. (Musaceae) |  | *Musa x paradisiaca* L. (Musaceae) |
| 189 | *Combretum mucronatum Schumach. & Thonn. (Combretaceae)* | *Triumfetta cordifolia A.Rich. (Malvaceae)* | *Microdesmis puberula* Hook.f. ex Planch. (Pandaceae) | *Nauclea diderrichii* (De Wild.) Merr. (Rubiaceae) |  | *Nauclea diderrichii* (De Wild.) Merr. (Rubiaceae) |
| 190 | *Combretum paniculatum Vent. (Combretaceae)* | *Urena lobata L. (Malvaceae)* | *Millettia aboensis* (Hook.) Baker (Fabaceae) | *Nauclea latifolia* Sm. (Rubiaceae) |  | *Nauclea latifolia* Sm. (Rubiaceae) |
| 191 | *Combretum racemosum P.Beauv. (Combretaceae)* | *Uvaria chamae P. Beauv. (Annonaceae)* | *Mimosa pudica* L. (Fabaceae) | *Neocarya macrophylla* (Sabine) Prance (Chrysobalanaceae) |  | *Newbouldia laevis* (Beauverd) Seem. (Bignoniaceae) |
| 192 | *Combretum sordidum Exell (Combretaceae)* | *Uvariopsis tripetala (Baker f.) G.E.Schatz (Annonaceae)* | *Mitracarpus hirtus*(L.) DC. (Rubiaceae) | *Nicotiana tabacum* L. (Solanaceae) |  | *Nicotiana tabacum* L. (Solanaceae) |
| 193 | *Combretum tomentosum G.Don (Combretaceae)* | *Vachellia nilotica (L.) P.J.H.Hurter & Mabb. (Fabaceae)* | *Mondia whitei* (Hook.f.) Skeels (Apocynaceae) | *Nigella sativa* L. (Ranunculaceae) |  | *Nymphaea lotus*L. (Nymphaeaceae) |
| 194 | *Commelina africana L. (Commelinaceae)* | *Vernonia amygdalina Delile (Asteraceae)* | *Monodora myristica* (Gaertn.) Dunal (Annonaceae) | *Nymphaea odorata* Aiton (Nymphaeaceae) |  | *Ocimum americanum* L. (Lamiaceae) |
| 195 | *Commelina erecta L. (Commelinaceae)* | *Vitex doniana sweet (Lamiaceae)* | *Monosis conferta* (Benth.) C.Jeffrey (Asteraceae) | *Ocimum basilicum* L. (Lamiaceae) |  | *Ocimum basilicum* L. (Lamiaceae) |
| 196 | *Corchorus aestuans L. (Malvaceae)* | *Woodwardia unigemmata (Makino) Nakai (Aspleniaceae)* | *Morinda lucida* Benth. (Rubiaceae) | *Ocimum gratissimum* L. (Lamiaceae) |  | *Ocimum gratissimum* L. (Lamiaceae) |
| 197 | *Corchorus olitorius L. (Malvaceae)* | *Ximenia americana L. (Olacaceae)* | *Morinda morindoides* (Baker) Milne-Redh. (Rubiaceae) | *Olax subscorpioidea*Oliv. (Olacaceae) |  | *Olax subscorpioidea*Oliv. (Olacaceae) |
| 198 | *Cordia platythyrsa Baker (Boraginaceae)* | *Xylopia aethiopica (Dunal) A.Rich. (Annonaceae)* | *Moringa oleifera* Lam. (Moringaceae) | *Olea europaea* L. (Oleaceae) |  | *Oxytenanthera abyssinica* (A.Rich.) Munro (Poaceae) |
| 199 | *Costus afer Ker Gawl. (Costaceae)* | *Zanthoxylum zanthoxyloides (Lam.) Zepern. & Timler (Rutaceae)* | *Musa ×sapientum* L. (Musaceae) | *Opilia amentacea* Roxb. (Opiliaceae) |  | *Ozoroa pulcherrima* (Schweinf.) R.Fern. & A.Fern. (Anacardiaceae) |
| 200 | *Coula edulis Baill. (Olacaceae)* | *Zingiber officinale Roscoe (Zingiberaceae)* | *Musa x paradisiaca* L. (Musaceae) | *Ormocarpum pubescens* (Hochst.) Cufod. ex J.B.Gillett (Fabaceae) | | *Parkia biglobosa* (Jacq.) R.Br. ex G.Don (Fabaceae) |
| 201 | *Crassocephalum crepidioides (Benth.) S.Moore (Asteraceae)* |  | *Musanga cecropioides* R.Br. ex Tedlie (Urticaceae) | *Ozoroa insignis*Delile (Anacardiaceae) |  | *Paullinia pinnata* L. (Sapindaceae) |
| 202 | *Crassocephalum rubens (Juss. ex Jacq.) S.Moore (Asteraceae)* |  | *Nauclea diderrichii* (De Wild.) Merr. (Rubiaceae) | *Ozoroa mucronata* (Bernh. ex C.Krauss) R.Fern. & A.Fern. (Anacardiaceae) | | *Peperomia pellucida* (L.) Kunth (Piperaceae) |
| 203 | *Crateva adansonii DC. (Capparaceae)* |  | *Nauclea latifolia* Sm. (Rubiaceae) | *Parkia biglobosa* (Jacq.) R.Br. ex G.Don (Fabaceae) |  | *Petiveria alliacea* L. (Petiveriaceae) |
| 204 | *Crateva monticola (Gilg & Gilg-Ben.) Christenh. & Byng (Capparaceae)* |  | *Neocarya macrophylla* (Sabine) Prance (Chrysobalanaceae) | *Parkia filicoidea*Welw. ex Oliv. (Fabaceae) |  | *Philenoptera laxiflora* (Guill. & Perr.) Roberty (Fabaceae) |
| 205 | *Crescentia cujete L. (Bignoniaceae)* |  | *Newbouldia laevis* (Beauverd) Seem. (Bignoniaceae) | *Pavonia senegalensis*(Cav.) Leistner (Malvaceae) |  | *Phyllanthus amarus* Schumach. & Thonn. (Phyllanthaceae) |
| 206 | *Crinum jagus (J.Thomps.) Dandy (Amaryllidaceae)* |  | *Ocimum basilicum* L. (Lamiaceae) | *Pennisetum purpureum* Schumach. (Poaceae) |  | *Physalis angulata* L. (Solanaceae) |
| 207 | *Crotalaria retusa L. (Fabaceae)* |  | *Ocimum gratissimum* L. (Lamiaceae ) | *Pericopsis laxiflora* (Benth. ex Baker) Meeuwen (Fabaceae) | | *Piliostigma reticulatum* (DC.) Hochst. (Fabaceae) |
| 208 | *Croton penduliflorus Hutch. (Euphorbiaceae)* |  | *Oenothera biennis* L. (Onagraceae) | *Persea americana* Mill. (Lauraceae) |  | *Piliostigma thonningii* (Schumach.) Milne-Redh. (Fabaceae) |
| 209 | *Croton zambesicus Müll.Arg. (Euphorbiaceae)* |  | *Olax subscorpioidea*Oliv. (Olacaceae) | *Phoenix dactylifera*L. (Arecaceae) |  | *Piper guineense* Schumach. & Thonn. (Piperaceae) |
| 210 | *Cryptolepis nigrescens (Wennberg) L.Joubert & Bruyns (Apocynaceae)* |  | *Olea europaea* L. (Olacaceae) | *Phragmites australis*(Cav.) Trin. ex Steud. (Poaceae) |  | *Pistia stratiotes* L. (Araceae) |
| 211 | *Cryptolepis sanguinolenta (Lindl.) Schltr. (Apocynaceae)* |  | *Origanum majorana* L. (Lamiaceae) | *Piliostigma reticulatum* (DC.) Hochst. (Fabaceae) |  | *Plumbago zeylanica* L. (Plumbaginaceae) |
| 212 | *Cucumis melo L. (Cucurbitaceae)* |  | *Oxyceros longiflorus* (Lam.) T.Yamaz. (Rubiaceae) | *Piliostigma thonningii* (Schumach.) Milne-Redh. (Fabaceae) | | *Polhillides velutina* (Willd.) H.Ohashi & K.Ohashi (Fabaceae) |
| 213 | *Cucurbita pepo L. (Cucurbitaceae)* |  | *Pachylobus edulis* G.Don (Burseraceae) | *Piper guineense* Schumach. & Thonn. (Piperaceae) |  | *Prosopis africana* (Guill. & Perr.) Taub. (Fabaceae) |
| 214 | *Culcasia scandens P.Beauv. (Araceae)* |  | *Pachylobus klaineanus* (Pierre) Guillaumin (Burseraceae) | *Pistia stratiotes* L. (Araceae) |  | *Pseudocedrela kotschyi* Harms (Meliaceae) |
| 215 | *Curculigo pilosa (Schumach. & Thonn.) Engl. (Hypoxidaceae)* |  | *Palisota hirsuta* (Thunb.) K.Schum. (Commelinaceae) | *Pleurotus tuber-regium* (Fr.) Singer (Pleurotaceae) |  | *Psidium guajava* L. (Myrtaceae) |
| 216 | *Curcuma longa L. (Zingiberaceae)* |  | *Pastinaca sativa* L. (Apiaceae) | *Prosopis africana* (Guill. & Perr.) Taub. (Fabaceae) |  | *Pterocarpus erinaceus* Poir. (Fabaceae) |
| 217 | *Cuscuta australis R.Br. (Convolvulaceae)* |  | *Pelargonium graveolens*L'Hér. (Geraniaceae) | *Pseudocedrela kotschyi* Harms (Meliaceae) |  | *Pterocarpus santalinoides* L'Hér. ex DC. (Fabaceae) |
| 218 | *Cyanthillium cinereum (L.) H.Rob. (Asteraceae)* |  | *Pentaclethra macrophylla* Benth. (Fabaceae) | *Psidium guajava* L. (Myrtaceae) |  | *Pycnanthus angolensis* (Welw.) Warb. (Myristicaceae) |
| 219 | *Cyathula prostrata (L.) Blume (Amaranthaceae)* |  | *Peperomia pellucida* (L.) Kunth (Piperaceae) | *Pterocarpus erinaceus* Poir. (Fabaceae) |  | *Rauvolfia vomitoria*Wennberg (Apocynaceae) |
| 220 | *Cymbopogon citratus (DC.) Stapf (Poaceae)* |  | *Pergularia daemia* (Forssk.) Chiov. (Apocynaceae) | *Ricinus communis*L. (Euphorbiaceae) |  | *Ricinus communis*L. (Euphorbiaceae) |
| 221 | *Cynometra mannii Oliv. (Fabaceae)* |  | *Persea americana* Mill. (Lauraceae) | *Rourea coccinea* (Schumach. & Thonn.) Benth. (Connaraceae) | | *Rourea coccinea* (Schumach. & Thonn.) Benth. (Connaraceae) |
| 222 | *Cyperus cyperoides (L.) Kuntze (Cyperaceae)* |  | *Petersianthus macrocarpus* (P.Beauv.) Liben (Lecythidaceae) | *Schwenckia americana* L. (Solanaceae) |  | *Saccharum officinarum* L. (Poaceae) |
| 223 | *Cyperus esculentus L. (Cyperaceae)* |  | *Petiveria alliacea* L. (Petiveriaceae) | *Sclerocarya birrea* (A.Rich.) Hochst. (Anacardiaceae) |  | *Schwenckia americana* L. (Solanaceae) |
| 224 | *Cyperus haspan L. (Cyperaceae)* |  | *Phyllanthus amarus* Schumach. & Thonn. (Phyllanthaceae) | *Scoparia dulcis*L. (Plantaginaceae) |  | *Secamone afzelii* (Roem. & Schult.) K.Schum. (Apocynaceae) |
| 225 | *Dalbergia lactea Vatke (Fabaceae)* |  | *Phyllanthus emblica*L. (Phyllanthaceae) | *Securidaca longipedunculata* Fresen. (Polygalaceae) |  | *Securidaca longipedunculata* Fresen. (Polygalaceae) |
| 226 | *Dalbergia saxatilis Hook.f. (Fabaceae)* |  | *Phyllanthus muellerianus* (Kuntze) Exell (Phyllanthaceae) | *Senegalia macrostachya* (Rchb. ex DC.) Kyal. & Boatwr. (Fabaceae) | | *Senna alata* (L.) Roxb. (Fabaceae) |
| 227 | *Daniellia ogea (Harms) Rolfe ex Holland (Fabaceae)* |  | *Physalis angulata* L. (Solanaceae) | *Senegalia polyacantha* (Willd.) Seigler & Ebinger (Fabaceae) | | *Senna obtusifolia* (L.) Irwin & Barneby (Fabaceae) |
| 228 | *Daniellia oliveri (Rolfe) Hutch. & Dalziel (Fabaceae)* |  | *Picralima nitida* (Stapf) T.Durand & H.Durand (Apocynaceae) | *Senegalia senegal (L.) Britton* (L.) Willd. (Fabaceae) |  | *Senna occidentalis* (L.) Link (Fabaceae ) |
| 229 | *Datura metel L. (Solanaceae)* |  | *Pimenta dioica*(L.) Merr. (Myrtaceae) | *Senna italica* Mill. (Fabaceae) |  | *Senna podocarpa* (Guill. & Perr.) Lock (Fabaceae) |
| 230 | *Datura stramonium L. (Solanaceae)* |  | *Piper guineense* Schumach. & Thonn. (Piperaceae) | *Senna obtusifolia* (L.) Irwin & Barneby (Fabaceae) |  | *Senna singueana* (Delile) Lock (Fabaceae) |
| 231 | *Deinbollia pinnata (Poir.) Schumach. & Thonn. (Sapindaceae)* |  | *Pistacia vera* L. (Anacardiaceae) | *Senna occidentalis* (L.) Link (Fabaceae ) |  | *Sesamum radiatum*Thonn. ex Hornem. (Pedaliaceae) |
| 232 | *Delonix regia (Bojer ex Hook.) Raf. (Fabaceae)* |  | *Plukenetia conophora* Müll.Arg. (Euphorbiaceae) | *Senna singueana* (Delile) Lock (Fabaceae) |  | *Sida acuta* Burm.f. (Malvaceae) |
| 233 | *Detarium microcarpum Guill. & Perr. (fabaceae)* |  | *Portulaca oleracea* L. (Portulacaceae) | *Senna tora* (L.) Roxb. (Fabaceae) |  | *Sida cordifolia* L. (Malvaceae) |
| 234 | *Dialium guineense Willd. (Fabaceae)* |  | *Psidium guajava* L. (Myrtaceae) | *Sesamum alatum* Thonn. (Pedaliaceae) |  | *Solanum dasyphyllum* Schumach. & Thonn. (Solanaceae) |
| 235 | *Dichapetalum barteri Engl. (Dichapetalaceae)* |  | *Pterocarpus mildbraedii* Harms (Fabaceae) | *Sesamum radiatum*Thonn. ex Hornem. (Pedaliaceae) |  | *Solanum lycopersicum* L. (Solanaceae) |
| 236 | *Dichapetalum madagascariense Poir. (Dichapetalaceae)* |  | *Pterocarpus santalinoides* L'Hér. ex DC. (Fabaceae) | *Sesbania dalzielii*E.Phillips & Hutc*h.* (Fabaceae) |  | *Solanum macrocarpon*L. (Solanaceae) |
| 237 | *Dichapetalum toxicarium (G.Don) Baill. (Dichapetalaceae)* |  | *Punica granatum* L. (Lythraceae) | *Sida ovata*Forssk. (Malvaceae) |  | *Sorghum bicolor* (L.) Moench (Poaceae) |
| 238 | *Dichrostachys cinerea (L.) Wight & Arn. (Fabaceae)* |  | *Pycnanthus angolensis* (Welw.) Warb. (Myristicaceae) | *Solanum americanum* Mill. (Solanaceae) |  | *Spathodea campanulata* P.Beauv. (Bignoniaceae) |
| 239 | *Dieffenbachia seguine (Jacq.) Schott (Araceae)* |  | *Pyrenacantha staudtii*(Engl.) Engl. (Icacinaceae) | *Solanum lycopersicum* L. (Solanaceae) |  | *Spermacoce verticillata* L. (Rubiaceae) |
| 240 | *Digitaria horizontalis Willd. (Poaceae)* |  | *Rauvolfia vomitoria*Wennberg (Apocynaceae) | *Solanum melongena* L. (Solanaceae) |  | *Sphenocentrum jollyanum* Pierre (Menispermaceae) |
| 241 | *Dioclea reflexa Hook.f. (Fabaceae)* |  | *Rheum palmatum* L. (Polygonaceae) | *Spondias mombin* L. (Anacardiaceae) |  | *Spondias mombin* L. (Anacardiaceae) |
| 242 | *Dioscorea bulbifera L. (Dioscoreaceae)* |  | *Ricinus communis* L. (Euphorbiaceae) | *Stachytarpheta jamaicensis* (L.) Vahl (Verbenaceae) |  | *Stachytarpheta indica*(L.) Vahl (Verbenaceae) |
| 243 | *Dioscorea cayenensis subsp. rotundata (Poir.) J.Miège (Dioscoreaceae)* |  | *Rinorea welwitschii*(Oliv.) Kuntze (Moraceae) | *Sterculia setigera* Delile (Malvaceae) |  | *Sterculia setigera* Delile (Malvaceae) |
| 244 | *Dioscorea dumetorum (Kunth) Pax (Dioscoreaceae)* |  | *Rosa canina* L. (Rosaceae) | *Stereospermum kunthianum* Cham. (Bignoniaceae) |  | *Stereospermum kunthianum* Cham. (Bignoniaceae) |
| 245 | *Dioscorea hirtiflora Benth. (Dioscoreaceae)* |  | *Rosmarinus officinalis* L. (Lamiaceae) | *Striga hermonthica* (Delile) Benth. (Orobanchaceae) |  | *Striga hermonthica* (Delile) Benth. (Orobanchaceae) |
| 246 | *Dioscorea praehensilis Benth. (Dioscoreaceae)* |  | *Rubus idaeus* L. (Rosaceae) | *Strychnos spinosa* Lam. (Loganiaceae) |  | *Strychnos spinosa* Lam. (Loganiaceae) |
| 247 | *Diospyros alboflavescens (Gürke) F.White (Ebenaceae)* |  | *Rytigynia nigerica* (S.Moore) Robyns (Rubiaceae) | *Stylosanthes erecta* P.Beauv. (Fabaceae) |  | *Syzygium aromaticum* (L.) Merr. & L.M.Perry (Myrtaceae) |
| 248 | *Diospyros canaliculata De Wild. (Ebenaceae)* |  | *Saccharum officinarum* L. (Poaceae) | *Syzygium aromaticum* (L.) Merr. & L.M.Perry (Myrtaceae) | | *Syzygium guineense* (Willd.) DC. (Myrtaceae) |
| 249 | *Diospyros mespiliformis Hochst. ex A.DC. (Ebenaceae)* |  | *Sacoglottis gabonensis*(Baill.) Urb. (Humiriaceae) | *Talinum fruticosum* (L.) Juss. (Talinaceae) |  | *Tamarindus indica* L. (Fabaceae) |
| 250 | *Diospyros monbuttensis Gürke (Ebenaceae)* |  | *Salvia officinalis* L. (Lamiaceae) | *Tamarindus indica* L. (Fabaceae) |  | *Tapinanthus globiferus* (A.Rich.) Tiegh. (Loranthaceae) |
| 251 | *Distemonanthus benthamianus Baill. (Fabaceae)* |  | *Satureja hortensis* L. (Lamiaceae) | *Tephrosia linearis* (Willd.) Pers. (Fabaceae) |  | *Terminalia avicennioides* Guill. & Perr. (Combretaceae) |
| 252 | *Distimake aegyptius (L.) A.R.Simões & Staples (Convolvulaceae)* |  | *Scoparia dulcis*L. (Plantaginaceae) | *Terminalia avicennioides* Guill. & Perr. (Combretaceae) | | *Terminalia macroptera* Guill. & Perr. (Combretaceae) |
| 253 | *Dombeya buettneri K.Schum. (Malvaceae)* |  | *Securidaca longipedunculata* Fresen. (Polygalaceae) | *Terminalia macroptera* Guill. & Perr. (Combretaceae) |  | *Terminalia mollis* M.A.Lawson (Combretaceae) |
| 254 | *Dorstenia prorepens Engl. (Moraceae)* |  | *Senegalia senegal* (L.) Britton (Fabaceae) | *Vachellia gerrardi* (Benth.) P.J.H.Hurter (Fabaceae) |  | *Terminalia schimperiana* Hochst (Combretaceae) |
| 255 | *Dracaena liberica (Gérôme & Labroy) Byng & Christenh. (Asparagaceae)* |  | *Senna alata* (L.) Roxb. (Fabaceae) | *Vachellia nilotica (L.) P.J.H.Hurter & Mabb.* (Fabaceae) | | *Tetracera potatoria* Afzel. ex G.Don (Dilleniaceae) |
| 256 | *Dracaena trifasciata subsp. Trifasciata (Prain) Mabb. (Asparagaceae)* |  | *Senna occidentalis* (L.) Link (Fabaceae) | *Vachellia sieberiana* (DC.) Kyal. & Boatwr. (Fabaceae) | | *Tetrapleura tetraptera* (Schum. and Thonn.) Taub. (Fabaceae) |
| 257 | *Drypetes gossweileri S.Moore (Putranjivaceae)* |  | *Senna podocarpa* (Guill. & Perr.) Lock (Fabaceae) | *Vernonia amygdalina* Delile (Asteraceae) |  | *Theobroma cacao* L. (Malvaceae) |
| 258 | *Duranta erecta L. (Verbenaceae)* |  | *Senna siamea* (Lam.) H.S.Irwin & Barneby (Fabaceae) | *Vernoniastrum ambiguum*(Kotschy & Peyr.) H.Rob. (Asteraceae) | | *Thymus vulgaris* L. (Lamiaceae) |
| 259 | *Dysphania ambrosioides (L.) Mosyakin & Clemants (Amaranthaceae)* |  | *Senna tora* (L.) Roxb. (Fabaceae) | *Veronica kotschyana* Benth. (Plantaginaceae) |  | *Tithonia diversifolia* (Hemsl.) A.Gray (Asteraceae) |
| 260 | *Eclipta alba (L.) Hassk. (Asteraceae)* |  | *Setaria sphacelata* (Schumach.) Stapf & C.E.Hubb. ex Moss (Poaceae) | *Vigna unguiculata* (L.) Walp. (Fabaceae) |  | *Trema orientale* (L.) Blume (Cannabaceae) |
| 261 | *Eclipta prostrata (L.) L. (Asteraceae)* |  | *Sida acuta* Burm.f. (Malvaceae) | *Vitellaria paradoxa* C.F Gaertn (Sapotaceae) |  | *Trichilia monadelpha* (Thonn.) J.J.de Wilde (Meliaceae) |
| 262 | *Ehretia cymosa Thonn. (Boraginaceae)* |  | *Solanum americanum* Mill. (Solanaceae) | *Vitex chrysocarpa* Planch. (Lamiaceae) |  | *Tridax procumbens* L. (Asteraceae) |
| 263 | *Elaeis guineensis Jacq. (Arecaceae)* |  | *Solanum melongena* L. (Solanaceae) | *Vitex doniana* sweet (Lamiaceae) |  | *Uvaria afzelii* Scott. Elliot (Annonaceae) |
| 264 | *Eleusine indica (L.) Gaertn. (Poaceae)* |  | *Solanum torvum* Sw. (Solanaceae) | *Waltheria indica* L. (Malvaceae) |  | *Uvaria chamae* P. Beauv. (Annonaceae) |
| 265 | *Eleutheranthera ruderalis (Sw.) Sch.Bip. (Asteraceae)* |  | *Solanum tuberosum* L. (Solanaceae) | *Xanthosoma sagittifolium* (L.) Schott (Araceae) |  | *Uvariopsis tripetala* (Baker f.) G.E.Schatz (Annonaceae) |
| 266 | *Elytraria marginata Vahl (Acanthaceae)* |  | *Solenostemon monostachyus* (P.Beauv.) Roberty (Lamiaceae) | *Ximenia americana* L. (Olacaceae) |  | *Vachellia amythethophylla* (Steud. ex A.Rich.) Kyal. & Boatwr. (Fabaceae) |
| 267 | *Emilia abyssinica (Sch.Bip. ex A.Rich.) C.Jeffrey (Asteraceae)* |  | *Sorghum bicolor* (L.) Moench (Poaceae) | *Zaleya pentandra* (L.) C.Jeffrey (Aizoaceae) |  | *Vachellia nilotica* (L.) P.J.H.Hurter & Mabb. (Fabaceae) |
| 268 | *Emilia coccinea (Sims) G.Don (Asteraceae)* |  | *Spathodea campanulata* P.Beauv. (Bignoniaceae) | *Zingiber officinale* Roscoe (Zingiberaceae) |  | *Vachellia seyal* (Delile) P.J.H.Hurter (Fabaceae) |
| 269 | *Emilia sonchifolia (L.) DC. (Asteraceae)* |  | *Spermacoce verticillata* L. (Rubiaceae) | *Ziziphus abyssinica* Hochst. ex A.Rich. (Rhamnaceae) |  | *Vachellia sieberiana* (DC.) Kyal. & Boatwr. (Fabaceae) |
| 270 | *Entada gigas (L.) Fawc. & Rendle (Fabaceae)* |  | *Sphenocentrum jollyanum* Pierre (Menispermaceae) | *Ziziphus jujuba* Mill. (Rhamnaceae) |  | *Vangueria agrestis*(Schweinf. ex Hiern) Lantz (Rubiaceae) |
| 271 | *Entandrophragma angolense (Welw.) C.DC. (Meliaceae)* |  | *Spondias mombin* L. (Anacardiaceae) | *Ziziphus lotus* (L.) Lam. (Rhamnaceae) |  | *Vernonia amygdalina* Delile (Asteraceae) |
| 272 | *Entandrophragma utile (Dawe & Sprague) Sprague (Meliaceae)* |  | *Stachytarpheta cayennensis* (Rich.) Vahl (Verbenaceae) | *Ziziphus mauritiana* Lam. (Rhamnaceae) |  | *Vigna unguiculata* (L.) Walp. (Fabaceae) |
| 273 | *Eragrostis japonica (Thunb.) Trin. (Poaceae)* |  | *Stellaria media* (L.) Vill. (Caryophyllaceae) | *Ziziphus mucronata* Willd. (Rhamnaceae) |  | *Viscum album* L. (Santalaceae) |
| 274 | *Erigeron floribundus (Kunth) Sch.Bip. (Asteraceae)* |  | *Sterculia tragacantha* Lindl. (Malvaceae) | *Ziziphus spina-christi* (L.) Desf. (Rhamnaceae) |  | *Vitellaria paradoxa* C.F Gaertn (Sapotaceae) |
| 275 | *Erythrina senegalensis DC. (Fabaceae)* |  | *Symphonia globulifera* L.f. (Clusiaceae) |  |  | *Vitex doniana* sweet (Lamiaceae) |
| 276 | *Erythrococca anomala (Juss. ex Poir.) Prain (Euphorbiaceae)* |  | *Synedrella nodiflora* (L.) Gaertn. (Asteraceae) |  |  | *Waltheria indica* L. (Malvaceae) |
| 277 | *Erythrophleum africanum (Benth.) Harms (Fabaceae)* |  | *Synsepalum dulcificum* (Schumach. & Thonn.) Daniell (Sapotaceae) |  |  | *Ximenia americana* L. (Olacaceae) |
| 278 | *Erythrophleum suaveolens (Guill. & Perr.) Brenan (Fabaceae)* |  | *Syzygium aromaticum* (L.) Merr. & L.M.Perry (Myrtaceae) |  |  | *Xylopia aethiopica* (Dunal) A.Rich. (Annonaceae) |
| 279 | *Eucalyptus camaldulensis Dehn. (Myrtaceae)* |  | *Talinum fruticosum* (L.) Juss. (Talinaceae) |  |  | *Zanthoxylum zanthoxyloides* (Lam.) Zepern. & Timler (Rutaceae) |
| 280 | *Eucalyptus globulus Labill. (Myrtaceae)* |  | *Telfairia occidentalis* Hook.fil. (Cucurbitaceae) |  |  | *Zingiber officinale* Roscoe (Zingiberaceae) |
| 281 | *Eugenia nigerina A.Chev. (Myrtaceae)* |  | *Terminalia avicennioides* Guill. & Perr. (Combretaceae) |  |  | *Ziziphus mucronata* Willd. (Rhamnaceae) |
| 282 | *Euphorbia convolvuloides Hochst. ex Benth. (Euphorbiaceae)* |  | *Terminalia catappa* L. (Combretaceae) |  |  |  |
| 283 | *Euphorbia deightonii Croizat (Euphorbiaceae)* |  | *Terminalia ivorensis* A.Chev. (Combretaceae) |  |  |  |
| 284 | *Euphorbia drupifera Thonn. (Euphorbiaceae)* |  | *Terminalia superba* Engl. & Diels (Combretaceae) |  |  |  |
| 285 | *Euphorbia glaucophylla Poir. (Euphorbiaceae)* |  | *Tetrapleura tetraptera* (Schum. and Thonn.) Taub. (Fabaceae) |  |  |  |
| 286 | *Euphorbia heterophylla L. (Euphorbiaceae)* |  | *Theobroma cacao* L. (Malvaceae) |  |  |  |
| 287 | *Euphorbia hirta L. (Euphorbiaceae)* |  | *Thymus vulgaris* L. (Lamiaceae) |  |  |  |
| 288 | *Euphorbia kamerunica Pax (Euphorbiaceae)* |  | *Tithonia diversifolia* (Hemsl.) A.Gray (Asteraceae) |  |  |  |
| 289 | *Euphorbia lateriflora Schumach. (Euphorbiaceae)* |  | *Treculia africana* Decne. ex Trécul (Moraceae) |  |  |  |
| 290 | *Euphorbia poissonii Pax (Euphorbiaceae)* |  | *Trema orientale* (L.) Blume (Cannabaceae) |  |  |  |
| 291 | *Euphorbia unispina N.E.Br. (Euphorbiaceae)* |  | *Trichilia monadelpha* (Thonn.) J.J.de Wilde (Meliaceae) |  |  |  |
| 292 | *Ficus asperifolia Miq. (Moraceae)* |  | *Tridax procumbens* L. (Asteraceae) |  |  |  |
| 293 | *Ficus benjamina L. (Moraceae)* |  | *Triumfetta cordifolia*A.Rich. (Malvaceae) |  |  |  |
| 294 | *Ficus capensis Thunb. (Moraceae)* |  | *Tussilago farfara* L. (Asteraceae) |  |  |  |
| 295 | *Ficus exasperata Vahl (Moraceae)* |  | *Urena lobata* L. (Malvaceae) |  |  |  |
| 296 | *Ficus mucuso Welw. ex Ficalho (Moraceae)* |  | *Uvaria chamae* P. Beauv. (Annonaceae) |  |  |  |
| 297 | *Ficus platyphylla Delile (Moraceae)* |  | *Uvariopsis tripetala* (Baker f.) G.E.Schatz (Annonaceae) |  |  |  |
| 298 | *Ficus polita Vahl (Moraceae)* |  | *Vaccinium myrtillus* L. (Ericaceae) |  |  |  |
| 299 | *Ficus sur Forssk. (Moraceae)* |  | *Vachellia nilotica* (L.) P.J.H.Hurter & Mabb. (Fabaceae) |  |  |  |
| 300 | *Ficus thonningii Blume (Moraceae)* |  | *Valeriana officinalis* L. (Caprifoliaceae) |  |  |  |
| 301 | *Flabellaria paniculata Cav. (Malpighiaceae)* |  | *Verbena officinalis* L. (Verbenaceae) |  |  |  |
| 302 | *Flueggea virosa (Roxb. ex Willd.) Royle (Phyllanthaceae)* |  | *Vernonia amygdalina* Delile (Asteraceae) |  |  |  |
| 303 | *Funtumia africana (Benth.) Stapf (Apocynaceae)* |  | *Viola tricolor* L. (Violaceae) |  |  |  |
| 304 | *Funtumia elastica (Preuss) Stapf (Apocynaceae)* |  | *Vitex doniana* sweet (Lamiaceae) |  |  |  |
| 305 | *Gambeya africana (A.DC.) Pierre (Sapotaceae)* |  | *Ximenia americana* L. (Olacaceae) |  |  |  |
| 306 | *Gambeya albida (G.Don) Aubrév. & Pellegr. (Sapotaceae)* |  | *Xylopia aethiopica* (Dunal) A.Rich. (Annonaceae) |  |  |  |
| 307 | *Garcinia kola Heckel (Clusiaceae)* |  | *Zanthoxylum gilletii* (De Wild.) P.G.Waterman (Rutaceae) |  |  |  |
| 308 | *Gardenia ternifolia Schumach. & Thonn. (Rubiaceae)* |  | *Zanthoxylum zanthoxyloides* (Lam.) Zepern. & Timler (Rutaceae) |  |  |  |
| 309 | *Gladiolus dalenii subsp. Dalenii (Iridaceae)* |  | *Zingiber officinale* Roscoe (Zingiberaceae) |  |  |  |
| 310 | *Gladiolus ferrugineus Goldblatt & J.C.Manning (Iridaceae)* |  |  |  |  |  |
| 311 | *Gliricidia sepium (Jacq.) Kunth (Fabaceae)* |  |  |  |  |  |
| 312 | *Gloriosa superba L. (Colchicaceae)* |  |  |  |  |  |
| 313 | *Glyphaea brevis (Biehler) Monach. (Malvaceae)* |  |  |  |  |  |
| 314 | *Gmelina arborea Roxb. ex Sm. (Lamiaceae)* |  |  |  |  |  |
| 315 | *Gnetum africanum Welw. (Gnetaceae)* |  |  |  |  |  |
| 316 | *Gomphrena celosioides Mart. (Amaranthaceae)* |  |  |  |  |  |
| 317 | *Gossypium arboreum L. (Malvaceae)* |  |  |  |  |  |
| 318 | *Gossypium barbadense L. (Malvaceae)* |  |  |  |  |  |
| 319 | *Gossypium hirsutum L. (Malvaceae)* |  |  |  |  |  |
| 320 | *Greenwayodendron suaveolens (Engl. & Diels) Verdc. (Annonaceae)* |  |  |  |  |  |
| 321 | *Grewia carpinifolia Juss. (Malvaceae)* |  |  |  |  |  |
| 322 | *Grewia mollis Juss. (Malvaceae)* |  |  |  |  |  |
| 323 | *Grewia pubescens P.Beauv. (Malvaceae)* |  |  |  |  |  |
| 324 | *Grewia venusta Fresen. (Malvaceae)* |  |  |  |  |  |
| 325 | *Griffonia simplicifolia (Vahl ex DC.) Baill. (Fabaceae)* |  |  |  |  |  |
| 326 | *Grona adscendens (Sw.) H.Ohashi & K.Ohashi (Fabaceae)* |  |  |  |  |  |
| 327 | *Guilandina bonduc L. (Fabaceae)* |  |  |  |  |  |
| 328 | *Gymnanthemum amygdalinum (Delile) Sch.Bip. ex Walp. (Asteraceae)* |  |  |  |  |  |
| 329 | *Harrisonia abyssinica Oliv. (Rutaceae)* |  |  |  |  |  |
| 330 | *Harungana madagascariensis Lam. ex Poir. (Hypericaceae)* |  |  |  |  |  |
| 331 | *Helianthus annuus L. (Asteraceae)* |  |  |  |  |  |
| 332 | *Helictonema velutinum (Afzel.) R.Wilczek (Celastraceae)* |  |  |  |  |  |
| 333 | *Heliotropium indicum L. (Boraginaceae)* |  |  |  |  |  |
| 334 | *Heterotis rotundifolia (Sm.) Jacq.-Fél. (Melastomataceae)* |  |  |  |  |  |
| 335 | *Hexasepalum sarmentosum (Sw.) Delprete & J.H.Kirkbr. (Rubiaceae)* |  |  |  |  |  |
| 336 | *Hexasepalum scandens (Sw.) J.H.Kirkbr. & Delprete (Rubiaceae)* |  |  |  |  |  |
| 337 | *Hibiscus acetosella Welw. ex Hiern (Malvaceae)* |  |  |  |  |  |
| 338 | *Hibiscus cannabinus L. (Malvaceae)* |  |  |  |  |  |
| 339 | *Hibiscus lunariifolius Willd. (Malvaceae)* |  |  |  |  |  |
| 340 | *Hibiscus sabdariffa L. (Malvaceae)* |  |  |  |  |  |
| 341 | *Hibiscus surattensis L. (Malvaceae)* |  |  |  |  |  |
| 342 | *Hibiscus × rosa-sinensis L. (Malvaceae)* |  |  |  |  |  |
| 343 | *Hildegardia barteri (Mast.) Kosterm. (Malvaceae)* |  |  |  |  |  |
| 344 | *Holarrhena floribunda (G.Don) T.Durand & Schinz (Apocynaceae)* |  |  |  |  |  |
| 345 | *Hordeum vulgare L. (Poaceae)* |  |  |  |  |  |
| 346 | *Hoslundia opposita Vahl (Lamiaceae)* |  |  |  |  |  |
| 347 | *Hunteria umbellata (K.Schum.) Hallier f. (Apocynaceae)* |  |  |  |  |  |
| 348 | *Hybanthus enneaspermus (L.) F.Muell. (Violaceae)* |  |  |  |  |  |
| 349 | *Hygrophila auriculata (Schumach.) Heine (Acanthaceae)* |  |  |  |  |  |
| 350 | *Hymenocardia acida Tul. (Phyllanthaceae)* |  |  |  |  |  |
| 351 | *Icacina trichantha Oliv. (Icacinaceae)* |  |  |  |  |  |
| 352 | *Imperata cylindrica (L.) Raeusch. (Poaceae)* |  |  |  |  |  |
| 353 | *Indigofera arrecta Hochst. ex A.Rich. (Fabaceae)* |  |  |  |  |  |
| 354 | *Indigofera hirsuta L. (Fabaceae)* |  |  |  |  |  |
| 355 | *Indigofera macrophylla Schumach. (Fabaceae)* |  |  |  |  |  |
| 356 | *Indigofera pilosa Poir. (Fabaceae)* |  |  |  |  |  |
| 357 | *Ipomoea asarifolia (Desr.) Roem. & Schult. (Convolvulaceae)* |  |  |  |  |  |
| 358 | *Ipomoea batatas (L.) Lam. (Convolvulaceae)* |  |  |  |  |  |
| 359 | *Ipomoea involucrata P.Beauv. (Convolvulaceae)* |  |  |  |  |  |
| 360 | *Ipomoea mauritiana Jacq. (Convolvulaceae)* |  |  |  |  |  |
| 361 | *Irvingia gabonensis (Aubry-Lecomte ex O'Rorke) Baill. (Irvingiaceae)* |  |  |  |  |  |
| 362 | *Ixora coccinea L. (Rubiaceae)* |  |  |  |  |  |
| 363 | *Jateorhiza macrantha (Hook.f.) Exell & Mendonça (Menispermaceae)* |  |  |  |  |  |
| 364 | *Jatropha curcas L. (Euphorbiaceae)* |  |  |  |  |  |
| 365 | *Jatropha gossypifolia L. (Euphorbiaceae)* |  |  |  |  |  |
| 366 | *Jatropha multifida L. (Euphorbiaceae)* |  |  |  |  |  |
| 367 | *Justicia ladanoides Lam. (Acanthaceae)* |  |  |  |  |  |
| 368 | *Kalanchoe crenata (Andrews) Haw. (Crassulaceae)* |  |  |  |  |  |
| 369 | *Kalanchoe pinnata (Lam.) Pers. (Crassulaceae)* |  |  |  |  |  |
| 370 | *Khaya grandifoliola C.DC. (Meliaceae)* |  |  |  |  |  |
| 371 | *Khaya ivorensis A.Chev. (Meliaceae)* |  |  |  |  |  |
| 372 | *Khaya senegalensis (Desr.) A.Juss.) (Meliaceae)* |  |  |  |  |  |
| 373 | *Kigelia africana (Lam.) Benth. (Bignoniaceae)* |  |  |  |  |  |
| 374 | *Klainedoxa gabonensis Pierre ex Engl. (Irvingiaceae)* |  |  |  |  |  |
| 375 | *Laccosperma secundiflorum (P.Beauv.) Kuntze (Arecaceae)* |  |  |  |  |  |
| 376 | *Lactuca inermis Forssk. (Asteraceae)* |  |  |  |  |  |
| 377 | *Lagenaria breviflora (Benth.) Roberty (Cucurbitaceae)* |  |  |  |  |  |
| 378 | *Lagenaria siceraria (Molina) Standl. (Cucurbitaceae)* |  |  |  |  |  |
| 379 | *Lagerstroemia speciosa (L.) Pers. (Lythraceae)* |  |  |  |  |  |
| 380 | *Landolphia dulcis (Sabine ex G.Don) Pichon (Apocynaceae)* |  |  |  |  |  |
| 381 | *Landolphia hirsuta (Hua) Pichon (Apocynaceae)* |  |  |  |  |  |
| 382 | *Lannea acida A.Rich. (Anacardiaceae)* |  |  |  |  |  |
| 383 | *Lannea egregia Engl. & K.Krause (Anacardiaceae)* |  |  |  |  |  |
| 384 | *Lannea kerstingii Engl. & K.Krause (Anacardiaceae)* |  |  |  |  |  |
| 385 | *Lannea nigritana (Scott Elliot) Keay (Anacardiaceae)* |  |  |  |  |  |
| 386 | *Lannea welwitschii (Hiern) Engl. (Anacardiaceae)* |  |  |  |  |  |
| 387 | *Lantana camara L. (Verbenaceae)* |  |  |  |  |  |
| 388 | *Laportea aestuans (L.) Chew (Urticaceae)* |  |  |  |  |  |
| 389 | *Launaea taraxacifolia (Willd.) Amin ex C.Jeffrey (Asteraceae)* |  |  |  |  |  |
| 390 | *Lawsonia inermis L. (Lythraceae)* |  |  |  |  |  |
| 391 | *Leptoderris micrantha Dunn (Fabaceae)* |  |  |  |  |  |
| 392 | *Leucaena leucocephala (Lam.) de Wit (Fabaceae)* |  |  |  |  |  |
| 393 | *Lippia multiflora Moldenke (Verbenaceae)* |  |  |  |  |  |
| 394 | *Lonchocarpus cyanescens (Schumach. & Thonn.) Benth. (Fabaceae)* |  |  |  |  |  |
| 395 | *Lonchocarpus sericeus (Poir.) Kunth ex DC. (Fabaceae)* |  |  |  |  |  |
| 396 | *Lophira alata Banks ex C.F.Gaertn. (Ochnaceae)* |  |  |  |  |  |
| 397 | *Lophira lanceolata Tiegh. ex Keay (Ochnaceae)* |  |  |  |  |  |
| 398 | *Loranthus L. Spp (Loranthaceae)* |  |  |  |  |  |
| 399 | *Lovoa trichilioides Harms (Meliaceae)* |  |  |  |  |  |
| 400 | *Ludwigia abyssinica A.Rich. (Onagraceae)* |  |  |  |  |  |
| 401 | *Ludwigia hyssopifolia (G.Don) Exell (Onagraceae)* |  |  |  |  |  |
| 402 | *Ludwigia peruviana (L.) H.Hara (Onagraceae)* |  |  |  |  |  |
| 403 | *Luffa cylindrica (L.) M.Roem. (Cucurbitaceae)* |  |  |  |  |  |
| 404 | *Macaranga barteri Müll.Arg. (Euphorbiaceae)* |  |  |  |  |  |
| 405 | *Macrosphyra longistyla (DC.) Hook.f. ex Hiern (Rubiaceae)* |  |  |  |  |  |
| 406 | *Malacantha alnifolia (Baker) Pierre (Sapotaceae)* |  |  |  |  |  |
| 407 | *Mallotus oppositifolius (Geiseler) Müll.Arg. (Euphorbiaceae)* |  |  |  |  |  |
| 408 | *Malvastrum coromandelianum (L.) Garcke (Malvaceae)* |  |  |  |  |  |
| 409 | *Mammea africana Sabine (Calophyllaceae)* |  |  |  |  |  |
| 410 | *Mangifera indica L. ( Anacardiaceae)* |  |  |  |  |  |
| 411 | *Manihot esculenta Crantz (Euphorbiaceae)* |  |  |  |  |  |
| 412 | *Manihot glaziovii Müll.Arg. (Euphorbiaceae)* |  |  |  |  |  |
| 413 | *Mansonia altissima (A.Chev.) A.Chev. (Malvaceae)* |  |  |  |  |  |
| 414 | *Margaritaria discoidea (Baill.) G.L.Webster (Phyllanthaceae)* |  |  |  |  |  |
| 415 | *Markhamia tomentosa (Benth.) K.Schum. ex Engl. (Bignoniaceae)* |  |  |  |  |  |
| 416 | *Marrubium vulgare L. (Lamiaceae)* |  |  |  |  |  |
| 417 | *Marsdenia latifolia (Benth.) K.Schum. (Apocynaceae)* |  |  |  |  |  |
| 418 | *Massularia acuminata (G.Don) Bullock ex Hoyle (Rubiaceae)* |  |  |  |  |  |
| 419 | *Melanthera scandens (Schumach. & Thonn.) Roberty (Asteraceae)* |  |  |  |  |  |
| 420 | *Melia azedarach L. (Meliaceae)* |  |  |  |  |  |
| 421 | *Melothria sphaerocarpa (Cogn.) H.Schaef. & S.S.Renner (Cucurbitaceae)* |  |  |  |  |  |
| 422 | *Merremia pterygocaulos (Choisy) Hallier f. (Convolvulaceae)* |  |  |  |  |  |
| 423 | *Mesosphaerum pectinatum (L.) Kuntze (Lamiaceae)* |  |  |  |  |  |
| 424 | *Mesosphaerum suaveolens (L.) Kuntze (Lamiaceae)* |  |  |  |  |  |
| 425 | *Mezoneuron benthamianum Baill. (Fabaceae)* |  |  |  |  |  |
| 426 | *Microdesmis puberula Hook.f. ex Planch. (Pandaceae)* |  |  |  |  |  |
| 427 | *Microstachys chamaelea (L.) Müll.Arg. (Euphorbiaceae)* |  |  |  |  |  |
| 428 | *Milicia excelsa (Welw.) C.C.Berg (Moraceae)* |  |  |  |  |  |
| 429 | *Millettia thonningii (Schumach. & Thonn.) Baker (Fabaceae)* |  |  |  |  |  |
| 430 | *Mimosa pigra L. (Fabaceae)* |  |  |  |  |  |
| 431 | *Mimosa pudica L. (Fabaceae)* |  |  |  |  |  |
| 432 | *Mitragyna ciliata Aubrév. & Pellegr. (Rubiaceae)* |  |  |  |  |  |
| 433 | *Mitragyna inermis (Willd.) Kuntze (Rubiaceae)* |  |  |  |  |  |
| 434 | *Momordica angustisepala Harms (Cucurbitaceae)* |  |  |  |  |  |
| 435 | *Momordica balsamina L. (Cucurbitaceae)* |  |  |  |  |  |
| 436 | *Momordica cabrae (Cogn.) C.Jeffrey (Cucurbitaceae)* |  |  |  |  |  |
| 437 | *Momordica charantia L. (Cucurbitaceae)* |  |  |  |  |  |
| 438 | *Momordica foetida Schumach. (Cucurbitaceae)* |  |  |  |  |  |
| 439 | *Mondia whitei (Hook.f.) Skeels (Apocynaceae)* |  |  |  |  |  |
| 440 | *Monodora myristica (Gaertn.) Dunal (Annonaceae)* |  |  |  |  |  |
| 441 | *Monoon longifolium (Sonn.) B.Xue & R.M.K.Saunders (Annonaceae)* |  |  |  |  |  |
| 442 | *Morinda lucida Benth. (Rubiaceae)* |  |  |  |  |  |
| 443 | *Morinda morindoides (Baker) Milne-Redh. (Rubiaceae)* |  |  |  |  |  |
| 444 | *Moringa oleifera Lam. (Moringaceae)* |  |  |  |  |  |
| 445 | *Motandra paniculata (Poir.) I.M.Turner (Apocynaceae)* |  |  |  |  |  |
| 446 | *Mucuna pruriens (L.) DC. (Fabaceae)* |  |  |  |  |  |
| 447 | *Mucuna sloanei Fawc. & Rendle (Fabaceae)* |  |  |  |  |  |
| 448 | *Musa ×sapientum L. (Musaceae)* |  |  |  |  |  |
| 449 | *Musa acuminata Colla (Musaceae)* |  |  |  |  |  |
| 450 | *Musa x paradisiaca L. (Musaceae)* |  |  |  |  |  |
| 451 | *Musanga cecropioides R.Br. ex Tedlie (Urticaceae)* |  |  |  |  |  |
| 452 | *Mussaenda elegans Schumach. & Thonn. (Rubiaceae)* |  |  |  |  |  |
| 453 | *Myrianthus arboreus P.Beauv. (Urticaceae)* |  |  |  |  |  |
| 454 | *Napoleonaea imperialis P.Beauv. (Lecythidaceae)* |  |  |  |  |  |
| 455 | *Nauclea diderrichii (De Wild.) Merr. (Rubiaceae)* |  |  |  |  |  |
| 456 | *Nauclea latifolia Sm. (Rubiaceae)* |  |  |  |  |  |
| 457 | *Neocarya macrophylla (Sabine) Prance (Chrysobalanaceae)* |  |  |  |  |  |
| 458 | *Neocarya macrophylla (Sabine) Prance (Chrysobalanaceae)* |  |  |  |  |  |
| 459 | *Neonauclea excelsa (Blume) Merr. (Rubiaceae)* |  |  |  |  |  |
| 460 | *Nerium oleander L. (Apocynaceae)* |  |  |  |  |  |
| 461 | *Nesogordonia papaverifera (A.Chev.) Capuron ex N.Hallé (Malvaceae)* |  |  |  |  |  |
| 462 | *Newbouldia laevis (Beauverd) Seem. (Bignoniaceae)* |  |  |  |  |  |
| 463 | *Nicotiana tabacum L. (Solanaceae)* |  |  |  |  |  |
| 464 | *Nigella sativa L. (Ranunculaceae)* |  |  |  |  |  |
| 465 | *Nymphaea lotus L. (Nymphaeaceae)* |  |  |  |  |  |
| 466 | *Ocimum americanum L. (Lamiaceae)* |  |  |  |  |  |
| 467 | *Ocimum basilicum L. (Lamiaceae)* |  |  |  |  |  |
| 468 | *Ocimum gratissimum L. (Lamiaceae )* |  |  |  |  |  |
| 469 | *Odyendea klaineana (Pierre) Engl. (Simaroubaceae)* |  |  |  |  |  |
| 470 | *Olax subscorpioidea Oliv. (Olacaceae)* |  |  |  |  |  |
| 471 | *Oncoba spinosa Forssk. (Salicaceae)* |  |  |  |  |  |
| 472 | *Oplismenus hirtellus (L.) P.Beauv. (Poaceae)* |  |  |  |  |  |
| 473 | *Opuntia tuna (L.) Mill. (Cactaceae)* |  |  |  |  |  |
| 474 | *Ouret lanata (L.) Kuntze (Amaranthaceae)* |  |  |  |  |  |
| 475 | *Oxyanthus formosus Hook.f. (Rubiaceae)* |  |  |  |  |  |
| 476 | *Oxytenanthera abyssinica (A.Rich.) Munro (Poaceae)* |  |  |  |  |  |
| 477 | *Pachyelasma tessmannii (Harms) Harms (Fabaceae)* |  |  |  |  |  |
| 478 | *Pachylobus edulis G.Don (Burseraceae)* |  |  |  |  |  |
| 479 | *Palisota hirsuta (Thunb.) K.Schum. (Commelinaceae)* |  |  |  |  |  |
| 480 | *Parinari congensis Didr. (Chrysobalanaceae)* |  |  |  |  |  |
| 481 | *Parinari curatellifolia Planch. ex Benth. (Rosaceae)* |  |  |  |  |  |
| 482 | *Parkia biglobosa (Jacq.) R.Br. ex G.Don (Fabaceae)* |  |  |  |  |  |
| 483 | *Passiflora foetida L. (Passifloraceae)* |  |  |  |  |  |
| 484 | *Paullinia pinnata L. (Sapindaceae)* |  |  |  |  |  |
| 485 | *Peltophorum pterocarpum (DC.) Backer ex K.Heyne (Fabaceae)* |  |  |  |  |  |
| 486 | *Pennisetum purpureum Schumach. (Poaceae)* |  |  |  |  |  |
| 487 | *Pentaclethra macrophylla Benth. (Fabaceae)* |  |  |  |  |  |
| 488 | *Peperomia pellucida (L.) Kunth (Piperaceae)* |  |  |  |  |  |
| 489 | *Pergularia daemia (Forssk.) Chiov. (Apocynaceae)* |  |  |  |  |  |
| 490 | *Perotis indica (L.) Kuntze (Poaceae)* |  |  |  |  |  |
| 491 | *Persea americana Mill. (Lauraceae)* |  |  |  |  |  |
| 492 | *Petiveria alliacea L. (Petiveriaceae)* |  |  |  |  |  |
| 493 | *Phaseolus vulgaris L. (Fabaceae)* |  |  |  |  |  |
| 494 | *Phaulopsis ciliata (Willd.) Hepper (Acanthaceae)* |  |  |  |  |  |
| 495 | *Phyllanthus amarus Schumach. & Thonn. (Phyllanthaceae)* |  |  |  |  |  |
| 496 | *Phyllanthus muellerianus (Kuntze) Exell (Phyllanthaceae)* |  |  |  |  |  |
| 497 | *Phyllanthus niruri L. (Phyllanthaceae)* |  |  |  |  |  |
| 498 | *Phyllanthus nummulariifolius Poir. (Phyllanthaceae)* |  |  |  |  |  |
| 499 | *Physalis angulata L. (Solanaceae)* |  |  |  |  |  |
| 500 | *Physostigma venenosum Balf. (Fabaceae)* |  |  |  |  |  |
| 501 | *Picralima nitida (Stapf) T.Durand & H.Durand (Apocynaceae)* |  |  |  |  |  |
| 502 | *Piliostigma reticulatum (DC.) Hochst. (Fabaceae)* |  |  |  |  |  |
| 503 | *Piliostigma thonningii (Schumach.) Milne-Redh. (Fabaceae)* |  |  |  |  |  |
| 504 | *Pinus caribaea Morelet (Pinaceae)* |  |  |  |  |  |
| 505 | *Piper nigrum L. (Piperaceae)* |  |  |  |  |  |
| 506 | *Piper umbellatum L. (Piperaceae)* |  |  |  |  |  |
| 507 | *Piper guineense Schumach. & Thonn. (Piperaceae)* |  |  |  |  |  |
| 508 | *Piptadeniastrum africanum (Hook.f.) (Fabaceae)* |  |  |  |  |  |
| 509 | *Pistia stratiotes L. (Araceae)* |  |  |  |  |  |
| 510 | *Platostoma africanum P.Beauv. (Lamiaceae)* |  |  |  |  |  |
| 511 | *Platycerium alcicorne (P.Willemet) Desv. (Polypodiaceae)* |  |  |  |  |  |
| 512 | *Pleiocarpa pycnantha (K.Schum.) Stapf (Apocynaceae)* |  |  |  |  |  |
| 513 | *Pleioceras barteri Baill. (Apocynaceae)* |  |  |  |  |  |
| 514 | *Pleurolobus gangeticus (L.) J.St.-Hil. ex H.Ohashi & K.Ohashi (Fabaceae)* |  |  |  |  |  |
| 515 | *Plukenetia conophora Müll.Arg. (Euphorbiaceae)* |  |  |  |  |  |
| 516 | *Plumbago zeylanica L. (Plumbaginaceae)* |  |  |  |  |  |
| 517 | *Polhillides velutina (Willd.) H.Ohashi & K.Ohashi (Fabaceae)* |  |  |  |  |  |
| 518 | *Portulaca oleracea L. (Portulacaceae)* |  |  |  |  |  |
| 519 | *Pouzolzia guineensis Benth. (Urticaceae)* |  |  |  |  |  |
| 520 | *Prosopis africana (Guill. & Perr.) Taub. (Fabaceae)* |  |  |  |  |  |
| 521 | *Pseudocedrela kotschyi Harms (Meliaceae)* |  |  |  |  |  |
| 522 | *Psidium guajava L. (Myrtaceae)* |  |  |  |  |  |
| 523 | *Psorospermum febrifugum Spach (Hypericaceae)* |  |  |  |  |  |
| 524 | *Psychotria brassii Hiern (Rubiaceae)* |  |  |  |  |  |
| 525 | *Pterocarpus erinaceus Poir. (Fabaceae)* |  |  |  |  |  |
| 526 | *Pterocarpus osun Craib (Fabaceae)* |  |  |  |  |  |
| 527 | *Pterocarpus santalinoides L'Hér. ex DC. (Fabaceae)* |  |  |  |  |  |
| 528 | *Pterygota macrocarpa K.Schum. (Malvaceae)* |  |  |  |  |  |
| 529 | *Pupalia lappacea (L.) Juss. (Amaranthaceae)* |  |  |  |  |  |
| 530 | *Pycnanthus angolensis (Welw.) Warb. (Myristicaceae)* |  |  |  |  |  |
| 531 | *Pyrenacantha staudtii (Engl.) Engl. (Icacinaceae)* |  |  |  |  |  |
| 532 | *Raphia farinifera (Gaertn.) Hyl. (Arecaceae)* |  |  |  |  |  |
| 533 | *Raphia hookeri G.Mann & H.Wendl. (Arecaceae)* |  |  |  |  |  |
| 534 | *Rauvolfia vomitoria Wennberg (Apocynaceae)* |  |  |  |  |  |
| 535 | *Reissantia indica (Willd.) N.Hallé (Celastraceae)* |  |  |  |  |  |
| 536 | *Rhaphiostylis beninensis (Hook.f. ex Planch.) Planch. ex Benth. (Metteniusaceae)* | |  |  |  |  |
| 537 | *Rhodognaphalon brevicuspe (Sprague) Roberty (Malvaceae)* |  |  |  |  |  |
| 538 | *Rhoicissus tridentata (L.f.) Wild & R.B.Drumm. (Vitaceae)* |  |  |  |  |  |
| 539 | *Ricinodendron heudelotii (Baill.) Heckel (Euphorbiaceae)* |  |  |  |  |  |
| 540 | *Ricinus communis L. (Euphorbiaceae)* |  |  |  |  |  |
| 541 | *Rinorea dentata (P.Beauv.) Kuntze (Violaceae)* |  |  |  |  |  |
| 542 | *Rothmannia longiflora Salisb. (Rubiaceae)* |  |  |  |  |  |
| 543 | *Rourea coccinea (Schumach. & Thonn.) Benth. (Connaraceae)* |  |  |  |  |  |
| 544 | *Rytigynia nigerica (S.Moore) Robyns (Rubiaceae)* |  |  |  |  |  |
| 545 | *Rytigynia umbellulata (Hiern) Robyns (Rubiaceae)* |  |  |  |  |  |
| 546 | *Sabicea calycina Benth. (Rubiaceae)* |  |  |  |  |  |
| 547 | *Saccharum officinarum L. (Poaceae)* |  |  |  |  |  |
| 548 | *Salacia pallescens Oliv. (Celastraceae)* |  |  |  |  |  |
| 549 | *Schrebera arborea A.Chev. (Oleaceae)* |  |  |  |  |  |
| 550 | *Schwenckia americana L. (Solanaceae)* |  |  |  |  |  |
| 551 | *Scleria depressa (C.B.Clarke) Nelmes (Cyperaceae)* |  |  |  |  |  |
| 552 | *Scleria racemosa Poir. (Cyperaceae)* |  |  |  |  |  |
| 553 | *Scoparia dulcis L. (Plantaginaceae)* |  |  |  |  |  |
| 554 | *Secamone afzelii (Roem. & Schult.) K.Schum. (Apocynaceae)* |  |  |  |  |  |
| 555 | *Securidaca longipedunculata Fresen. (Polygalaceae)* |  |  |  |  |  |
| 556 | *Senegalia ataxacantha (DC.) Kyal. & Boatwr. (Fabaceae)* |  |  |  |  |  |
| 557 | *Senegalia senegal (L.) Britton (Fabaceae)* |  |  |  |  |  |
| 558 | *Senna alata (L.) Roxb. (Fabaceae)* |  |  |  |  |  |
| 559 | *Senna hirsuta (L.) H.S.Irwin & Barneby (Fabaceae)* |  |  |  |  |  |
| 560 | *Senna obtusifolia (L.) Irwin & Barneby (Fabaceae)* |  |  |  |  |  |
| 561 | *Senna occidentalis (L.) Link (Fabaceae)* |  |  |  |  |  |
| 562 | *Senna podocarpa (Guill. & Perr.) Lock (Fabaceae)* |  |  |  |  |  |
| 563 | *Senna siamea (Lam.) H.S.Irwin & Barneby (Fabaceae)* |  |  |  |  |  |
| 564 | *Senna tora (L.) Roxb. (Fabaceae)* |  |  |  |  |  |
| 565 | *Sesamum indicum L. (Pedaliaceae)* |  |  |  |  |  |
| 566 | *Sesamum radiatum Thonn. ex Hornem. (Pedaliaceae)* |  |  |  |  |  |
| 567 | *Setaria scandens Schrad. (Poaceae)* |  |  |  |  |  |
| 568 | *Sida acuta Burm.f. (Malvaceae)* |  |  |  |  |  |
| 569 | *Sida cordifolia L. (Malvaceae)* |  |  |  |  |  |
| 570 | *Sida linifolia Juss. ex Cav. (Malvaceae)* |  |  |  |  |  |
| 571 | *Sida urens L. (Malvaceae)* |  |  |  |  |  |
| 572 | *Smilax anceps Willd. (Smilacaceae)* |  |  |  |  |  |
| 573 | *Solanecio biafrae (Oliv. & Hiern) C.Jeffrey (Asteraceae)* |  |  |  |  |  |
| 574 | *Solanum aethiopicum L. (Solanaceae)* |  |  |  |  |  |
| 575 | *Solanum americanum Mill. (Solanaceae)* |  |  |  |  |  |
| 576 | *Solanum pseudocapsicum L. (Solanaceae)* |  |  |  |  |  |
| 577 | *Solanum torvum Sw. (Solanaceae)* |  |  |  |  |  |
| 578 | *Solenostemon monostachyus (P.Beauv.) Roberty (Lamiaceae)* |  |  |  |  |  |
| 579 | *Sorghum bicolor (L.) Moench (Poaceae)* |  |  |  |  |  |
| 580 | *Sorghum bicolor subsp. bicolor (L.) Moench (Poaceae)* |  |  |  |  |  |
| 581 | *Spathodea campanulata P.Beauv. (Bignoniaceae)* |  |  |  |  |  |
| 582 | *Spermacoce ocymoides Burm.f. (Rubiaceae)* |  |  |  |  |  |
| 583 | *Spermacoce ruelliae DC. (Rubiaceae)* |  |  |  |  |  |
| 584 | *Spermacoce verticillata L. (Rubiaceae)* |  |  |  |  |  |
| 585 | *Sphenocentrum jollyanum Pierre (Menispermaceae)* |  |  |  |  |  |
| 586 | *Spigelia anthelmia L. (Loganiaceae)* |  |  |  |  |  |
| 587 | *Spondias mombin L. (Anacardiaceae)* |  |  |  |  |  |
| 588 | *Sporobolus indicus (L.) R.Br. (Poaceae)* |  |  |  |  |  |
| 589 | *Stachytarpheta cayennensis (Rich.) Vahl (Verbenaceae)* |  |  |  |  |  |
| 590 | *Stachytarpheta indica (L.) Vahl (Verbenaceae)* |  |  |  |  |  |
| 591 | *Stachytarpheta mutabilis (Jacq.) Vahl (Verbenaceae)* |  |  |  |  |  |
| 592 | *Staudtia kamerunensis var. gabonensis (Warb.) Fouilloy (Myristicaceae)* |  |  |  |  |  |
| 593 | *Stephania abyssinica (Quart.-Dill. & A.Rich.) Walp. (Menispermaceae)* |  |  |  |  |  |
| 594 | *Sterculia oblonga Mast. (Malvaceae)* |  |  |  |  |  |
| 595 | *Sterculia rhinopetala K.Schum. (Malvaceae)* |  |  |  |  |  |
| 596 | *Sterculia setigera Delile (Malvaceae)* |  |  |  |  |  |
| 597 | *Sterculia tragacantha Lindl. (Malvaceae)* |  |  |  |  |  |
| 598 | *Stereospermum acuminatissimum K.Schum. (Bignoniaceae)* |  |  |  |  |  |
| 599 | *Strombosia grandifolia Hook.fil. ex Benth (Strombosiaceae)* |  |  |  |  |  |
| 600 | *Strophanthus hispidus DC. (Apocynaceae)* |  |  |  |  |  |
| 601 | *Strophanthus sarmentosus DC. (Apocynaceae)* |  |  |  |  |  |
| 602 | *Strychnos nux-vomica L. (Loganiaceae)* |  |  |  |  |  |
| 603 | *Symphonia globulifera L.f. (Clusiaceae)* |  |  |  |  |  |
| 604 | *Synclisia scabrida Miers (Menispermaceae)* |  |  |  |  |  |
| 605 | *Synedrella nodiflora (L.) Gaertn. (Asteraceae)* |  |  |  |  |  |
| 606 | *Synsepalum dulcificum (Schumach. & Thonn.) Daniell (Sapotaceae)* |  |  |  |  |  |
| 607 | *Syzygium aromaticum (L.) Merr. & L.M.Perry (Myrtaceae)* |  |  |  |  |  |
| 608 | *Syzygium guineense (Willd.) DC. (Myrtaceae)* |  |  |  |  |  |
| 609 | *Syzygium jambos (L.) Alston (Myrtaceae)* |  |  |  |  |  |
| 610 | *Syzygium malaccense (L.) Merr. & L.M.Perry (Myrtaceae)* |  |  |  |  |  |
| 611 | *Tacca leontopetaloides (L.) Kuntze (Dioscoreaceae)* |  |  |  |  |  |
| 612 | *Talinum fruticosum (L.) Juss. (Talinaceae)* |  |  |  |  |  |
| 613 | *Tapinanthus globiferus (A.Rich.) Tiegh. (Loranthaceae)* |  |  |  |  |  |
| 614 | *Tectona grandis L.f. (Lamiaceae)* |  |  |  |  |  |
| 615 | *Telfairia occidentalis Hook.fil. (Cucurbitaceae)* |  |  |  |  |  |
| 616 | *Terminalia avicennioides Guill. & Perr. (Combretaceae)* |  |  |  |  |  |
| 617 | *Terminalia catappa L. (Combretaceae)* |  |  |  |  |  |
| 618 | *Terminalia engleri Gere & Boatwr. (Combretaceae)* |  |  |  |  |  |
| 619 | *Terminalia ivorensis A.Chev. (Combretaceae)* |  |  |  |  |  |
| 620 | *Terminalia leiocarpa (DC.) Baill. (Combretaceae)* |  |  |  |  |  |
| 621 | *Terminalia macroptera Guill. & Perr. (Combretaceae)* |  |  |  |  |  |
| 622 | *Terminalia randii Baker f. (Combretaceae)* |  |  |  |  |  |
| 623 | *Terminalia schimperiana Hochst (Combretaceae)* |  |  |  |  |  |
| 624 | *Terminalia superba Engl. & Diels (Combretaceae)* |  |  |  |  |  |
| 625 | *Tetracera alnifolia Willd. (Dilleniaceae)* |  |  |  |  |  |
| 626 | *Tetracera potatoria Afzel. ex G.Don (Dilleniaceae)* |  |  |  |  |  |
| 627 | *Tetrapleura tetraptera (Schum. and Thonn.) Taub. (Fabaceae)* |  |  |  |  |  |
| 628 | *Thaumatococcus daniellii (Benn.) Benth. ex Eichler (Marantaceae)* |  |  |  |  |  |
| 629 | *Theobroma cacao L. (Malvaceae)* |  |  |  |  |  |
| 630 | *Thonningia sanguinea Vahl (Balanophoraceae)* |  |  |  |  |  |
| 631 | *Tithonia diversifolia (Hemsl.) A.Gray (Asteraceae)* |  |  |  |  |  |
| 632 | *Tragia benthamii Baker (Euphorbiaceae)* |  |  |  |  |  |
| 633 | *Treculia africana Decne. ex Trécul (Moraceae)* |  |  |  |  |  |
| 634 | *Trema orientale (L.) Blume (Cannabaceae)* |  |  |  |  |  |
| 635 | *Tribulus terrestris L. (Zygophyllaceae)* |  |  |  |  |  |
| 636 | *Trichilia monadelpha (Thonn.) J.J.de Wilde (Meliaceae)* |  |  |  |  |  |
| 637 | *Triclisia subcordata Oliv. (Menispermaceae)* |  |  |  |  |  |
| 638 | *Tridax procumbens L. (Asteraceae)* |  |  |  |  |  |
| 639 | *Trilepisium madagascariense DC. (Moraceae)* |  |  |  |  |  |
| 640 | *Triplochiton scleroxylon K.Schum. (Malvaceae)* |  |  |  |  |  |
| 641 | *Triumfetta cordifolia A.Rich. (Malvaceae)* |  |  |  |  |  |
| 642 | *Triumfetta rhomboidea Jacq. (Malvaceae)* |  |  |  |  |  |
| 643 | *Uraria picta (Jacq.) Desv. ex DC. (Fabaceae)* |  |  |  |  |  |
| 644 | *Urena lobata L. (Malvaceae)* |  |  |  |  |  |
| 645 | *Uvaria afzelii Scott. Elliot (Annonaceae)* |  |  |  |  |  |
| 646 | *Uvaria chamae P. Beauv. (Annonaceae)* |  |  |  |  |  |
| 647 | *Uvariopsis tripetala (Baker f.) G.E.Schatz (Annonaceae)* |  |  |  |  |  |
| 648 | *Vachellia nilotica (L.) P.J.H.Hurter & Mabb. (Fabaceae)* |  |  |  |  |  |
| 649 | *Vernonia amygdalina Delile (Asteraceae)* |  |  |  |  |  |
| 650 | *Vernonia colorata (Willd.) Drake (Asteraceae)* |  |  |  |  |  |
| 651 | *Vigna racemosa (G.Don) Hutch. & Dalziel ex Baker f. (Fabaceae)* |  |  |  |  |  |
| 652 | *Vigna unguiculata (L.) Walp. (Fabaceae)* |  |  |  |  |  |
| 653 | *Vincetoxicum sylvaticum (Decne.) Kuntze (Apocynaceae)* |  |  |  |  |  |
| 654 | *Viscum album L. (Santalaceae)* |  |  |  |  |  |
| 655 | *Vitellaria paradoxa C.F Gaertn (Sapotaceae)* |  |  |  |  |  |
| 656 | *Vitex agnus-castus L. (Lamiaceae)* |  |  |  |  |  |
| 657 | *Vitex doniana sweet (Lamiaceae)* |  |  |  |  |  |
| 658 | *Vitis vinifera L. (Vitaceae)* |  |  |  |  |  |
| 659 | *Voacanga africana Stapf (Apocynaceae)* |  |  |  |  |  |
| 660 | *Waltheria indica L. (Malvaceae)* |  |  |  |  |  |
| 661 | *Xanthosoma sagittifolium (L.) Schott (Araceae)* |  |  |  |  |  |
| 662 | *Ximenia americana L. (Olacaceae)* |  |  |  |  |  |
| 663 | *Xylopia aethiopica (Dunal) A.Rich. (Annonaceae)* |  |  |  |  |  |
| 664 | *Xylopia quintasii Pierre ex Engl. & Diels (Annonaceae)* |  |  |  |  |  |
| 665 | *Xylopia villosa Chipp (Annonaceae)* |  |  |  |  |  |
| 666 | *Zanthoxylum zanthoxyloides (Lam.) Zepern. & Timler (Rutaceae)* |  |  |  |  |  |
| 667 | *Zapoteca portoricensis (Jacq.) H.M.Hern. (Fabaceae)* |  |  |  |  |  |
| 668 | *Zea mays L. (Poaceae)* |  |  |  |  |  |
| 669 | *Zehneria capillacea (Schumach.) C.Jeffrey (Cucurbitaceae)* |  |  |  |  |  |
| 670 | *Zingiber officinale Roscoe (Zingiberaceae)* |  |  |  |  |  |
| 671 | *Zygotritonia bongensis (Pax) Mildbr. (Iridaceae)* |  |  |  |  |  |
| 672 | *Waltheria indica* L. (Malvaceae) |  |  |  |  |  |
| 673 | *Xanthosoma sagittifolium* (L.) Schott (Araceae) |  |  |  |  |  |
| 674 | *Ximenia americana* L. (Olacaceae) |  |  |  |  |  |
| 675 | *Xylopia aethiopica (Dunal) A.Rich. (Annonaceae)* |  |  |  |  |  |
| 676 | *Xylopia quintasii* Pierre ex Engl. & Diels (Annonaceae) |  |  |  |  |  |
| 677 | *Xylopia villosa* Chipp (Annonaceae) |  |  |  |  |  |
| 678 | *Zanthoxylum zanthoxyloides* (Lam.) Zepern. & Timler (Rutaceae) |  |  |  |  |  |
| 679 | *Zapoteca portoricensis* (Jacq.) H.M.Hern. (Fabaceae) |  |  |  |  |  |
| 680 | *Zea mays* L. (Poaceae) |  |  |  |  |  |
| 681 | *Zehneria capillacea* (Schumach.) C.Jeffrey (Cucurbitaceae) |  |  |  |  |  |
| 682 | *Zingiber officinale* Roscoe (Zingiberaceae) |  |  |  |  |  |
| 683 | *Zygotritonia bongensis* (Pax) Mildbr. (Iridaceae) |  |  |  |  |  |

**Summary of Analysis**

| **Plants per region:** |
| --- |
| SW: 683 species = 70.98% |
| SE: 200 species = 20.77% |
| SS: 309 species = 32.09% |
| NW: 274 species = 28.45% |
| NE: 99 species = 10.28% |
| NC: 281 species = 29.18% |

| **Unique plants per region:** |
| --- |
| SW: 308 species (45.2%) |
| SE: 36 species (18.1%) |
| SS: 64 species (20.8%) |
| NW: 86 species (31.5%) |
| NE: 13 species (13.3%) |
| NC: 39 species (13.9%) |

**Plants common to all regions: 19 plant species**

*Acanthospermum hispidum* DC. (Asteraceae)

*Adansonia* *digitata* L. (Malvaceae)

*Anogeissus* *leiocarpa* (DC.) (Annonaceae)

*Azadirachta* indica A. Juss. (Meliaceae)

*Citrus* *sinensis* (Rutaceae)

*Citrus* × *aurantiifolia* (Christm.) (Rutaceae)

*Cymbopiogon* *citratus* (DC.) (Poaceae)

*Eucalyptus* *camaldulensis* Dehn. (Myrtaceae)

*Gossypium barbadense* L. (Malvaceae)

*Jatropha curcas* L. (Euphorbiaceae)

*Lawsonia inermis* L. (Lythraceae)

*Mangifera indica* L. (Anacardiaceae)

*Moringa oleifera* Lam. (Moringaceae)

*Psidium guajava* L. (Myrtaceae)

*Securidaca longipedunculata* Fresen. (Polygalaceae)

*Vachellia nilotica* (Fabaceae)

*Vernonia amygdalina* Delile (Asteraceae)

*Vitex doniana* sweet (Lamiaceae)

*Ximenia americana* L. (Olacaceae)

**Top 10 dominant plant families across the six (6) regions in Nigeria**

| **Plant families** | **Southwest (SW)** | **Southeast (SE)** | **South-South** | **Northwest (NW)** | **Northwast (NE)** | **North Central (NC)** |
| --- | --- | --- | --- | --- | --- | --- |
| Fabaceae | 91 | 14 | 29 | 47 | 21 | 40 |
| Malvaceae | 46 | 12 | 19 | 15 | 6 | 14 |
| Asteraceae | 32 | 11 | 18 | 9 | 2 | 11 |
| Euphorbiaceae | 31 | 4 | 14 | 11 | 4 | 11 |
| Apocynaceae | 30 | 12 | 11 | 6 | 2 | 12 |
| Rubiaceae | 25 | 6 | 15 | 9 | 2 | 11 |
| Poaceae | 22 | 6 | 7 | 12 | 2 | 10 |
| Combretaceae | 18 | 3 | 5 | 11 | 2 | 10 |
| Lamiaceae | 17 | 5 | 13 | 7 | 2 | 9 |
| Moraceae | 16 | 4 | 6 | 11 | 4 | 7 |
